# Heterocyclic Iminoquinones and Quinones from the National Cancer Institute (NCI, USA) COMPARE Analysis

**DOI:** 10.3390/molecules28135202

**Published:** 2023-07-04

**Authors:** Naemah Haji, Masoma Faizi, Panayiotis A. Koutentis, Michael P. Carty, Fawaz Aldabbagh

**Affiliations:** 1 Department of Pharmacy, School of Life Sciences, Pharmacy and Chemistry, Kingston University, Penrhyn Road, Kingston upon Thames, London KT1 2EE, UK; naemah.haji@gmail.com (N.H.); faizi.masoma2@gmail.com (M.F.); 2 Department of Chemistry, University of Cyprus, P.O. Box 20537, Nicosia 1678, Cyprus; koutenti@ucy.ac.cy; 3 School of Biological and Chemical Sciences, University of Galway, University Road, H91 TK33 Galway, Ireland; michael.carty@universityofgalway.ie

**Keywords:** bioreduction, heterocycles, natural products, NQO1, reductases, synthesis, thioredoxin

## Abstract

This review uses the National Cancer Institute (NCI) COMPARE program to establish an extensive list of heterocyclic iminoquinones and quinones with similarities in differential growth inhibition patterns across the 60-cell line panel of the NCI Developmental Therapeutics Program (DTP). Many natural products and synthetic analogues are revealed as potential NAD(P)H:quinone oxidoreductase 1 (NQO1) substrates, through correlations to dipyridoimidazo[5,4-*f*]benzimidazoleiminoquinone (DPIQ), and as potential thioredoxin reductase (TrxR) inhibitors, through correlations to benzo[1,2,4]triazin-7-ones and pleurotin. The strong correlation to NQO1 infers the enzyme has a major influence on the amount of the active compound with benzo[*e*]perimidines, phenoxazinones, benz[*f*]pyrido[1,2-*a*]indole-6,11-quinones, seriniquinones, kalasinamide, indolequinones, and furano[2,3-*b*]naphthoquinones, hypothesised as prodrugs. Compounds with very strong correlations to known TrxR inhibitors had inverse correlations to the expression of both reductase enzymes, NQO1 and TrxR, including naphtho[2,3-*b*][1,4]oxazepane-6,11-diones, benzo[*a*]carbazole-1,4-diones, pyranonaphthoquinones (including kalafungin, nanaomycin A, and analogues of griseusin A), and discorhabdin C. Quinoline-5,8-dione scaffolds based on streptonigrin and lavendamycin can correlate to either reductase. Inhibitors of TrxR are not necessarily (imino)quinones, e.g., parthenolides, while oxidising moieties are essential for correlations to NQO1, as with the mitosenes. Herein, an overview of synthetic methods and biological activity of each family of heterocyclic imino(quinone) is provided.

## 1. Introduction

The National Cancer Institute’s (NCI, USA) Developmental Therapeutics Program (DTP) has supported the development of numerous clinical anticancer agents, including synthetic and natural compounds, vaccines, and antibodies [1,2,3,4]. The NCI human cancer 60-cell line panel consists of the nine major histological tissue types, allowing high throughput screening of thousands of compounds and natural product extracts each month, using the same assay under strictly identical conditions. Compounds showing high toxicity and variable patterns at an initial single dose (10 *µ*M) may be selected for five dose *in vitro* testing. The *in vitro* mean growth (inhibition) data against each cell line represents a pattern, or “fingerprint”, for the evaluated compound (the seed). The seed is identified using a designated NCI accession number (the Cancer Chemotherapy National Service Center number, NSC number). Paull *et al*. transformed numerical cell line response data into the mean graph format, for visualising differential growth inhibition [5]. After five dose testing, differential growth inhibition is depicted on a log scale by bars (in delta units), which project either side of the mean (e.g., Figures S1–S82, in Supplementary Materials). The COMPARE algorithm is used to rank, in order of similarity to the seed, the activity of compounds in the huge NCI-DTP database, as well as the similarity of the activity of the seed to the expression of key cancer molecular targets in the panel of cell lines [1,2,3,5]. A molecular target is a protein, enzyme, gene, or any other cellular molecule whose presence within the 60-cell line panel has been identified and quantified by the NCI. For some test compounds, a single cellular component may determine activity, while in most cases, cell sensitivity is complex and determined by gene expression, cell signalling, and repair pathways. The similarity of anti-cancer activity patterns to the seed is expressed quantitatively as a Pearson correlation coefficient (PCC). PCCs are between −1 and +1, with −1 indicating a perfect inverse correlation, zero indicating no correlation, and +1 indicating a perfect direct correlation. A PCC of 0.3–0.5 is generally accepted as weak to moderate, 0.5–0.7 as being moderate to strong, and above 0.7 as strong to very strong [1,5,6]. The toxicity evaluation service and COMPARE is available to the public, free of charge, from the NCI website (https://dtp.cancer.gov/, (accessed on 20 October 2022 until 12 May 2023)), and as a new technology platform. Herein, we continue to assess similarities using the 50% growth inhibition (GI_50_) parameter. GI_50_ is defined as the concentration that prevents half the proliferation from baseline compared to the maximal value of the untreated cells. Strong correlations may indicate similarities in the mechanisms of the action of anti-cancer compounds, as well as structure–activity relationships. Two previous drug discovery investigations are revisited that established two diverse heterocyclic iminoquinone scaffolds as potent anti-cancer agents [7,8,9,10,11]. Among the tools utilised to ascertain molecular targets was the NCI COMPARE program [7,10,11], which now reveals a more extensive list of correlated compounds. Other high-throughput compound screening programs are not utilised in this review [3,4].

**Scheme 1:**
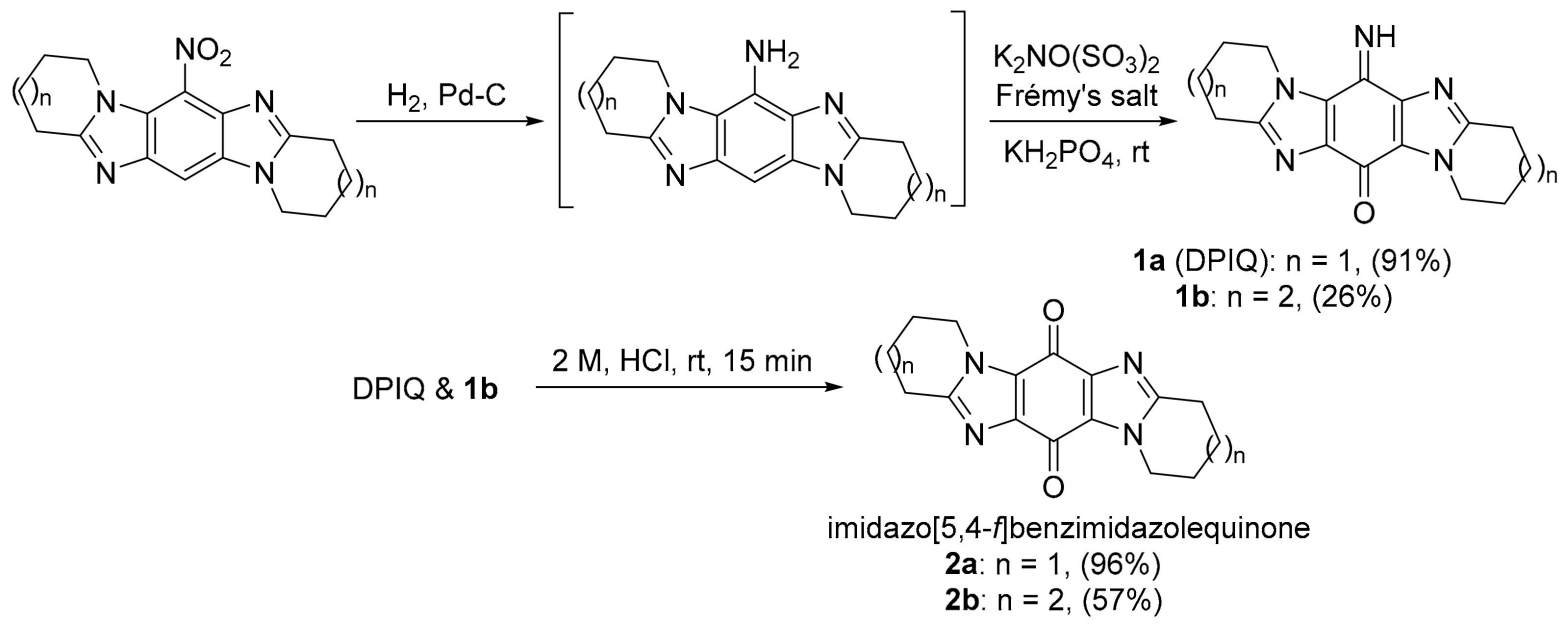
Iminoquinones via the synthesis of imidazo[5,4-*f*]benzimidazolequinones [7,9].

Aldabbagh and co-workers introduced ring-fused imidazo[5,4-*f*]benzimidazolequinones, e.g., **2a** and **2b**, with iminoquinones **1a** and **1b** isolated from the Frémy oxidation of the amine intermediate in the presence of KH_2_PO_4_ acidic buffer (Scheme 1) [7,9].

**Figure 1:**
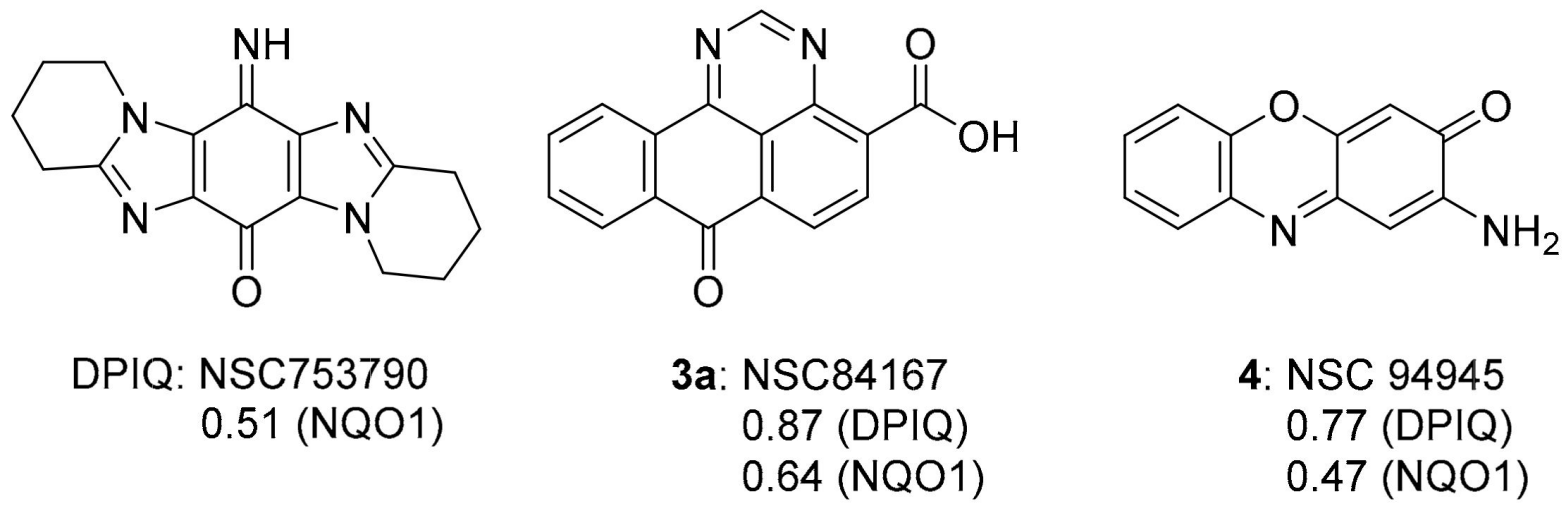
6-Imino-1,2,3,4,8,9,10,11-octahydropyrido[1,2-*a*]pyrido[1’,2’:1,2]imidazo[5,4-*f*]benzimidazole-13-one (DPIQ), **3a** and **4**, and PCCs from COMPARE analysis [7].

The dipyrido-fused iminoquinone **1a** (DPIQ) was isolated in high yield (91%). DPIQ exhibited significant and variable cell growth inhibitory activity against the NCI-60 cell lines, and was selected for five-dose testing [7]. The seven-membered (azepino) analogue **1b** is inactive, and the hydrolysed quinones **2a** and **2b** were less potent than DPIQ [7]. Notably, the isomeric iminoquinone of DPIQ, the imidazo[4,5-*f*]benzimidazole also exhibited low toxicity at the NCI [12]. The COMPARE analysis gave a moderate correlation for DPIQ (PCC = 0.51) to NAD(P)H:quinone oxidoreductase 1 (NQO1, formerly known as DT-diaphorase) [7], an enzyme with heterogeneity of expression across the NCI-60 panel (Figure 1) [13]. The COMPARE analysis of DPIQ against the huge library of synthetic compounds in the NCI-DTP database gave strong correlations in anti-cancer activity to two other heterocyclic iminoquinones **3a** (PCC = 0.87) and **4** (0.77), indicating that the compounds possibly possess similar mechanisms of action. Iminoquinone **3a** (PCC = 0.64) gave a stronger correlation than DPIQ and **4** (0.47) to NQO1 expression in the NCI-DTP 60 cell lines. The higher PCC for **3a** was supported by computational docking at the NQO1 active site, which predicted a higher affinity and a shorter distance for hydride reduction from the isoalloxazine ring of FADH_2_ [7].

**Figure 2:**
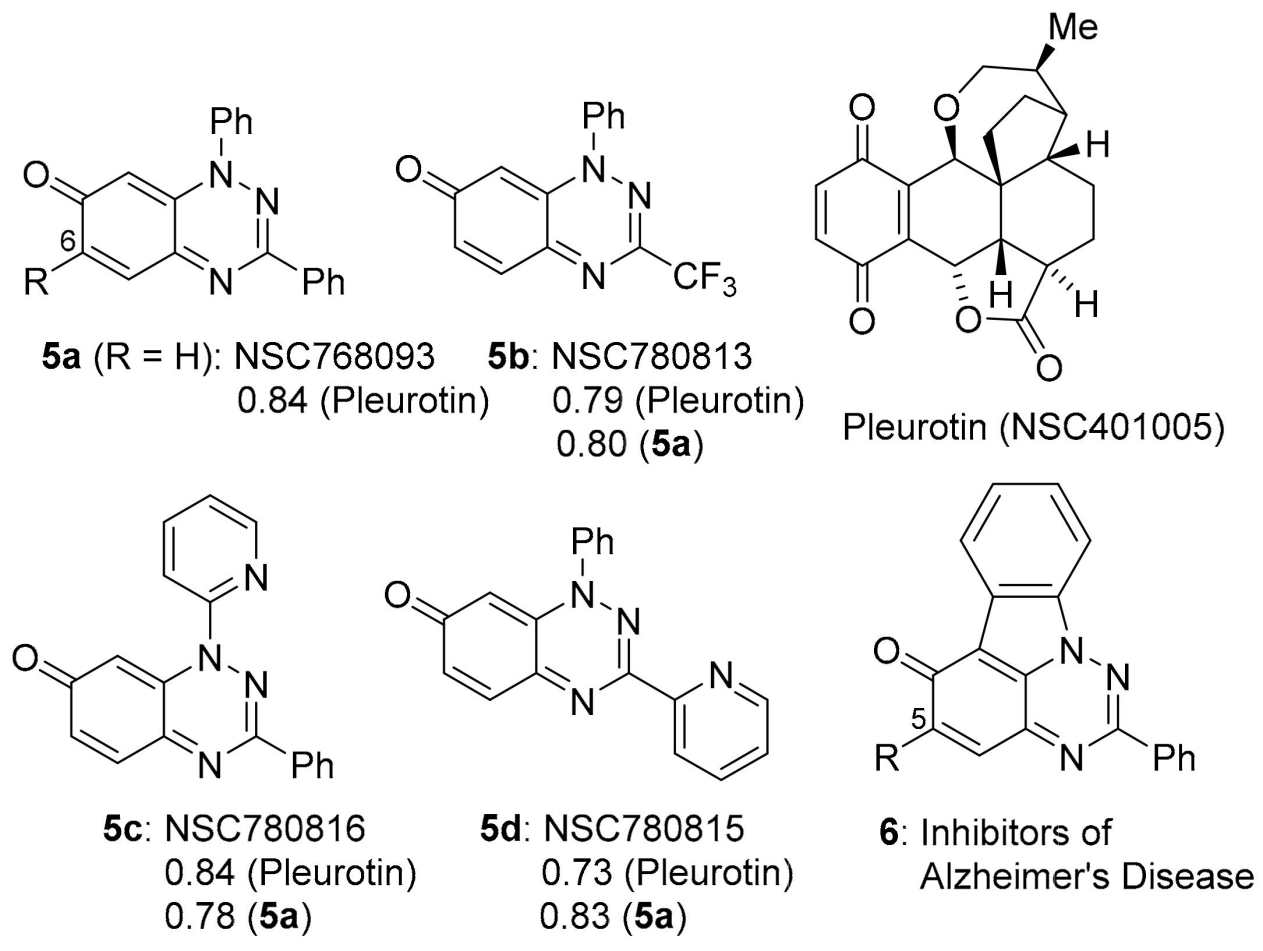
Benzo[1,2,4]triazin-7-ones **5a**–**d** and PCCs to pleurotin [10,11], and anti-AD [1,2,4]triazino[5,6,1-*jk*]carbazol-6-one **6** [14].

A collaboration between the groups of Aldabbagh and Koutentis led to the discovery of anti-cancer activity for the benzo[*e*][1,2,4]triazin-7-ones, e.g., **5a**–**d**, (Figure 2) based on inhibition of the growth of cancer cell lines [10,11]. An earlier report demonstrated the potency of 6-substituted 1,3-diphenylbenzo[1,2,4]triazinones, and 5-substituted [1,2,4]triazino[5,6,1-*jk*]carbazol-6-one derivatives **6**, as multi-target inhibitors for Alzheimer’s disease (AD) [14].

The anti-cancer evaluation studies were stimulated by our interest in the iminoquinone motif, which is common to both DPIQ and the benzo[1,2,4]triazinones (Figure 1 and Figure 2). The benzotriazinones **5a**–**d** showed sufficient potency for selection by the NCI for five-dose testing. The five-dose testing gave the GI_50_ parameter used in the COMPARE analysis. For **5a**–**d**, the PCCs of ~0.8 indicate close to perfect direct correlations to the naturally occurring saturated benzoquinone, pleurotin [10,11]. Pleurotin possesses antibiotic and anti-cancer activities, including the inhibiting of hypoxia-induced factor 1α (HIF-1α) [15]. Pleurotin is a potent irreversible inhibitor of thioredoxin reductase (TrxR), and compounds **5a** and **5b** are reversible inhibitors with *K*_i_ values of 3.90 and 0.78 *µ*M, respectively [10]. The greater enzyme inhibition by **5b** was assumed to be related to the highly electron-withdrawing 3-CF_3_ group increasing the susceptibility to reductive activation and thereby increasing the specificity towards cancer cell lines.

**Scheme 2:**
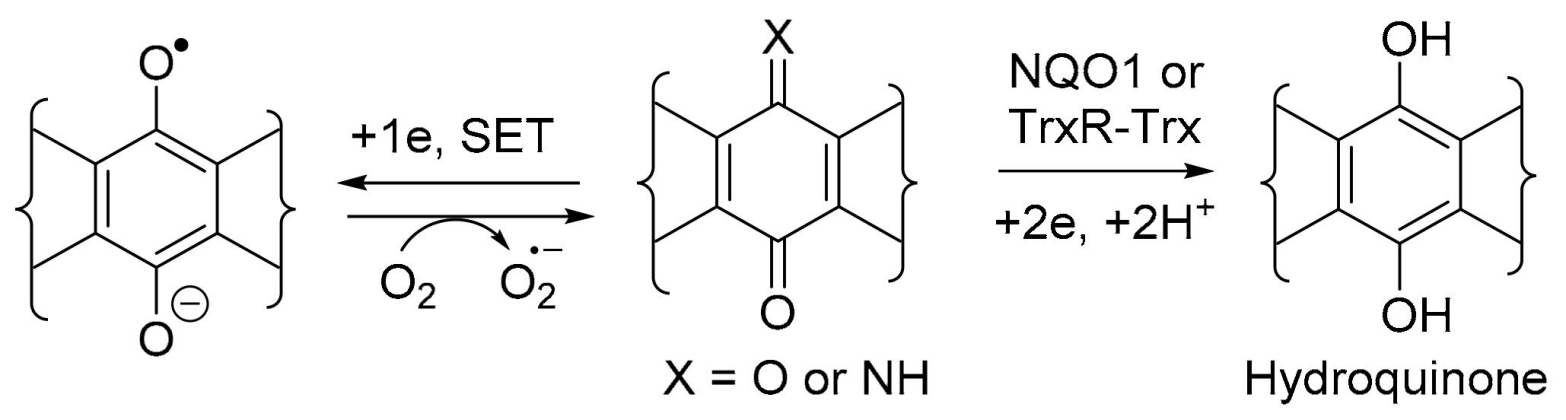
General mechanism for NQO1 and TrxR-Trx quinone detoxification (hydrolysis required for X = NH).

NQO1 and TrxR are obligatory 2-electron reductases involved in regulating the production of toxic reactive oxygen species (ROS), including superoxide (O_2_^●−^) (Scheme 2). Although O_2_^●−^ is used in cells of the immune system and provides a defence against pathogens, excessive amounts are implicated in many physiological disorders, including cancer [16]. The 2-electron reduction overrides the single electron transfer (SET) by detoxifying the quinone, through the formation of a relatively stable aromatic hydroquinone, which is eliminated through the conjugation to glutathione, sulfate, or glucose [17].

**Figure 3:**
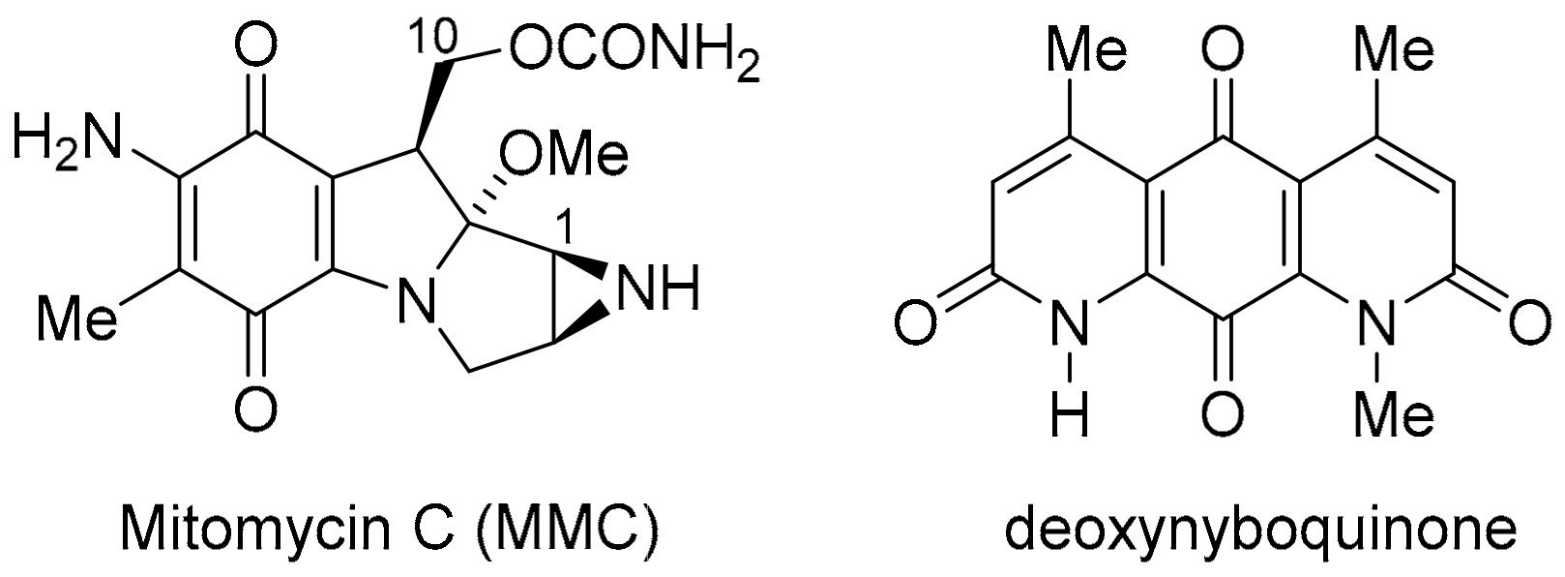
Bioreductive anti-cancer agents.

NQO1 and TrxR, however, are over-expressed in many solid tumours and, thus, are viable molecular targets for cancer therapy [18,19]. In this context, the heterocyclic (imino)quinone is designed as a prodrug to form cytotoxic hydroquinone. For instance, in the case of the archetypical bioreductive quinone prodrug, mitomycin C, cytotoxicity occurs through quinone methide formation at C-1 and C-10, which react with the nucleophilic DNA bases [20] (Figure 3). However, in the case of heterocyclic quinones and iminoquinones, such as DPIQ, which lack obvious reactive sites upon reduction, cell death is likely attributed to ROS formation with simultaneous reoxidation of the hydroquinone to the quinone, as observed in deoxynyboquinone (Figure 3) [21].

Similar to the NQO1 reductive activation mechanism [22], NAD(P)H provides hydride reducing equivalents for transfer via a cascade in the TrxR-Trx (thioredoxin protein) system to the (imino)quinone [19]. Consequently, inhibition of TrxR leads to the accumulation of Trx protein and the activation of ROS-induced apoptosis pathways [16].

We now employ the NCI COMPARE analysis to examine heterocyclic quinones and iminoquinones, specifically focusing on their strong correlations with DPIQ, benzo[1,2,4]triazinone **5a** and pleurotin. The objective of this analysis is to determine whether these correlations in anti-cancer activity patterns reflect actual similarities in mechanisms of action and structure-activity relationships. In addition to assessing the biological activities, we provide descriptions of selected syntheses utilised to obtain each heterocyclic iminoquinone and quinone scaffold. We believe that this review represents the first compilation of heterocyclic compounds derived from a COMPARE analysis.

## 2. Compound Search Methods

Our previous COMPARE analyses were repeated [6,7,10,11,23], and new analyses were carried out under identical conditions to allow comparisons of Pearson correlation coefficients (PCCs). The public COMPARE (https://dtp.cancer.gov/public_compare/, (accessed on 20 October 2022 until 12 May 2023)) was used to access the NCI-60 data, which did not include one-dose experiments. The mean graphs generated are listed along with the PCCs in the Supplementary Materials document (Figures S1–S82). Scifinder^n^ (https://scifinder-n.cas.org, https://dtp.cancer.gov/public_compare/, (accessed on 20 October 2022 until 12 May 2023)) was used to find the relevant literature using chemical structure and citation searches with, where applicable, publication data refinements.

### 2.1. COMPARE Using DPIQ and ***5a*** as the Seed

**Scheme 3:**
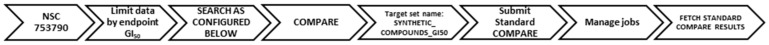
Workflow for COMPARE analysis on DPIQ, as the seed.

The seed NSC numbers for DPIQ (NCS753790) and benzo[1,2,4]triazinone **5a** (NCS768093) were used to search using the COMPARE algorithm screening data limited to GI_50_ end points. A user’s guide is provided below (Scheme 3). The mean graph data was subjected to a standard COMPARE using GI_50_ of the synthetic compound as the target set, with compounds ranked according to the magnitude of the PCCs. The process was repeated using pleurotin (NSC401005) as the seed to confirm similarities in PCCs with **5a**.

### 2.2. COMPARE Using Molecular Target Expression

**Scheme 4:**
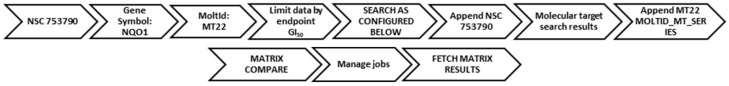
Workflow for COMPARE analysis using NQO1 as the molecular target.

A COMPARE analysis was carried out for the selected compound NSC numbers against the molecular target expression in the NCI-60 (Scheme 4). NAD(P)H:quinone oxidoreductase 1 (NQO1) used the MT22 MOLTID.MT.SERIES, and thioredoxin reductase (TrxR) used the MT143 TXNRD1 MOLTID.MT.SERIES. A PCC was obtained using a matrix COMPARE by appending the GI_50_ cell viability profile of a given NSC number against an appended molecular target expression across the NCI-60.

The strongest correlations to NQO1 (MT22 MOLTID.MT.SERIES) were found by submitting the molecular target expression data to a standard COMPARE using screening data limited to GI_50_ end points. Compounds were listed according to the magnitude of the PCCs.

## 3. Discussion

### 3.1. COMPARE Analysis: Strong Correlations to DPIQ as the Seed

**Figure 4:**
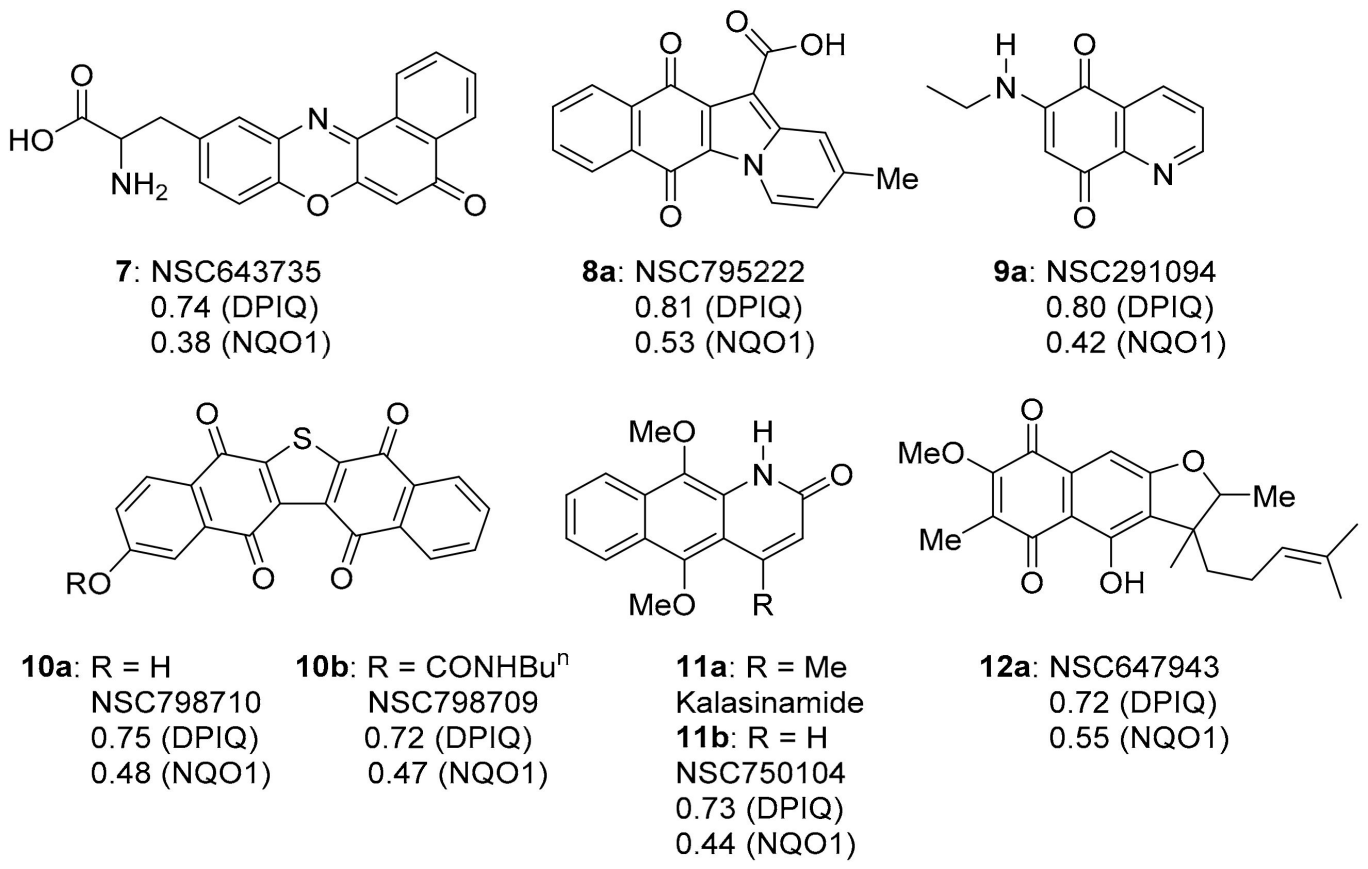
The strongest COMPARE correlations to DPIQ, excluding **3a** and **4**.

This section reviews compounds **3a**, **4**, **7**–**12** with strong to very strong correlations (PCC = 0.72–0.87) in anti-cancer activity to DPIQ (Figure 1 and Figure 4). COMPARE gave planar fused heterocyclic compounds containing the oxidising quinone or iminoquinone motif, apart from azaanthracenone **11b**, which is the *para*-dimethoxybenzene (bio)synthetic precursor (see Section 3.1.5). Weak to moderate correlations (PCC = 0.38–0.64) to NQO1 expression across the NCI-60 cell line panel were demonstrated, although benzo[*e*]perimidine-4-carboxylic acid **3a** is found to have a stronger correlation to NQO1 (PCC = 0.64, Figure 1). The following sub-sections review each heterocyclic scaffold, apart from quinoline-5,8-dione **9a**, since quinoline-5,8-diones also have strong correlations to the benzo[1,2,4]triazinone **5a** (reviewed in Section 3.3).

#### 3.1.1. Benzo[*e*]perimidines

Commercially available benzo[*e*]perimidine-4-carboxylic acid **3a** displays the greatest similarity in anti-cancer activity to DPIQ, with a PCC of 0.87, and a moderate to strong PCC of 0.64 to NQO1 expression across the NCI-60 cell line panel (Figure 1). Recently, iminoquinone **3a** was shown to induce apoptosis in NCI cell lines with high nuclear factor erythroid 2-related factor 2 (NRF2) activation [24]. NRF2 is a transcription factor over-expressed in pancreatic adenocarcinomas, which regulates expression of many redox enzymes, including NQO1 [25]. Compound **3a** induced apoptosis *in ex vivo* cultures of pancreatic cancer xenografts with a high NQO1/NRF2 activation, and inhibited the biosynthesis of amino acids, including asparagine and methionine [24].

**Scheme 5:**
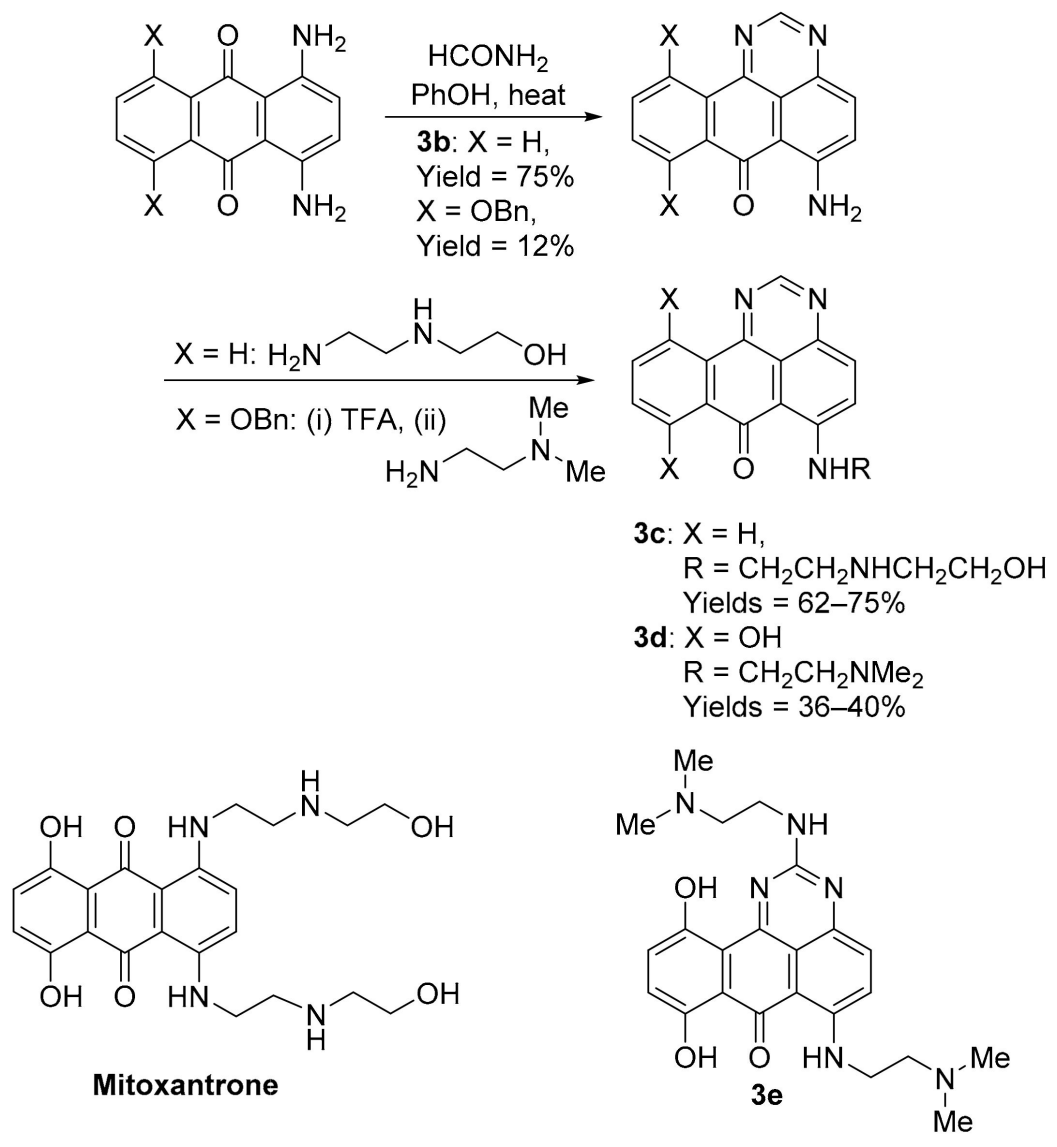
Synthesis and anti-cancer evaluation of benzo[*e*]perimidines **3b**–**e** [26,27,28].

The benzo[*e*]perimidine scaffold is obtained using the condensation of acetamide with l,4-diamino-9,10-anthracenedione in molten phenol, with transamination providing 6-[(aminoalkyl)amino]-substituted examples **3c** and **3d** (Scheme 5) [26]. Substitutions mimic the well-known clinical anti-cancer agent mitoxantrone, originally used to treat leukaemia. Borowski and co-workers demonstrated the requirement of the 6-[(aminoalkyl)amino]-substituent in antileukemic activity, e.g., **3c**, with the synthetic precursor **3b** inactive [26], and 8,11-dihydroxybenzo[*e*]perimidin-7-one **3d** displaying significant toxicity against NCI leukemic and solid tumour cell lines [27]. Compound **3d** displayed *in vivo* toxicity and overcomes multi-drug resistance tumour cells and, like mitoxantrone, induces cell cycle accumulation in the G2/M phase. The same group evaluated guanidine condensation adducts, with **3e** displaying the greatest *in vitro* toxicity [28].

#### 3.1.2. Phenoxazinones

**Figure 5:**
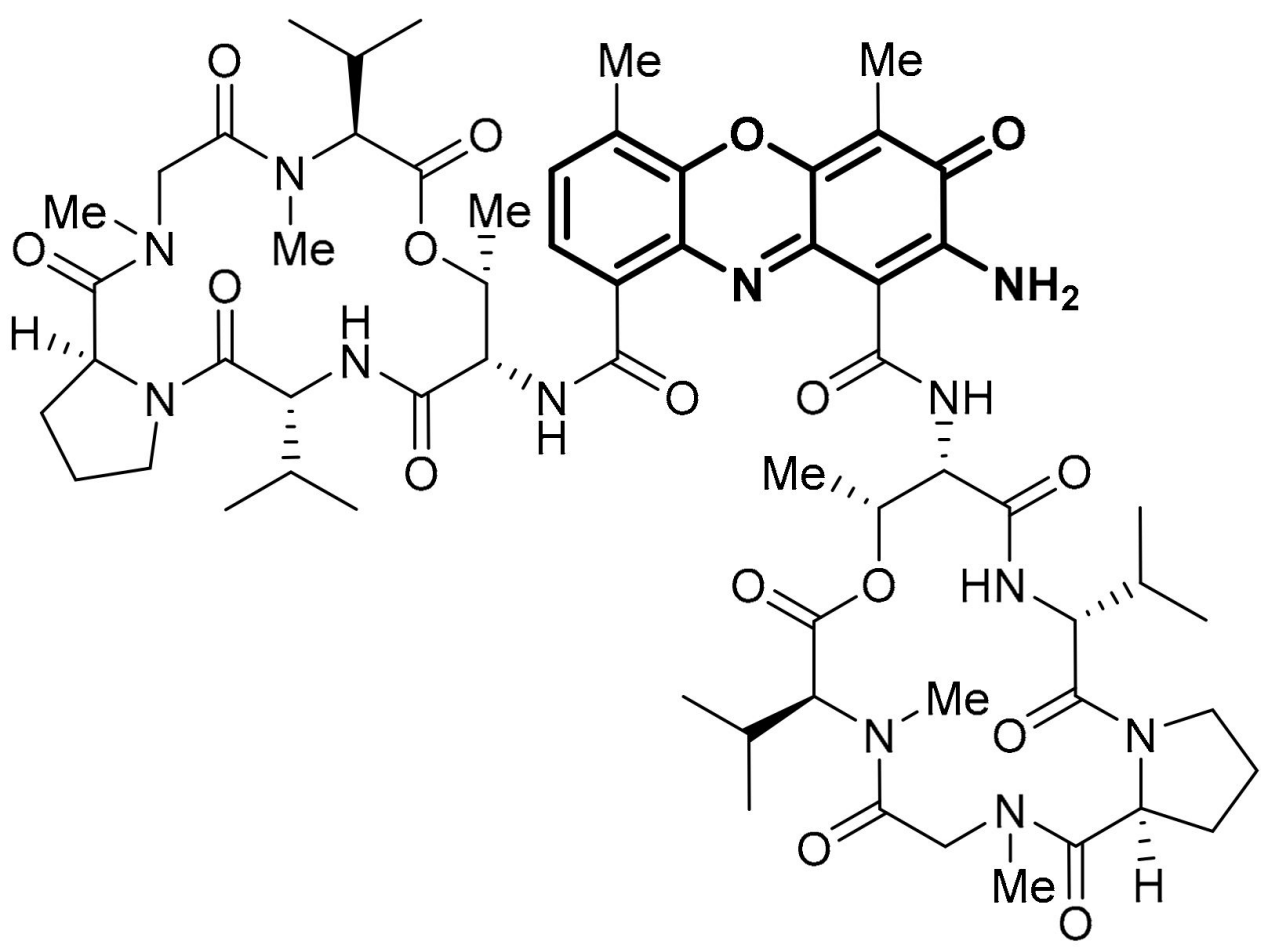
Actinomycin D, showing 2-aminophenoxazin-3-one **4** core in bold.

DPIQ displays a strong similarity in anti-cancer activity to the extensively studied and abundant natural chromophore, 2-aminophenoxazin-3-one **4** (PCC = 0.77, Figure 1), also known as the antibiotic questiomycin A [29,30,31,32,33]. Iminoquinone **4** is isolated from the 6-electron oxidative coupling of two molecules of 2-aminophenol [33,34], mediated by the enzyme phenoxazinone synthase [35]. The antibiotic has a weak to moderate correlation to NQO1 expression (PCC = 0.47) [7]. Anti-cancer activity of **4** includes cytotoxicity against a variety of human cancer cells lines, with induction of apoptosis [31,36,37] and suppression of NO and prostaglandin E2 production leading to anti-inflammatory effects [38]. Cancer spread was monitored *in vivo* in mice, where the number of pulmonary metastasis of B6 melanoma cells remained the same [37]. The first reported antibiotic with anti-cancer activity is actinomycin D [39], containing 2-aminophenoxazin-3-one **4** at its core (Figure 5) [34]. Actinomycin D (also known as dactinomycin) has the trade name Cosmegen and is clinically used to treat a variety of solid tumours.

The phenoxazinone scaffold is contained in heterocyclic iminoquinone **7**, which also displays a strong PCC of 0.74 to DPIQ activity (Figure 4). Benzo[*a*]phenoxazin-5-one **7** was assembled by condensation of 1-nitroso-2-naphthol with *L*-tyrosine [40]. The iminoquinone was identified from the NCI diversity set of 2000 compounds as an inhibitor of HIF-1α induced by insulin-like growth factor-1 [41]. HIF-1α is one of the sub-units of HIF-1, the transcription factor over-expressed in hypoxic tumour cells, and responsible for regulating anaerobic metabolism that leads to tumour progression and angiogenesis [42]. SET, which is reversible by oxygen and mediated by NADPH-cytochrome (P450) reductase, is associated with tumour hypoxia [16,43,44], and is prevented by the NQO1-mediated two-electron reduction (Scheme 2) [7,18,21,45]. This specificity towards hypoxia provides a rationale for the weak correlation of iminoquinone **7** (0.38) to NQO1 expression across the NCI 60 cell lines.

Increased cell signalling mediated by reversible protein phosphorylation supports cancer cell proliferation. The human cytoplasmic protein tyrosine phosphatases (HCPTPs) are overexpressed in hypophosphorylated breast cancer cells [46]. Benzo[*a*]phenoxazin-5-one **7** was identified as the third-most potent inhibitor of HCPTP isoform B (IC_50_ = 31 µM), after *in silico* docking of the compounds of the NCI Diversity Set I and enzyme inhibition assays on five selected compounds [47].

#### 3.1.3. Benz[*f*]pyrido[1,2-*a*]indole-6,11-quinone

**Figure 6:**
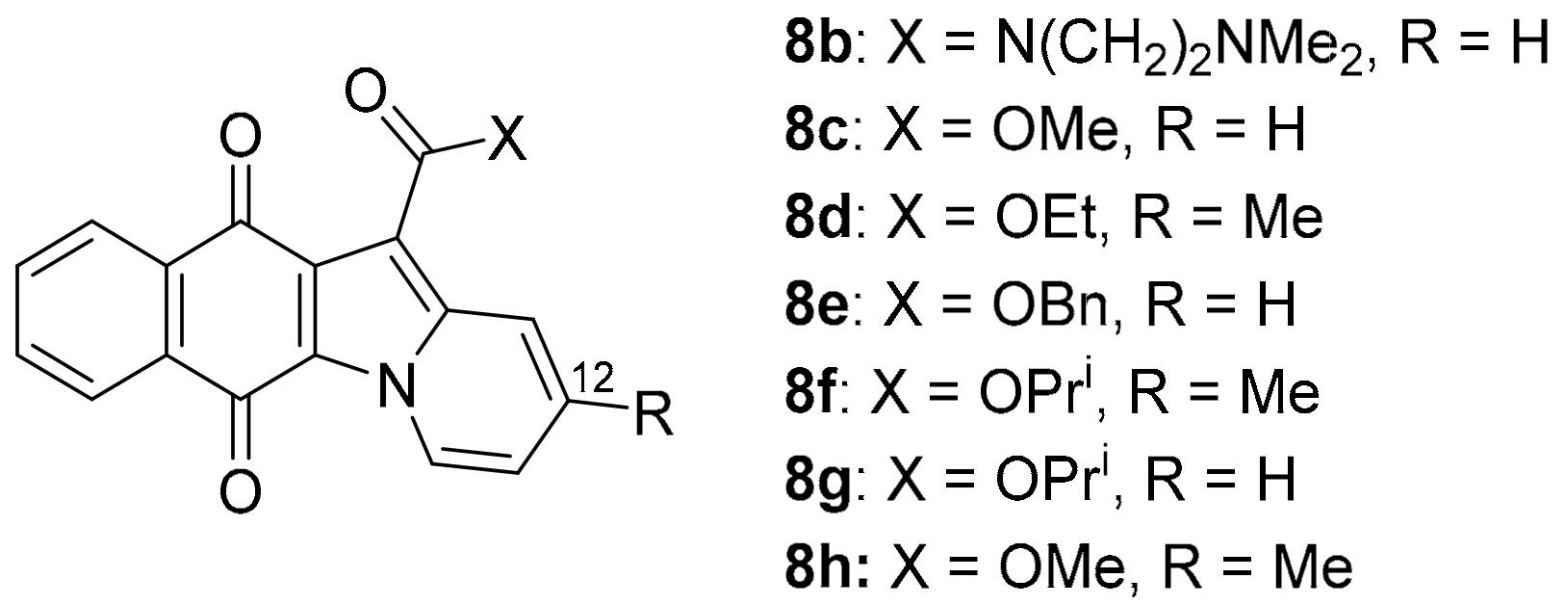
Biologically active benz[*f*]pyrido[1,2-*a*]indole-6,11-quinones [48,49,51,52].

Updating the 2012 COMPARE analysis on DPIQ [7] revealed that the carboxylic acid **8a** of benz[*f*]pyrido[1,2-*a*]indole-6,11-quinone (Figure 4) has the second-highest PCC of 0.81. Testing against the NCI-DTP cell panel revealed the *N*,*N*-dimethylethyl carboxamide **8b** to be the most potent amongst carboxamide derivatives evaluated (Figure 6), with cytotoxicity against the adriamycin-resistant breast tumour cell line (NCI/ADR-RES) at a concentration lower than clinical anticancer agents, daunorubicin and mitoxantrone [48]. More recently, methylester **8c** was found to inhibit indoleamine 2,3-dioxygenase 1 (IDO1) [49]. IDO1 depletes tryptophan, with deficiencies in this essential amino acid leading to suppression of immune response to tumours [50]. 12-Unsubstituted and carboxylate ester derivatives of **8a**, including ethylester **8d**, exhibit anti-fungal activities [51]. Carboxylates **8e**–**h** display micromolar anti-bactericidal activity against the Erdman strain of *Mycobacterium tuberculosis*, with the molecular target being a membrane-bound, iron−thiol reductase (IspQ) [52].

**Scheme 6:**
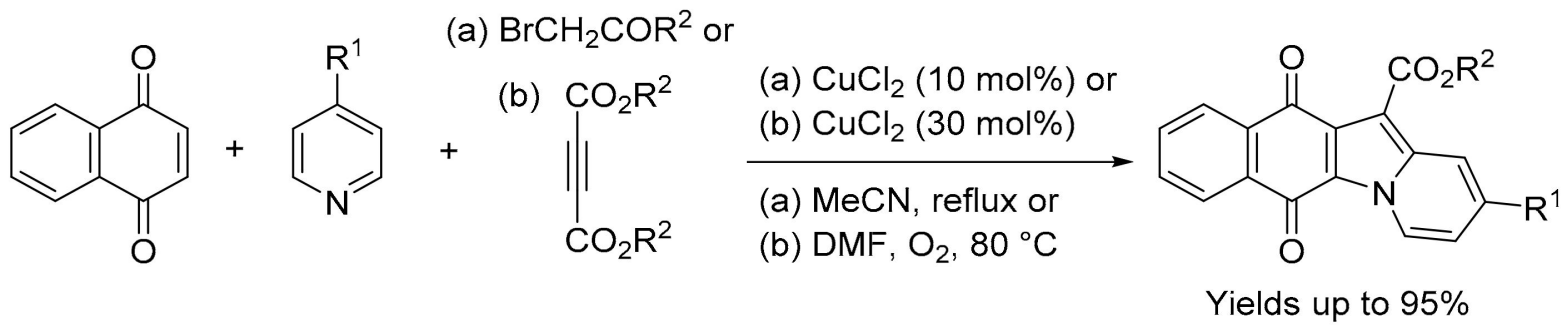
Synthesis of benz[*f*]pyrido[1,2-*a*]indole-6,11-quinones [53,54].

For the biological evaluation purposes described above, the benz[*f*]pyrido[1,2-*a*]indole-6,11-quinone scaffold was obtained using adaptations of the traditional three-component, one-pot condensation of 2,3-dichloro-1,4-naphthoquinone, an active methylene dicarbonyl and pyridine [48,49,51,52]. Yields are improved when using three-component reactions with 1,4-naphthoquinone and pyridine catalysed by CuCl_2_ with acyl bromides [53], or cleavage of butynedioates [54] (Scheme 6).

#### 3.1.4. Analogues of Seriniquinone

**Figure 7:**
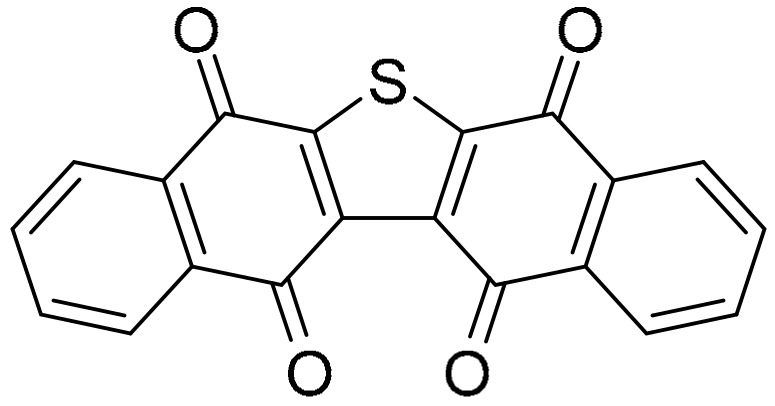
Seriniquinone.

Dibenzo[*b*,*i*]thianthrene-5,7,12,14-tetrone was first synthesized in 1991 [55], and identified in 2014 as seriniquinone (Figure 7), derived from the marine bacterium *Serinicoccus marinus* [56].

**Scheme 7:**
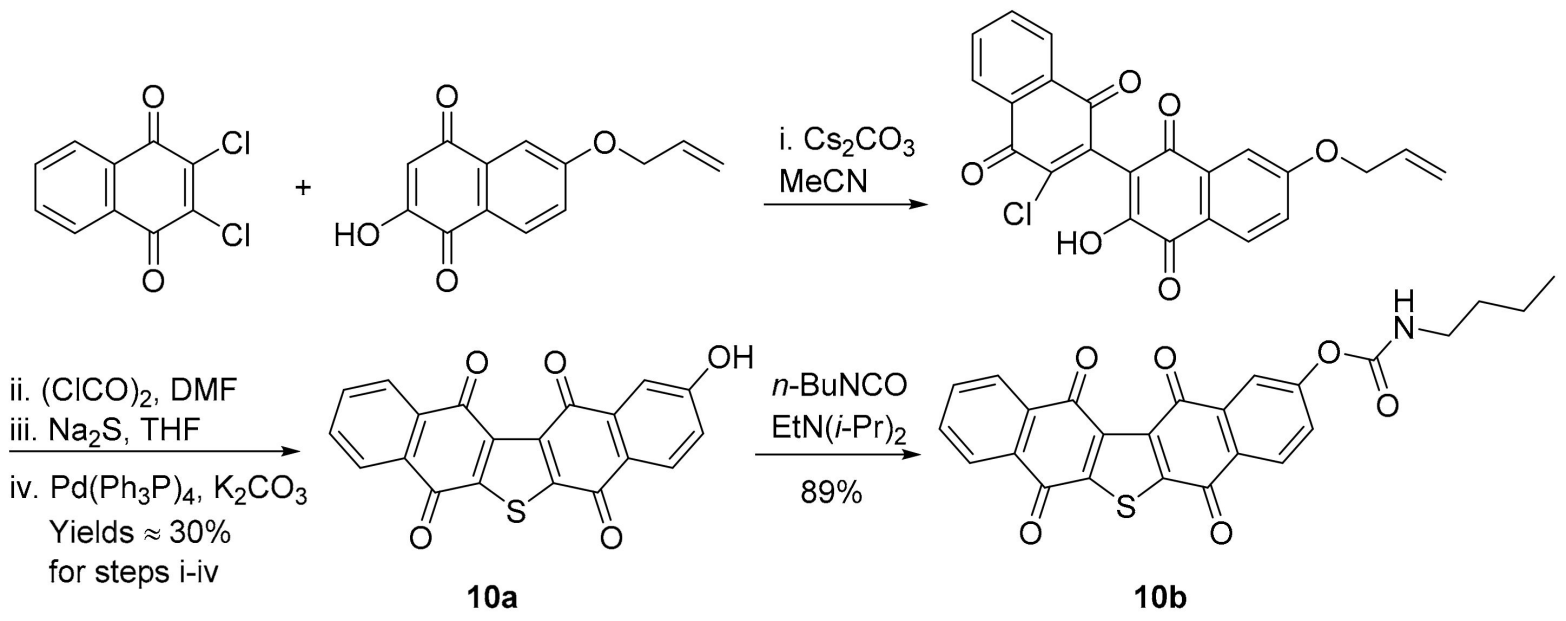
Synthesis of seriniquinone analogues [57].

Synthetic analogues of seriniquinone are required due to its poor water solubility, with **10a** and **10b** showing strong PCCs of 0.75 and 0.72 to DPIQ anti-cancer activity, respectively, but weak to moderate PCCs of 0.47 and 0.48 to NQO1 expression, respectively (Figure 4). Phenol **10a** and carbamate **10b** were prepared on a multi-gram scale via naphthalene-1,4-dione coupling, thiophene ring-formation, and Pd-induced deprotection (Scheme 7) [57]. Carbamate **10b** was shown to hydrolyse at room temperature to **10a** under phosphate-buffered saline (PBS) at pH 7.2. Analogues **10a** and **10b**, like DPIQ [7], possess specificity towards the NCI melanoma cell lines. Cytotoxicity of seriniquinones was demonstrated through binding to dermcidin [56,57], a small protein involved in cancer cell proliferation [58] and induction of cell death via autophagocytosis [56]. More recently, seriniquinones were shown to induce elevated levels of intracellular ROS, which trigger apoptotic events [59], supporting the relatively weak correlations to NQO1. ROS is related to the SET radical producing processes (Scheme 2), which are not mediated by NQO1.

#### 3.1.5. Azaanthracenone

**Scheme 8:**
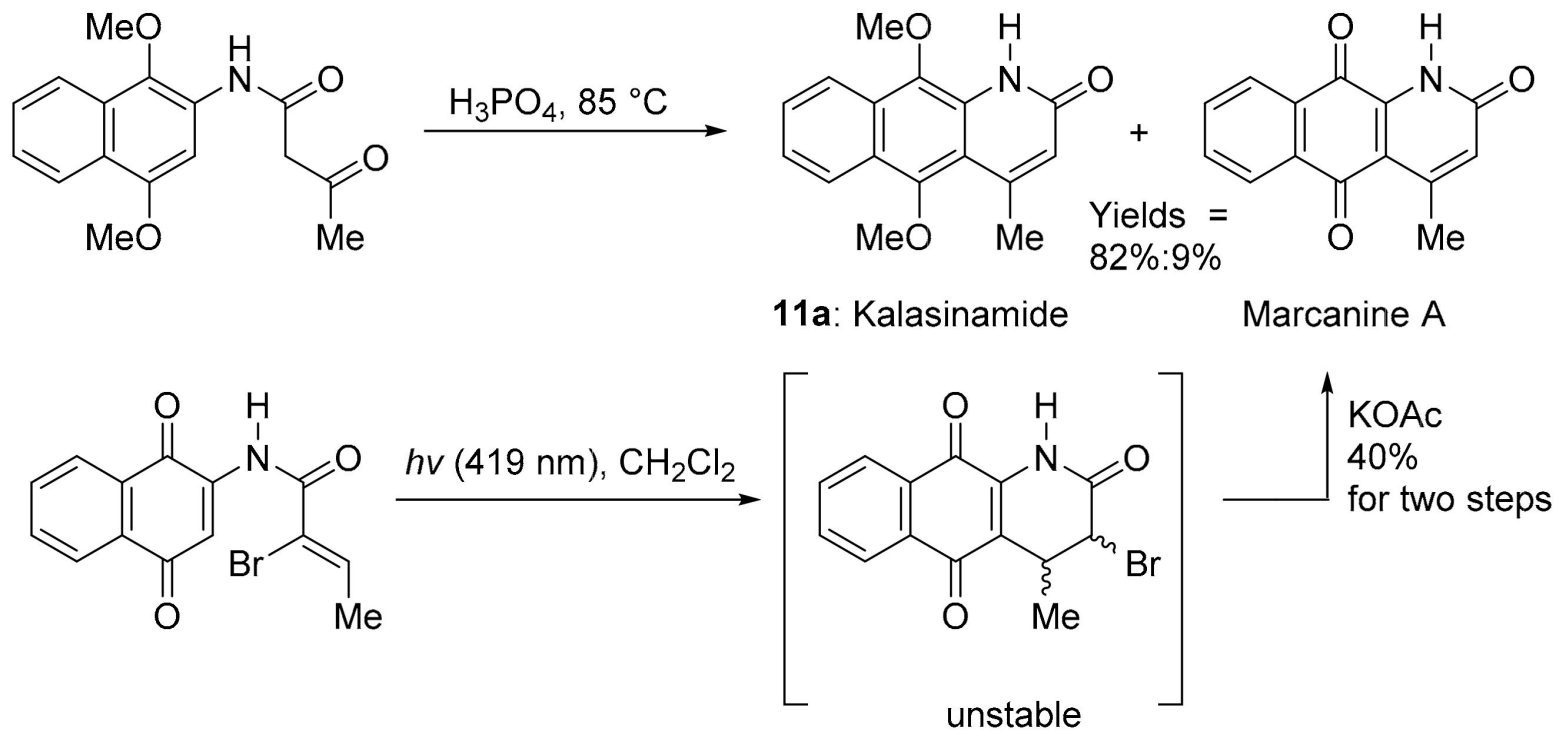
Syntheses of kalasinamide and marcanine A with yields [61,64].

Kalasinamide (**11a**) is an azaanthracenone first isolated from the tree *Polyalthia suberosa* (Figure 4) [60]. The unreported analogue **11b** without the 4-methyl substituent was found by the COMPARE analysis to have a strong PCC to DPIQ of 0.73 and a weak to moderate PCC to NQO1 of 0.44. Kalasinamide is a photosensitiser thought to prevent the invasion of pathogens by generating singlet oxygen, and/or through conversion to the quinone marcanine A [61]. Kalasinamide is prepared through acid-mediated substitutions onto the activated *p*-dimethoxybenzene [61,62], including using Knorr cyclisation, which gives a mixture of **11a** and marcanine A, in the presence of oxygen and light (Scheme 8). Marcanine A is a plant natural product [61,63], also prepared through photochemical cyclisation onto the naphthoquinone acrylamide substituent [64]. The potent anti-bacterial marcanine A [63] has cytotoxicity comparable to adriamycin, using five different solid tumour cell lines [65].

#### 3.1.6. Furano[2,3-*b*]naphthoquinone (FNQ)

**Figure 8:**
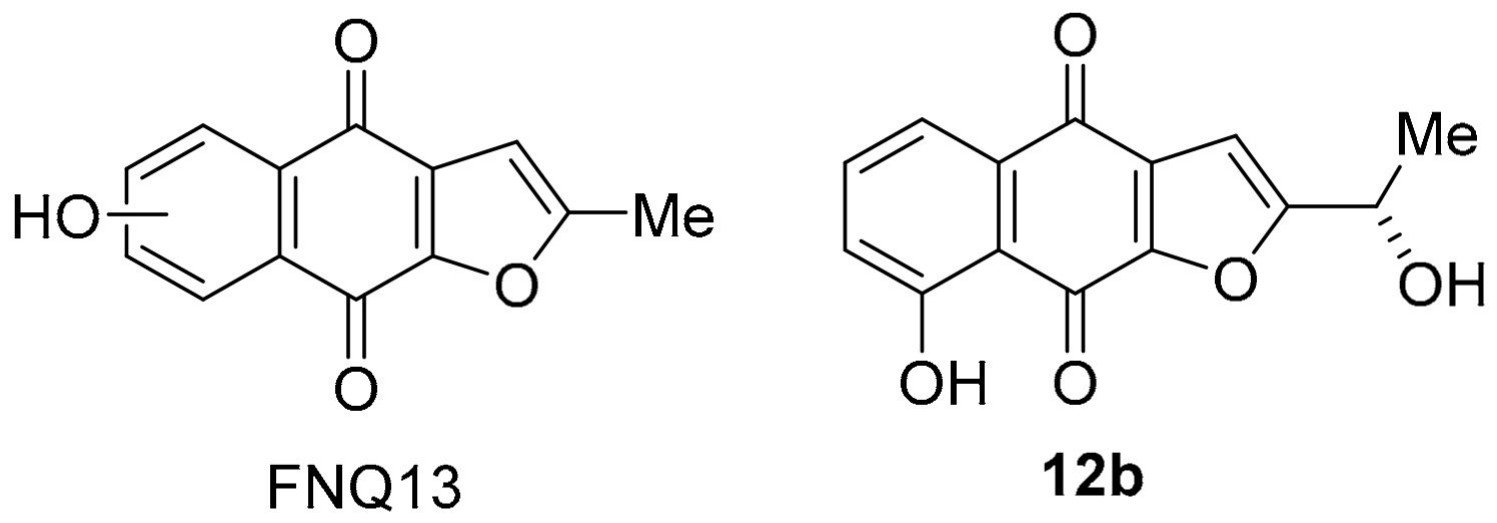
Anti-cancer FNQs.

The polyketide-isoprenoid **12a** was isolated from *Streptomyces cinnamonensis* (Figure 4) [66]. The biosynthesis of furano[2,3-*b*]naphthoquinone (FNQ) **12a** has attracted considerable attention [67]; however, there is no literature on antimicrobial and anti-cancer studies. The COMPARE analysis shows that **12a** exhibits a strong PCC to DPIQ of 0.72, and a moderate PCC of 0.55 to NQO1 expression across the NCI-60, which is comparable with DPIQ (0.51) (Figure 1). Related synthetic structures (FNQ13, Figure 8) induce mitochondrial swelling and apoptosis, due to induction of ROS including H_2_O_2_ [68]. Furano[2,3-*b*]naphtho-4,9-dione **12b**, isolated from *Tabebuia avellanedae*, shows a preference for inhibition of cancer cell growth over normal cells, with STAT3 inhibitory mechanisms proposed [69], while other FNQ natural products inhibit IDO1 (see Section 3.1.3) [70]. Inhibition of STAT3 may be important because the Janus kinase/signal transducers and activators of transcription (JAK/STAT) pathway plays a key role in membrane-to-nucleus signalling, which is critical in mediation of cancer and inflammation [71].

**Scheme 9:**
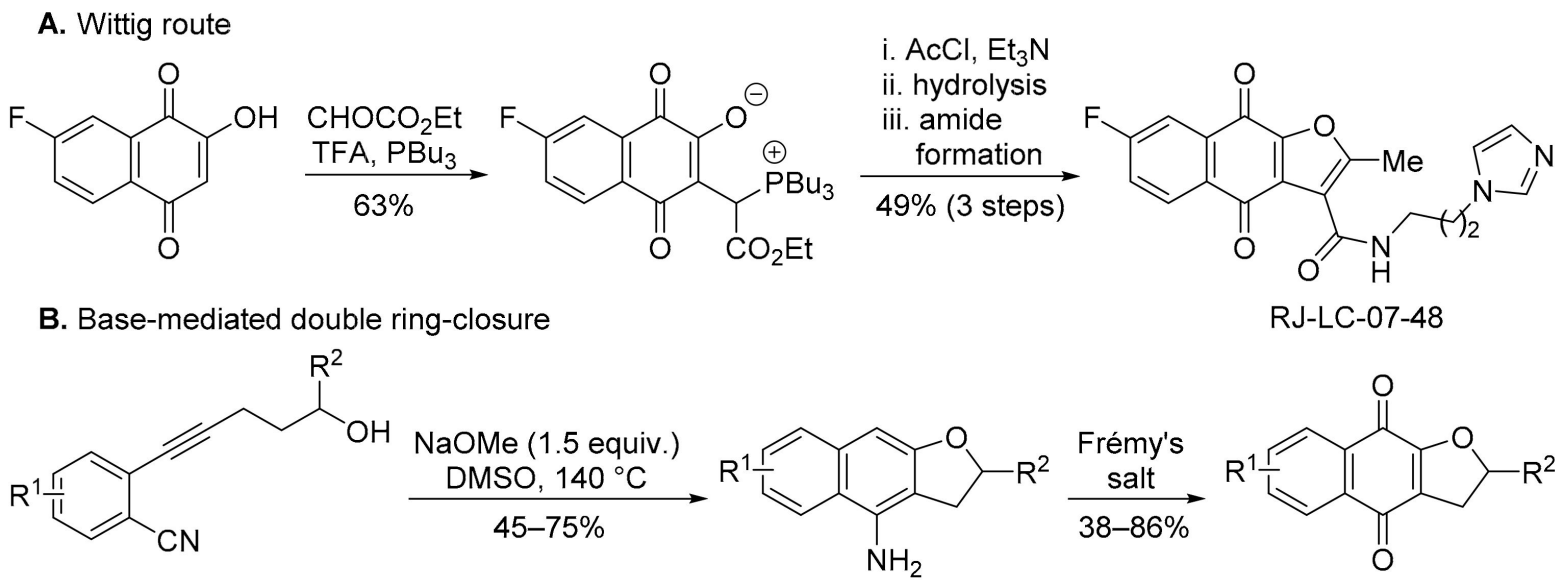
Syntheses of furano[2,3-*b*]naphtho-4,9-diones [73,74].

Most syntheses of FNQ involve forming the furan ring from 2-hydroxy-1,4-naphthoquinone [70,72,73], including by an acetyl chloride/Et_3_N-mediated Wittig reaction (Scheme 9A) [72,73]. The product (RJ-LC-07-48) is potent against drug-resistant non-small-cell lung cancer (NSCLC) cells by interaction with the minichromosomal maintenance protein MCM2, disrupting the formation of the MCM complex that is required for the initiation of DNA replication [73]. Similar specificity towards non-small cell lung cancer cell lines was shown by DPIQ [7]. Wu and co-workers transformed 2-(5-hydroxy-1-pentynyl)benzonitriles to FNQ via a NaOMe-mediated ring-closure, followed by Frémy’s salt oxidation (Scheme 9B) [74].

### 3.2. COMPARE Analysis: Strong Correlations to Benzo[1,2,4]triazin-7-one ***5a*** as the Seed

**Figure 9:**
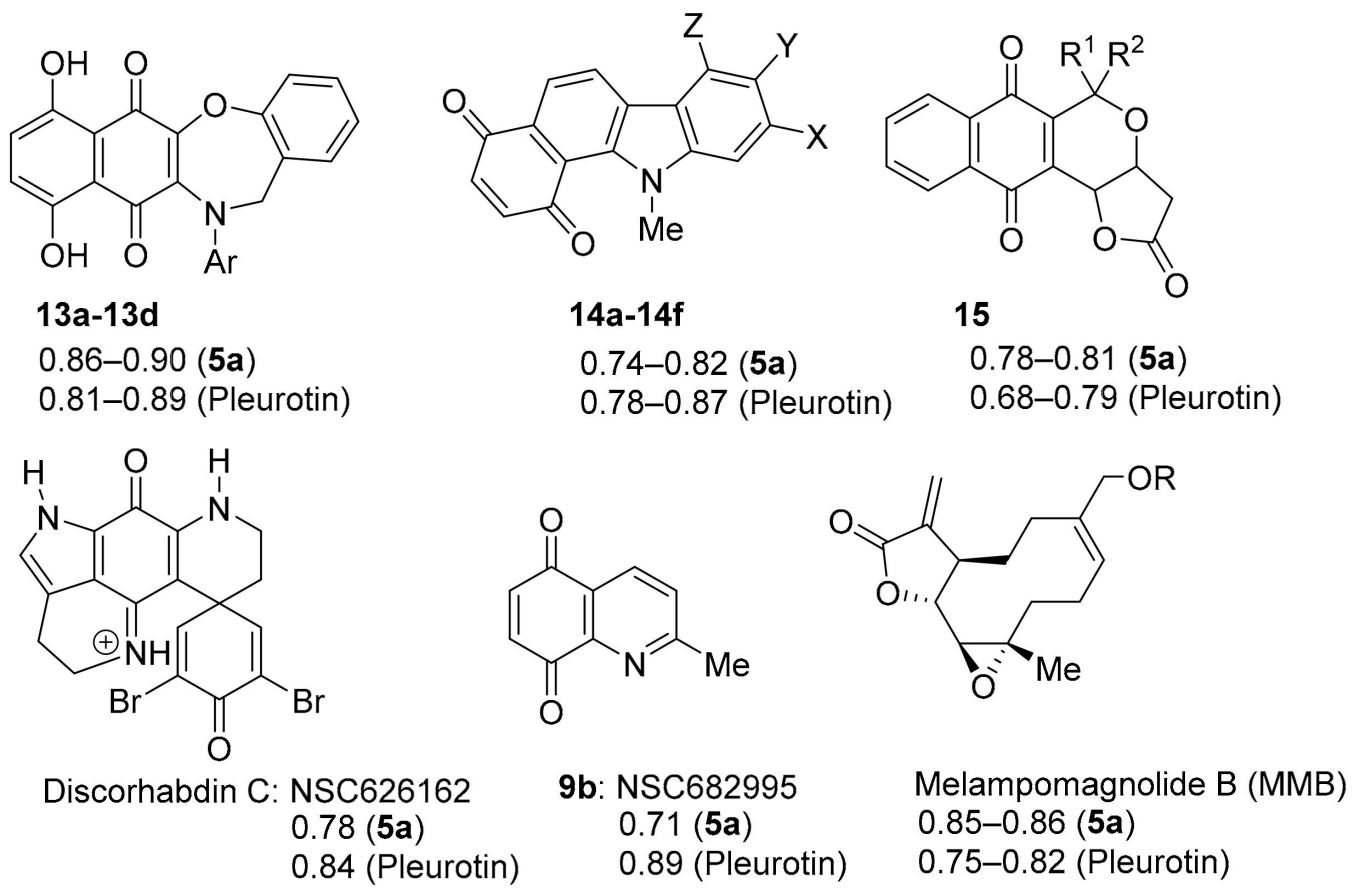
Very strong COMPARE correlations to benzo[1,2,4]triazinone **5a** and pleurotin.

The NCI COMPARE analysis using benzo[1,2,4]triazin-7-ones **5a** and pleurotin, as the seed revealed very strong correlations to synthetic heterocyclic quinone scaffolds **13a**–**d** and **14a**–**f**, as well as to the natural products pyranonaphthoquinones **15** and discorhabdin C (PCC = 0.68–0.90, Figure 9). However, unlike DPIQ (above), very strong PCCs of ~0.85 (to **5a**) are also found to non-quinone structures, notably synthetic derivatives of the anti-cancer sesquiterpene lactone melampomagnolide B (MMB), isolated from *Magnolia grandiflora* [75,76]. MMB derivatives show specificity towards leukemic cell lines [76,77], with dimeric examples showing nanomolar activity against solid tumour cell lines in the NCI-60 panel [77]. Similarly, benzotriazin-7-ones **5a**–**d** also showed potent anti-proliferative effects against most NCI leukemic cell lines [10,11]. MMB is an extensively investigated scaffold that is thought to target the NFkB pathway through inhibition of the IkBa/p65/p50 kinase complex (IKK) [78]. The following section reviews the heterocyclic quinones and iminoquinones that have strong to very strong correlations to the anti-cancer activity of TrxR inhibitors **5a** and pleurotin. Quinoline-5,8-diones (e.g., **9b**) are reviewed separately in Section 3.3.

#### 3.2.1. Naphtho[2,3-*b*][1,4]oxazepine-6,11-dione

**Figure 10:**
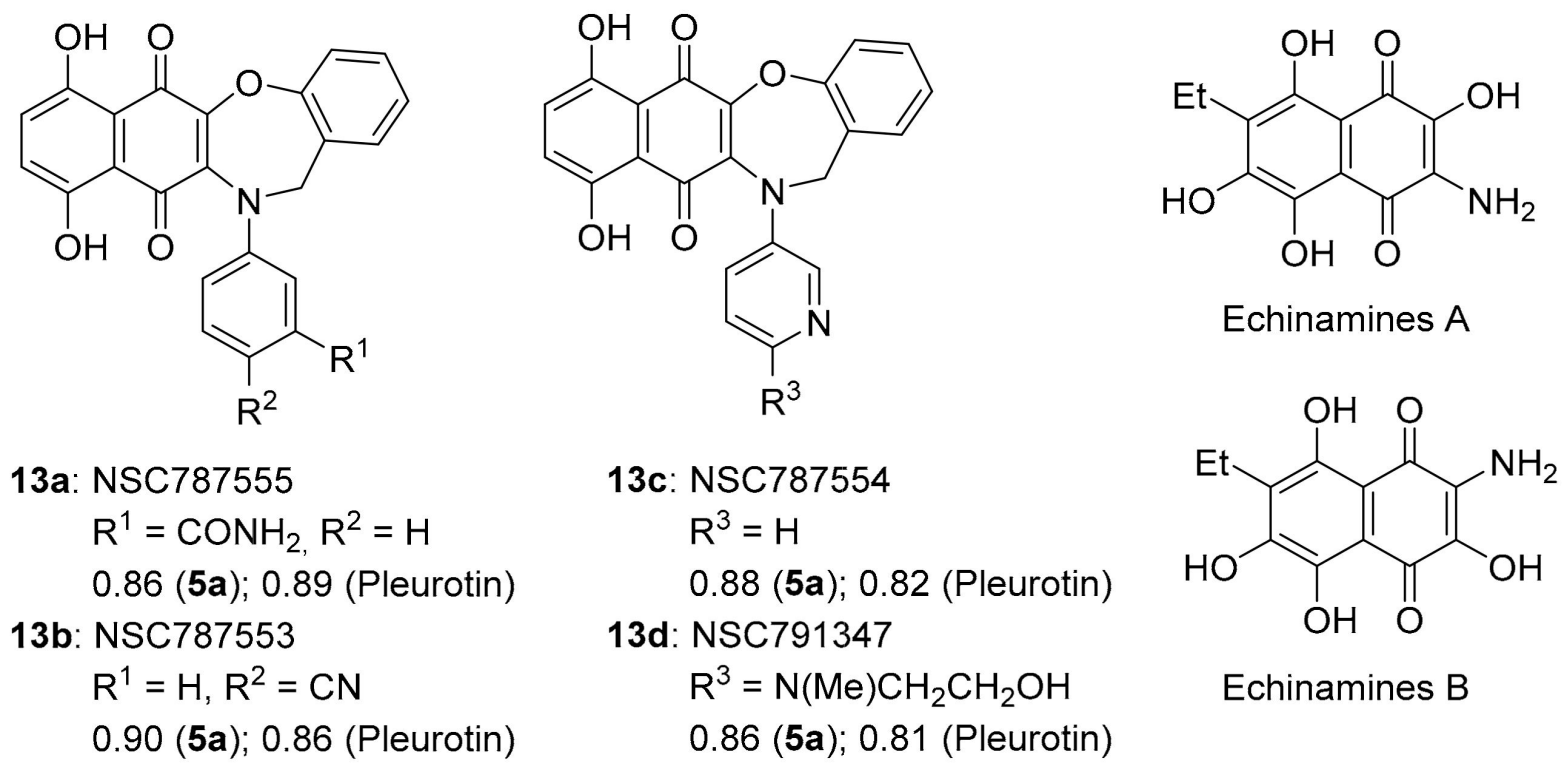
1,4-Benzoxazepine derivatives of 1,4-naphthoquinones with PCCs and the related natural products Echinamine A and B.

COMPARE gave an almost perfect PCC of ~0.90 for the anti-cancer activity of benzo[1,2,4]triazinone **5a** to 1,4-benzoxazepine derivatives of 5,8-dihydroxy-1,4-naphthoquinones **13a**–**d** (related to Echinamines A and B) (Figure 10). Echinamines A and B are antioxidants isolated from the sea urchin *Scaphechinus mirabilis* [79], which suppress herpes simplex virus type 1 infection in the Vero monkey kidney cell line [80]. COMPARE analysis-derived 1,4-benzoxazepines **13a**–**d** are unreported, giving (Figure 3) very strong correlations to pleurotin anticancer activity (PCC = 0.8–0.9), analogous to **5a**–**d**. The almost perfect correlations of **13a**–**d** to pleurotin are perhaps unsurprising, given the common 1,4-naphthoquinone motif.

**Scheme 10:**
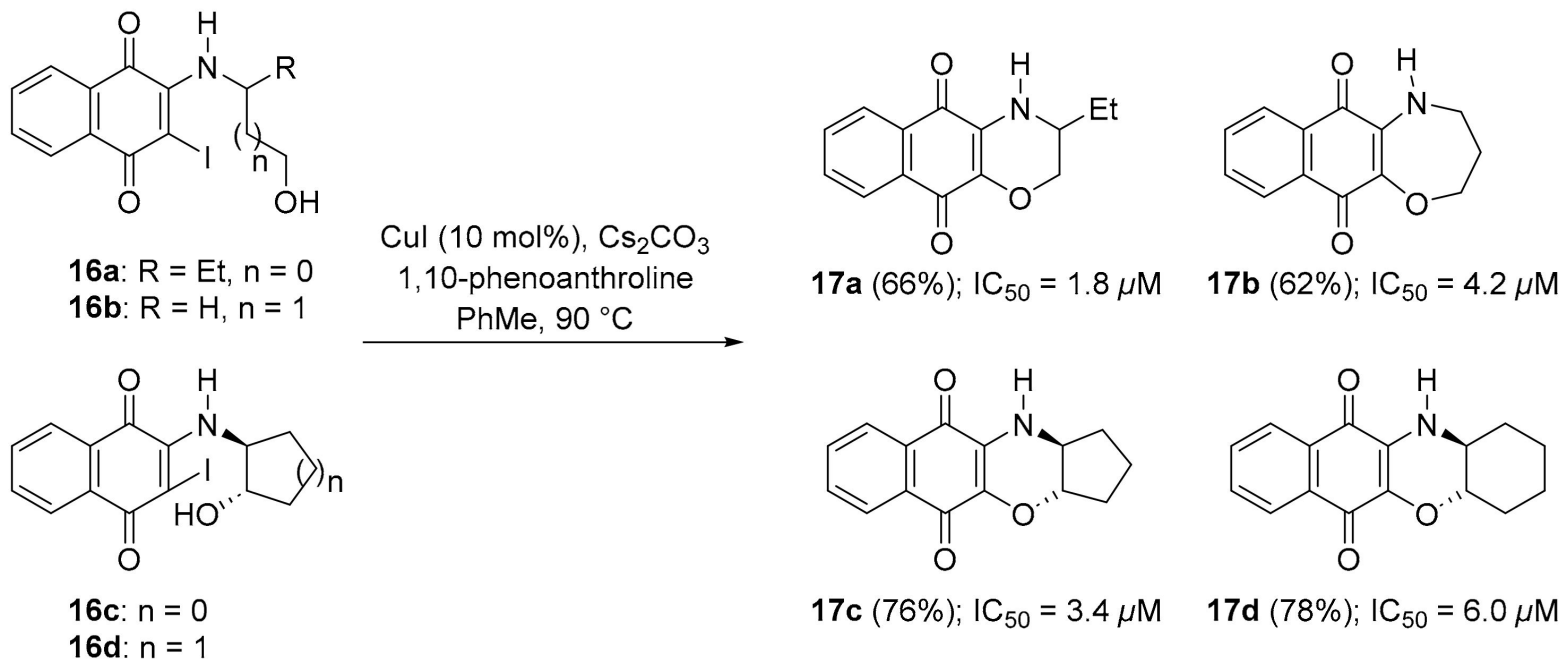
Synthesis (with yields) of naphtho[2,3-*b*][1,4]-oxazine-1,4-diones **17a**–**d** and IC_50_ against A549 cells [81].

You *et al*. used an intramolecular copper-catalysed hydroxyl displacement of iodides (**16a**–**d**) to synthesize homomorpholine **17b**-fused and morpholine-fused, e.g., **17a**, **17c** and **17d**, naphthoquinone adducts with compounds giving moderate inhibition of lung A549 cell growth (Scheme 10) [81].

#### 3.2.2. Benzo[*a*]carbazole-1,4-dione

**Figure 11:**
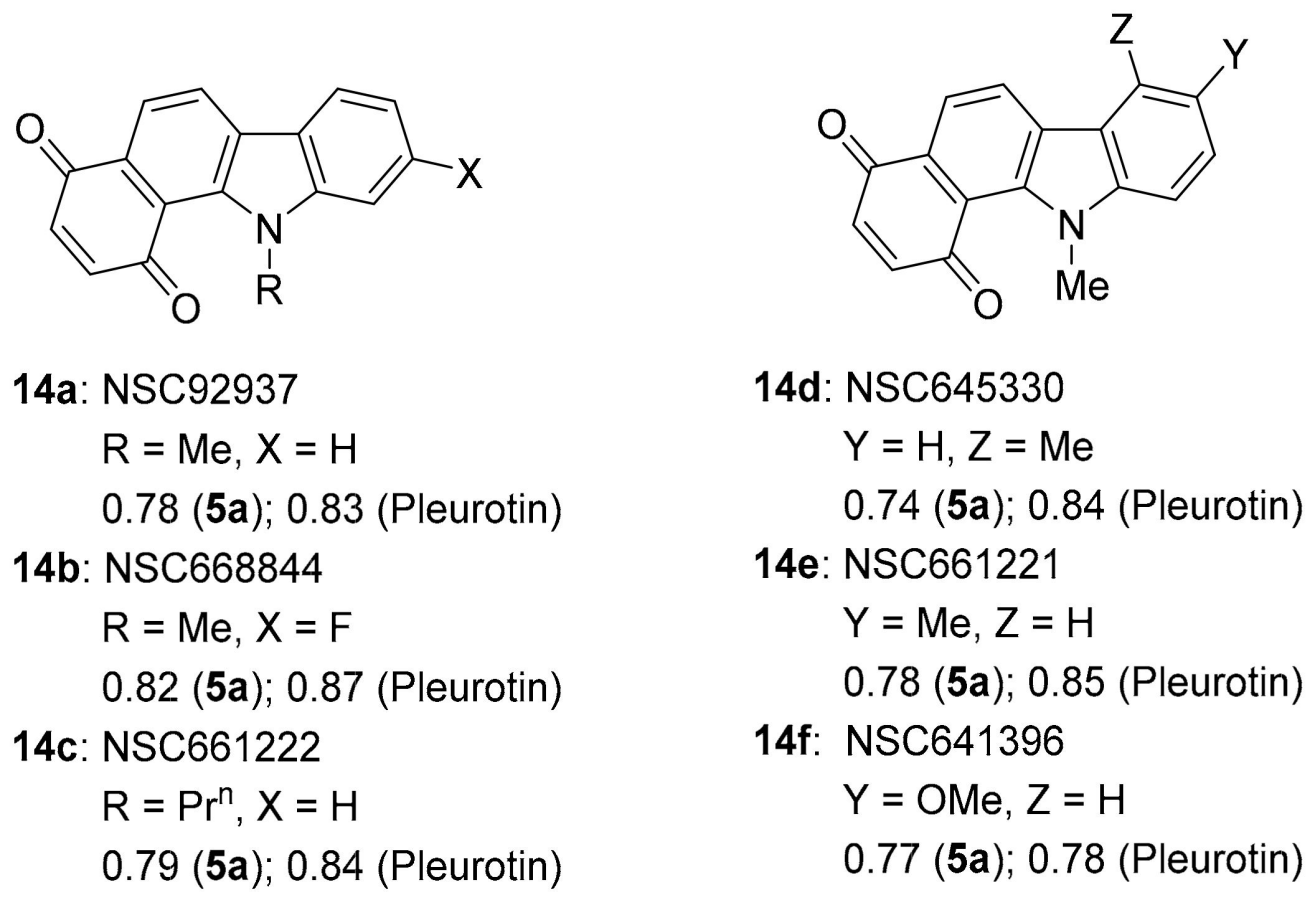
Benzo[*a*]carbazole-1,4-dione antibiotics and anti-cancer agents [82,83,84,85].

Benzo[*a*]carbazole-1,4-dione **14c** and others appear in the patent literature as inhibitors for the thioredoxin TrxR-Trx system (Figure 11) [82]. This supports the very strong anti-cancer activity correlations with TrxR inhibitors, benzo[*e*][1,2,4]triazin-7-one **5a** (~0.74–0.82) and pleurotin (0.78–0.87). Benzo[a]carbazole-1,4-dione **14a** was one of 6 hits out of 2000 in the NCI library to inhibit *Plasmodium* kinesin-5 from both human and malaria *vivax* [83]. Compounds **14a** and **14d**–**f** were amongst 35 hits out of 1597 compounds in the NCI Diversity SET III screen with >50% inhibitory activity, at concentrations of 20 µM of the immunosuppressive enzyme IDO1 [84]. Benzo[a]carbazole-1,4-dione **14a** is effective against vancomycin-resistant *Staphylococcus aureus* by targeting the cysteine thiol of bacterial MarR transcription factors [85].

**Scheme 11:**
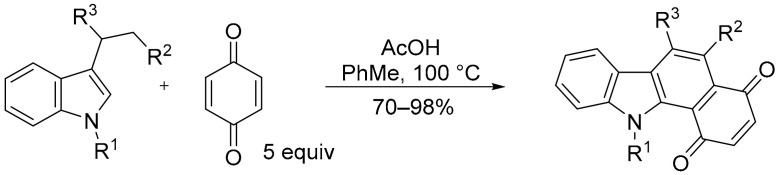
Synthesis of benzo[*a*]carbazole-1,4-dione [87].

The traditional synthesis of the benzo[*a*]carbazole-1,4-dione scaffold (Figure 11) involves a Diels–Alder cycloaddition of 3-vinylindole with benzoquinone and oxidative dehydrogenation [86,87]. The 3-vinylindole can be generated *in situ* from 3-ethylindole using benzoquinone as the oxidising agent, and the benzoquinone then acts as the dienophile to give the benzo[*a*]carbazole (Scheme 11) [87].

#### 3.2.3. Pyranonaphthoquinones

**Figure 12:**
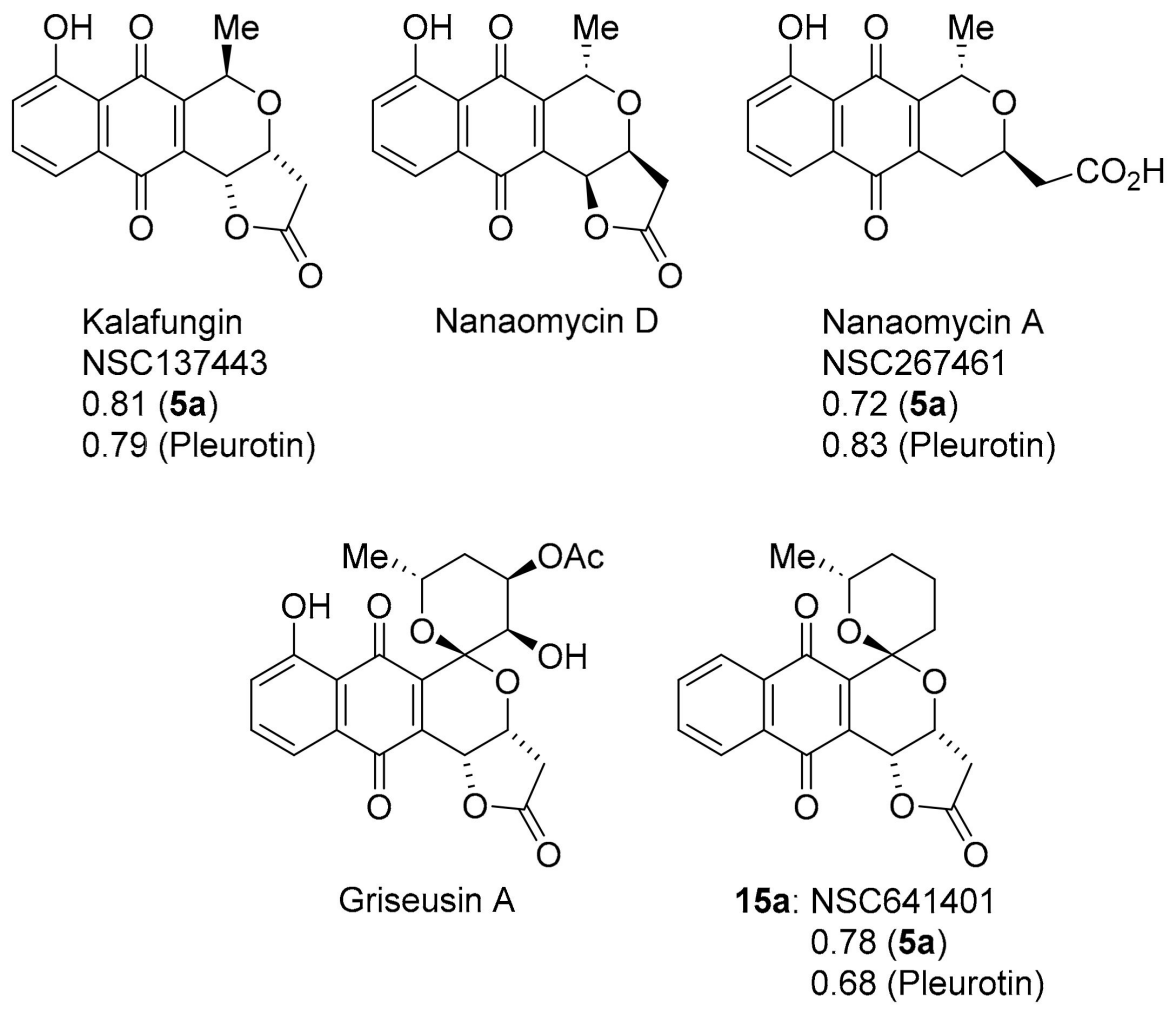
The pyranonaphthoquinones with COMPARE analysis PCCs.

Pyranonaphthoquinones are natural products possessing the naphtho[2,3-*c*]pyran-5,10-dione ring system (Figure 12) [88,89]. Benzotriazinone **5a** and pleurotin show strong to very strong PCCs of 0.72–0.83 to the antibiotics kalafungin and nanaomycin A first isolated from the *Streptomyces tanashiensis* strain Kala [90,91]. Nanaomycin D isolated from *Streptomyces rosa* is enantiomeric to kalafungin with nanaomycin A, the cleaved lactone [92]. Kalafungin and nanaomycin analogues show specificity towards breast cancer cell lines over-expressing cytochrome P450 oxidoreductase with cytotoxicity diminished under anoxia, where reactive oxygen production is inhibited [93]. The SET recycling mechanism is also important in antibacterial activity [94]. Conversely, kalafungin and analogues were reported inhibitors of the serine-threonine kinase AKT via a proposed two-electron reduction to the hydroquinone with alkylation of the quinone methide by a cysteine in the activation loop (T-loop) of the kinase domain of AKT [95]. Nanaomycin A was identified in a screening program as a DNA methyltransferase inhibitor, with biochemical assays revealing specificity towards DNMT3B with docking hypothesising a reduction in nanaomycin A by the sulfur atom of a cysteine in the catalytic site [96].

**Scheme 12:**
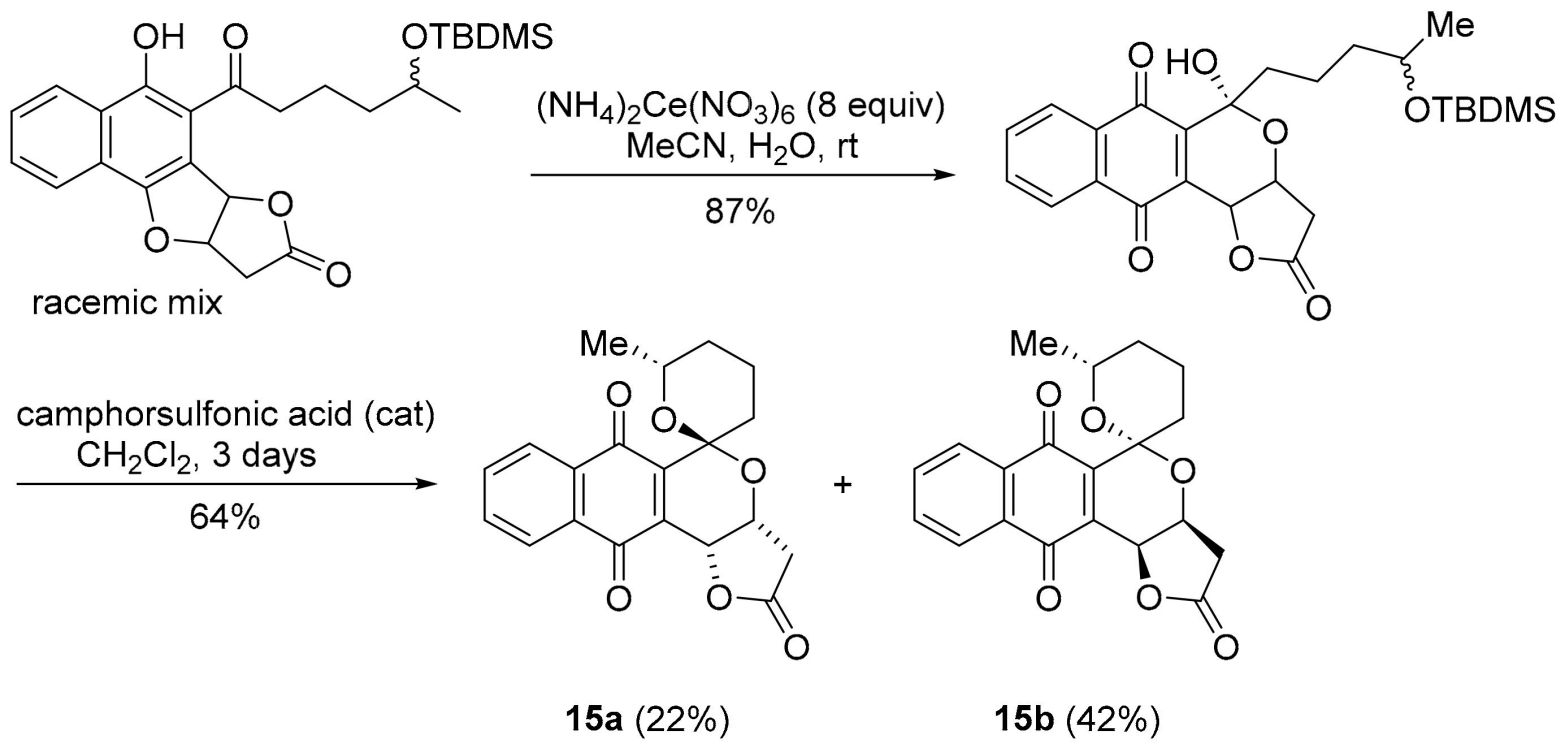
Brimble and co-workers synthesis of the griseusin scaffold [99,100].

Griseusin A, isolated from *Streptomyces griseus*, is a pyranonaphthoquinone antibiotic [97], that bears structural similarity to kalafungin, but possesses a 1,7-dioxaspiro[5,5]-undecane ring system. Synthetic griseusin **15a** exhibits a strong correlation in anti-cancer activity to benzotriazinone **5a** (PCC = 0.78) and pleurotin (0.68) (Figure 12). The multi-step synthesis of griseusin **15a** involves a cerium(IV) ammonium nitrate-mediated oxidative rearrangement and acid-mediated cyclisation to the spiroacetal [98,99,100,101]. Brimble and co-workers have reported the separation of isomers **15a** and **15b** using flash chromatography (Scheme 12) [99,100,101].

#### 3.2.4. Discorhabdin C

**Figure 13:**
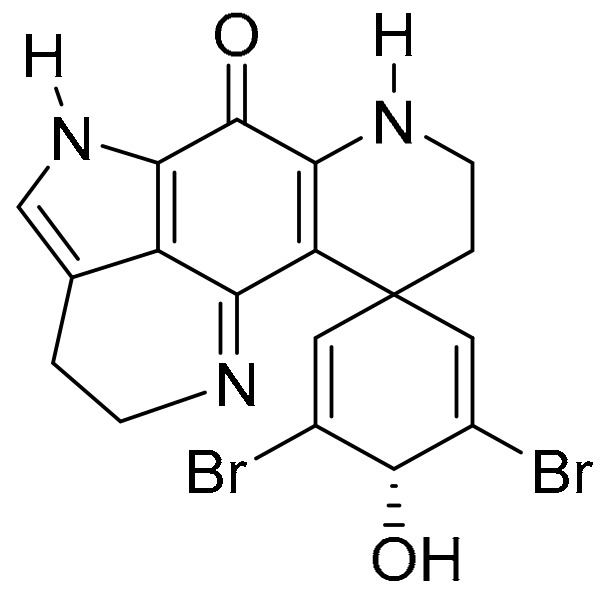
3-Dihydrodiscorhabdin C.

The pyrroloiminoquinone alkaloids, discorhabdins, are isolated from numerous cold water marine sponges, with cytotoxic discorhabdin C from the New Zealand sponge, *Latrunculia* Bocage [102,103]. Discorhabdin C exhibits strong to very strong PCCs to benzotriazinone **5a** (0.78) and pleurotin (0.84) (Figure 9). Munro and co-workers reported selectivity towards the NCI colon and leukaemia subpanels [104], while others reported *in vitro* anti-hepatitis virus C, antimalarial and antimicrobial activities [105]. Figg and co-workers performed high-throughput screens on crude natural product extracts and identified 3-dihydro-discorhabdin C (Figure 13), as a HIF-1α/p300 inhibitor [106], which interferes with the HIF-1α and p300 protein−protein interaction to decrease HIF-1α-dependent transcription [43].

**Scheme 13:**
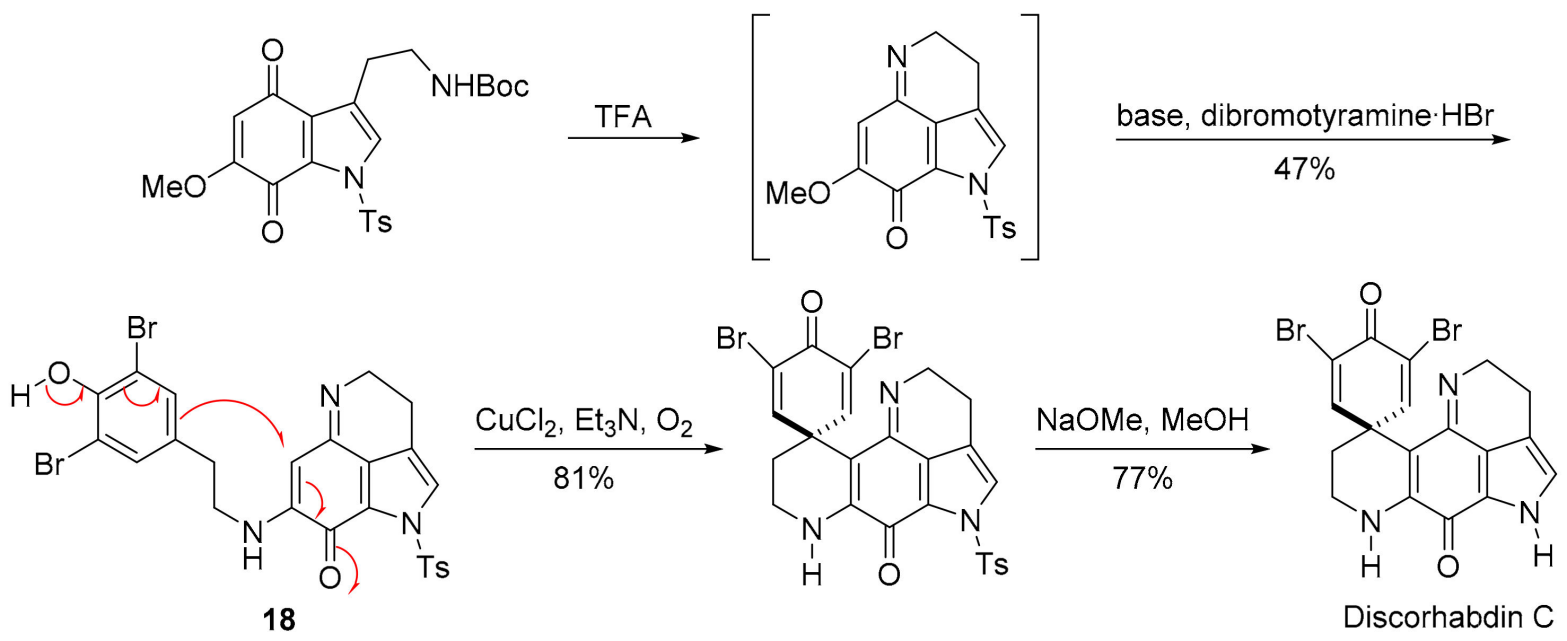
Aubart and Heathcock synthesis of discorhabdin C [111].

In the 1990s, many multi-step total syntheses of discorhabdin C were reported, with approaches starting from the quinoline with the formation of the fused pyrrole [107,108], and from the indole with the formation of the six-membered imino-ring through condensation with the quinone [109,110,111]. One approach involves oxidation of the tyramine-substituted indoloquinonimine **18** in a nucleophilic addition onto the iminoquinone (Scheme 13) [111]. Kublak and Confalone had earlier used the *para*-phenoxide approach for alkylation of an adjacent naphthoquinone ethylamino-substituent by displacement of a mesylate [112].

### 3.3. COMPARE Analysis: Strong Correlations to DPIQ and Benzo[1,2,4]triazin-7-one ***5a*** as the Seed: Quinoline-5,8-diones

**Figure 14:**
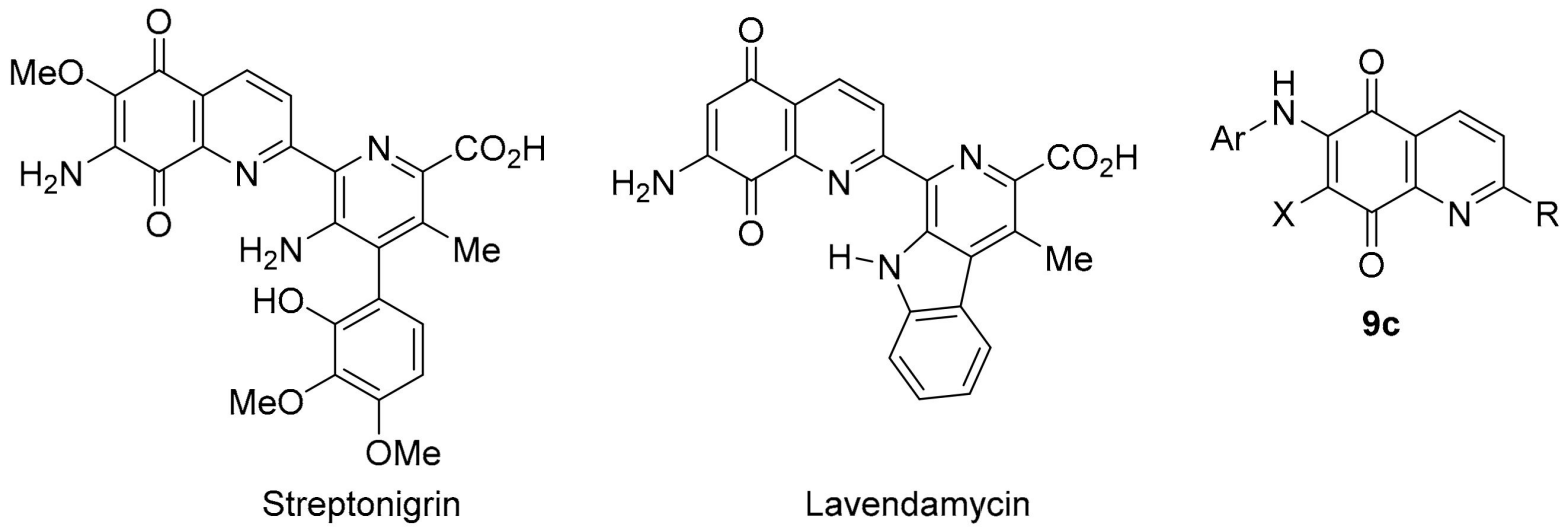
Popular quinoline-5,8-dione scaffolds for biological activity investigations.

Synthetic quinoline-5,8-diones scaffolds correlate strongly with the anti-cancer activities of DPIQ, benzotriazinone **5a**, and pleurotin. 6-Aminoethyl substituted derivative **9a** shows one of the strongest PCC to DPIQ of 0.80, with a relatively weak PCC to NQO1 expression of 0.42 (Figure 4). 2-Methyl substituted derivative **9b** shows a very strong PCC to pleurotin of 0.89, and a strong correlation to benzotriazinone **5a** of 0.71 (Figure 9). There is an abundance of synthetic and anti-cancer studies on quinoline-5,8-diones [113], with most stimulated by the broad range of cytotoxicity against solid tumours displayed by streptonigrin, a recognised substrate for NQO1 (Figure 14) [114]. Streptonigrin was isolated from *Streptomyces flocculus* [115], with lavendamycin a biosynthetically related antibiotic isolated from *Streptomyces lavendulae* [116]. Unfortunately, both streptonegrin and lavendamycin have proved too toxic for clinical use, although analogues of lavendamycin display potent HIV reverse-transcription inhibition [117], and improved specificity for NQO1 [118].

*N*-Alkylamino compounds, analogous to dione **9a**, also possess antimalarial activity [119], while compound **9b** is a reported inhibitor of Cdc25B (IC_50_ = 4.6 µM), a protein phosphatase involved in regulating cyclin-dependent kinase activity during the cell cycle [120]. Recent studies have reported 6-*N*-arylquinoline-5,8-diones **9c** (Figure 14) as inhibitors of *Mycobacterium tuberculosis* [121], as well as of Gram-negative and Gram-positive bacteria [122]. 6-*N*-Arylquinoline-5,8-diones **9c** are also reported to cleave DNA as an underlying mechanism for apoptosis induction in leukemic cell lines [123]. Nevertheless, the consensus is that there is a strong correlation between NQO1 bioreduction and anticancer activity for many quinoline-5,8-diones [118,124,125], given that NQO1 is strongly over-expressed in solid tumours [13] relative to normal tissues [126].

### 3.4. COMPARE Analysis Using Molecular Target Expression

In this section, the COMPARE algorithm was used to derive PCCs for the similarity in expression of chosen cancer molecular targets, NQO1 and TrxR, to compound growth inhibition across the NCI-60 cell line panel. Section 3.4.1 deals with NQO1 expression and establishes the strongest correlations to compound cytotoxicity. Section 3.4.2 reveals the PCCs of compounds with almost perfect direct correlations to the anti-cancer activity of known TrxR inhibitors, benzo[1,2,4]triazinones **5a**–**d** and pleurotin.

#### 3.4.1. Compound Correlations to NQO1 Expression

**Figure 15:**
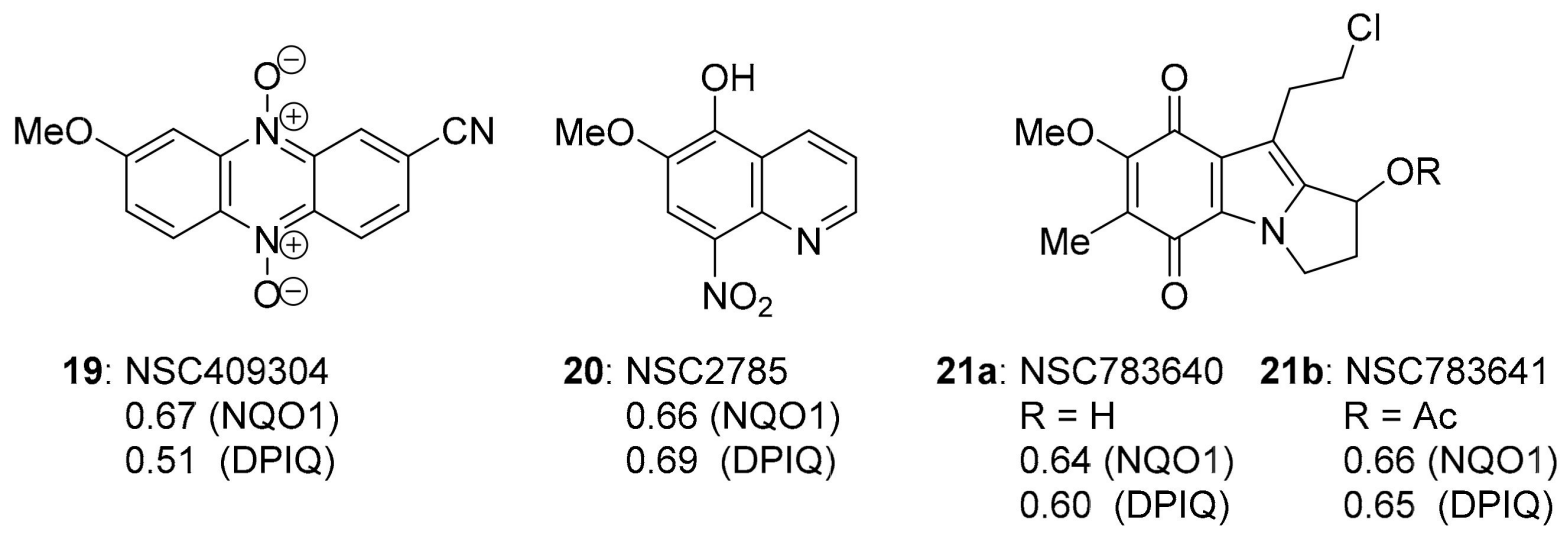
The three strongest PCCs to NQO1.

Since most compounds that correlated strongly with DPIQ anti-cancer activity gave modest PCCs of 0.38–0.55 to NQO1 expression, except for benzo[*e*]perimidine **3a**, which was noticeably stronger (PCC = 0.64, Figure 1 and Figure 4), we searched for compounds with the strongest PCCs to NQO1 expression. The strongest compound correlations to NQO1 were of similar magnitude to the PCC for benzo[*e*]perimidine **3a** (Figure 15). Phenazine-5,10-dioxide **19**, 5-hydroxy-6-methoxy-8-nitroquinoline **20**, and indolequinones **21a** and **21b**, gave PCCs of ~0.6–0.7 to NQO1 and DPIQ. Phenazine-5,10-dioxide **19** is unreported, and has a marginally lower PCC of 0.51 than the other NQO1 substrates to DPIQ.

**Figure 16:**
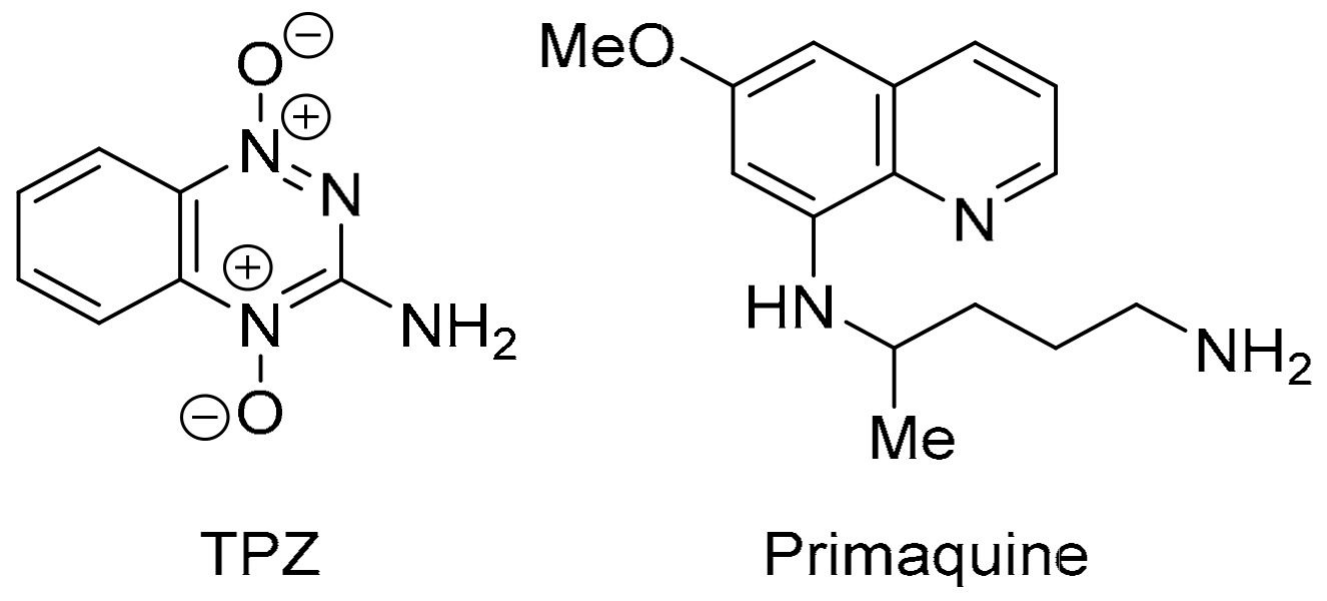
TPZ and primaquine related to **19** and **20**, respectively.

Analogues of phenazine **19** are reported as π-stacking DNA intercalators with differential toxicity through DNA-damaging ^●^OH release under hypoxic conditions [127,128]. Phenazine-5,10-dioxides are designed to model 3-amino-1,2,4-benzotriazine-1,4-dioxide (tirapazamine, TPZ, Figure 16), which reached advanced clinical trials as a hypoxia-activated prodrug [129]. However, although there are successful Phase I and Phase II trials, Phase III randomised controlled trials showed no benefit of TPZ in chemotherapy without using an approach to ensure sustained tumour hypoxia [130]. Quinoline **20** is a synthetic precursor for 5-alkoxy derivatives of the clinical anti-malarial primaquine [131]. The biological activity of **20** is unreported, and, tentatively, the compound is metabolised to a quinoline-5,8-dione antibiotic upon reductive activation-oxidation (Section 3.3).

**Scheme 14:**
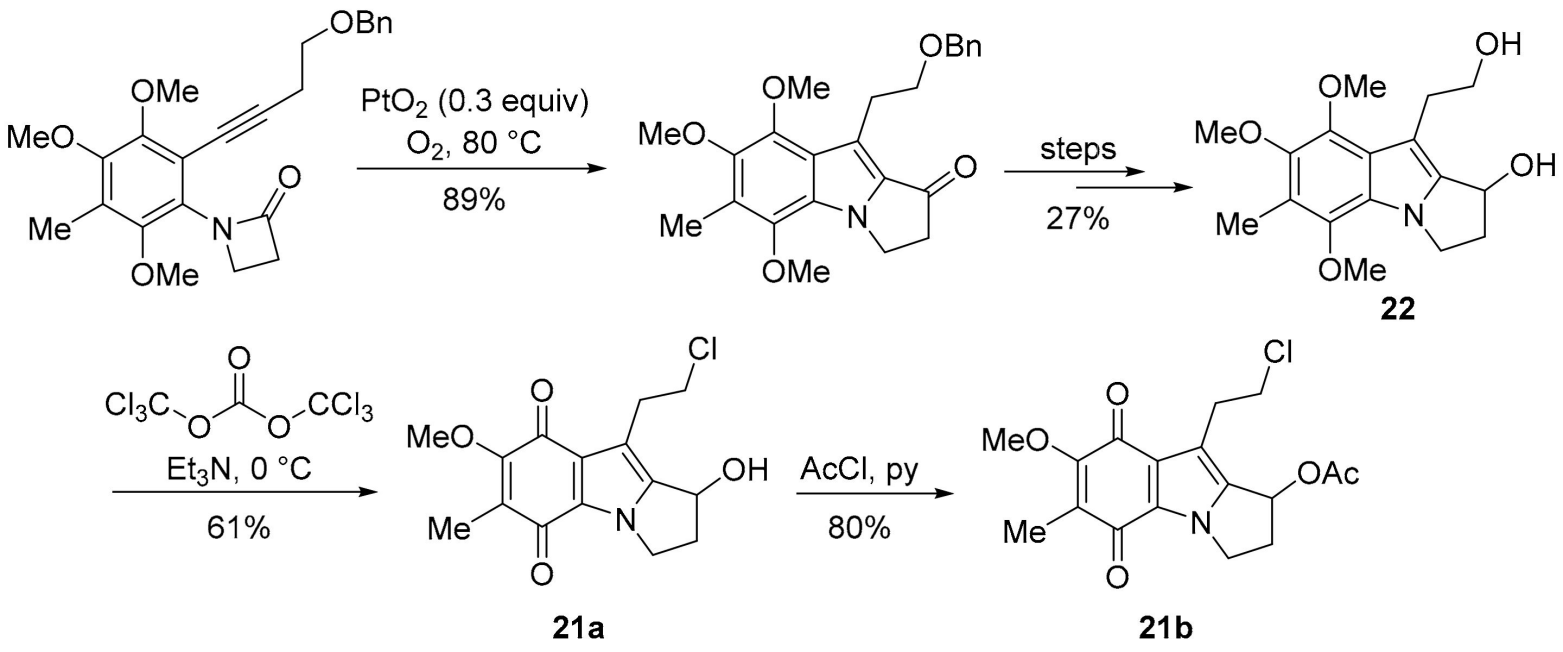
Cycloisomerisation to the 7-methoxymitosene skeleton [134,135].

Pyrrolo[1,2-*a*]indoles are highly pursued synthetic targets [132], because they form the core of mitomycins, in particular MMC (Figure 3) [133]. The 7-methoxymitosene skeleton of **21a** and **21b** was accessed via a Pt-promoted cyclisation of a *β*-lactam onto an internal acetylene (Scheme 14) [134]. Triphosgene and triethylamine preferentially chlorinate the primary alcohol over the secondary alcohol in **22** to give **21a** [135], with the substitution reported to be driven by steric demand [136].

**Scheme 15:**
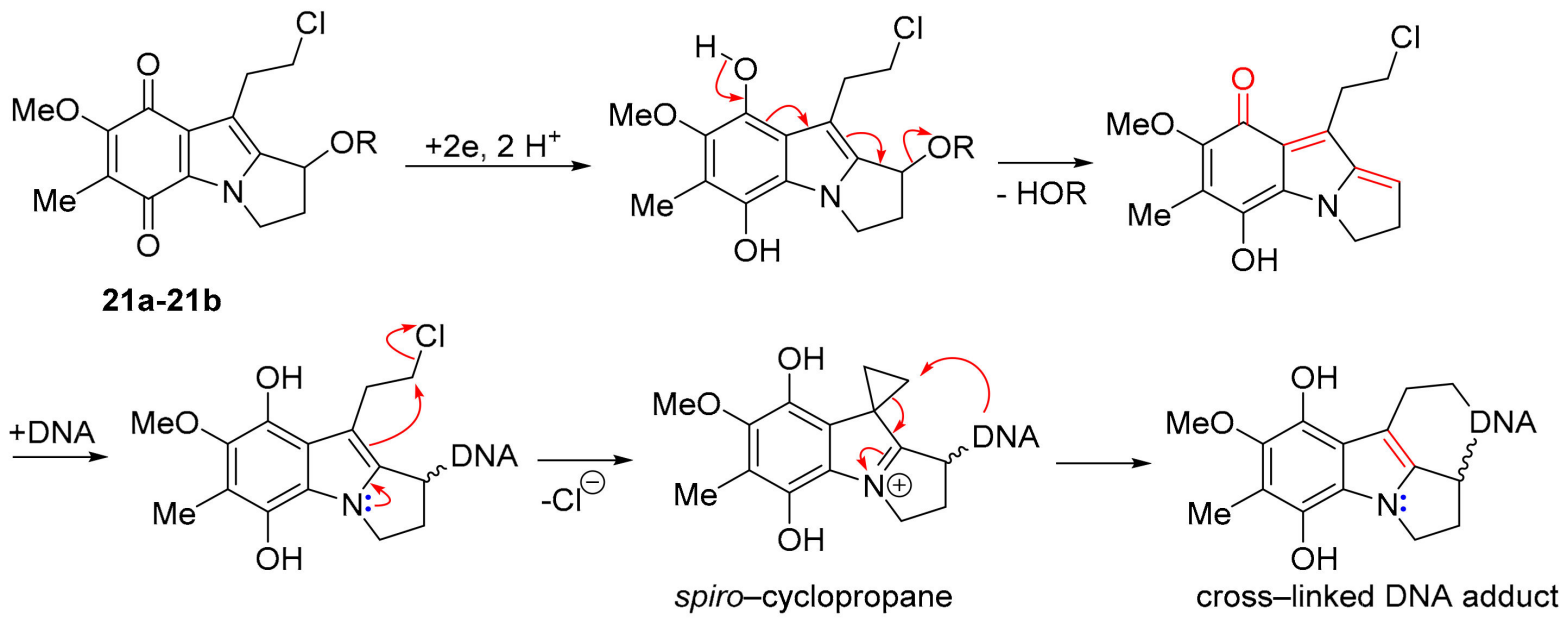
Hypothesis for cytotoxicity of 7-methoxymitosenes [135].

7-Methoxymitosenes **21a** and **22b** are designed to form an electrophilic *spiro*-cyclopropane intermediate upon reductive activation, which enables crosslinking, possibly within the same DNA molecule (Scheme 15) [135]. Indolequinones **21a** and **21b** were evaluated using the prostate cancer cell line PPC-1 and the normal prostate cell line RWPE-1, with alcohol **21a** exhibiting the greater selectivity towards the cancer cell line [135].

Interestingly, known TrxR inhibitors, benzo[1,2,4]triazinones **5a, 5b** and pleurotin, as well as compounds that correlate very strongly to their anti-cancer activity, benzo[*a*]carbazole-1,4-dione **14b**, kalafungin, and discorhabdin C, gave negative PCCs to NQO1 expression across the NCI-60 panel (PCC = −0.27 to −0.48, Table 1). This suggests that TrxR inhibitors may also act as inhibitors of other two-electron reductases over-expressed in solid tumours, namely NQO1 (see discussion below).

#### 3.4.2. Compound Correlations to TrxR

**Figure 17:**
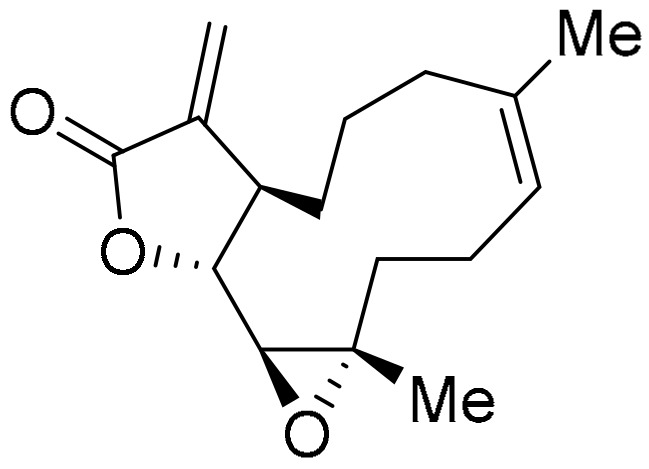
Parthenolide.

The negative correlations to TrxR (TXNRD1) expression by reported TrxR-Trx inhibitory compounds [10,11,15,19,137], as well as by compounds with very strong similarities in anti-cancer activity, suggest that the cytotoxicity of these compounds is greater when TrxR levels are low (Table 1). Increased inhibition may, thus, prevent the reducing system’s essential role in tumour development, such as inhibiting apoptosis, and angiogenesis promotion [19,137]. The TrxR-Trx system modulates cell signalling, in particular through interactions with the tumour suppressor protein PTEN (protein tyrosine phosphatase and tensin homolog). The TrxR-Trx system activates PTEN through reduction [138], with oxidation of PTEN leading to inactivation [138,139]. Trx-1–PTEN interactions through disulfide bond formation between Cys 32 in the Trx-1 active site and Cys 212 in the lipid membrane binding domain of PTEN inhibits PTEN phosphatase activity [140]. The inactivation of PTEN leads to activation of tumour propagating PI3K-AKT kinase signalling pathways [138]. The naturally occurring sesquiterpene lactone, parthenolide (structurally similar to MMB, Figure 9), is reported to inhibit TrxR by shifting the enzyme from antioxidant activity to ROS generation, leading to promotion of apoptosis in HeLa cells (Figure 17) [141]. Cell death through ROS generation seems a common mode of action for most classes of compounds inhibiting TrxR [19,93,137,141,142]. Alternatively, the greater potency of compounds as cytotoxins when reductase expression is low may be due to the TrxR-Trx or NQO1 systems detoxifying the heterocyclic (imino)quinones through bioreduction (Scheme 2). Further, COMPARE reveals a diverse range of chemical structures, including many structures without the (imino)quinone moiety, that have strong PCCs to the anti-cancer activity of TrxR inhibitors, **5a**, **5b** and pleurotin. Since benzotriazinones **5a** and **5b** exhibit reversible mixed and uncompetitive inhibition of TrxR, respectively [10,137], with binding to positions other than the active site likely, it seems that bioreduction may not be directly involved in the inhibition or inactivation of TrxR by many compounds.

**Figure 18:**
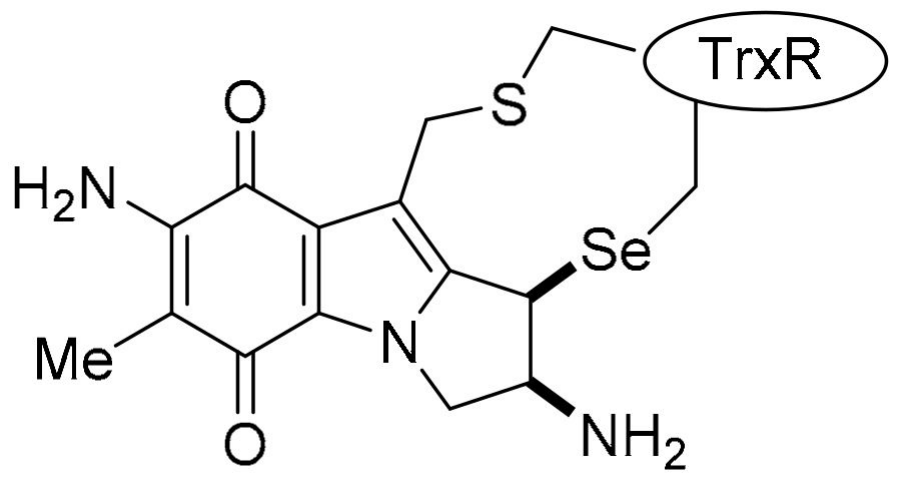
Inactivation of TrxR: MMC-TrxR adduct [143].

Importantly, specificity for NQO1 was demonstrated by DPIQ, benzo[*e*]perimidine **3a**, 2-aminophenoxazinone **4** and phenazine **19**, which have no correlations to TrxR (TXNRD1: PCC = −0.15 to 0.12), but moderate to strong correlations to NQO1 expression across the NCI-60 cell line panel (PCC = 0.47–0.67, Table 2). This supports the premise that these compounds are specifically substrates for cellular NQO1, which facilitates reductively-activated cytotoxicity. Further, MMC and other recognised indolequinone NQO1 substrates [7,13,143,144,145], are shown to irreversibly inhibit TrxR through formation of covalently bound adducts (Figure 18) [135,143,145]. Comparing mean growth (inhibition) graphs of NQO1 substrates, the greatest cytotoxicity is towards solid tumour cell lines, including most melanoma, non-small cell lung, and colon cancer cell lines, with negligible toxicity towards leukemic cell lines, in contrast with the strong anti-leukemic activity of TrxR inhibitors (see Supplementary Materials).

## 4. Conclusions

The NCI COMPARE program has enabled the composition of this review on the synthesis and biological activity of heterocyclic iminoquinones and quinones, where scaffolds are categorised according to similarities in their anti-cancer activity. Although it is important to emphasise that all correlations herein need to be verified experimentally [2], as with many literature studies [1,2,3,4,5,146], COMPARE has enabled hypotheses of mechanisms of actions to be made. We reveal several natural products with strong to very strong correlations in patterns of anti-cancer activity to DPIQ, benzo[1,2,4]triazin-7-one **5a**, and pleurotin. Most heterocyclic scaffolds exhibit significant biological activity to warrant extensive synthetic investigations, while others are yet to be synthesized and are revealed as important future targets for medicinal chemists.

Compounds strongly correlating to DPIQ, and showing specificity to NQO1 expression, have no correlation to the alternative two-electron reductase, TrxR. These “NQO1 specific prodrugs” are flat aromatic heterocycles with fused oxidizable, e.g., quinone or iminoquinone, moieties. The correlations to NQO1 expression, however, are modest, suggesting that NQO1 is not the sole cellular molecular component that influences cell sensitivity. NQO1 has a “Janus” effect in cancer biology [18], where it behaves as either a tumour suppressor or a tumour promotor, with the former based on the prevention of SET processes that lead to an accumulation of harmful ROS. However, the redox appears interchangeable, and NQO1-activated pathways can lead to ROS-induced apoptosis [18,21]. Ultimately, designed prodrugs for NQO1 may also effectively act as inhibitors of redox defensive signalling pathways that contribute to carcinogenesis, including the transcriptional regulators Nrf2 [25], HIF-1α [43], and JAK/STAT [71].

There is a diverse range of compounds with almost direct correlations to the anti-cancer activity patterns of the TrxR inhibitors, benzotriazinone **5a**, and pleurotin. These compounds do not always possess an oxidising (iminoquinone or quinone) group, suggesting bioreduction is unnecessary, and compounds are more cytotoxic in the absence of the reductase. Among these compounds, there are similarities in biological activity, with many possessing antiviral, antimicrobial (including antimalarial), as well as anti-cancer activity. These compounds tend to be cytotoxic towards leukemic as well as solid tumour cell lines, unlike NQO1 substrates, which show specificity towards solid tumours over-expressing NQO1. There are similarities in the mechanism of action, with compounds correlating to NQO1 expression or acting as TrxR inhibitors, both showing inhibition of the monomeric oxidase, IDO1 [50]. 1,3-Diphenylbenzo[1,2,4]triazinones are inhibitors in AD [14], as well as cancer [10,11,137]. Reduced Trx protein binds to apoptosis signal-regulating kinase 1 (ASK1), thus inhibition of TrxR leads to oxidised Trx, which cannot bind to ASK1. The outcome is mitochondrial apoptosis through activation of ASK1, downstream JNK and mitogen-activated protein kinase (MAPK14) signalling pathways [145], which lead to reduced inflammatory response and tumorigenesis or the initiating of neurodegenerative disorders, such as AD [137,142].

## Figures and Tables

**Table 1:** COMPARE analysis-derived PCCs to NQO1 and TrxR expression across the NCI-60 panel for TrxR inhibitors ^1^.

Compound	NQO1	TrxR
**5a**	−0.37	−0.24
**5b**	−0.30	−0.12
Pleurotin	−0.27	−0.21
**14b**	−0.48	−0.22
Kalafungin	−0.27	−0.24
Discorhabdin C	−0.29	−0.37

^1^ See Section 2 for the method [6,7,23].

**Table 2:** COMPARE analysis-derived PCCs to NQO1 and TrxR expression across the NCI-60 panel for NQO1 substrates ^1^.

Compound	NQO1	TrxR
DPIQ	0.51	0.12
**3a**	0.64	0.11
**4**	0.47	−0.15
**19**	0.67	0.10

^1^ See Section 2 for the method [6,7,23], PCC for DPIQ, **3a** and **4** with NQO1 previously reported [7].

## Data Availability

Not applicable.

## Supplementary Materials

The following supporting information can be downloaded at: https://www.mdpi.com/article/10.3390/molecules28135202/s1.
