# Supplementary material for: Heterocyclic Iminoquinones and Quinones from the National Cancer Institute (NCI, USA) COMPARE Analysis
Source: Molecules. 2023 Jul 4;28(13):5202. doi: 10.3390/molecules28135202 (PMC10343902; doi:10.3390/molecules28135202)
Supplement: Supplementary file 1 [file molecules-28-05202-s001.zip › molecules-2459210-supplementary.pdf]

Heterocyclic Iminoquinones and Quinones from National Cancer Institute (USA) COMPARE Analysis

Naemah Haji [1‡], Masoma Faizi [1‡], Panayiotis A. Koutentis [2], Michael P. Carty [3] and Fawaz Aldabbagh [1,*]

[1] Department of Pharmacy, School of Life Sciences, Pharmacy and Chemistry, Kingston University, Penrhyn Road, Kingston upon Thames, KT1 2EE, UK
[2] Department of Chemistry, University of Cyprus, PO Box 20537, 1678 Nicosia, Cyprus
[3] School of Biological and Chemical Sciences, National University of Ireland Galway, University Road, H91 TK33 Galway, Ireland

‡ Equal contributions
* Correspondence: F.Aldabbagh@kingston.ac.uk

Supplementary Materials

Correlation of DPIQ (NSC753790) to NQO1	*Figure S1*
Correlation of DPIQ (NSC753790) to **3a** (NSC84167)	*Figure S2*
Correlation of **3a** (NSC84167) to NQO1	*Figure S3*
Correlation of DPIQ (NSC753790) to **4** (NSC94945)	*Figure S4*
Correlation of **4** (NSC94945) to NQO1	*Figure S5*
Correlation of **5a** (NSC768093) to Pleurotin (NSC401005)	*Figure S6*
Correlation of **5b** (NSC780813) to Pleurotin (NSC401005)	*Figure S7*
Correlation of **5a** (NSC768093) to **5b** (NSC780813)	*Figure S8*
Correlation of **5c** (NSC780816) to Pleurotin (NSC401005)	*Figure S9*
Correlation of **5a** (NSC768093) to **5c** (NSC780816)	*Figure S10*
Correlation of **5d** (NSC780815) to Pleurotin (NSC401005)	*Figure S11*
Correlation of **5a** (NSC768093) to **5d** (NSC780815)	*Figure S12*
Correlation of DPIQ (NSC753790) to **7** (NSC643735)	*Figure S13*
Correlation of **7** (NSC643735) to NQO1	*Figure S14*
Correlation of DPIQ (NSC753790) to **8a** (NSC795222)	*Figure S15*
Correlation of **8a** (NSC795222) to NQO1	*Figure S16*
Correlation of DPIQ (NSC753790) to **9a** (NSC291094)	*Figure S17*
Correlation of **9a** (NSC291094) to NQO1	*Figure S18*
Correlation of DPIQ (NSC753790) to **10a** (NSC798710)	*Figure S19*
Correlation of **10a** (NSC798710) to NQO1	*Figure S20*
Correlation of DPIQ (NSC753790) to **10b** (NSC798709)	*Figure S21*
Correlation of **10b** (NSC798709) to NQO1	*Figure S22*
Correlation of DPIQ (NSC753790) to **11b** (NSC750104)	*Figure S23*
Correlation of **11b** (NSC750104) to NQO1	*Figure S24*
Correlation of DPIQ (NSC753790) to **12** (NSC647943)	*Figure S25*
Correlation of **12** (NSC647943) to NQO1	*Figure S26*
Correlation of **5a** (NSC768093) to **13a** (NSC787555)	*Figure S27*
Correlation of **13a** (NSC787555) to Pleurotin (NSC401005)	*Figure S28*
Correlation of **5a** (NSC768093) to **13b** (NSC787553)	*Figure S29*
Correlation of **13b** (NSC787553) to Pleurotin (NSC401005)	*Figure S30*
Correlation of **5a** (NSC768093) to **13c** (NSC787554)	*Figure S31*
Correlation of **13c** (NSC787554) to Pleurotin (NSC401005)	*Figure S32*
Correlation of **5a** (NSC768093) to **13d** (NSC791347)	*Figure S33*
Correlation of **13d** (NSC791347) to Pleurotin (NSC401005)	*Figure S34*
Correlation of **5a** (NSC768093) to **14a** (NSC92937)	*Figure S35*
Correlation of **14a** (NSC92937) to Pleurotin (NSC401005)	*Figure S36*
Correlation of **5a** (NSC768093) to **14b** (NSC668844)	*Figure S37*
Correlation of **14b** (NSC668844) to Pleurotin (NSC401005)	*Figure S38*

Correlation of **5a** (NSC768093) to **14c** (NSC661222) *Figure S39*
Correlation of **14c** (NSC661222) to Pleurotin (NSC401005) *Figure S40*
Correlation of **5a** (NSC768093) to **14d** (NSC645330) *Figure S41*
Correlation of **14d** (NSC645330) to Pleurotin (NSC401005) *Figure S42*
Correlation of **5a** (NSC768093) to **14e** (NSC661221) *Figure S43*
Correlation of **14e** (NSC661221) to Pleurotin (NSC401005) *Figure S44*
Correlation of **5a** (NSC768093) to **14f** (NSC641396) *Figure S45*
Correlation of **14f** (NSC641396) to Pleurotin (NSC401005) *Figure S46*
Correlation of **5a** (NSC768093) to Kalafungin (NSC137443) *Figure S47*
Correlation of Kalafungin (NSC137443) to Pleurotin (NSC401005) *Figure S48*
Correlation of **5a** (NSC768093) to Nanomycin A (NSC267461) *Figure S49*
Correlation of Nanomycin A (NSC267461) to Pleurotin (NSC401005) *Figure S50*
Correlation of **5a** (NSC768093) to **15a** (NSC641401) *Figure S51*
Correlation of **15a** (NSC641401) to Pleurotin (NSC401005) *Figure S52*
Correlation of **5a** (NSC768093) to Discorhabdin C (NSC626162) *Figure S53*
Correlation of Discorhabdin C (NSC626162) to Pleurotin (NSC401005) *Figure S54*
Correlation of **5a** (NSC768093) to **9b** (NSC682995) *Figure S55*
Correlation of **9b** (NSC682995) to Pleurotin (NSC401005) *Figure S56*
Correlation of **5a** (NSC768093) to Melampomagnolide B (MMB) analogue (NSC801078) *Figure S57*
Correlation of MMB analogue (NSC801078) to Pleurotin (NSC401005) *Figure S58*
Correlation of NQO1 to **19** (NSC409304) *Figure S59*
Correlation of **19** (NSC409304) to DPIQ (NSC753790) *Figure S60*
Correlation of NQO1 to **20** (NSC2785) *Figure S61*
Correlation of **20** (NSC2785) to DPIQ (NSC753790) *Figure S62*
Correlation of NQO1 to **21a** (NSC783640) *Figure S63*
Correlation of **21a** (NSC783640) to DPIQ (NSC753790) *Figure S64*
Correlation of NQO1 to **21b** (NSC783641) *Figure S65*
Correlation of **21b** (NSC783641) to DPIQ (NSC753790) *Figure S66*
Correlation of NQO1 to **5a** (NSC768093) *Figure S67*
Correlation of NQO1 to **5b** (NSC780813) *Figure S68*
Correlation of NQO1 to Pleurotin (NSC401005) *Figure S69*
Correlation of NQO1 to **14b** (NSC668844) *Figure S70*
Correlation of NQO1 to Kalafungin (NSC137443) *Figure S71*
Correlation of NQO1 to Discorhabdin C (NSC626162) *Figure S72*
Correlation of TrxR to **5a** (NSC768093) *Figure S73*
Correlation of TrxR to **5b** (NSC780813) *Figure S74*
Correlation of TrxR to Pleurotin (NSC401005) *Figure S75*
Correlation of TrxR to **14b** (NSC668844) *Figure S76*
Correlation of TrxR to Kalafungin (NSC137443) *Figure S77*
Correlation of TrxR to Discorhabdin C (NSC626162) *Figure S78*
Correlation of TrxR to DPIQ (NSC753790) *Figure S79*
Correlation of TrxR to **3a** (NSC84167) *Figure S80*
Correlation of TrxR to **4** (NSC94945) *Figure S81*
Correlation of TrxR to **19** (NSC409304) *Figure S82*

COMPARE Analysis of DPIQ (NSC753790) to NQO1

---SEED---
NSC:S753790 Endpoint:GI50 ExpId:AVGDATA logHiConc:-4.0
Id: 373263 Count Experiments: 2
---TARGET---
MoltId:MT22 Genecard:NQO1 TargetSet:MOLTID_MT_SERIES

Correlation: 0.51
JobId: 2835201994318757494

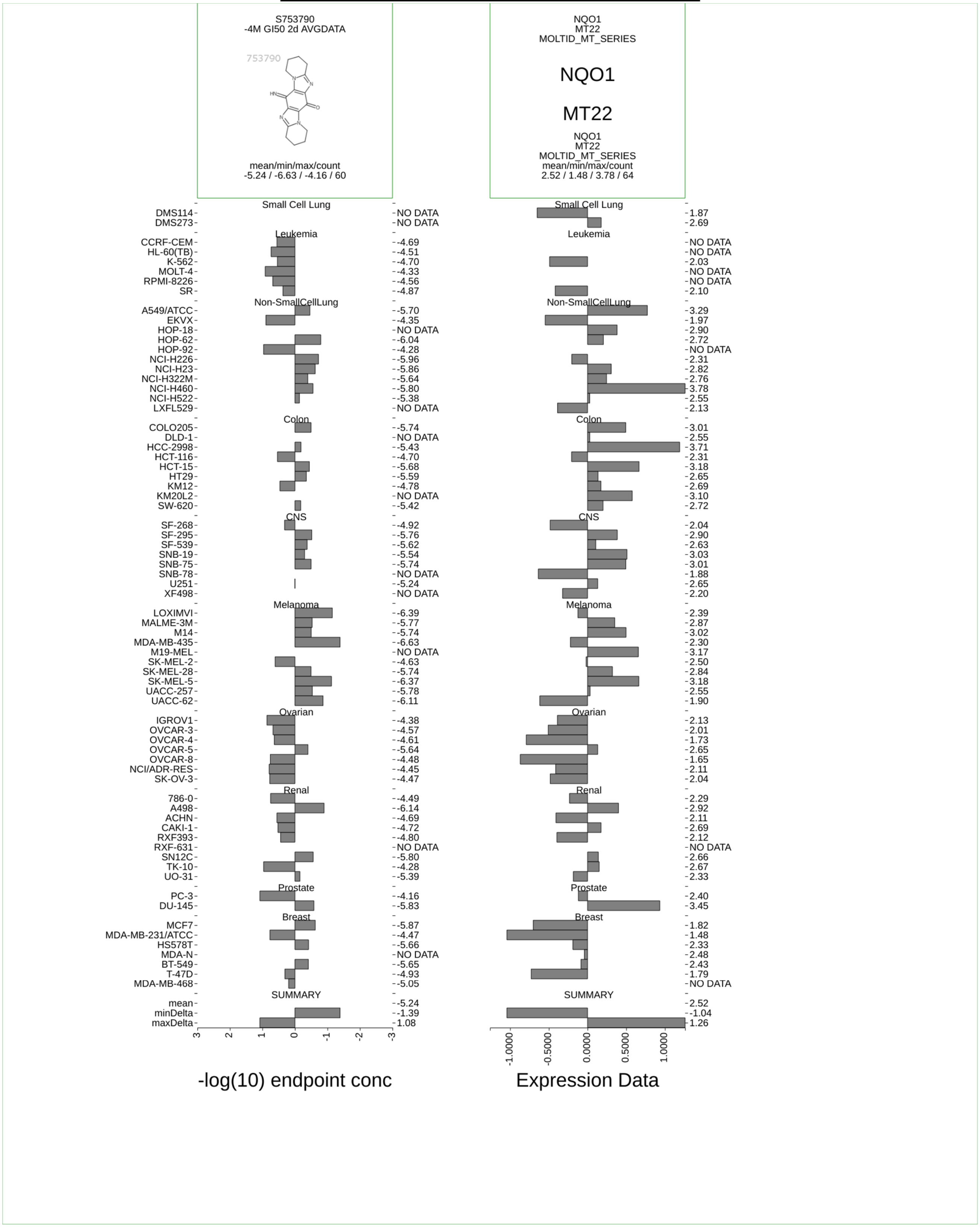

Figure S1

COMPARE Analysis of DPIQ (NSC753790) to **3a** (NSC84167)

---SEED---
NSC:S753790 Endpoint:GI50 ExpId:AVGDATA logHiConc:-4.0
Id: 373263 Count Experiments: 2
---TARGET---
NSC:S84167 Endpoint:GI50 ExpId:AVGDATA logHiConc:-4.0
Id: 372937 Count Experiments: 2

Correlation: 0.86
JobId: 8881243693771965163

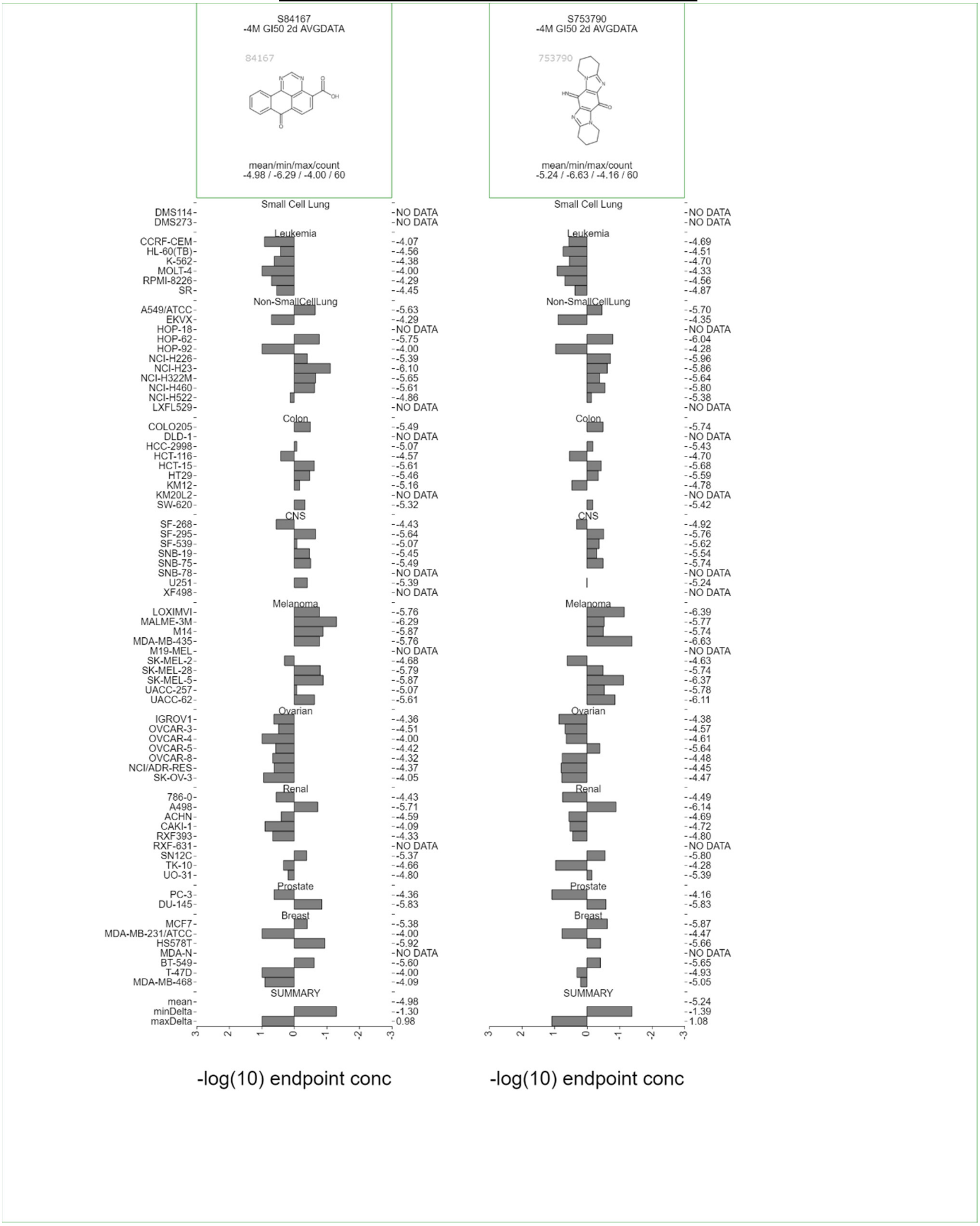

Figure S2

COMPARE Analysis of **3a** (NSC84167) to NQO1

---SEED---

NSC:S84167 Endpoint:GI50 ExpId:AVGDATA logHiConc:-4.0

Id: 372937 Count Experiments: 2

---TARGET---

MoltId:MT22 Genecard:NQO1 TargetSet:MOLTID_MT_SERIES

Correlation: 0.64

JobId: 4809108248135995984

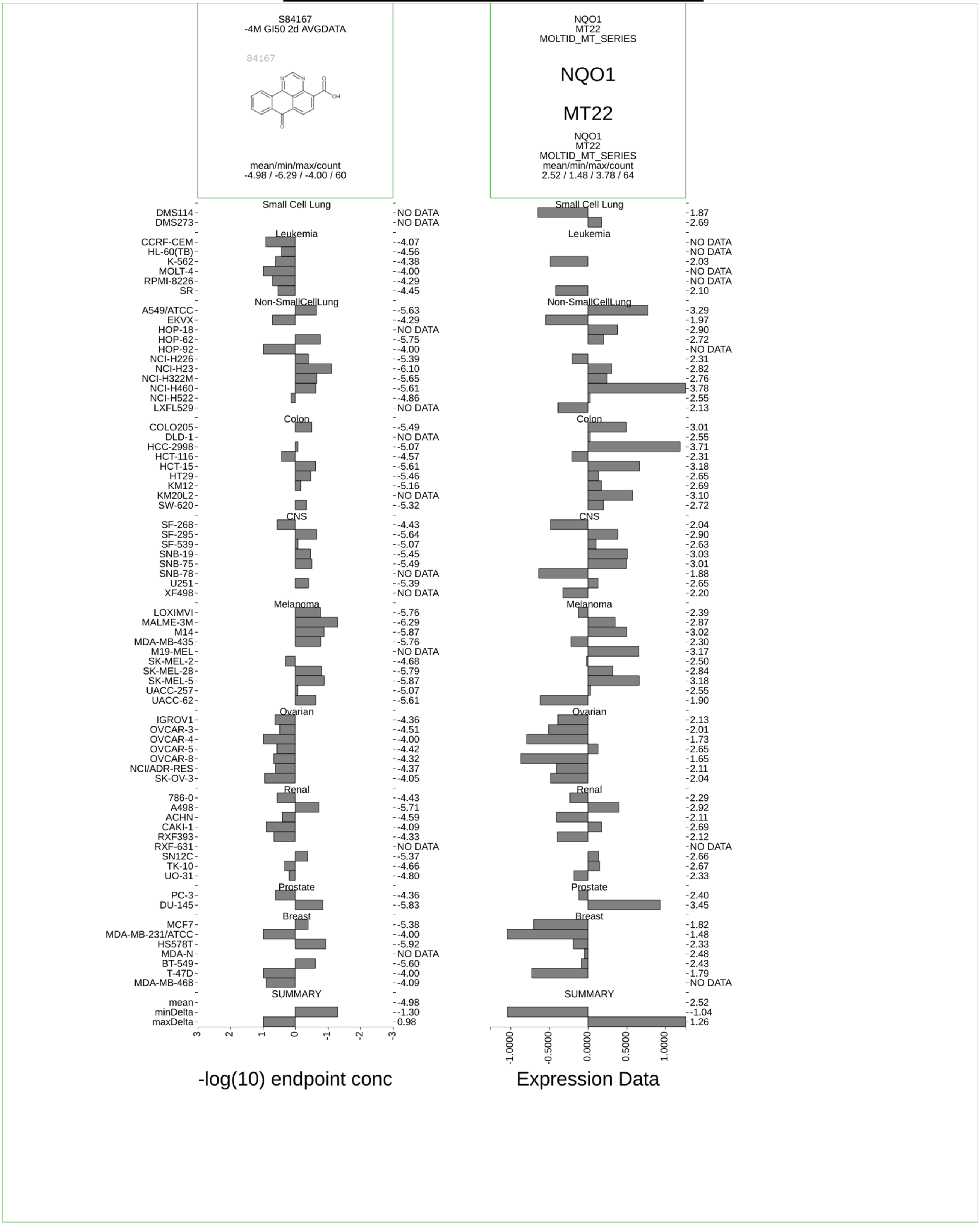

Figure S3

COMPARE Analysis of DPIQ (NSC753790) to **4** (NSC94945)

---SEED---
NSC:S753790 Endpoint:GI50 ExpId:AVGDATA logHiConc:-4.0
Id: 373263 Count Experiments: 2
---TARGET---
NSC:S94945 Endpoint:GI50 ExpId:AVGDATA logHiConc:-4.0
Id: 11715 Count Experiments: 2

Correlation: 0.77
JobId: 8226154143438375649

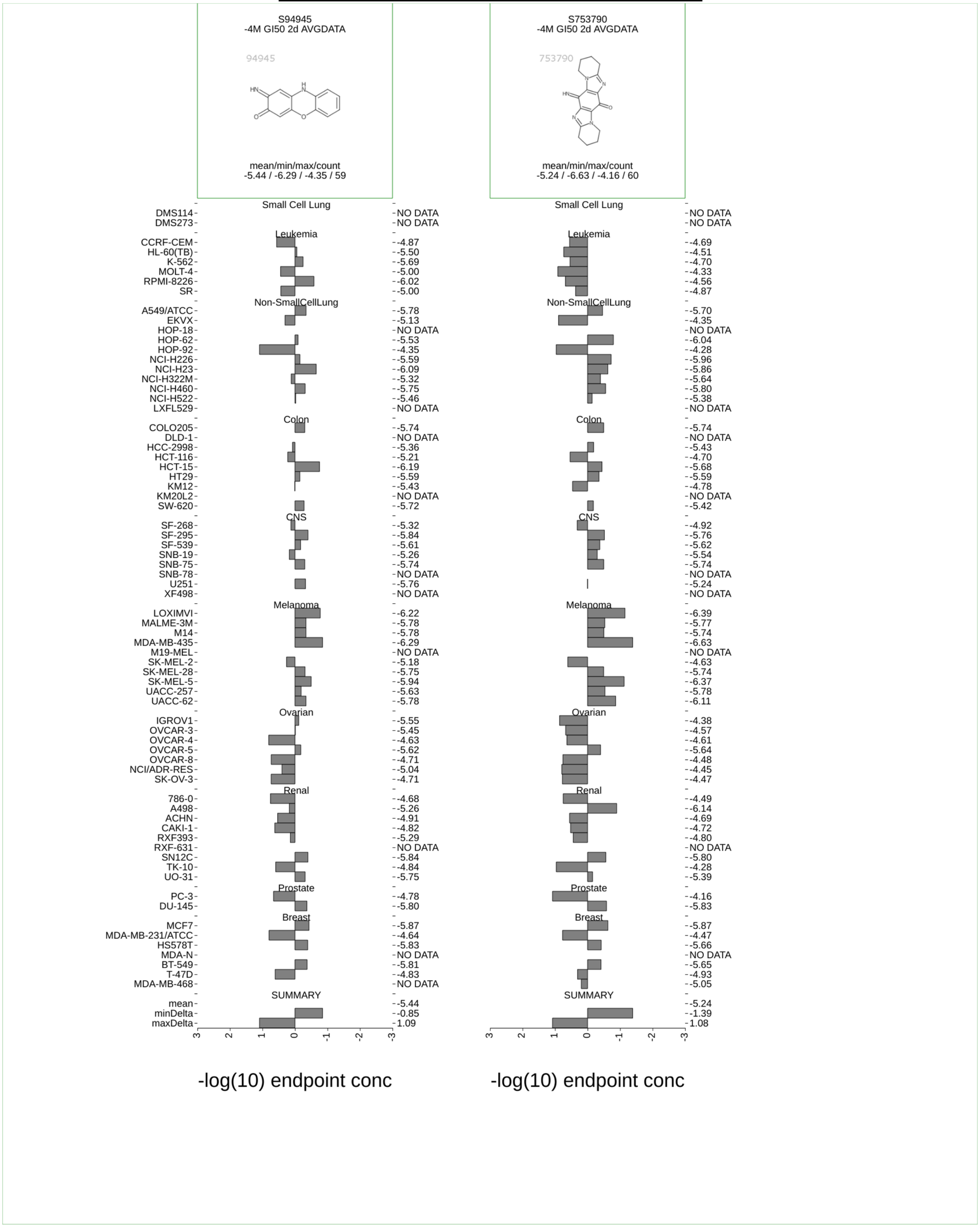

Figure S4

COMPARE Analysis of **4** (NSC94945) to NQO1

---SEED---
NSC:S94945 Endpoint:GI50 ExpId:AVGDATA logHiConc:-4.0
Id: 11715 Count Experiments: 2
---TARGET---
MoltId:MT22 Genecard:NQO1 TargetSet:MOLTID_MT_SERIES

Correlation: 0.47
JobId: 8449072155707022498

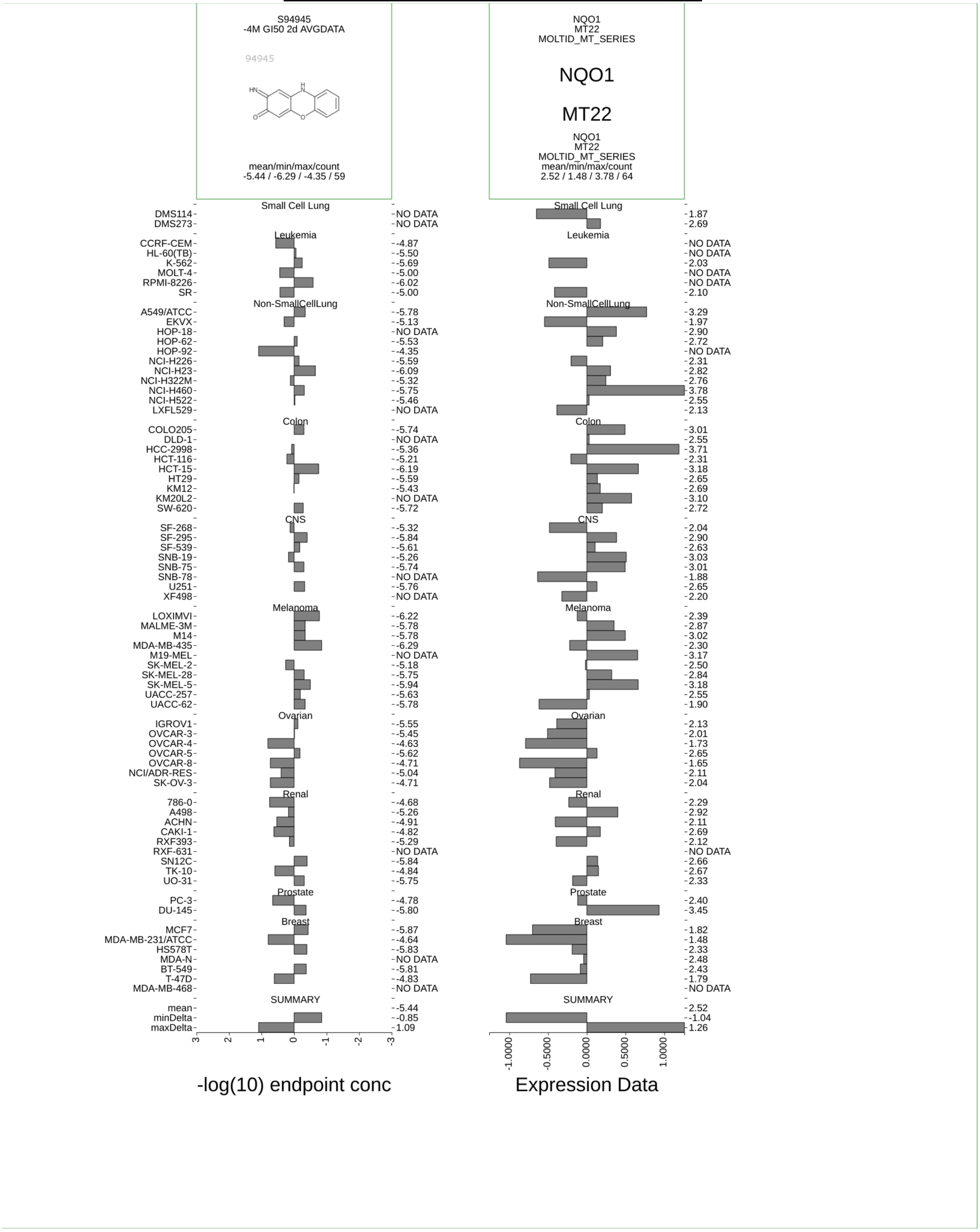

Figure S5

COMPARE Analysis of **5a** (NSC768093) to Pleurotin (NSC401005)

---SEED---
NSC:S768093 Endpoint:GI50 ExpId:AVGDATA logHiConc:-4.0
Id: 253700 Count Experiments: 2
---TARGET---
NSC:S401005 Endpoint:GI50 ExpId:AVGDATA logHiConc:-4.0
Id: 243481 Count Experiments: 2

Correlation: 0.84
JobId: 8898937600775438742

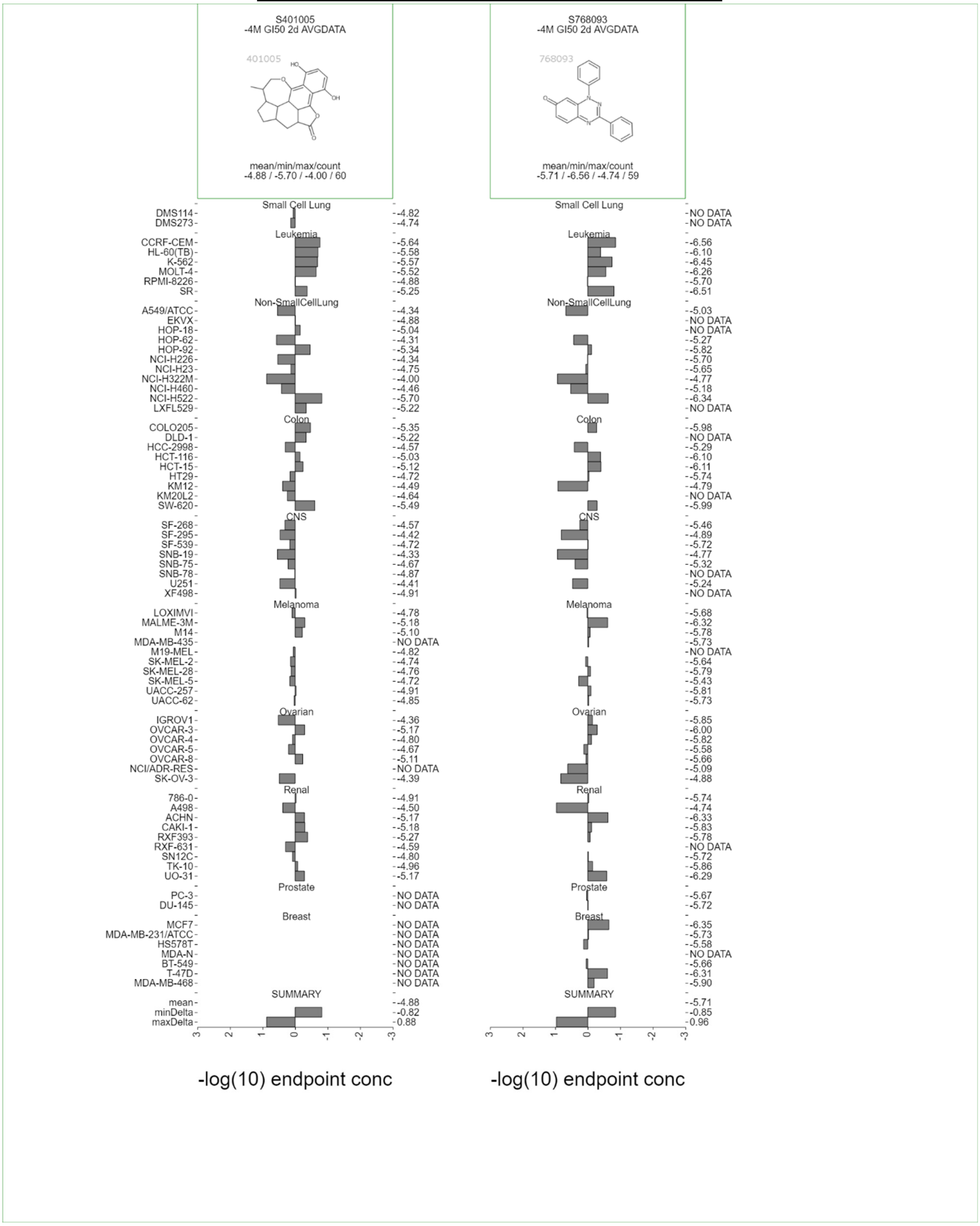

Figure S6

COMPARE Analysis of **5b** (NSC780813) to Pleurotin (NSC401005)

---SEED---
NSC:S780813 Endpoint:GI50 ExpId:AVGDATA logHiConc:-4.0
Id: 314185 Count Experiments: 1
---TARGET---
NSC:S401005 Endpoint:GI50 ExpId:AVGDATA logHiConc:-4.0
Id: 243481 Count Experiments: 2

Correlation: 0.80
JobId: 4376827337522088035

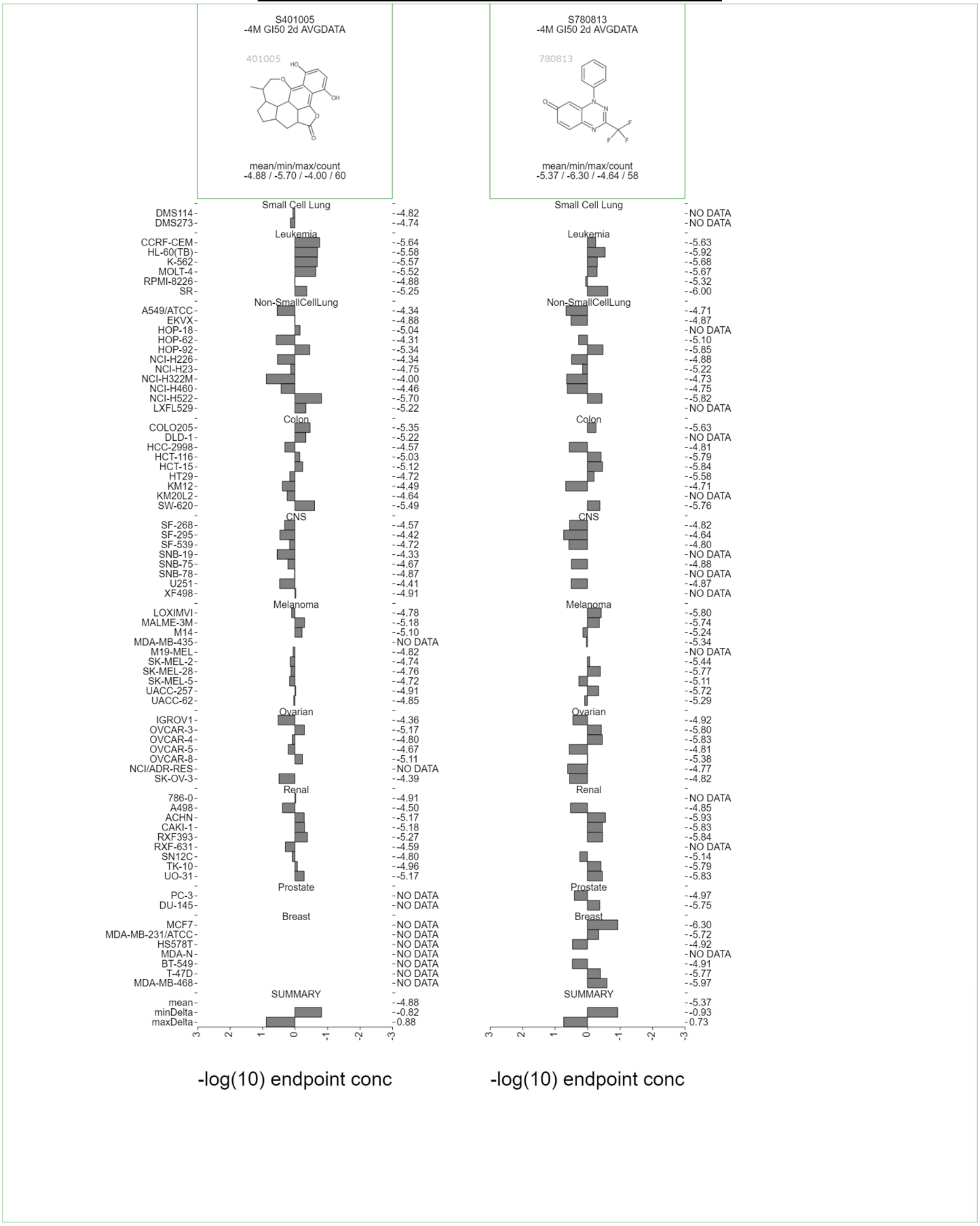

Figure S7

COMPARE Analysis of **5a** (NSC768093) to **5b** (NSC780813)

---SEED---
NSC:S768093 Endpoint:GI50 ExpId:AVGDATA logHiConc:-4.0
Id: 253700 Count Experiments: 2
---TARGET---
NSC:S780813 Endpoint:GI50 ExpId:AVGDATA logHiConc:-4.0
Id: 314185 Count Experiments: 1

Correlation: 0.80
JobId: 178613746223602897

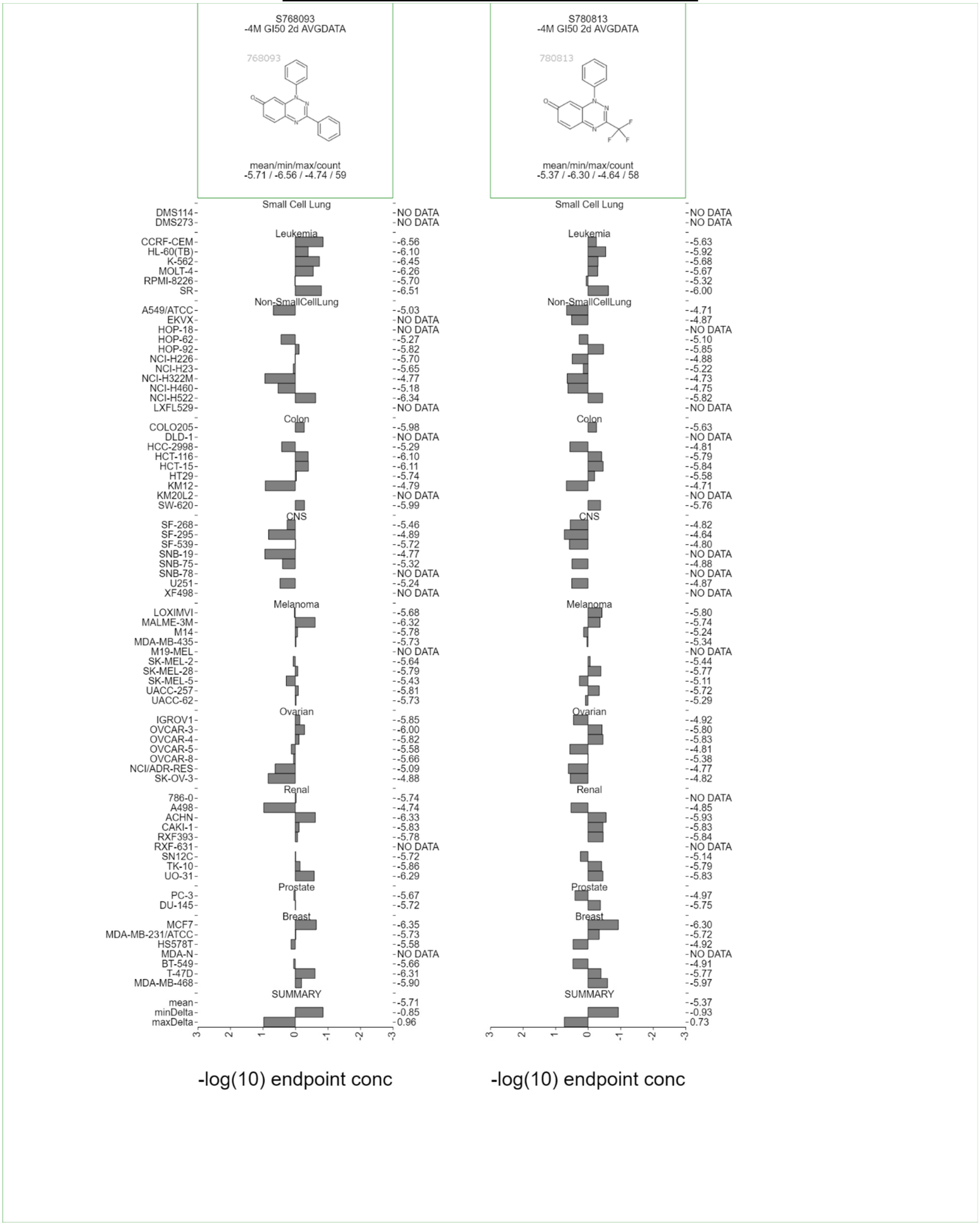

Figure S8

COMPARE Analysis of **5c** (NSC780816) to Pleurotin (NSC401005)

---SEED---
NSC:S780816 Endpoint:GI50 ExpId:AVGDATA logHiConc:-4.0
Id: 132828 Count Experiments: 1
---TARGET---
NSC:S401005 Endpoint:GI50 ExpId:AVGDATA logHiConc:-4.0
Id: 243481 Count Experiments: 2

Correlation: 0.84
JobId: 9115622029100680995

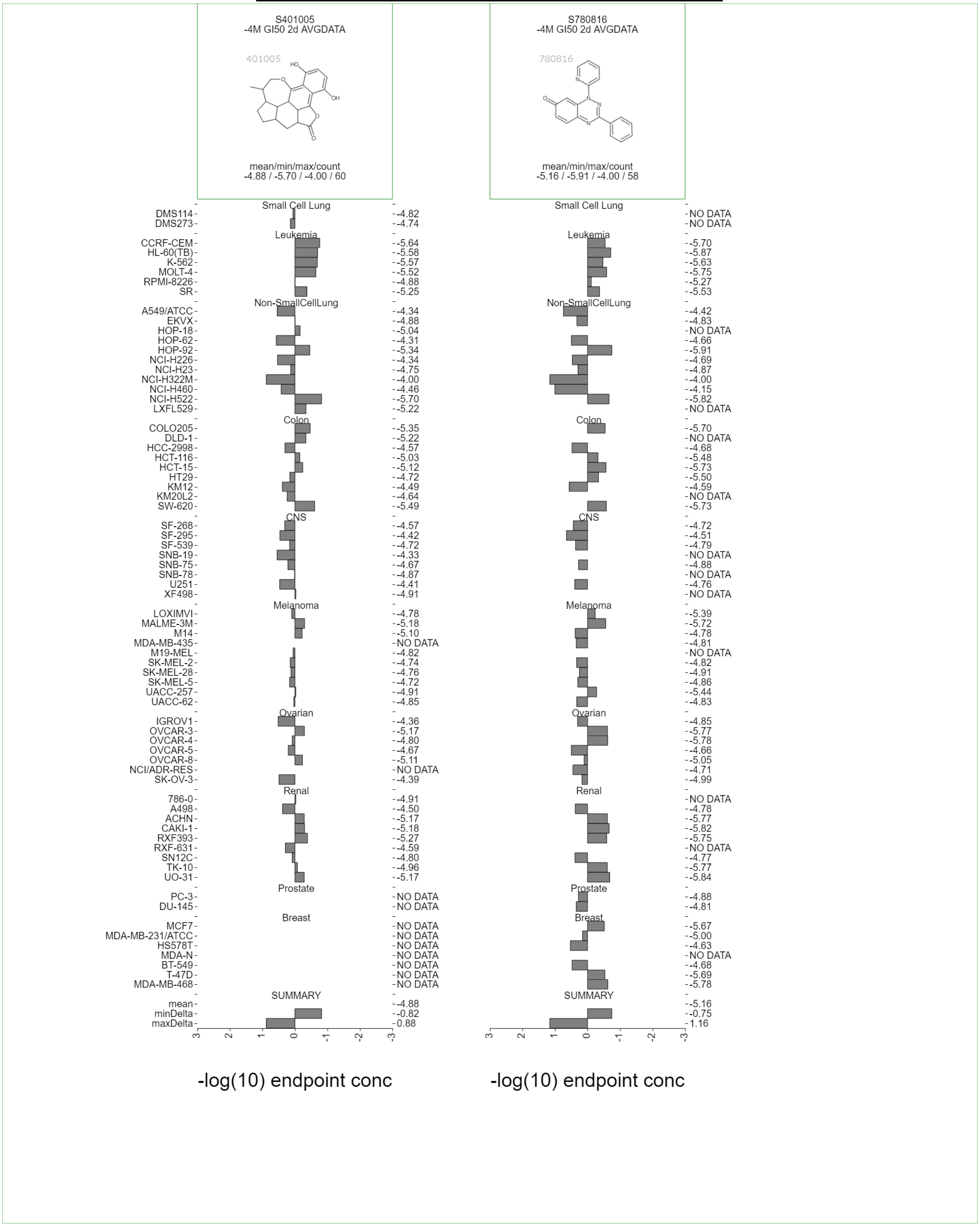

Figure S9

COMPARE Analysis of **5a** (NSC768093) to **5c** (NSC780816)

---SEED---

NSC:S768093 Endpoint:GI50 ExpId:AVGDATA logHiConc:-4.0

Id: 253700 Count Experiments: 2

---TARGET---

NSC:S780816 Endpoint:GI50 ExpId:AVGDATA logHiConc:-4.0

Id: 132828 Count Experiments: 1

Correlation: 0.78

JobId: 4300782396264764936

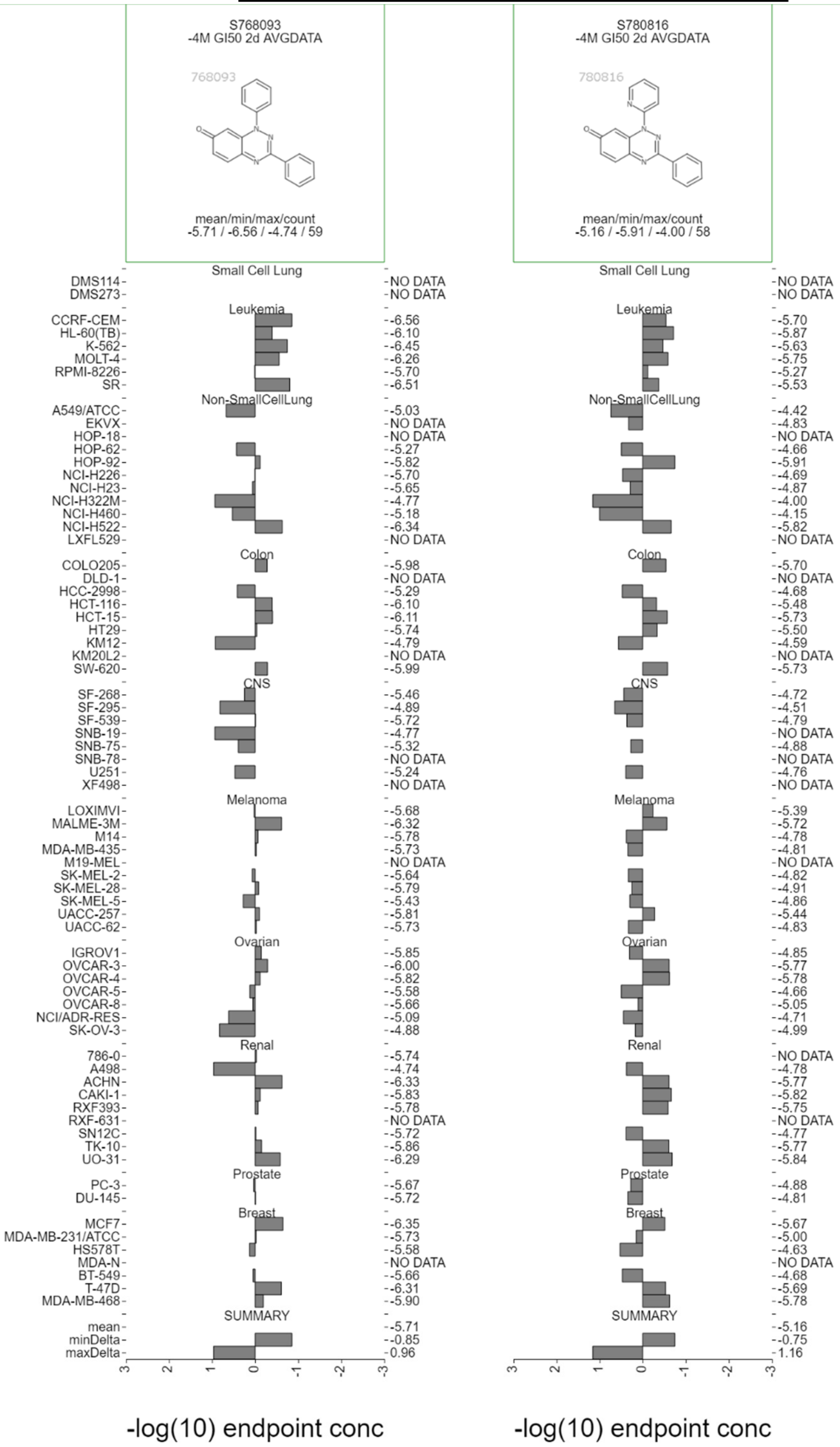

Figure S10

COMPARE Analysis of **5d** (NSC780815) to Pleurotin (NSC401005)

---SEED---
NSC:S780815 Endpoint:GI50 ExpId:AVGDATA logHiConc:-4.0
Id: 12861 Count Experiments: 1
---TARGET---
NSC:S401005 Endpoint:GI50 ExpId:AVGDATA logHiConc:-4.0
Id: 243481 Count Experiments: 2

Correlation: 0.73
JobId: 8698170618292679257

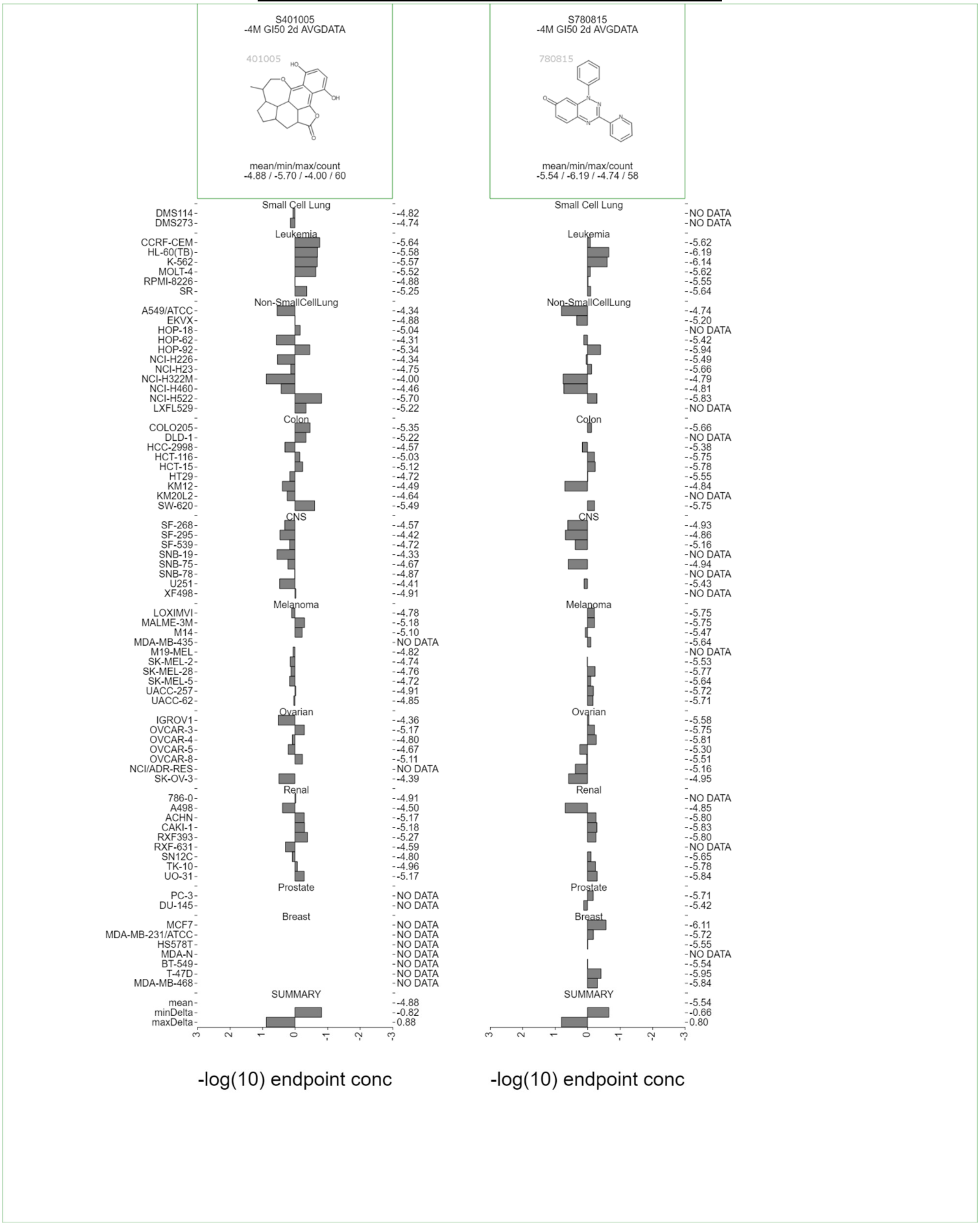

Figure S11

COMPARE Analysis of **5a** (NSC768093) to **5d** (NSC780815)

---SEED---
NSC:S768093 Endpoint:GI50 ExpId:AVGDATA logHiConc:-4.0
Id: 253700 Count Experiments: 2
---TARGET---
NSC:S780815 Endpoint:GI50 ExpId:AVGDATA logHiConc:-4.0
Id: 12861 Count Experiments: 1

Correlation: 0.83
JobId: 7840953164841129662

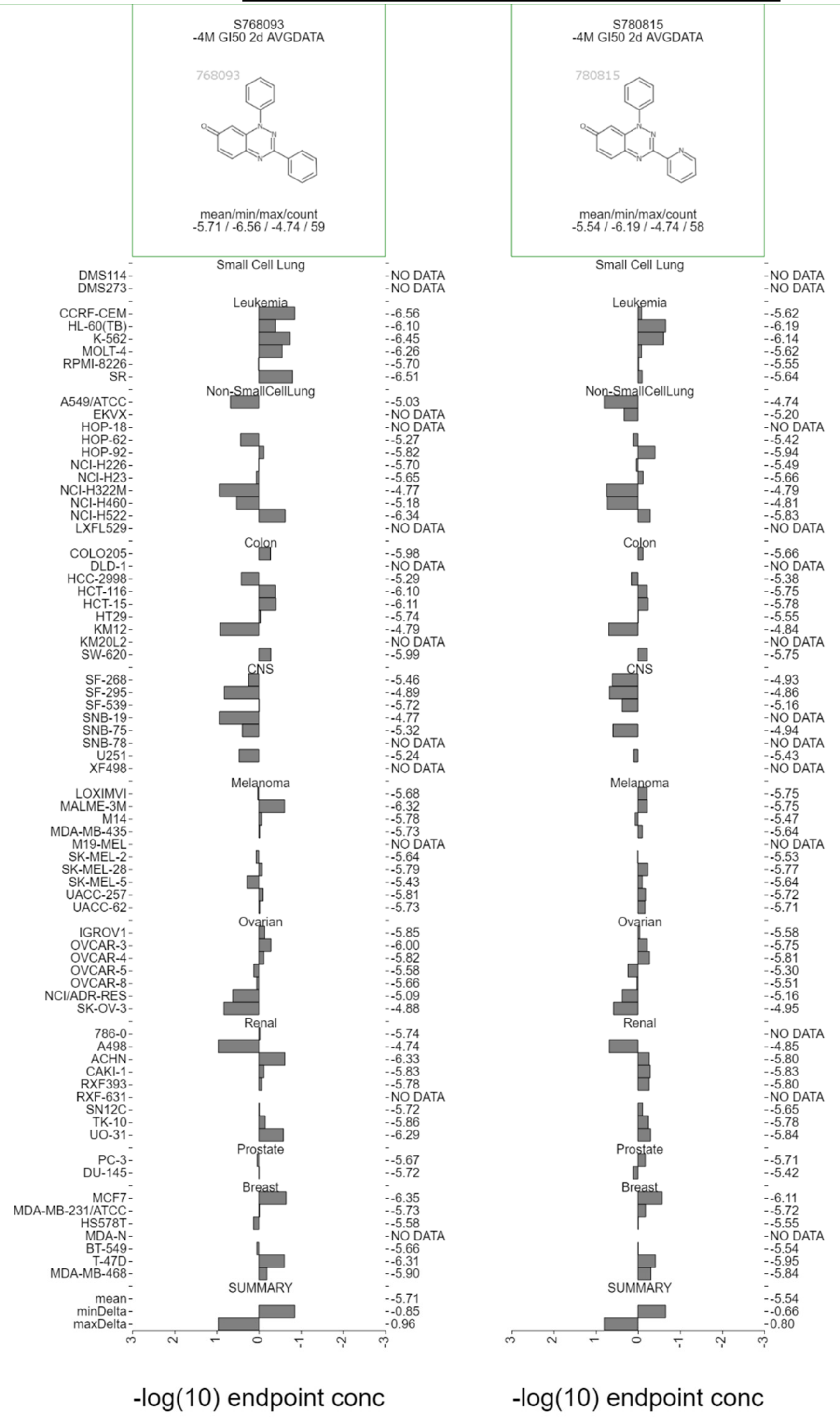

Figure S12

COMPARE Analysis of DPIQ (NSC753790) to **7** (NSC643735)

---SEED---
NSC:S753790 Endpoint:GI50 ExpId:AVGDATA logHiConc:-4.0
Id: 373263 Count Experiments: 2
---TARGET---
NSC:S643735 Endpoint:GI50 ExpId:AVGDATA logHiConc:-4.0
Id: 243352 Count Experiments: 6

Correlation: 0.74
JobId: 1612004397578779132

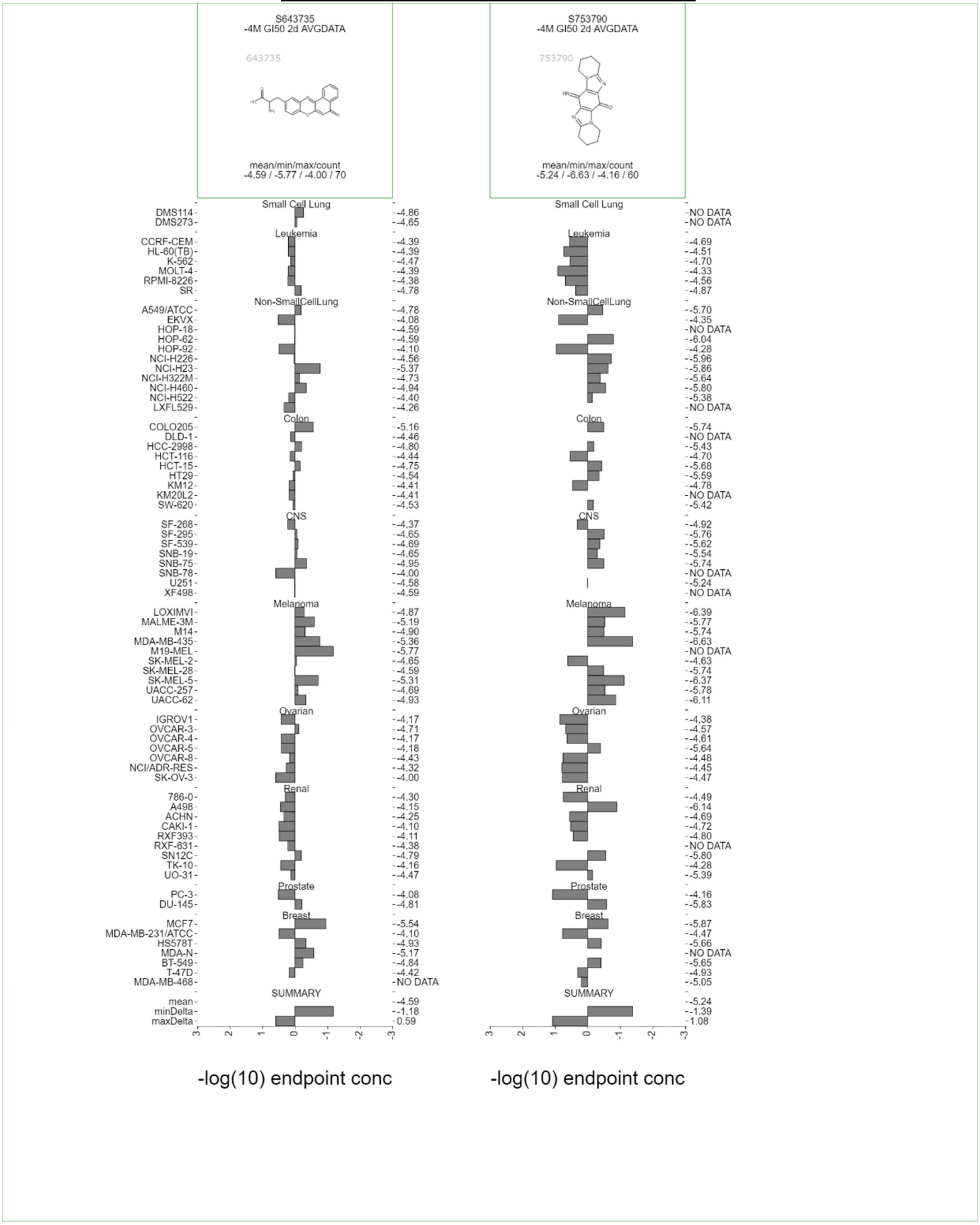

Figure S13

COMPARE Analysis of **7** (NSC643735) to NQO1

---SEED---
NSC:S643735 Endpoint:GI50 ExpId:AVGDATA logHiConc:-4.0
Id: 243352 Count Experiments: 6
---TARGET---
MoltId:MT22 Genecard:NQO1 TargetSet:MOLTID_MT_SERIES

Correlation: 0.38
JobId: 9083362337997199411

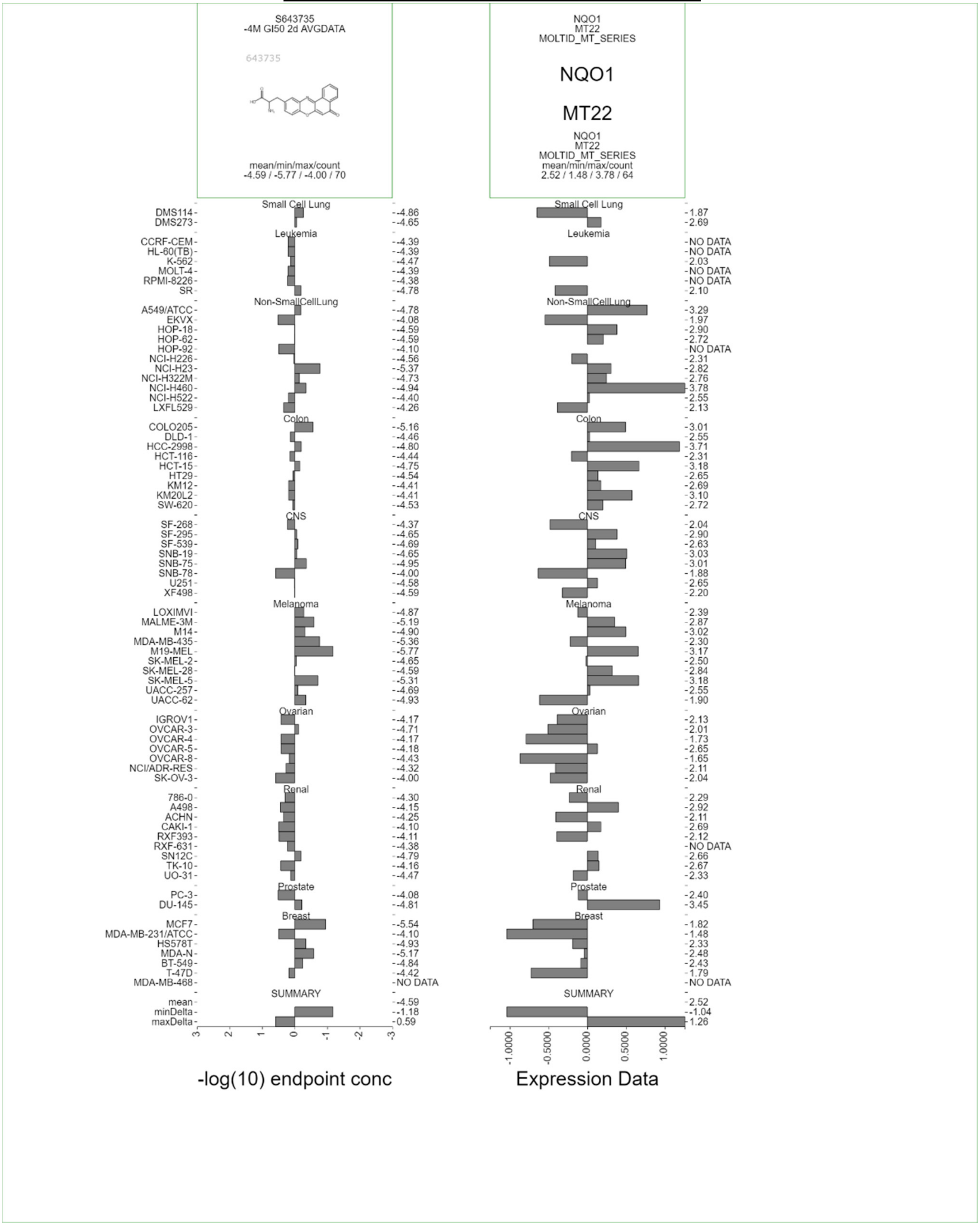

Figure S14

COMPARE Analysis of DPIQ (NSC753790) to **8a** (NSC795222)

---SEED---
NSC:S753790 Endpoint:GI50 ExpId:AVGDATA logHiConc:-4.0
Id: 373263 Count Experiments: 2
---TARGET---
NSC:S795222 Endpoint:GI50 ExpId:AVGDATA logHiConc:-4.0
Id: 13092 Count Experiments: 2

Correlation: 0.81
JobId: 850257062471574592

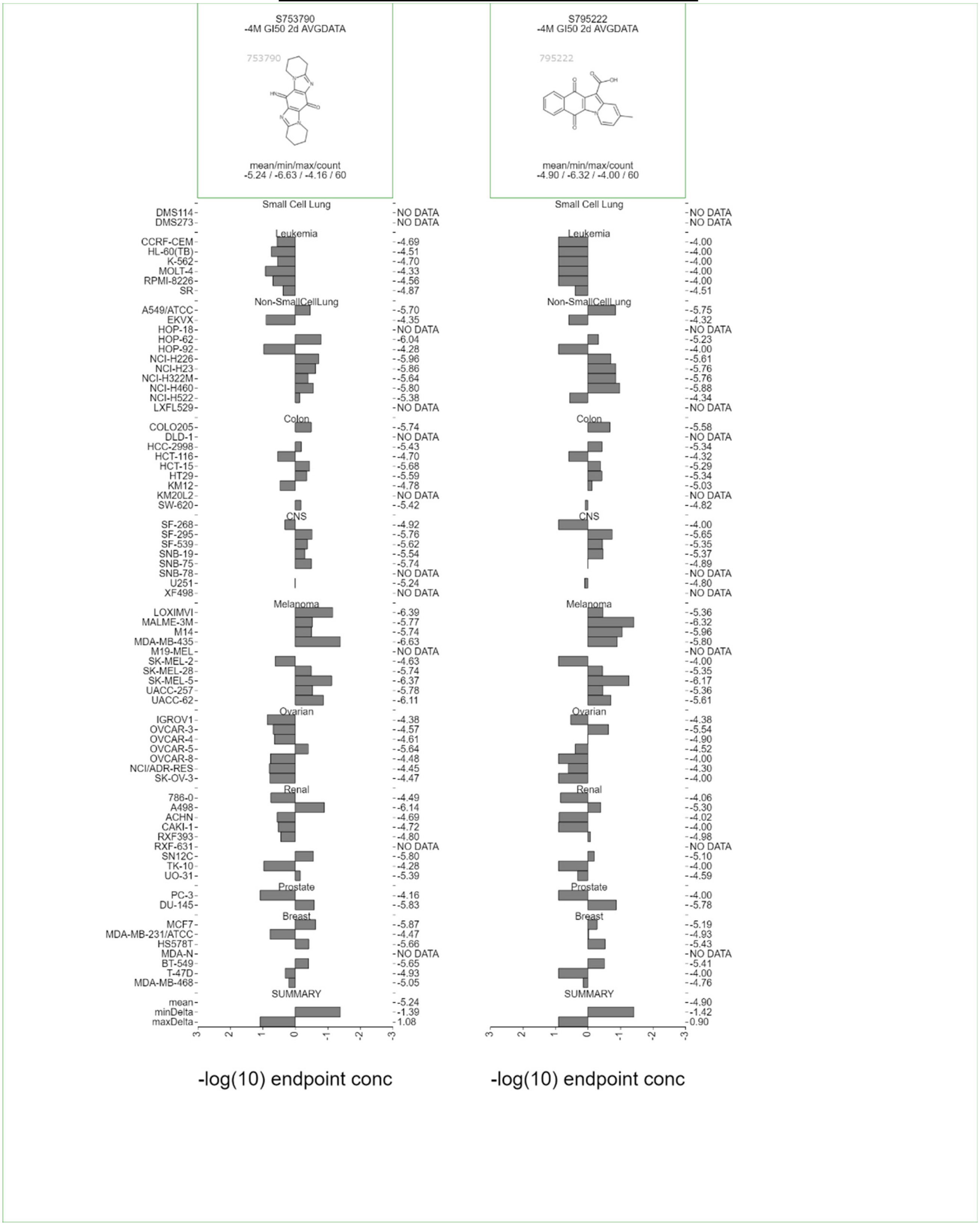

Figure S15

COMPARE Analysis of **8a** (NSC795222) to NQO1

---SEED---
NSC:S795222 Endpoint:GI50 ExpId:AVGDATA logHiConc:-4.0
Id: 13092 Count Experiments: 2
---TARGET---
MoltId:MT22 Genecard:NQO1 TargetSet:MOLTID_MT_SERIES

Correlation: 0.53
JobId: 5161239735822820917

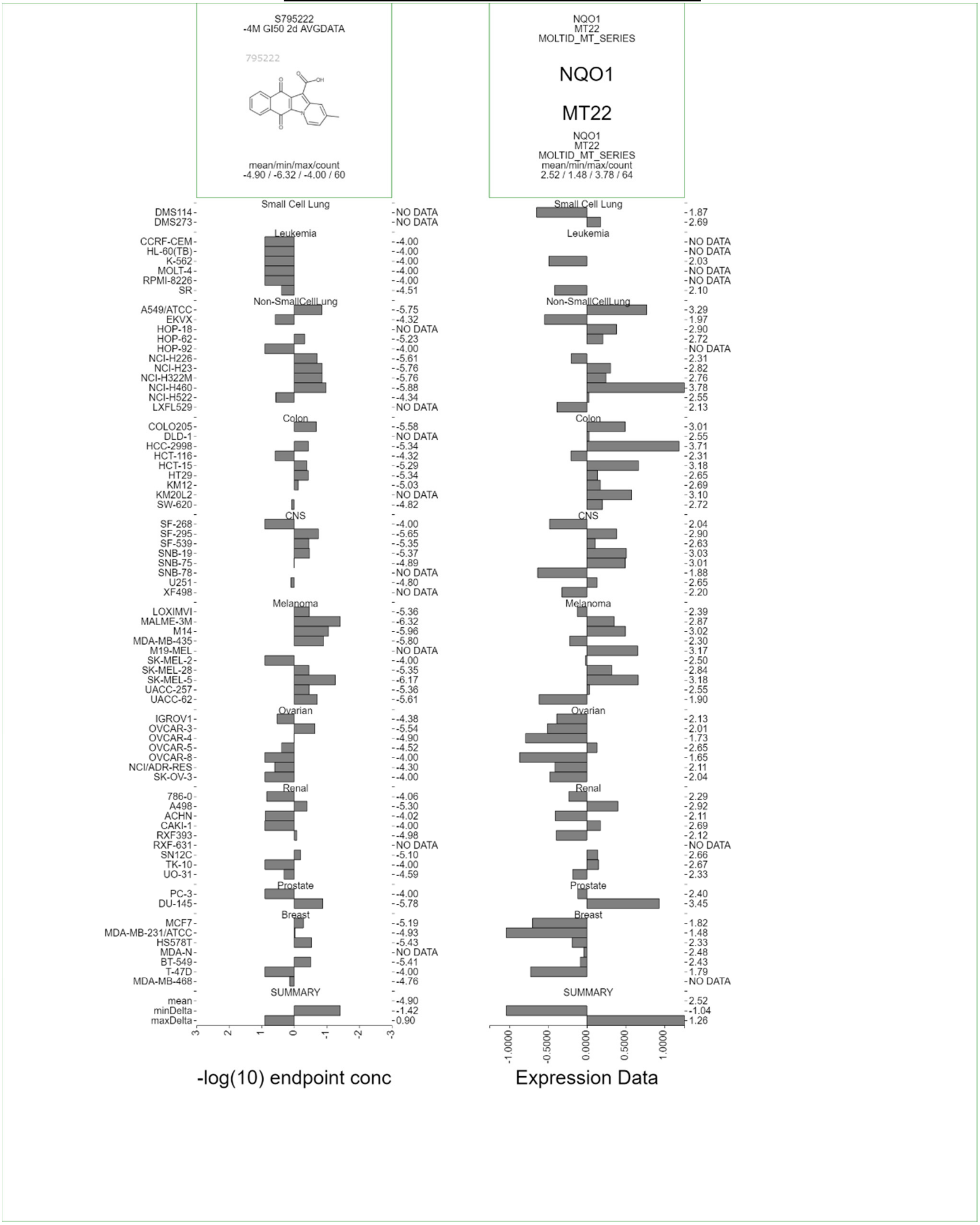

Figure S16

COMPARE Analysis of DPIQ (NSC753790) to **9a** (NSC291094)

---SEED---
NSC:S753790 Endpoint:GI50 ExpId:AVGDATA logHiConc:-4.0
Id: 373263 Count Experiments: 2
---TARGET---
NSC:S291094 Endpoint:GI50 ExpId:AVGDATA logHiConc:-4.0
Id: 193856 Count Experiments: 1

Correlation: 0.80
JobId: 5900945876456588451

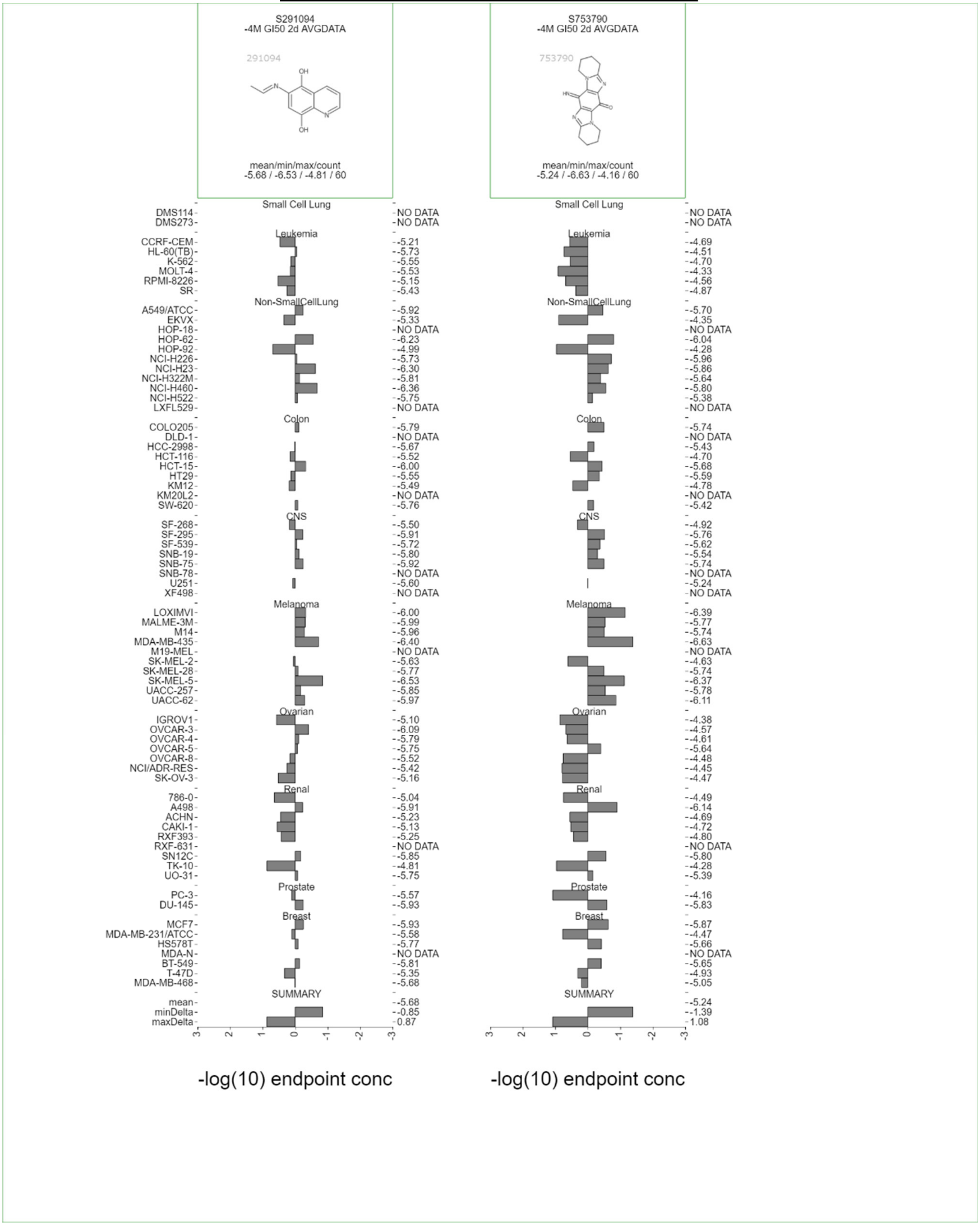

Figure S17

COMPARE Analysis of **9a** (NSC291094) to NQO1

---SEED---

NSC:S291094 Endpoint:GI50 ExpId:AVGDATA logHiConc:-4.0

Id: 193856 Count Experiments: 1

---TARGET---

MoltId:MT22 Genecard:NQO1 TargetSet:MOLTID_MT_SERIES

Correlation: 0.42

JobId: 3132994177876046533

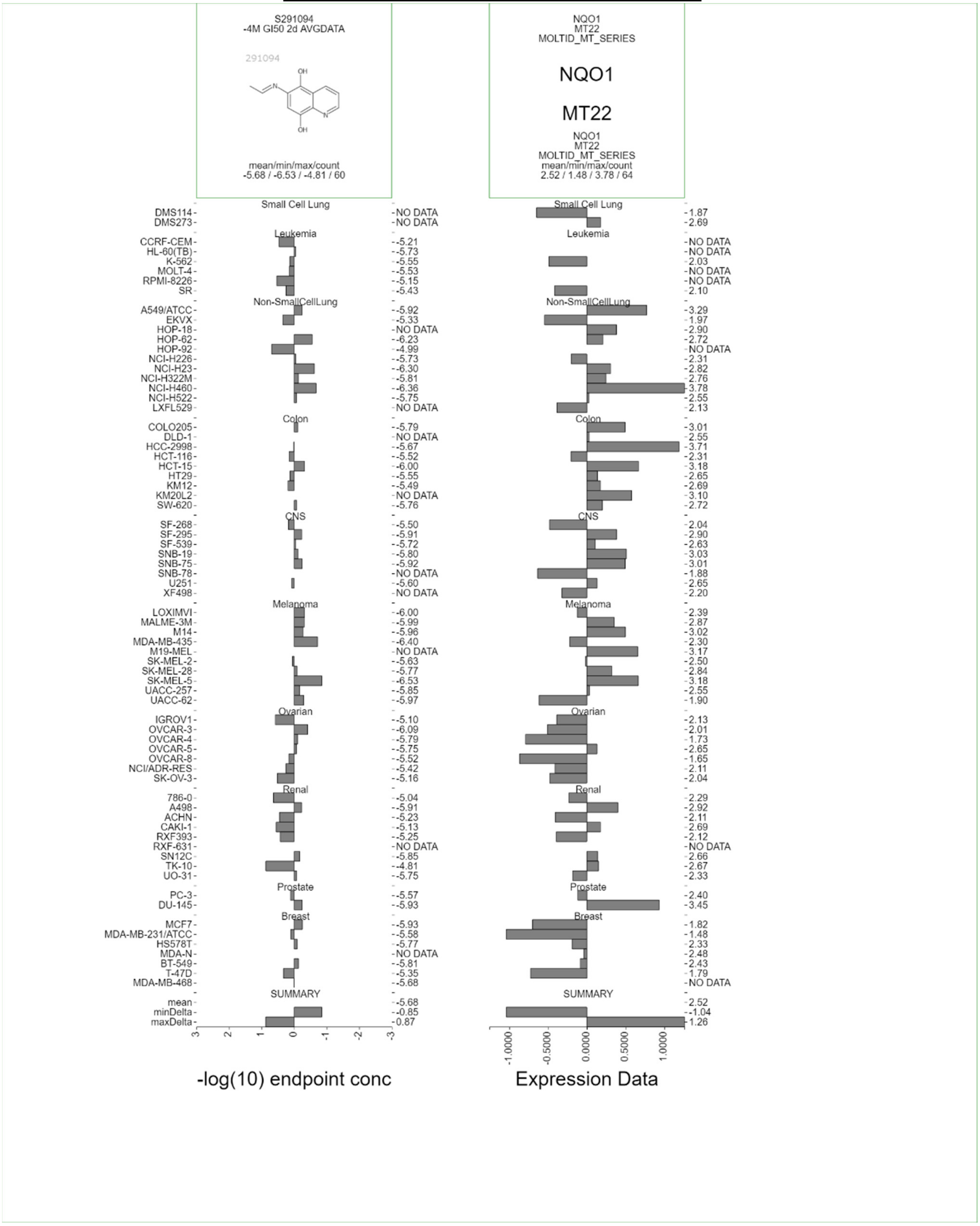

Figure S18

COMPARE Analysis of DPIQ (NSC753790) to **10a** (NSC798710)

---SEED---
NSC:S753790 Endpoint:GI50 ExpId:AVGDATA logHiConc:-4.0
Id: 373263 Count Experiments: 2
---TARGET---
NSC:S798710 Endpoint:GI50 ExpId:AVGDATA logHiConc:-4.0
Id: 72893 Count Experiments: 1

Correlation: 0.75
JobId: 3400101552620000377

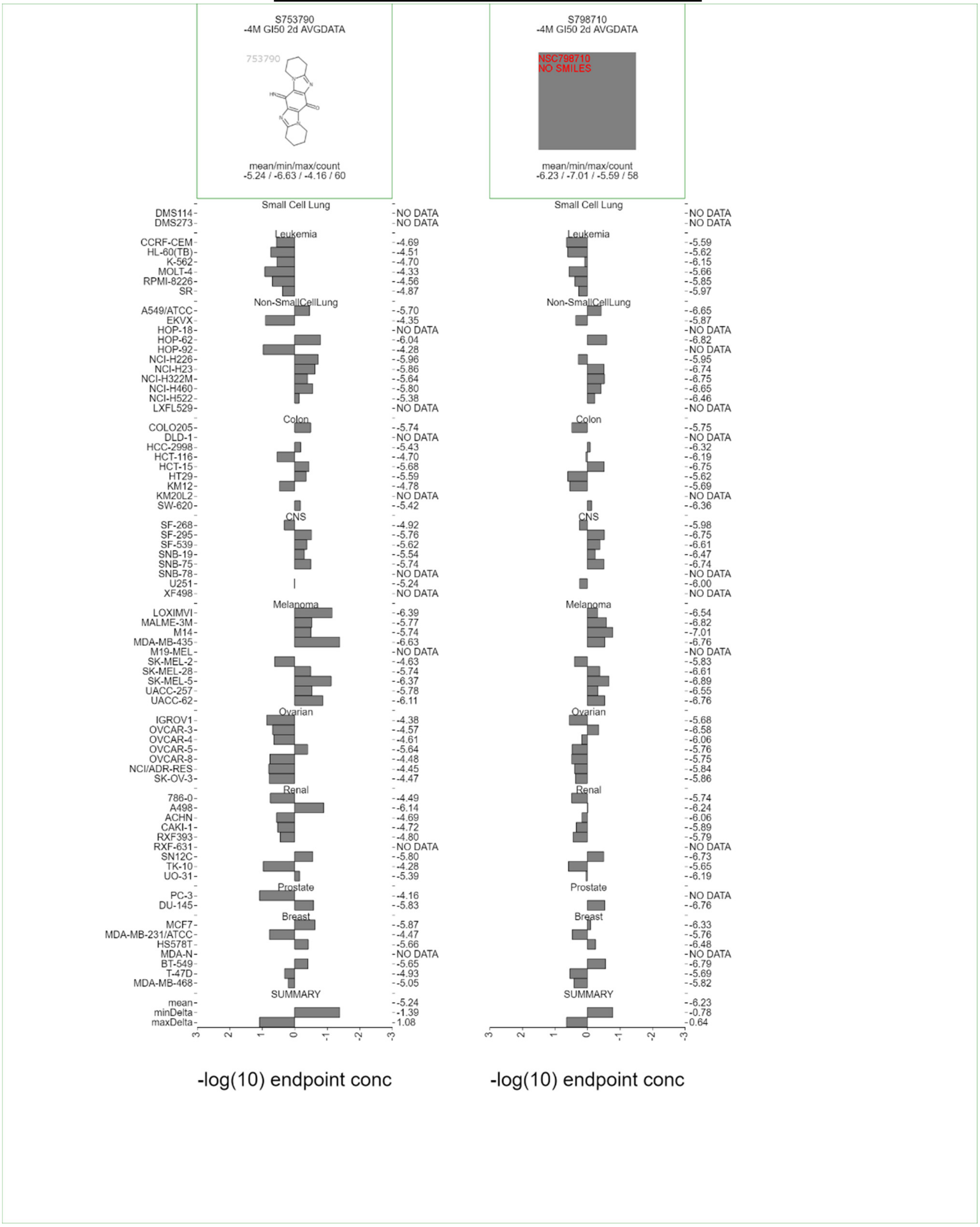

Figure S19

COMPARE Analysis of **10a** (NSC798710) to NQO1

---SEED---

NSC:S798710 Endpoint:GI50 ExpId:AVGDATA logHiConc:-4.0

Id: 72893 Count Experiments: 1

---TARGET---

MoltId:MT22 Genecard:NQO1 TargetSet:MOLTID_MT_SERIES

Correlation: 0.48

JobId: 7109766531662089207

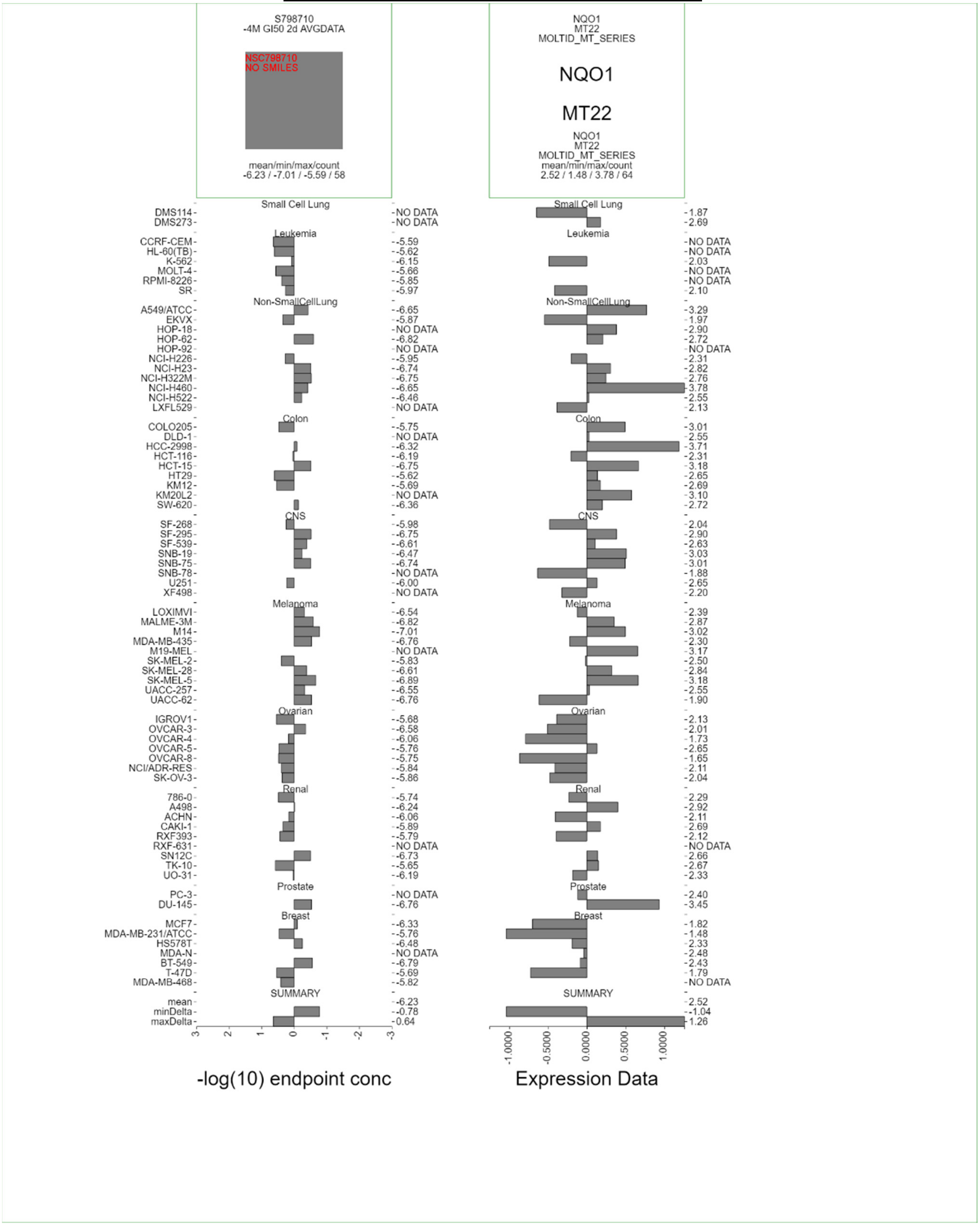

Figure S20

COMPARE Analysis of DPIQ (NSC753790) to **10b** (NSC798709)

---SEED---
NSC:S753790 Endpoint:GI50 ExpId:AVGDATA logHiConc:-4.0
Id: 373263 Count Experiments: 2
---TARGET---
NSC:S798709 Endpoint:GI50 ExpId:AVGDATA logHiConc:-4.0
Id: 193914 Count Experiments: 1

Correlation: 0.72
JobId: 3502385323016355770

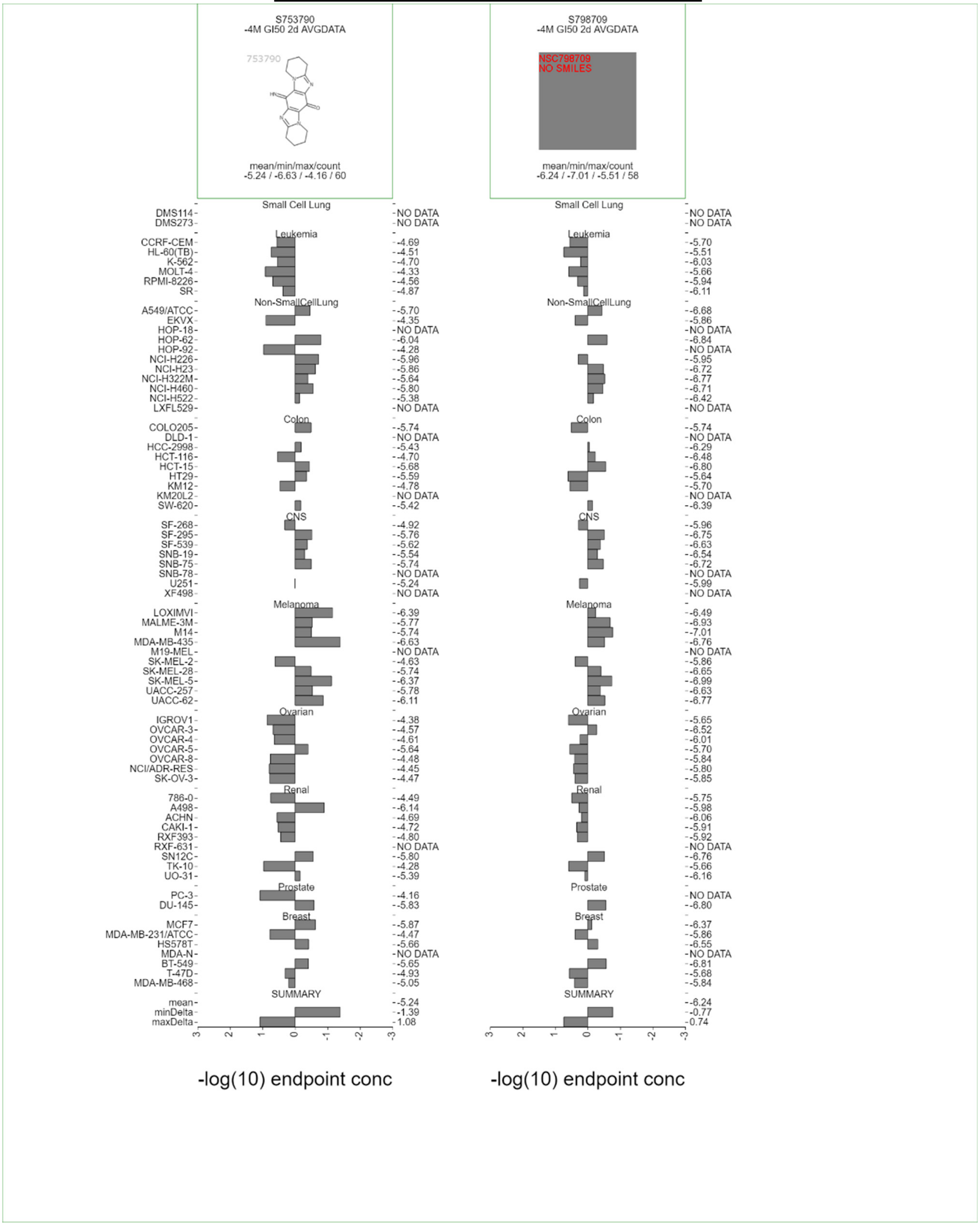

Figure S21

COMPARE Analysis of **10b** (NSC798709) to NQO1

---SEED---
NSC:S798709 Endpoint:GI50 ExpId:AVGDATA logHiConc:-4.0
Id: 193914 Count Experiments: 1
---TARGET---
MoltId:MT22 Genecard:NQO1 TargetSet:MOLTID_MT_SERIES

Correlation: 0.47
JobId: 5437088846418911921

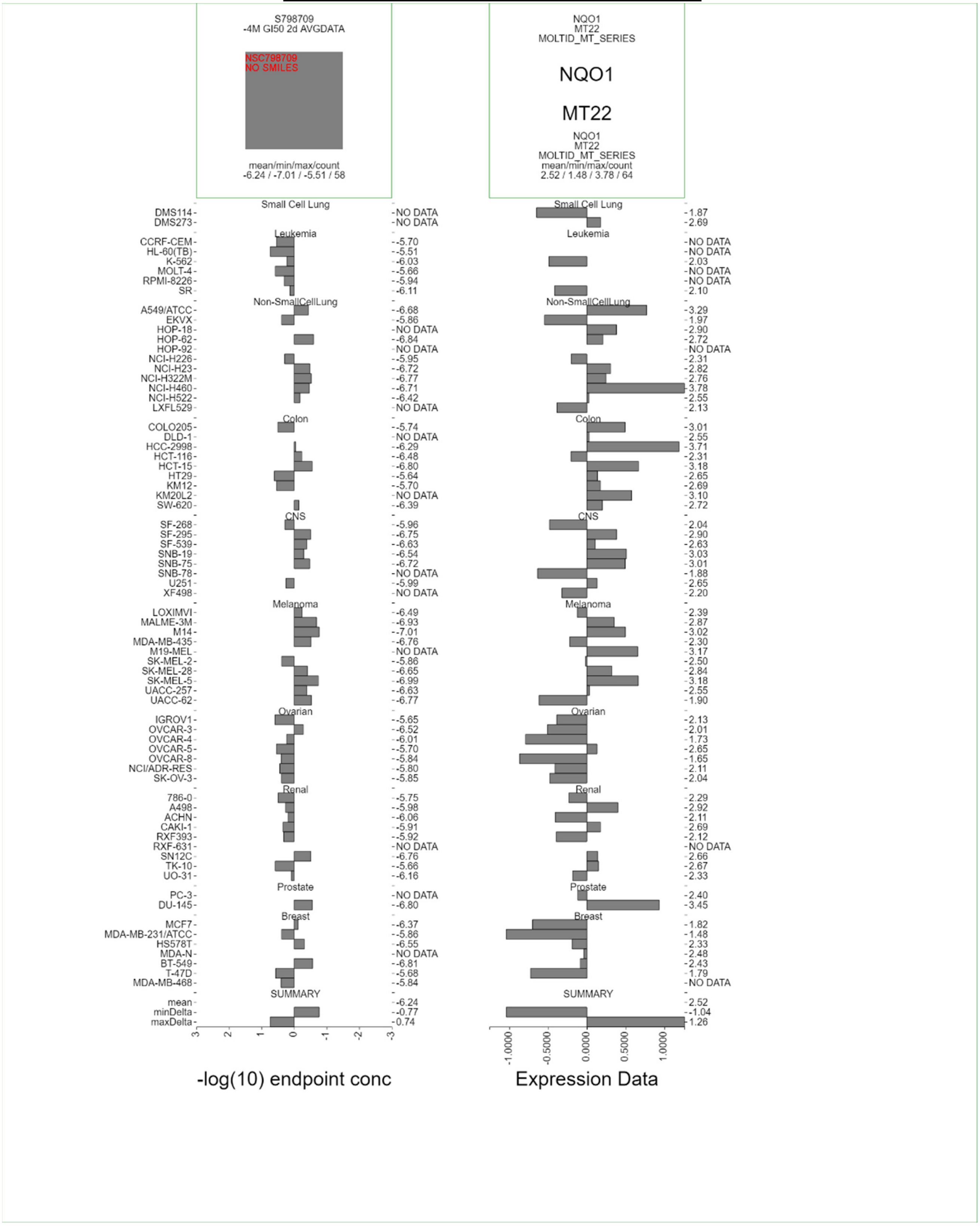

Figure S22

COMPARE Analysis of DPIQ (NSC753790) to **11b** (NSC750104)

---SEED---
NSC:S753790 Endpoint:GI50 ExpId:AVGDATA logHiConc:-4.0
Id: 373263 Count Experiments: 2
---TARGET---
NSC:S750104 Endpoint:GI50 ExpId:AVGDATA logHiConc:-4.0
Id: 192468 Count Experiments: 2

Correlation: 0.73
JobId: 3236006900684295182

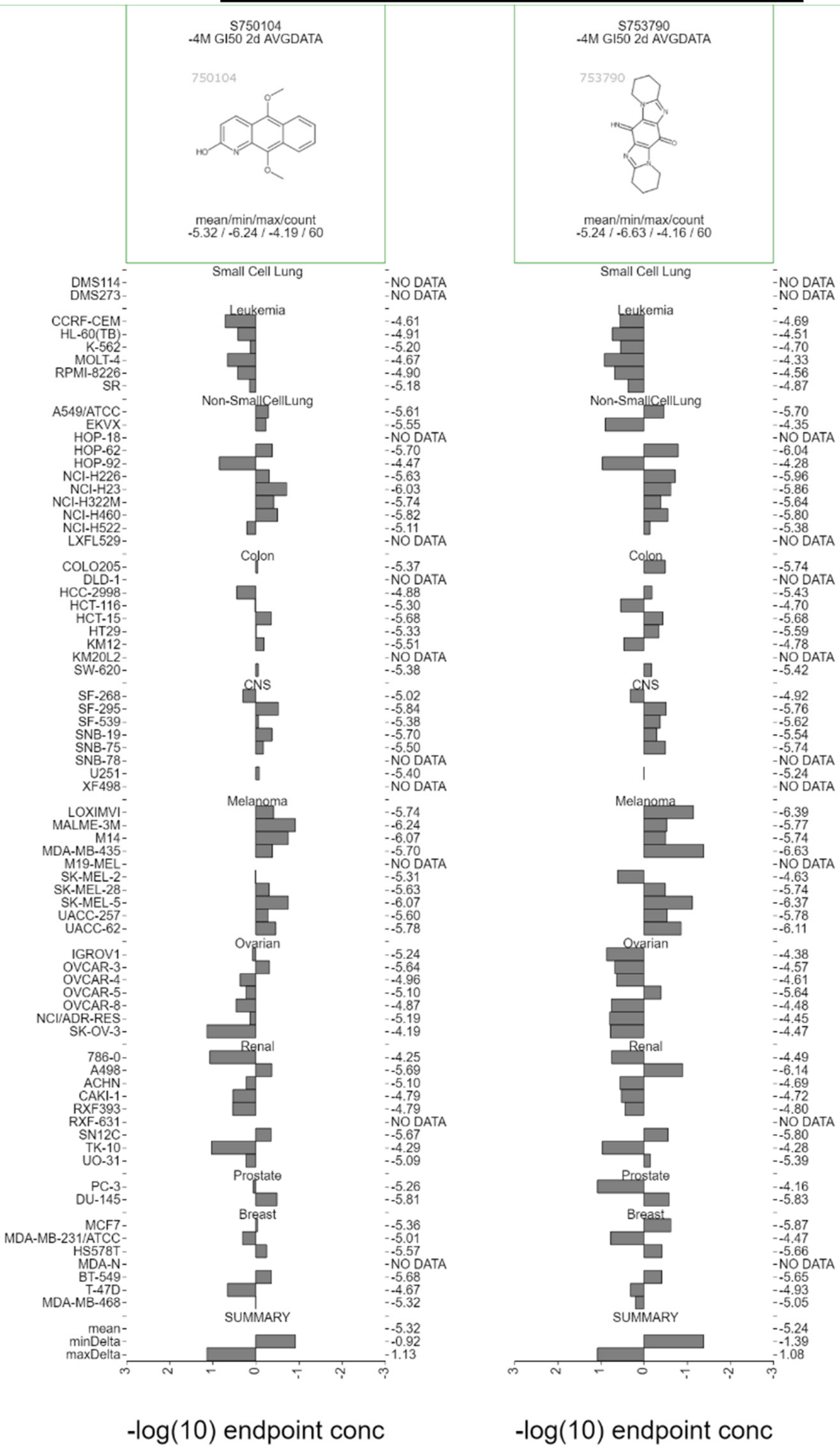

Figure S23

COMPARE Analysis of **11b** (NSC750104) to NQO1

---SEED---

NSC:S750104 Endpoint:GI50 ExpId:AVGDATA logHiConc:-4.0

Id: 192468 Count Experiments: 2

---TARGET---

MoltId:MT22 Genecard:NQO1 TargetSet:MOLTID_MT_SERIES

Correlation: 0.44

JobId: 6783138607820758427

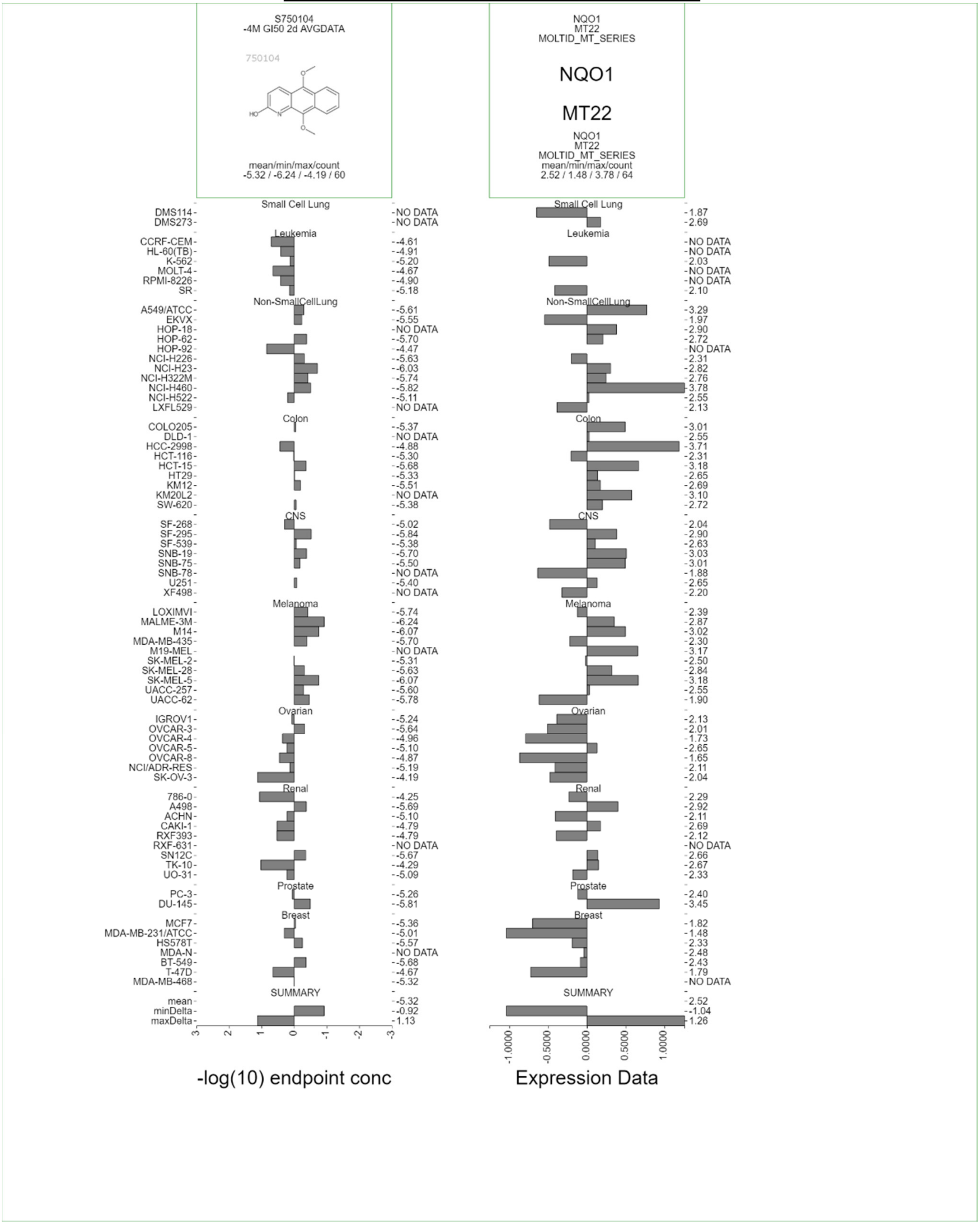

Figure S24

COMPARE Analysis of DPIQ (NSC753790) to **12** (NSC647943)

---SEED---
NSC:S753790 Endpoint:GI50 ExpId:AVGDATA logHiConc:-4.0
Id: 373263 Count Experiments: 2
---TARGET---
NSC:S647943 Endpoint:GI50 ExpId:AVGDATA logHiConc:-4.0
Id: 3059 Count Experiments: 2

Correlation: 0.72
JobId: 7470274996145913057

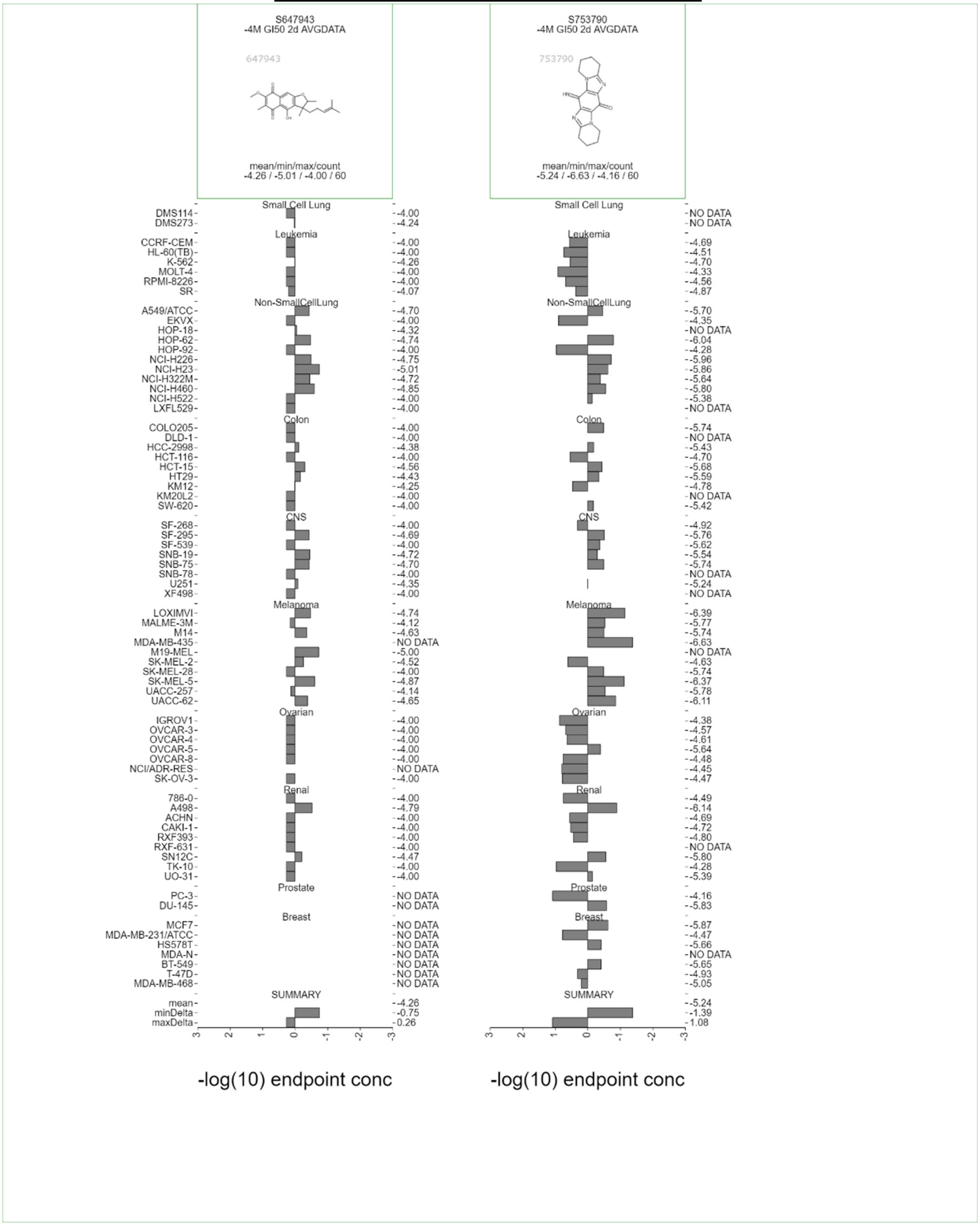

Figure S25

COMPARE Analysis of **12** (NSC647943) to NQO1

---SEED---
NSC:S647943 Endpoint:GI50 ExpId:AVGDATA logHiConc:-4.0
Id: 3059 Count Experiments: 2
---TARGET---
MoltId:MT22 Genecard:NQO1 TargetSet:MOLTID_MT_SERIES

Correlation: 0.55
JobId: 1070728578726149116

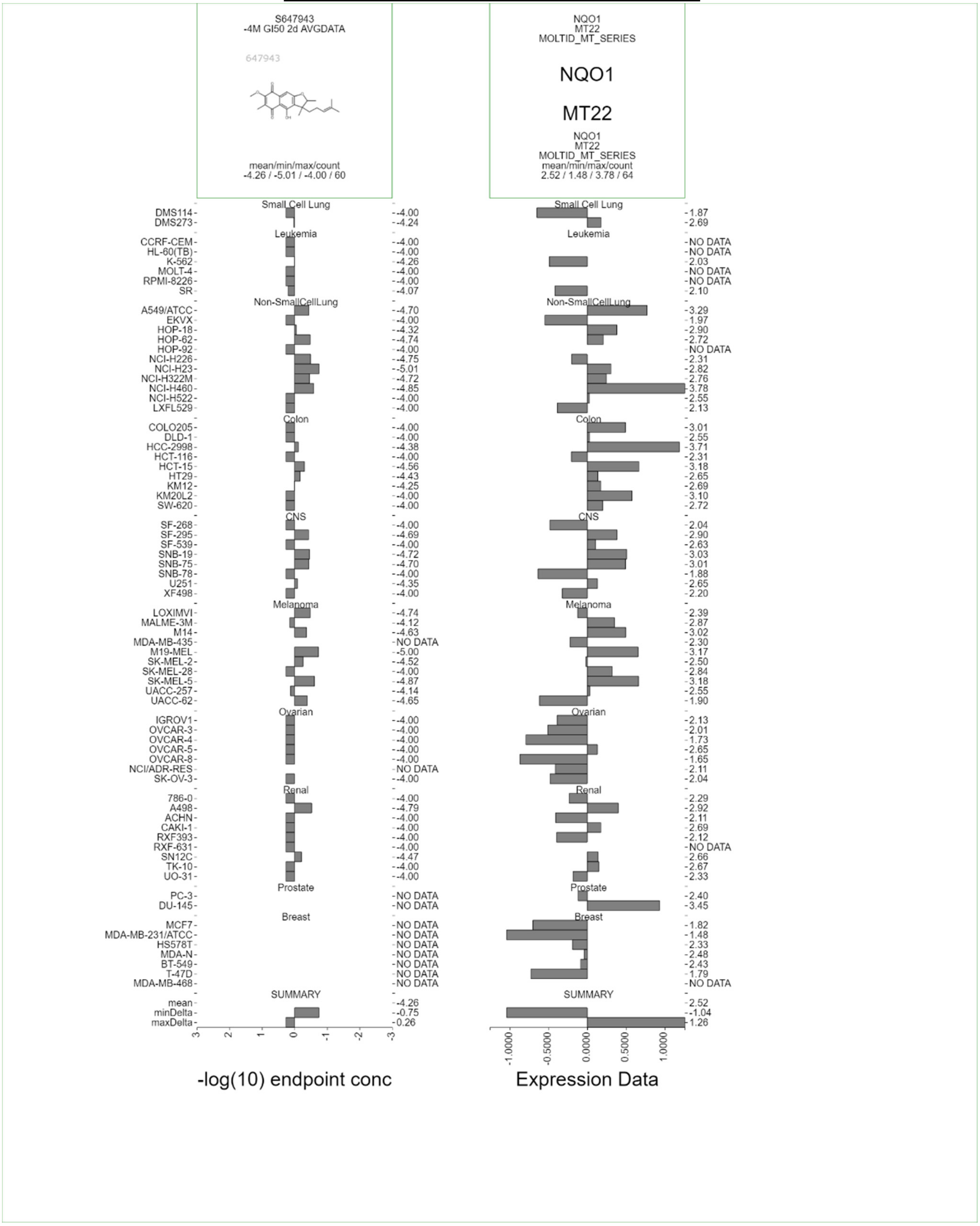

Figure S26

COMPARE Analysis of **5a** (NSC768093) to **13a** (NSC787555)

---SEED---
NSC:S768093 Endpoint:GI50 ExpId:AVGDATA logHiConc:-4.0
Id: 253700 Count Experiments: 2
---TARGET---
NSC:S787555 Endpoint:GI50 ExpId:AVGDATA logHiConc:-4.0
Id: 254082 Count Experiments: 3

Correlation: 0.85
JobId: 3773562751148899517

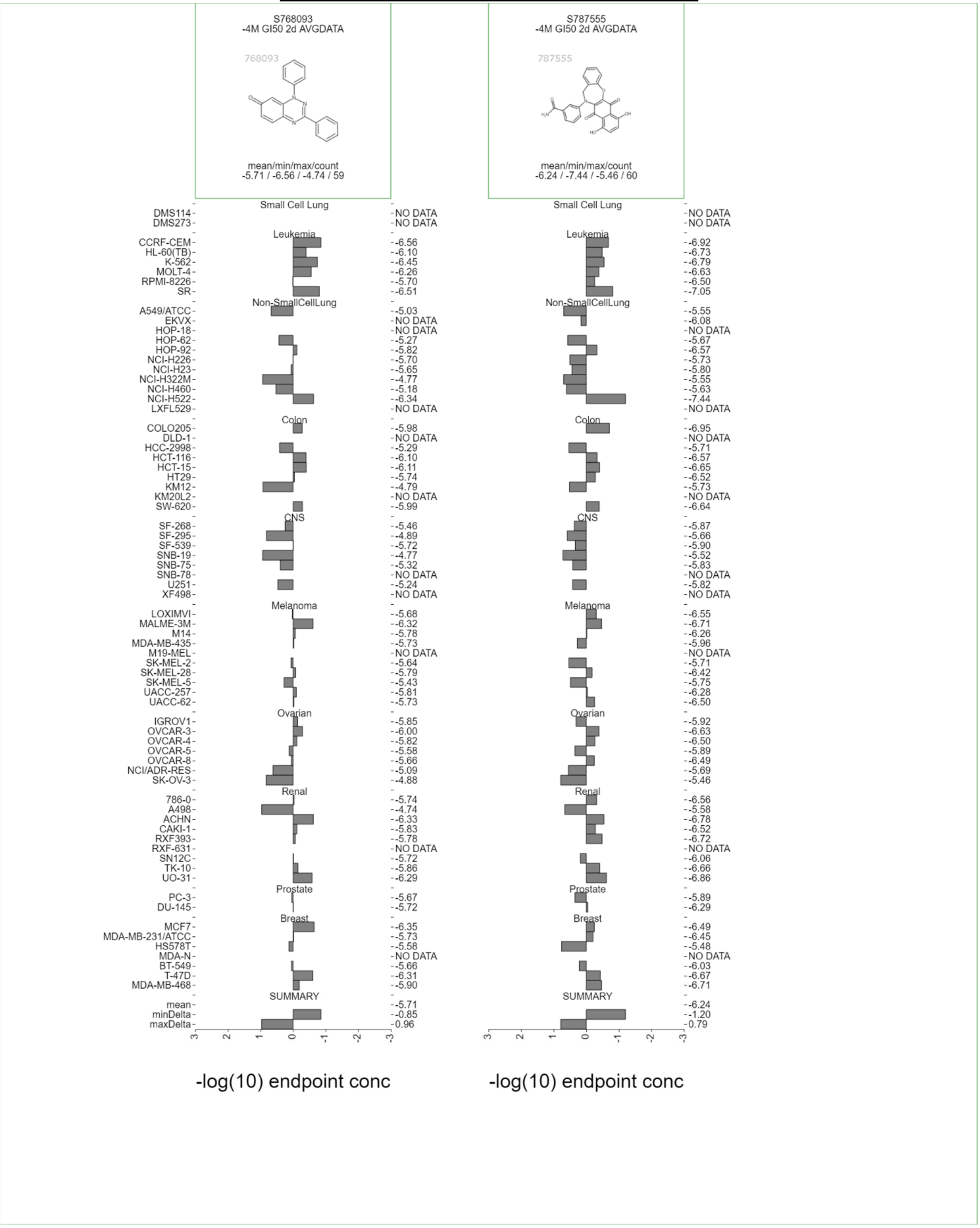

Figure S27

COMPARE Analysis of **13a** (NSC787555) to Pleurotin (NSC401005)

---SEED---
NSC:S787555 Endpoint:GI50 ExpId:AVGDATA logHiConc:-4.0
Id: 12861 Count Experiments: 3
---TARGET---
NSC:S401005 Endpoint:GI50 ExpId:AVGDATA logHiConc:-4.0
Id: 243481 Count Experiments: 2

Correlation: 0.89
JobId: 1099128821080064092

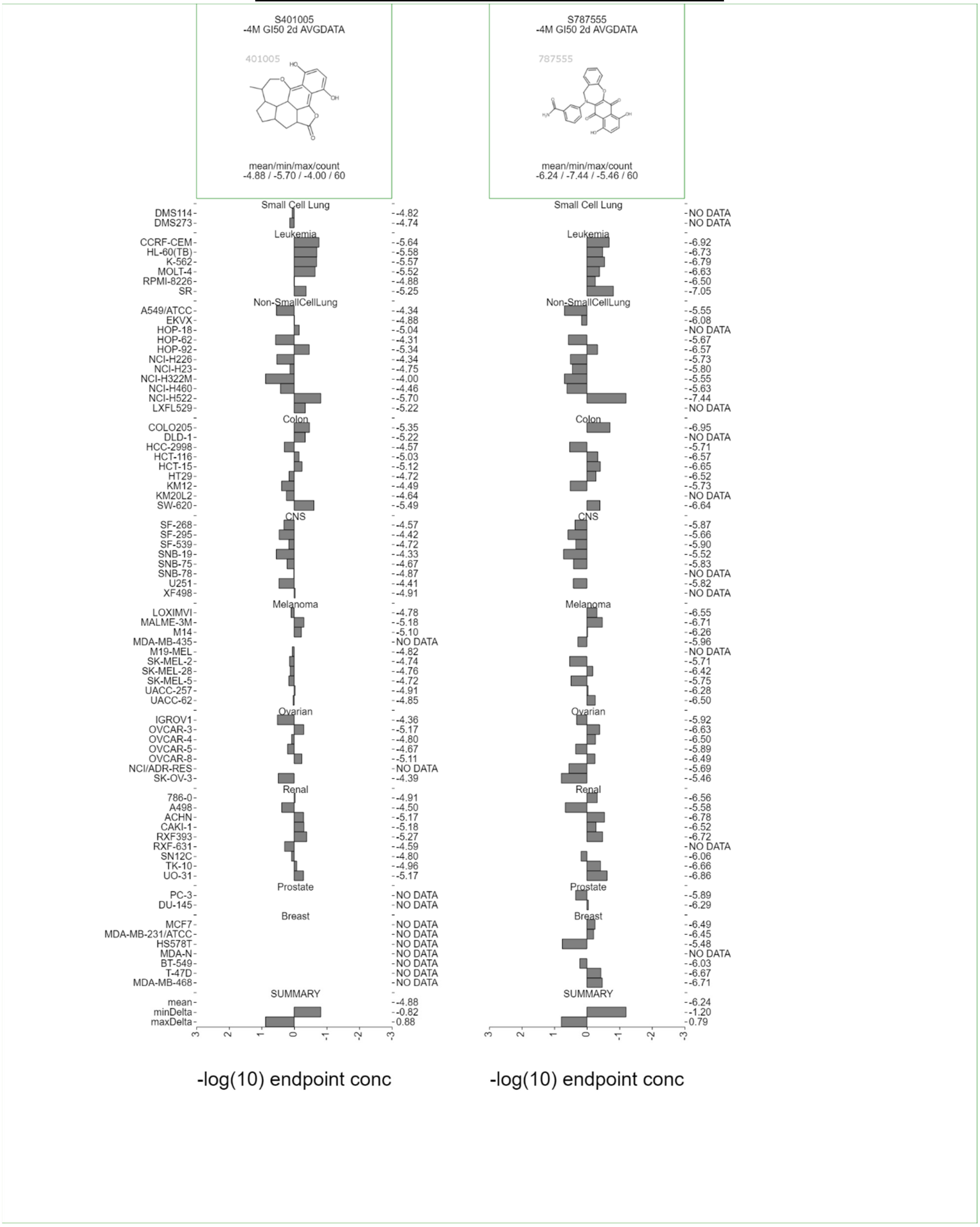

Figure S28

COMPARE Analysis of **5a** (NSC768093) to **13b** (NSC787553)

---SEED---
NSC:S768093 Endpoint:GI50 ExpId:AVGDATA logHiConc:-4.0
Id: 253700 Count Experiments: 2
---TARGET---
NSC:S787553 Endpoint:GI50 ExpId:AVGDATA logHiConc:-4.0
Id: 133007 Count Experiments: 2

Correlation: 0.90
JobId: 2819999011519452973

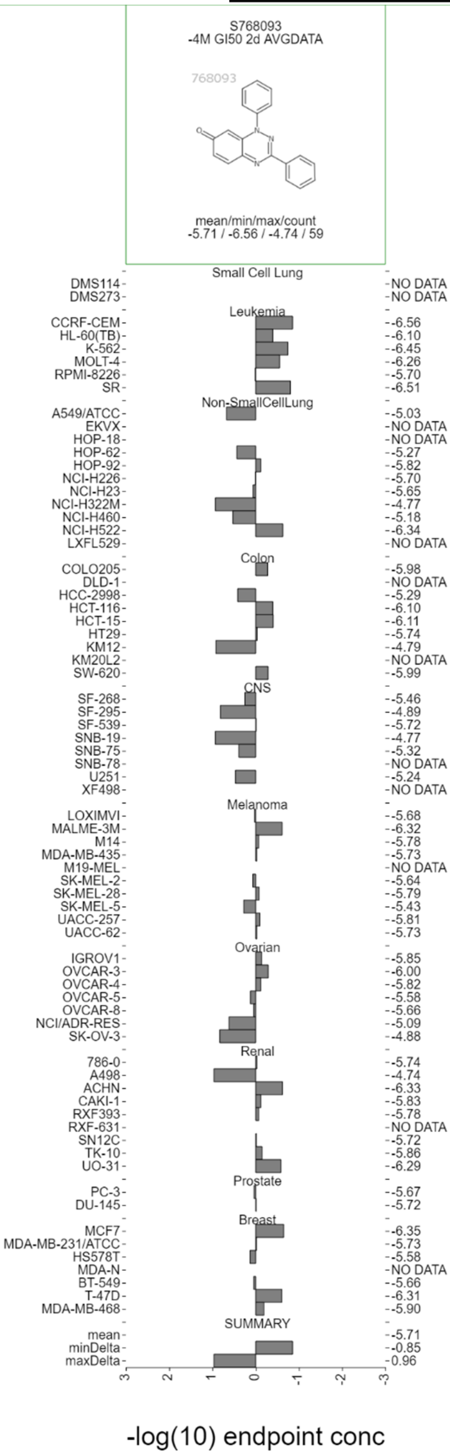

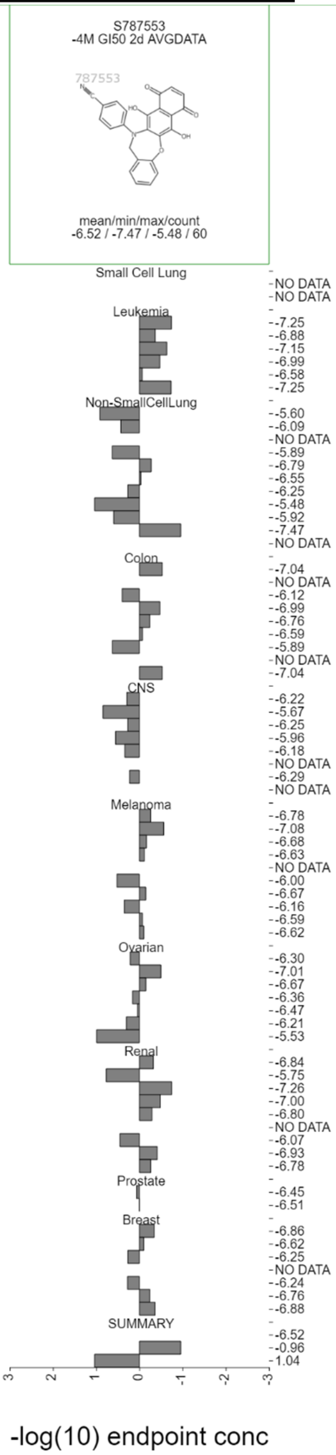

Figure S29

COMPARE Analysis of **13b** (NSC787553) to Pleurotin (NSC401005)

---SEED---
NSC:S787553 Endpoint:GI50 ExpId:AVGDATA logHiConc:-4.0
Id: 133007 Count Experiments: 2
---TARGET---
NSC:S401005 Endpoint:GI50 ExpId:AVGDATA logHiConc:-4.0
Id: 243481 Count Experiments: 2

Correlation: 0.86
JobId: 7810175962957311835

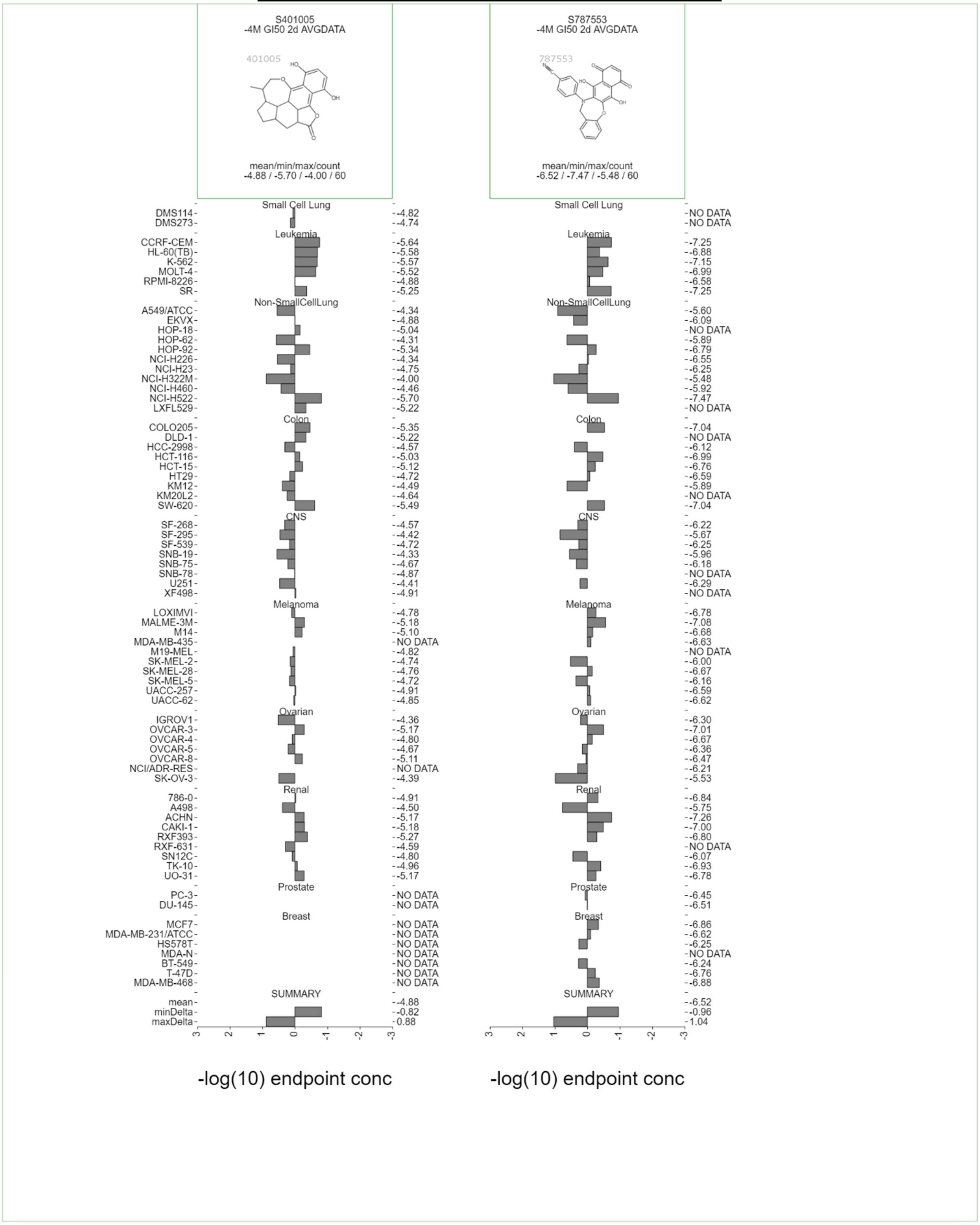

Figure S30

COMPARE Analysis of **5a** (NSC768093) to **13c** (NSC787554)

---SEED---
NSC:S768093 Endpoint:GI50 ExpId:AVGDATA logHiConc:-4.0
Id: 253700 Count Experiments: 2
---TARGET---
NSC:S787554 Endpoint:GI50 ExpId:AVGDATA logHiConc:-4.0
Id: 72712 Count Experiments: 2

Correlation: 0.88
JobId: 152861254556724592

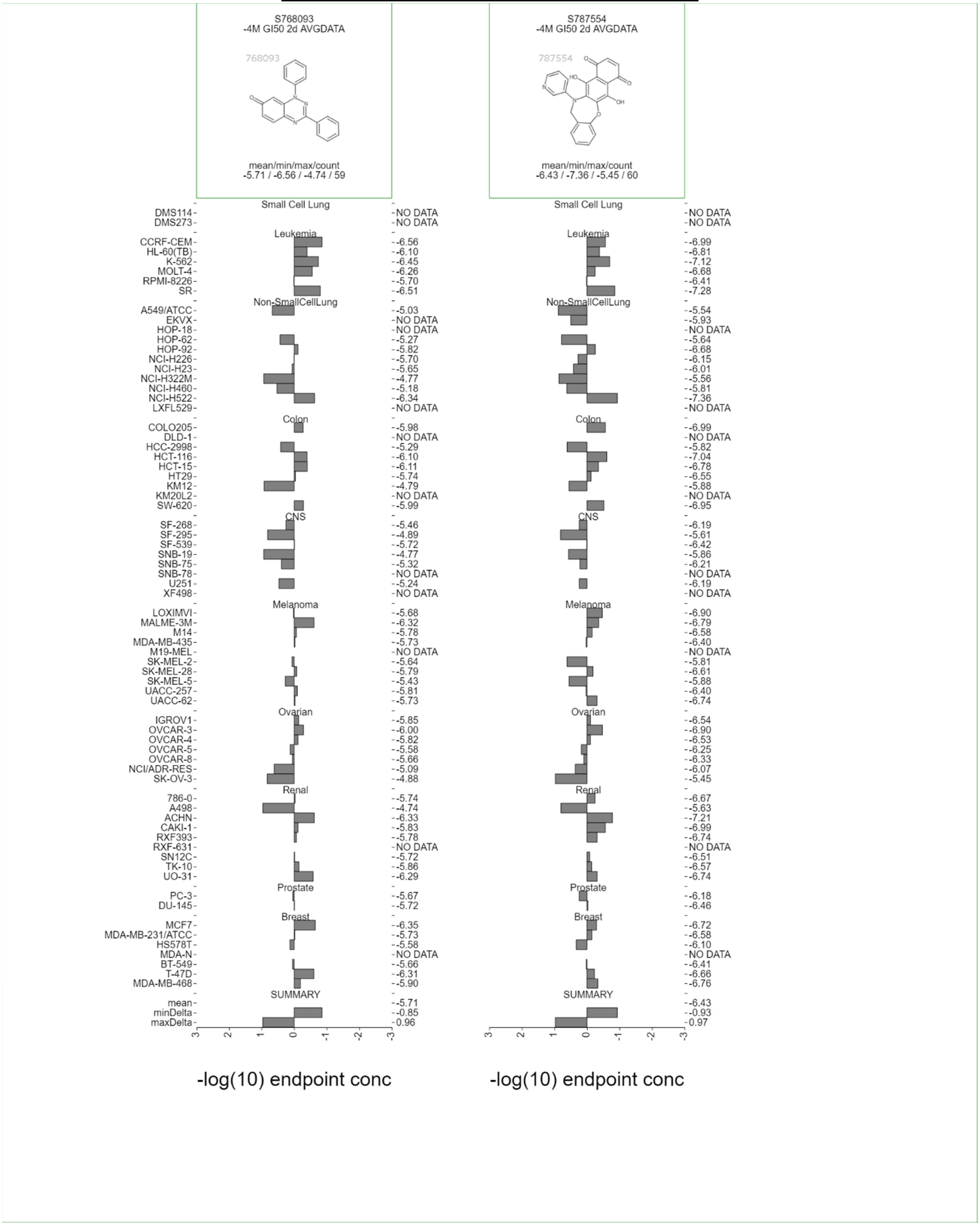

Figure S31

COMPARE Analysis of **13c** (NSC787554) to Pleurotin (NSC401005)

---SEED---
NSC:S787554 Endpoint:GI50 ExpId:AVGDATA logHiConc:-4.0
Id: 72712 Count Experiments: 2
---TARGET---
NSC:S401005 Endpoint:GI50 ExpId:AVGDATA logHiConc:-4.0
Id: 243481 Count Experiments: 2

Correlation: 0.82
JobId: 2239556794382988653

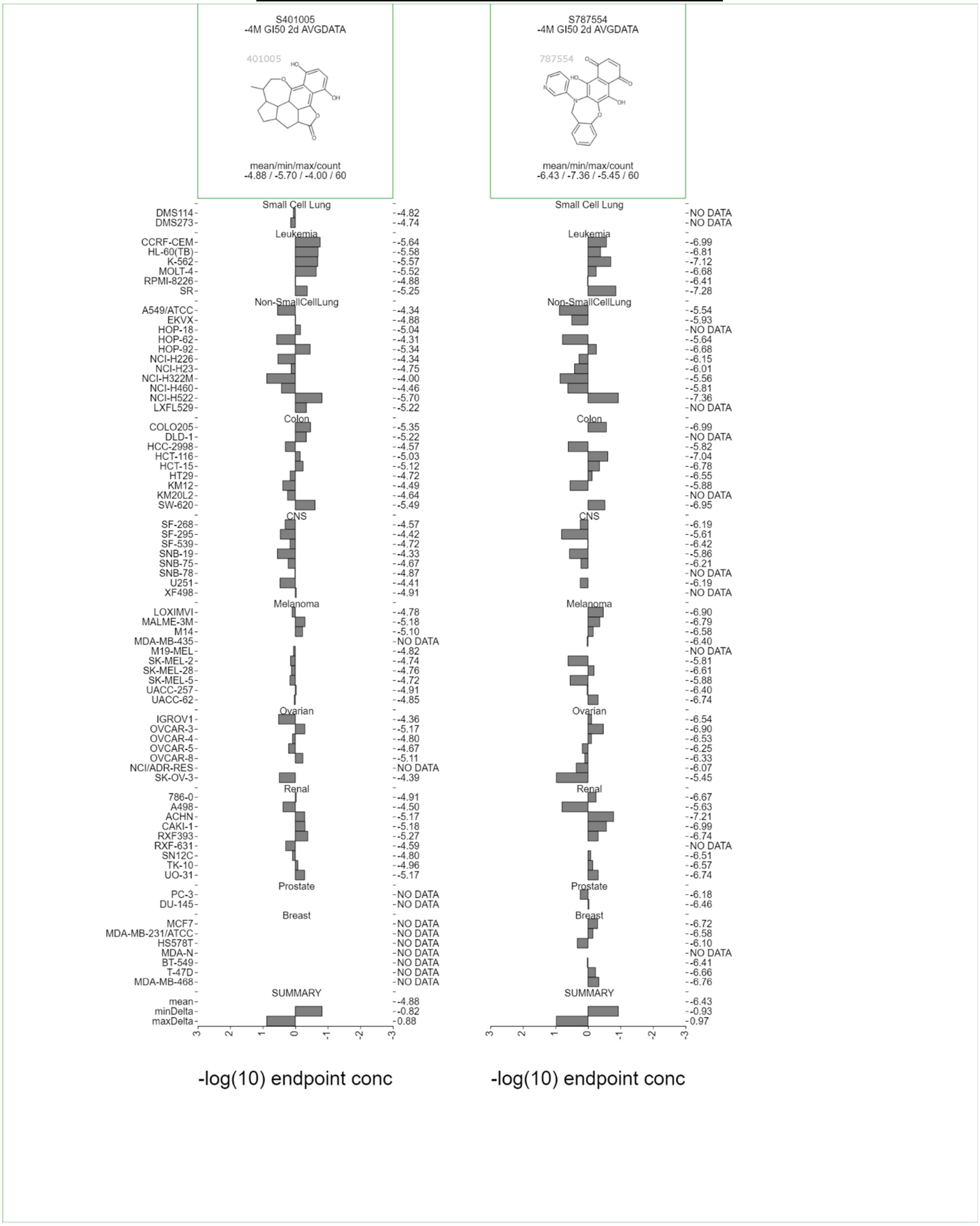

Figure S32

COMPARE Analysis of **5a** (NSC768093) to **13d** (NSC791347)

---SEED---
NSC:S768093 Endpoint:GI50 ExpId:AVGDATA logHiConc:-4.0
Id: 253700 Count Experiments: 2
---TARGET---
NSC:S791347 Endpoint:GI50 ExpId:AVGDATA logHiConc:-4.0
Id: 193820 Count Experiments: 2

Correlation: 0.86
JobId: 979145823477207243

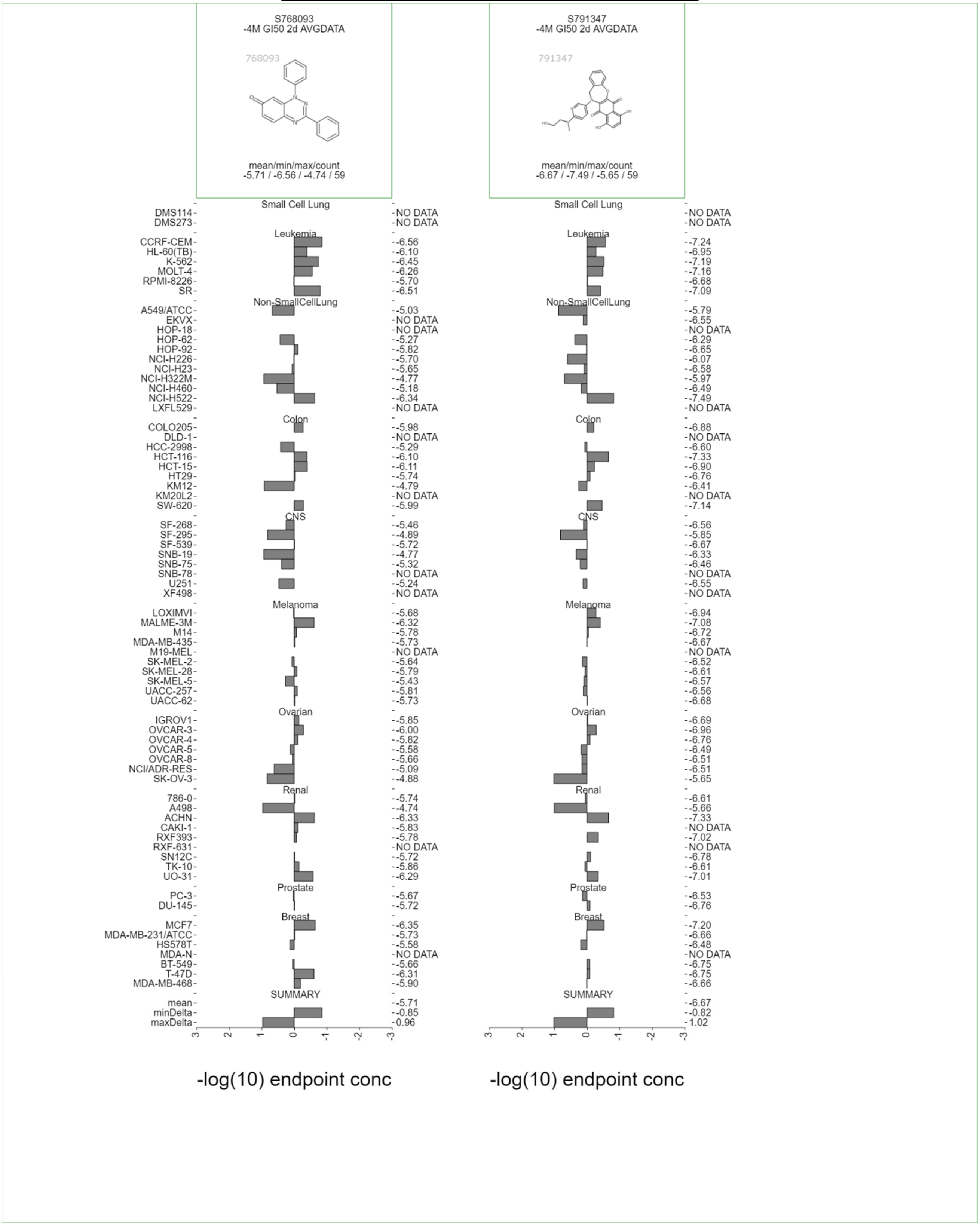

Figure S33

COMPARE Analysis of **13d** (NSC791347) to Pleurotin (NSC401005)

---SEED---
NSC:S791347 Endpoint:GI50 ExpId:AVGDATA logHiConc:-4.0
Id: 193820 Count Experiments: 2
---TARGET---
NSC:S401005 Endpoint:GI50 ExpId:AVGDATA logHiConc:-4.0
Id: 243481 Count Experiments: 2

Correlation: 0.81
JobId: 802301456905305864

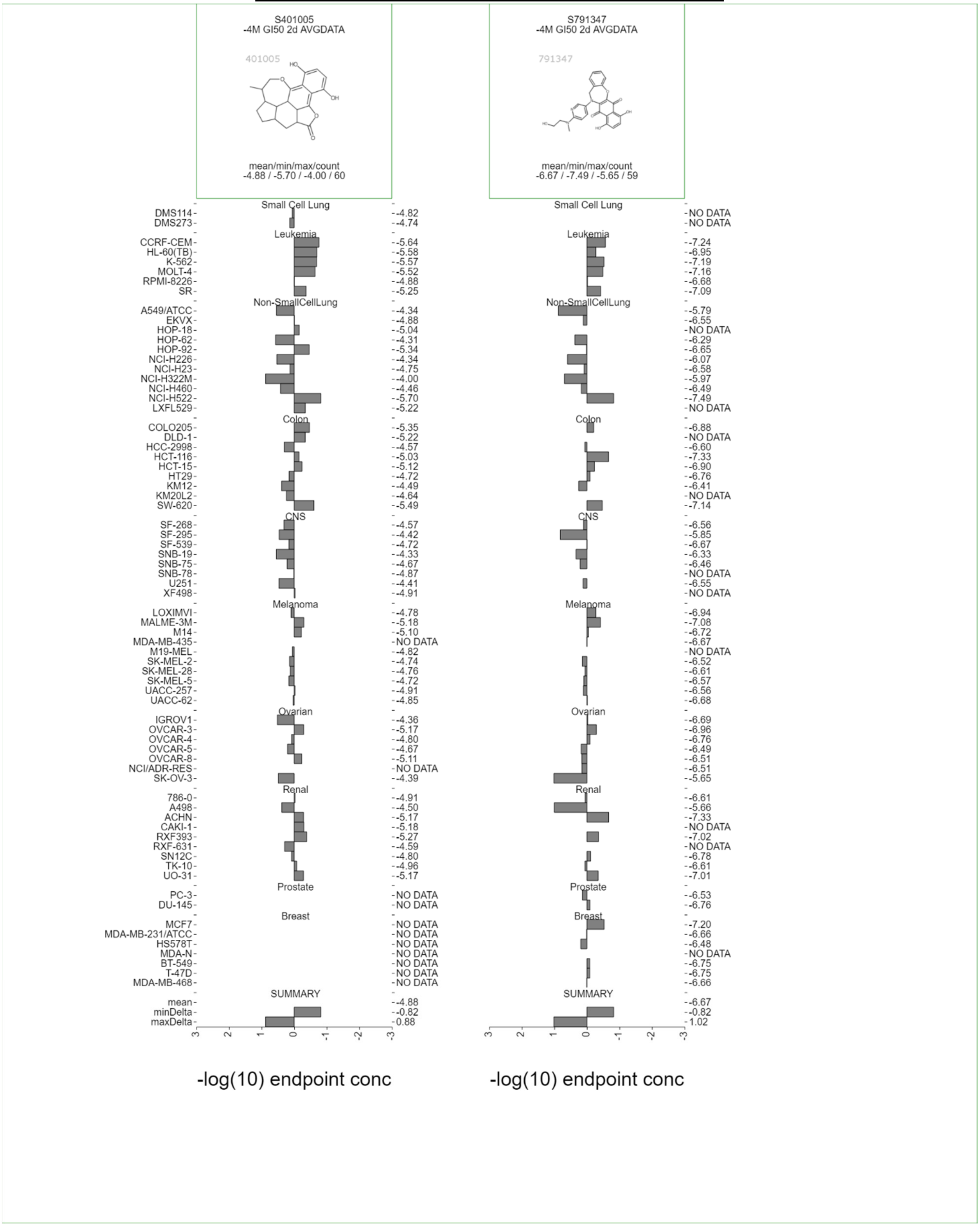

Figure S34

COMPARE Analysis of **5a** (NSC768093) to **14a** (NSC92937)

---SEED---
NSC:S768093 Endpoint:GI50 ExpId:AVGDATA logHiConc:-4.0
Id: 253700 Count Experiments: 2
---TARGET---
NSC:S92937 Endpoint:GI50 ExpId:AVGDATA logHiConc:-4.0
Id: 62826 Count Experiments: 4

Correlation: 0.78
JobId: 6305036997033980351

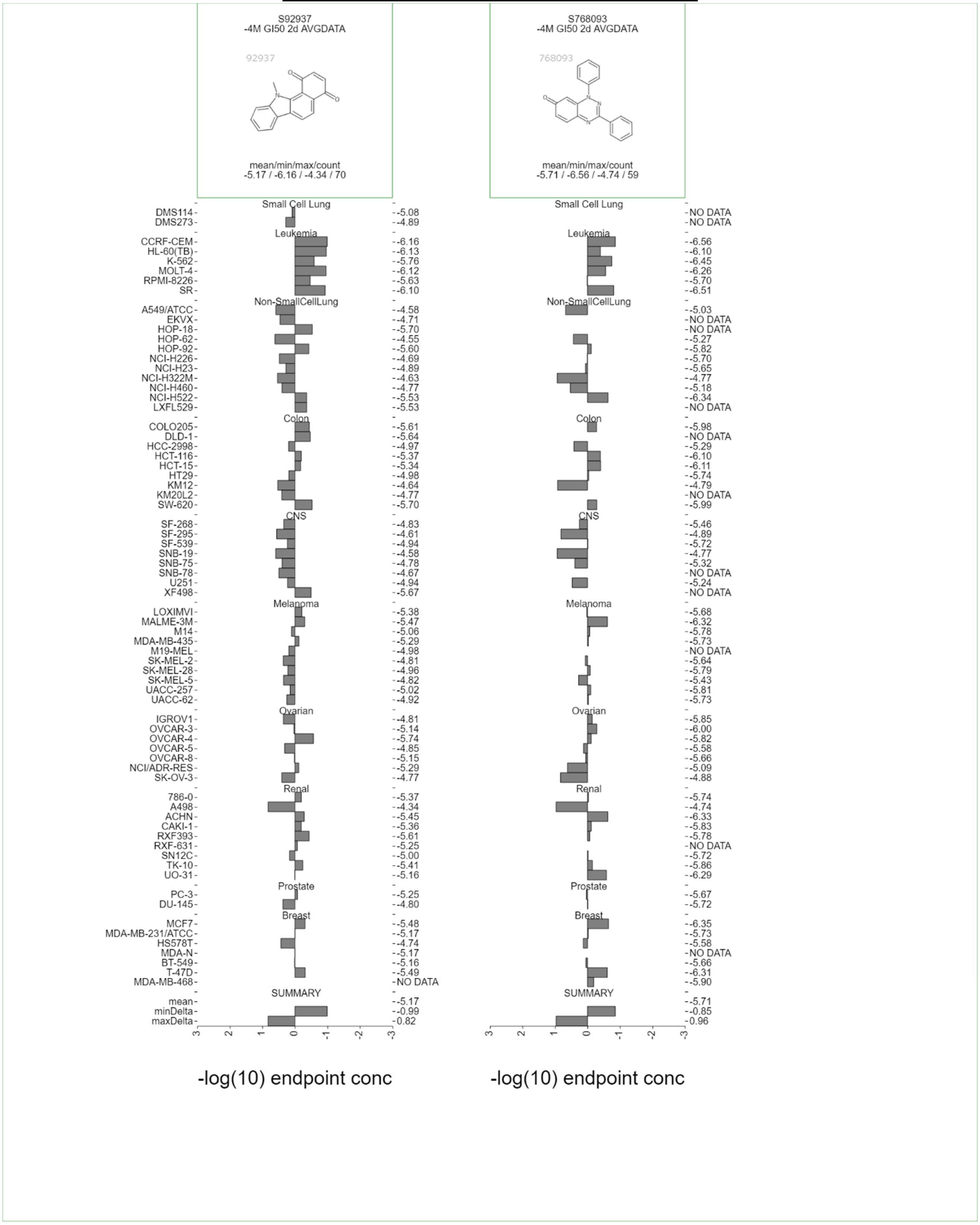

Figure S35

COMPARE Analysis of **14a** (NSC92937) to Pleurotin (NSC401005)

---SEED---
NSC:S92937 Endpoint:GI50 ExpId:AVGDATA logHiConc:-4.0
Id: 62826 Count Experiments: 4
---TARGET---
NSC:S401005 Endpoint:GI50 ExpId:AVGDATA logHiConc:-4.0
Id: 243481 Count Experiments: 2

Correlation: 0.83
JobId: 2862509136855109658

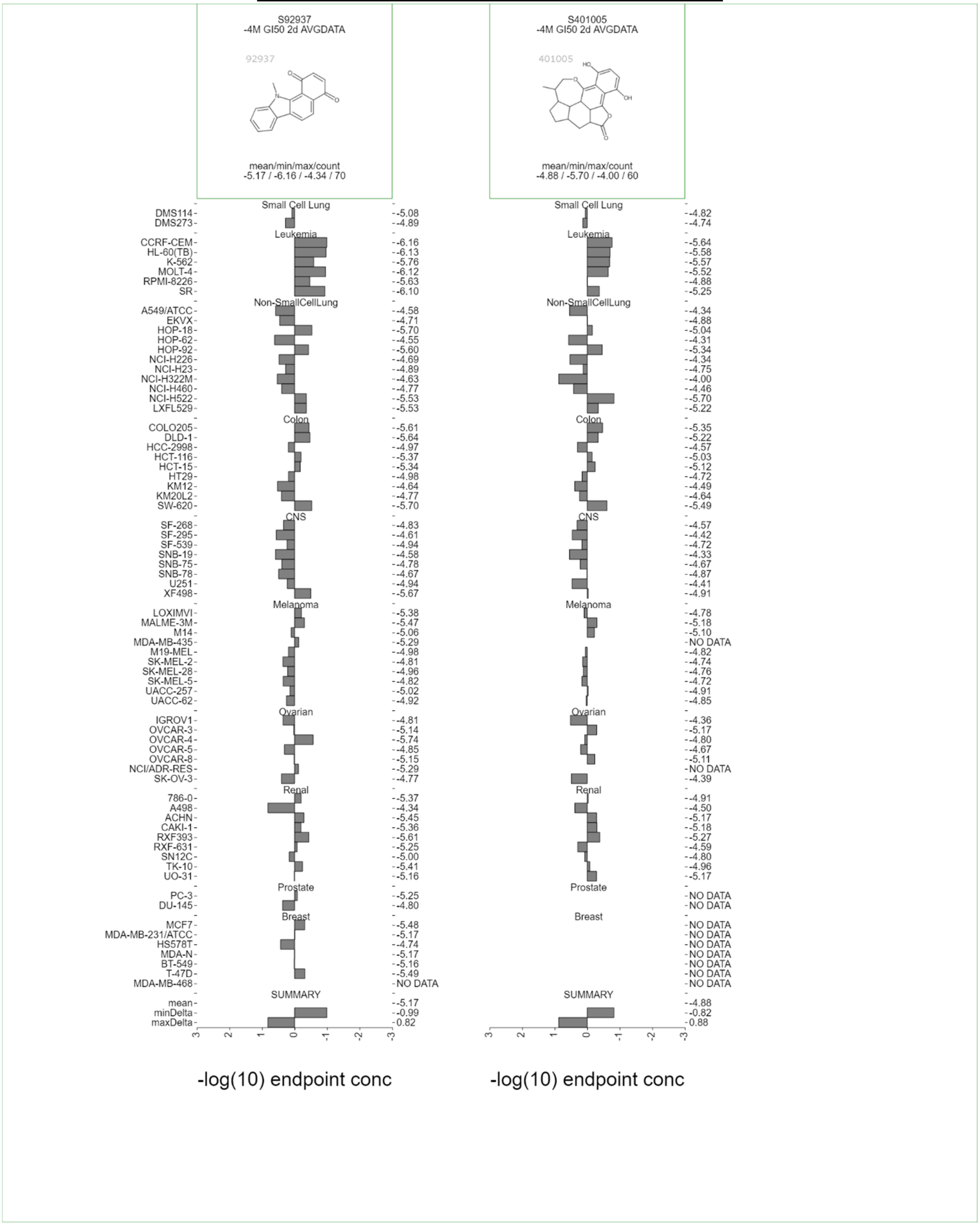

Figure S36

COMPARE Analysis of **5a** (NSC768093) to **14b** (NSC668844)

---SEED---
NSC:S768093 Endpoint:GI50 ExpId:AVGDATA logHiConc:-4.0
Id: 253700 Count Experiments: 2
---TARGET---
NSC:S668844 Endpoint:GI50 ExpId:AVGDATA logHiConc:-4.0
Id: 426305 Count Experiments: 3

Correlation: 0.82
JobId: 608925587626226542

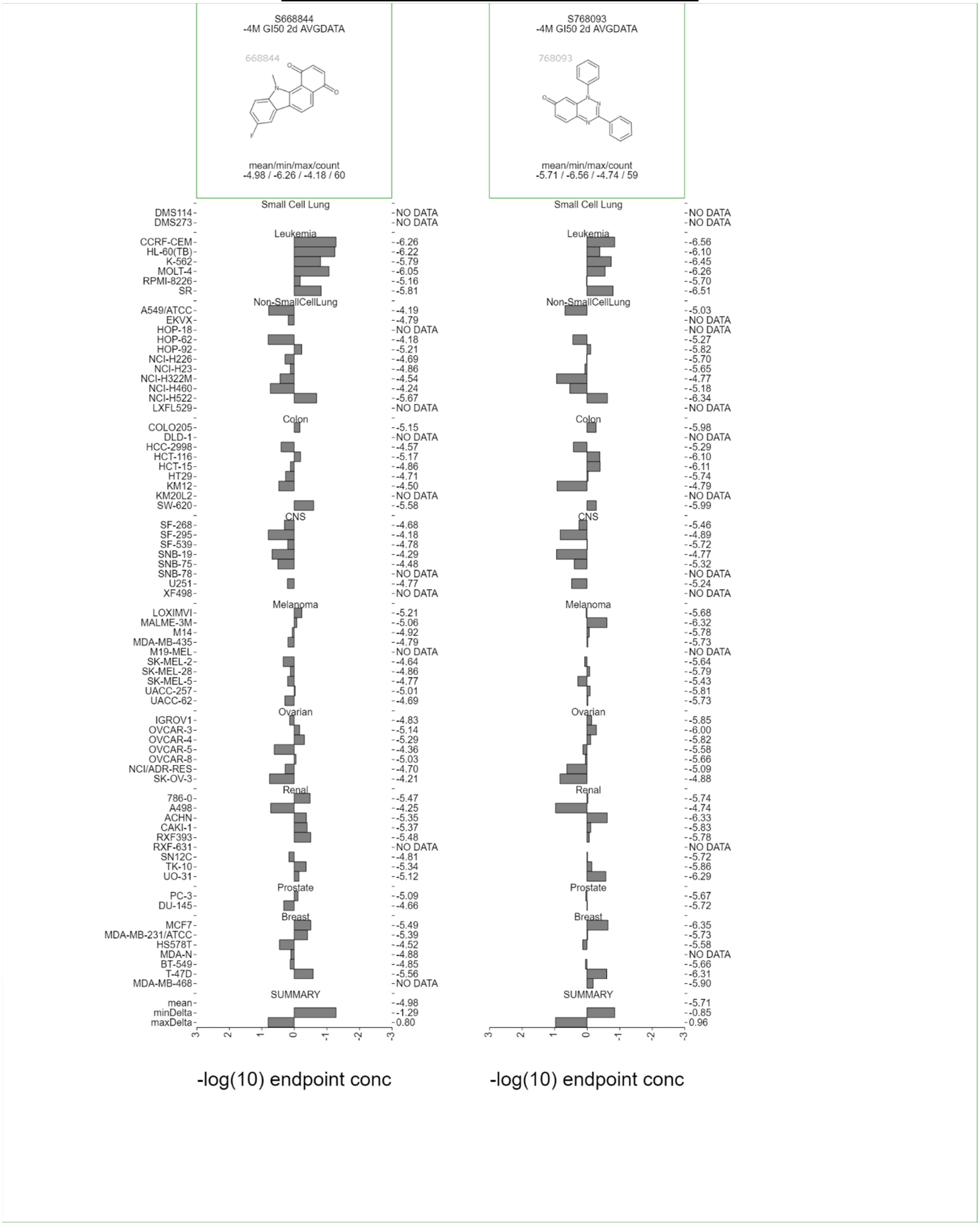

Figure S37

COMPARE Analysis of **14b** (NSC668844) to Pleurotin (NSC401005)

---SEED---
NSC:S668844 Endpoint:GI50 ExpId:AVGDATA logHiConc:-4.0
Id: 426305 Count Experiments: 3
---TARGET---
NSC:S401005 Endpoint:GI50 ExpId:AVGDATA logHiConc:-4.0
Id: 243481 Count Experiments: 2

Correlation: 0.87
JobId: 8039315470687304686

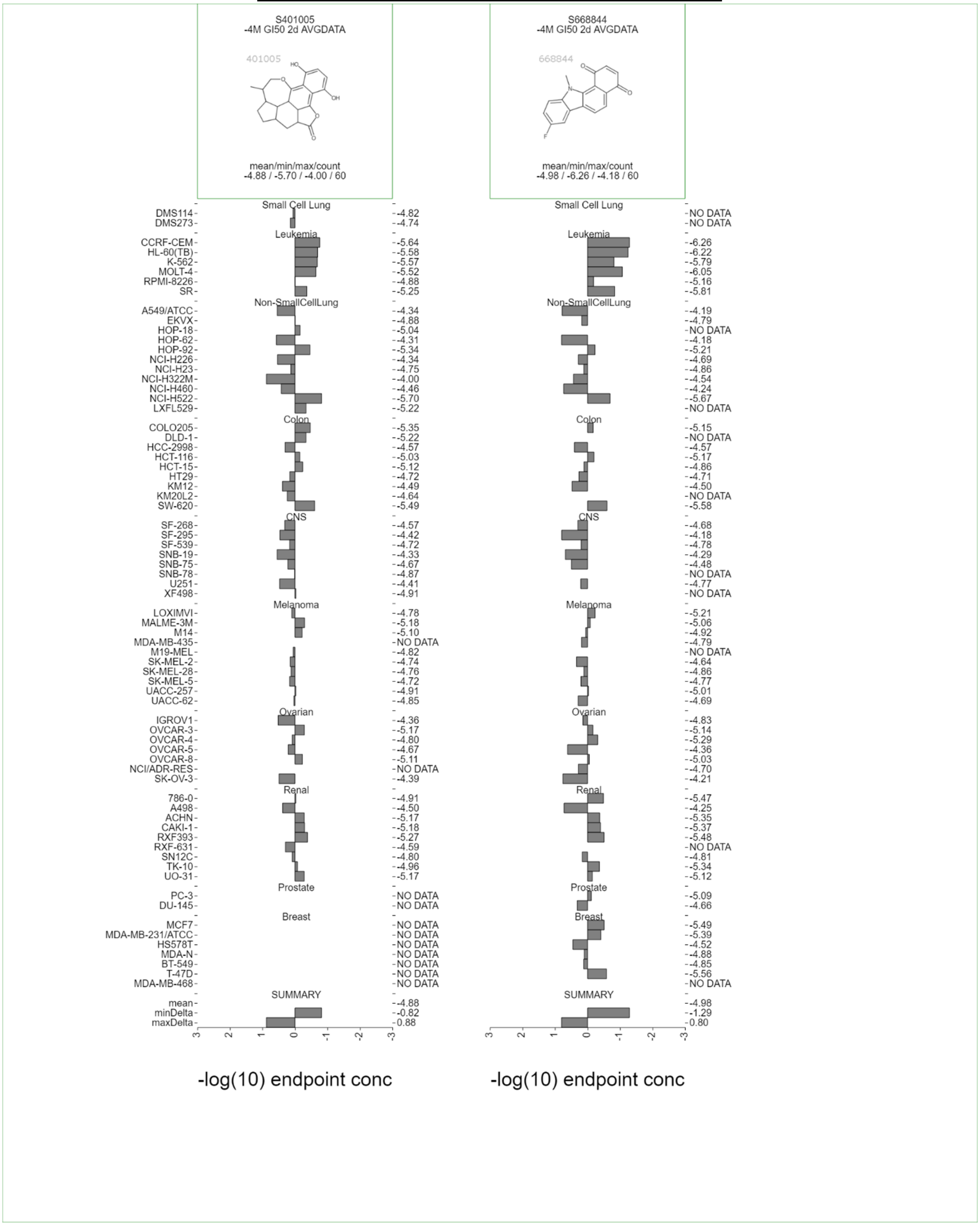

Figure S38

COMPARE Analysis of **5a** (NSC768093) to **14c** (NSC661222)

---SEED---
NSC:S768093 Endpoint:GI50 ExpId:AVGDATA logHiConc:-4.0
Id: 253700 Count Experiments: 2
---TARGET---
NSC:S661222 Endpoint:GI50 ExpId:AVGDATA logHiConc:-4.0
Id: 425825 Count Experiments: 2

Correlation: 0.79
JobId: 4240006198921766318

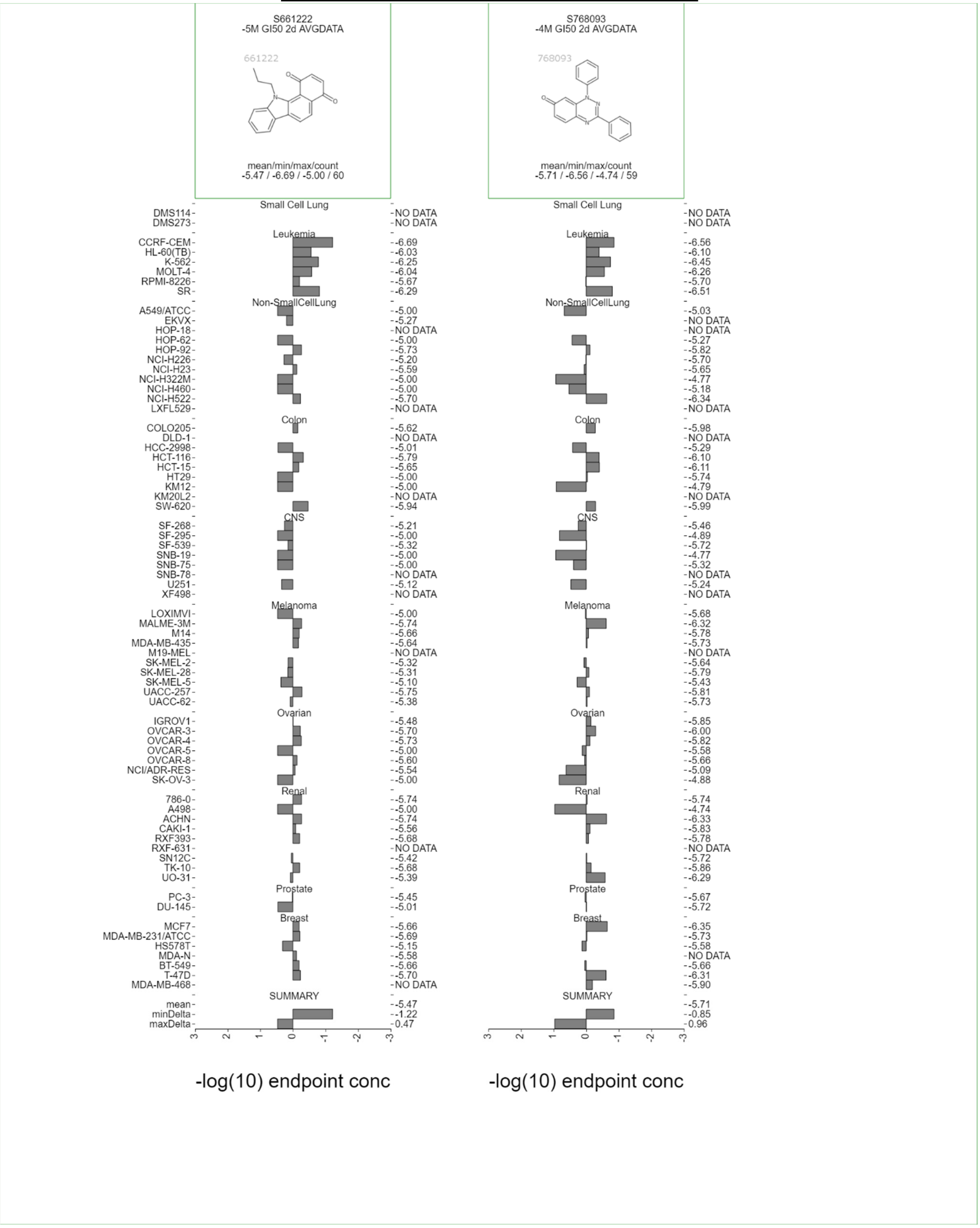

Figure S39

COMPARE Analysis of **14c** (NSC661222) to Pleurotin (NSC401005)

---SEED---
NSC:S661222 Endpoint:GI50 ExpId:AVGDATA logHiConc:-4.0
Id: 425825 Count Experiments: 2
---TARGET---
NSC:S401005 Endpoint:GI50 ExpId:AVGDATA logHiConc:-4.0
Id: 243481 Count Experiments: 2

Correlation: 0.84
JobId: 2729056243356103181

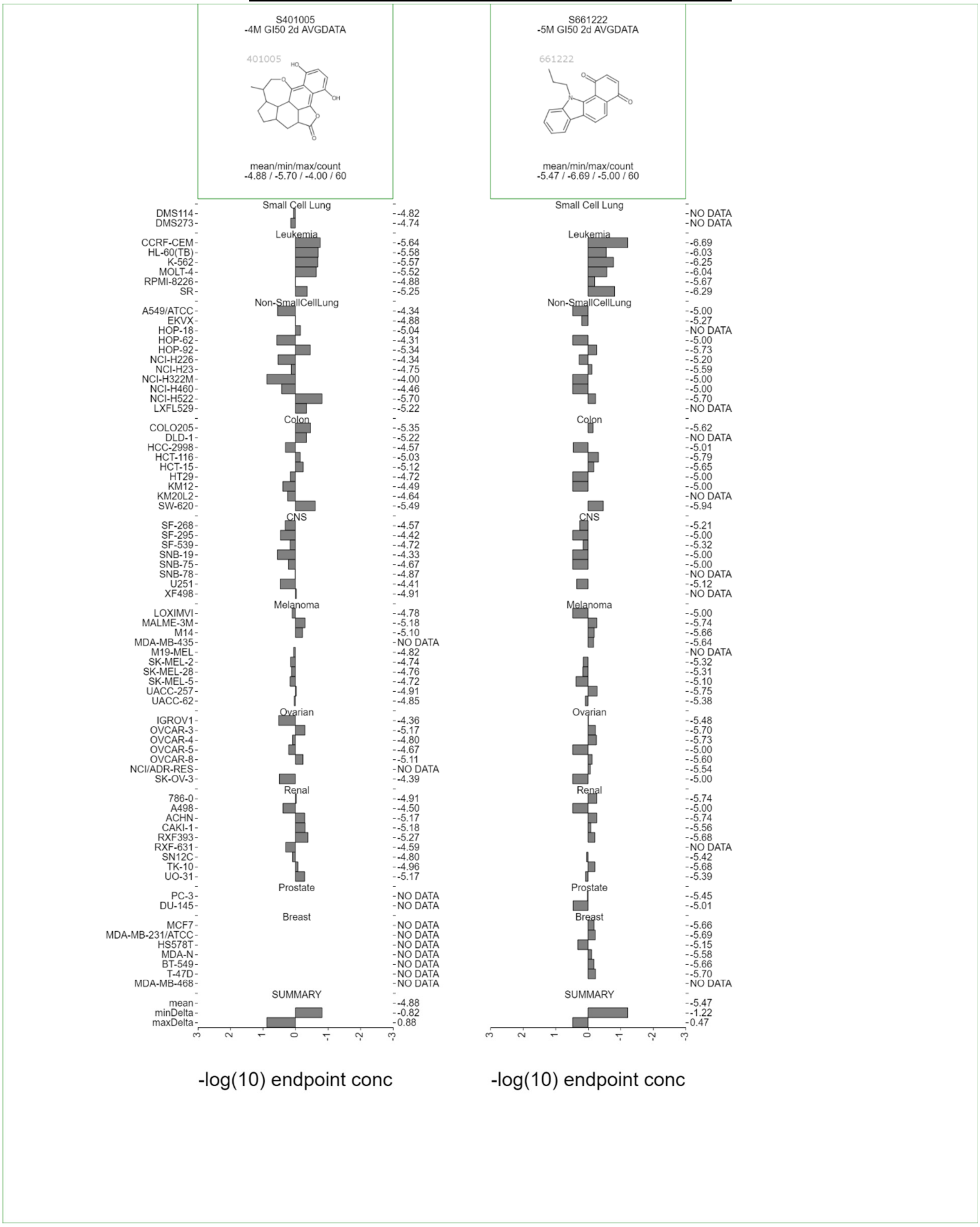

Figure S40

COMPARE Analysis of **5a** (NSC768093) to **14d** (NSC645330)

---SEED---
NSC:S768093 Endpoint:GI50 ExpId:AVGDATA logHiConc:-4.0
Id: 253700 Count Experiments: 2
---TARGET---
NSC:S645330 Endpoint:GI50 ExpId:AVGDATA logHiConc:-4.0
Id: 2668 Count Experiments: 2

Correlation: 0.74
JobId: 6456704583804920861

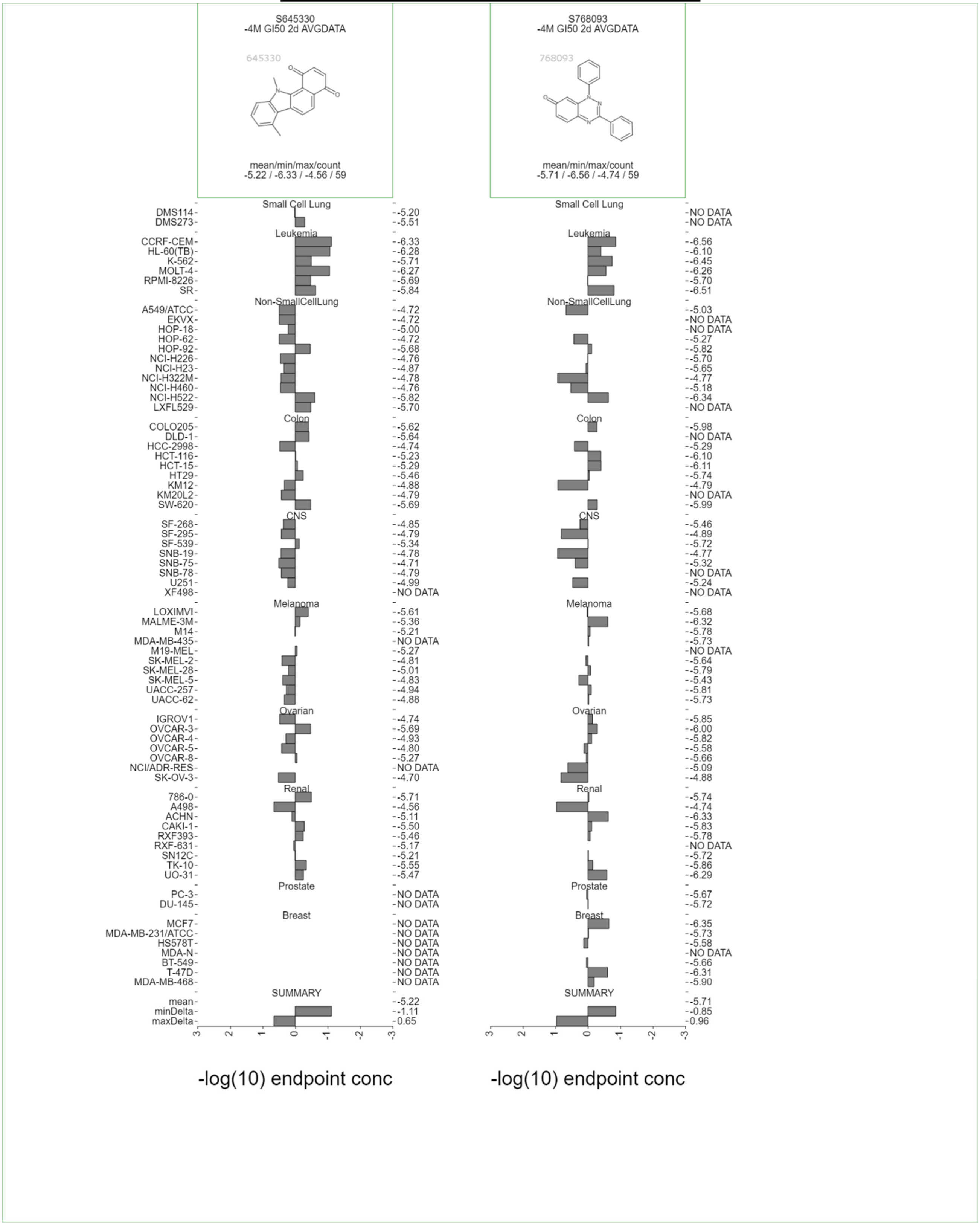

Figure S41

COMPARE Analysis of **14d** (NSC645330) to Pleurotin (NSC401005)

---SEED---
NSC:S645330 Endpoint:GI50 ExpId:AVGDATA logHiConc:-4.0
Id: 2668 Count Experiments: 2
---TARGET---
NSC:S401005 Endpoint:GI50 ExpId:AVGDATA logHiConc:-4.0
Id: 243481 Count Experiments: 2

Correlation: 0.82
JobId: 8924669832522260190

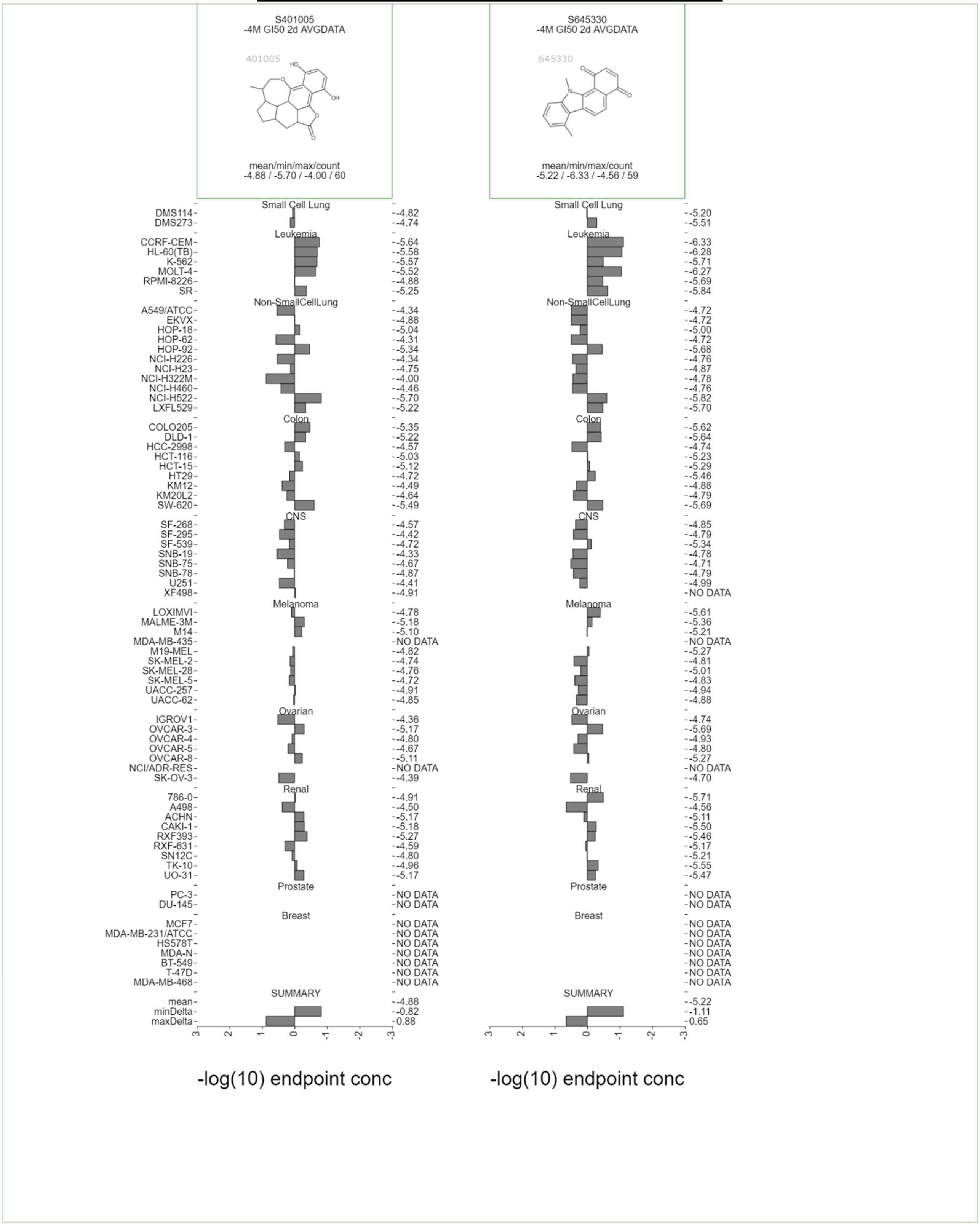

Figure S42

COMPARE Analysis of **5a** (NSC768093) to **14e** (NSC661221)

---SEED---
NSC:S768093 Endpoint:GI50 ExpId:AVGDATA logHiConc:-4.0
Id: 253700 Count Experiments: 2
---TARGET---
NSC:S661221 Endpoint:GI50 ExpId:AVGDATA logHiConc:-4.0
Id: 425824 Count Experiments: 2

Correlation: 0.78
JobId: 2832546849340558660

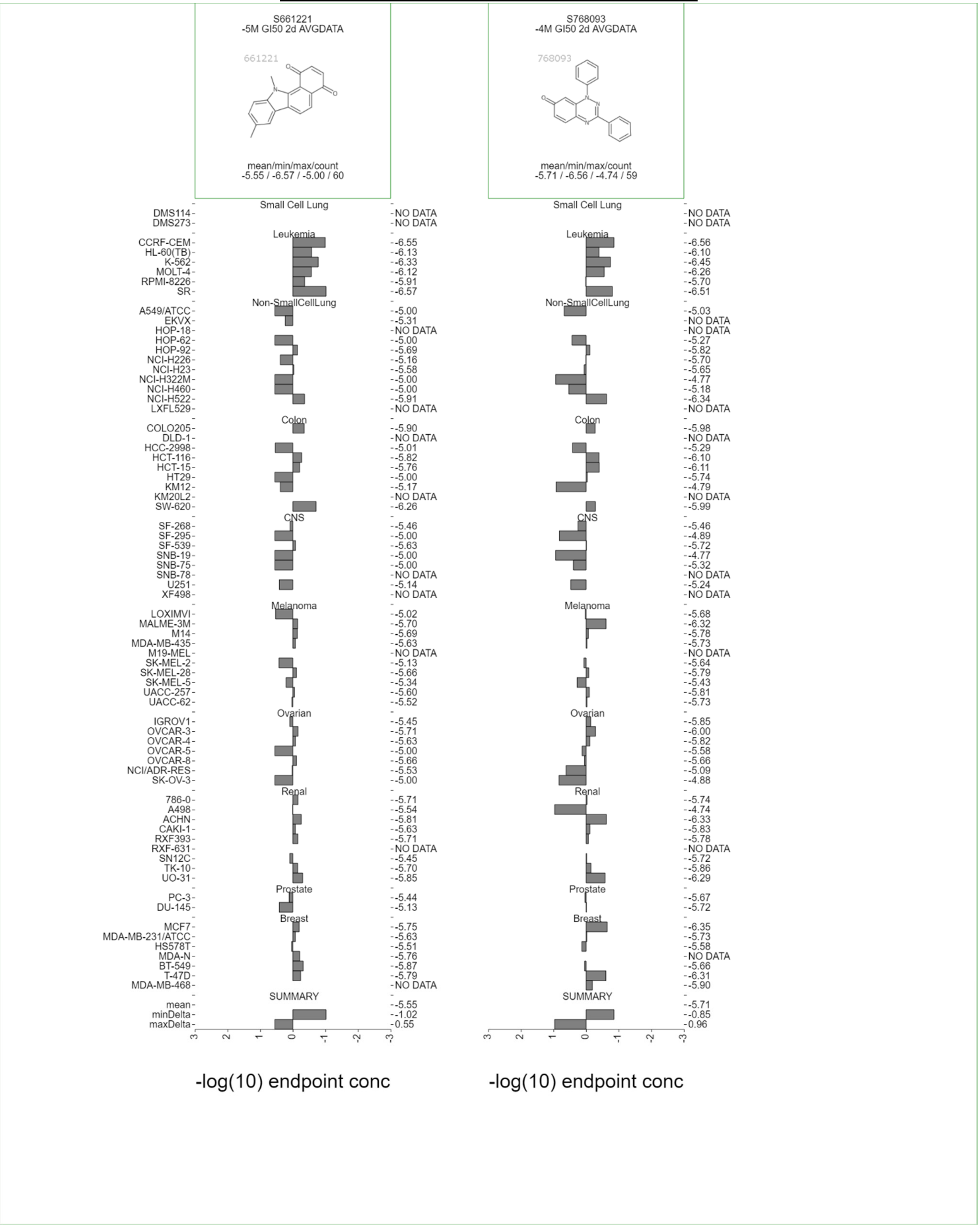

Figure S43

COMPARE Analysis of **14e** (NSC661221) to Pleurotin (NSC401005)

---SEED---
NSC:S661221 Endpoint:GI50 ExpId:AVGDATA logHiConc:-4.0
Id: 425824 Count Experiments: 2
---TARGET---
NSC:S401005 Endpoint:GI50 ExpId:AVGDATA logHiConc:-4.0
Id: 243481 Count Experiments: 2

Correlation: 0.85
JobId: 8272752536982514267

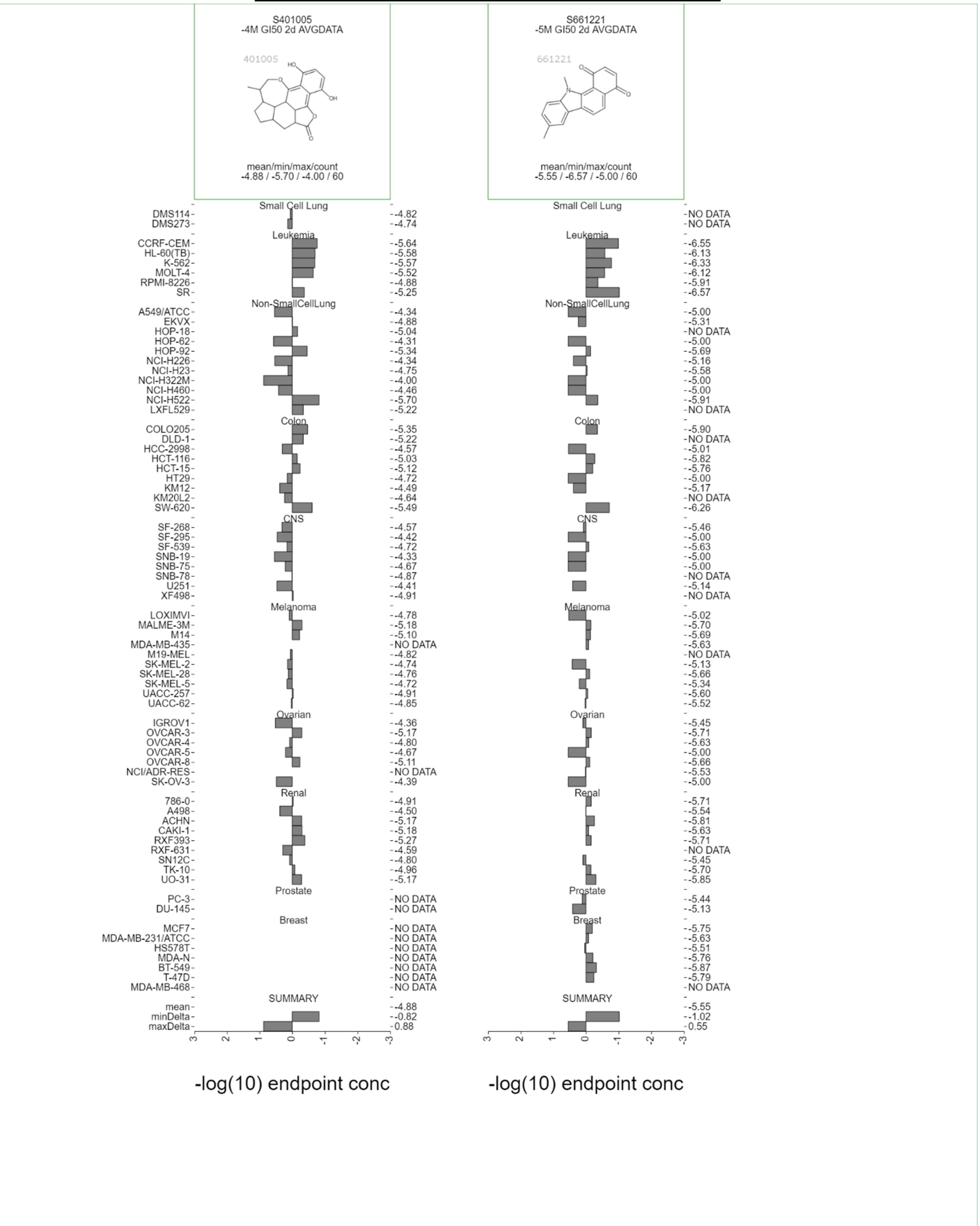

Figure S44

COMPARE Analysis of **5a** (NSC768093) to **14f** (NSC641396)

---SEED---
NSC:S768093 Endpoint:GI50 ExpId:AVGDATA logHiConc:-4.0
Id: 253700 Count Experiments: 2
---TARGET---
NSC:S641396 Endpoint:GI50 ExpId:AVGDATA logHiConc:-4.0
Id: 182589 Count Experiments: 4

Correlation: 0.77
JobId: 6992511647736348161

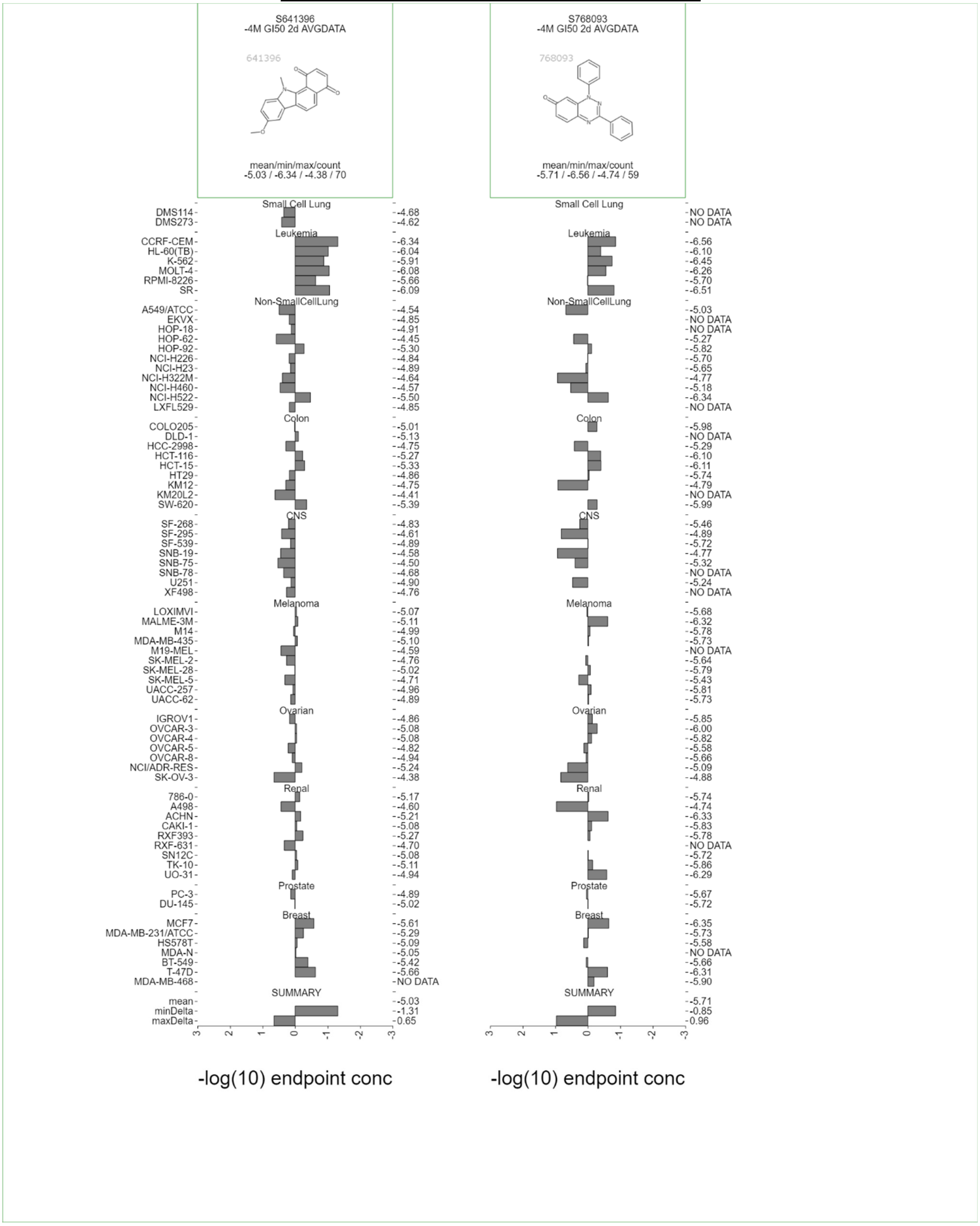

Figure S45

COMPARE Analysis of **14f** (NSC641396) to Pleurotin (NSC401005)

---SEED---
NSC:S641396 Endpoint:GI50 ExpId:AVGDATA logHiConc:-4.0
Id: 182589 Count Experiments: 4
---TARGET---
NSC:S401005 Endpoint:GI50 ExpId:AVGDATA logHiConc:-4.0
Id: 243481 Count Experiments: 2

Correlation: 0.78
JobId: 7250576864606418500

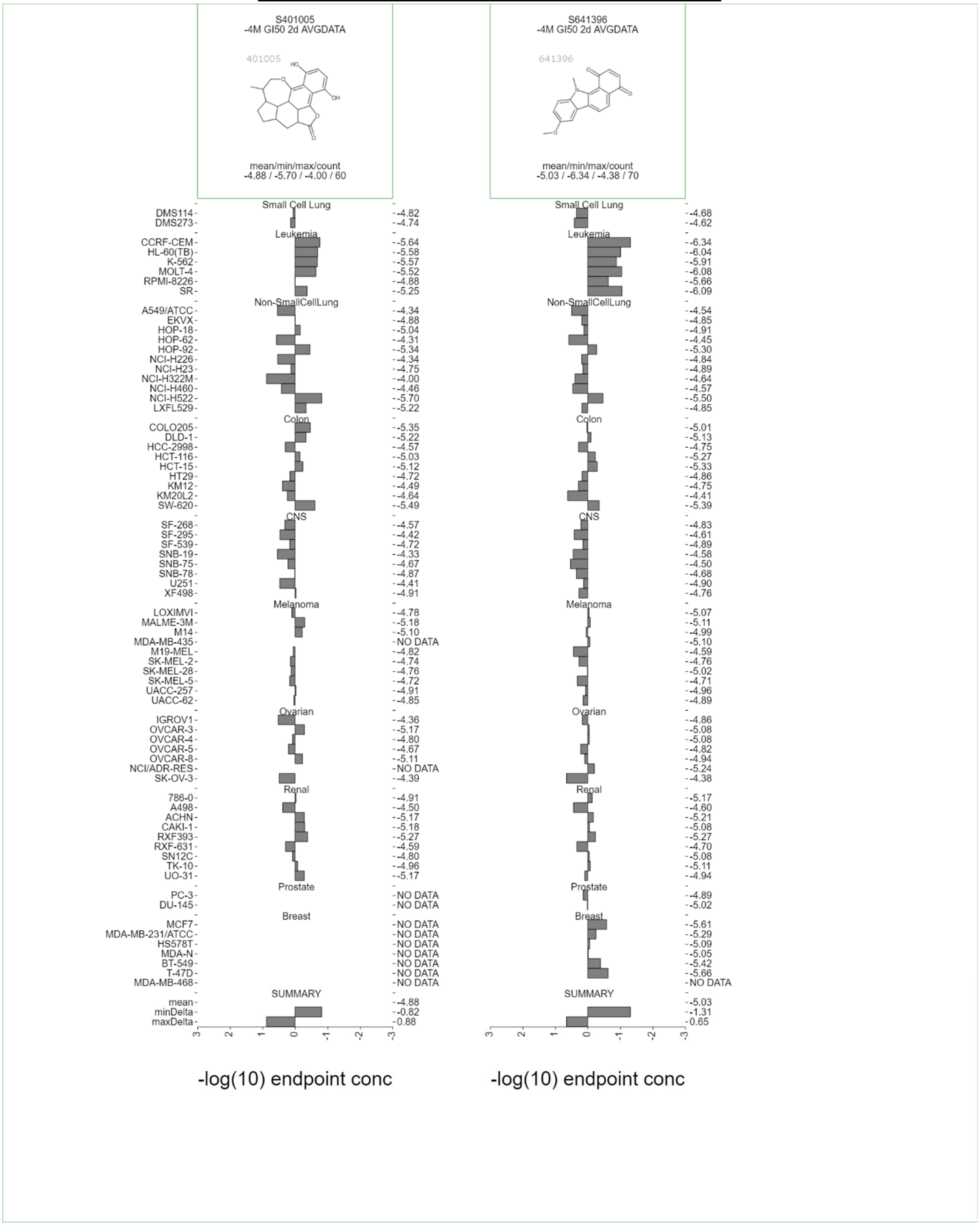

Figure S46

COMPARE Analysis of **5a** (NSC768093) to Kalafungin (NSC137443)

---SEED---
NSC:S768093 Endpoint:GI50 ExpId:AVGDATA logHiConc:-4.0
Id: 253700 Count Experiments: 2
---TARGET---
NSC:S137443 Endpoint:GI50 ExpId:AVGDATA logHiConc:-4.0
Id: 71608 Count Experiments: 2

Correlation: 0.81
JobId: 9060594243988086729

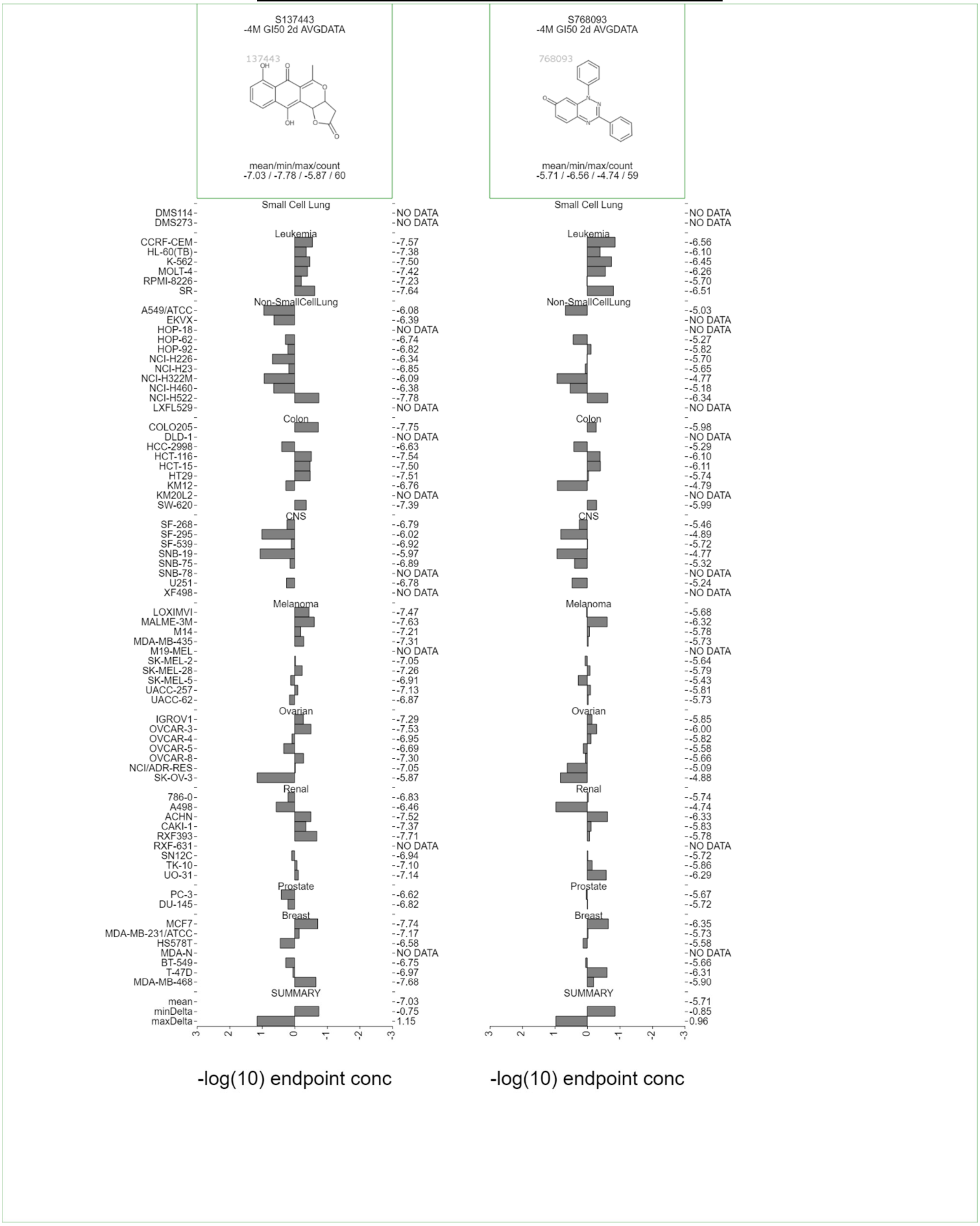

Figure S47

COMPARE Analysis of Kalafungin (NSC137443) to Pleurotin (NSC401005)

---SEED---
NSC:S137443 Endpoint:GI50 ExpId:AVGDATA logHiConc:-4.0
Id: 71608 Count Experiments: 2

---TARGET---
NSC:S401005 Endpoint:GI50 ExpId:AVGDATA logHiConc:-4.0
Id: 243481 Count Experiments: 2

Correlation: 0.79
JobId: 613988420058587479

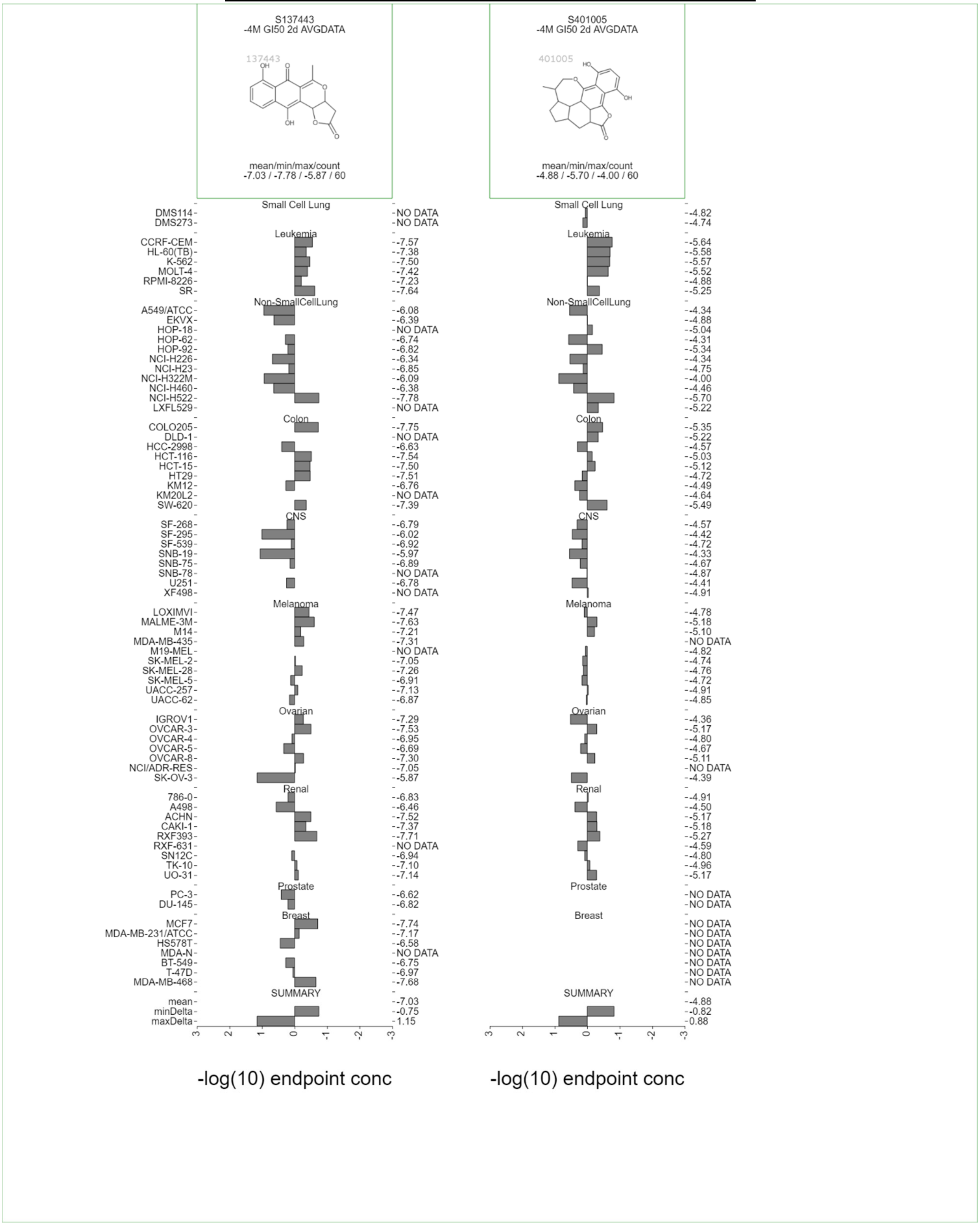

Figure S48

COMPARE Analysis of **5a** (NSC768093) to Nanaomycin A (NSC267461)

---SEED---
NSC:S768093 Endpoint:GI50 ExpId:AVGDATA logHiConc:-4.0
Id: 253700 Count Experiments: 2
---TARGET---
NSC:S267461 Endpoint:GI50 ExpId:AVGDATA logHiConc:-4.0
Id: 121294 Count Experiments: 4

Correlation: 0.72
JobId: 4481058418583182031

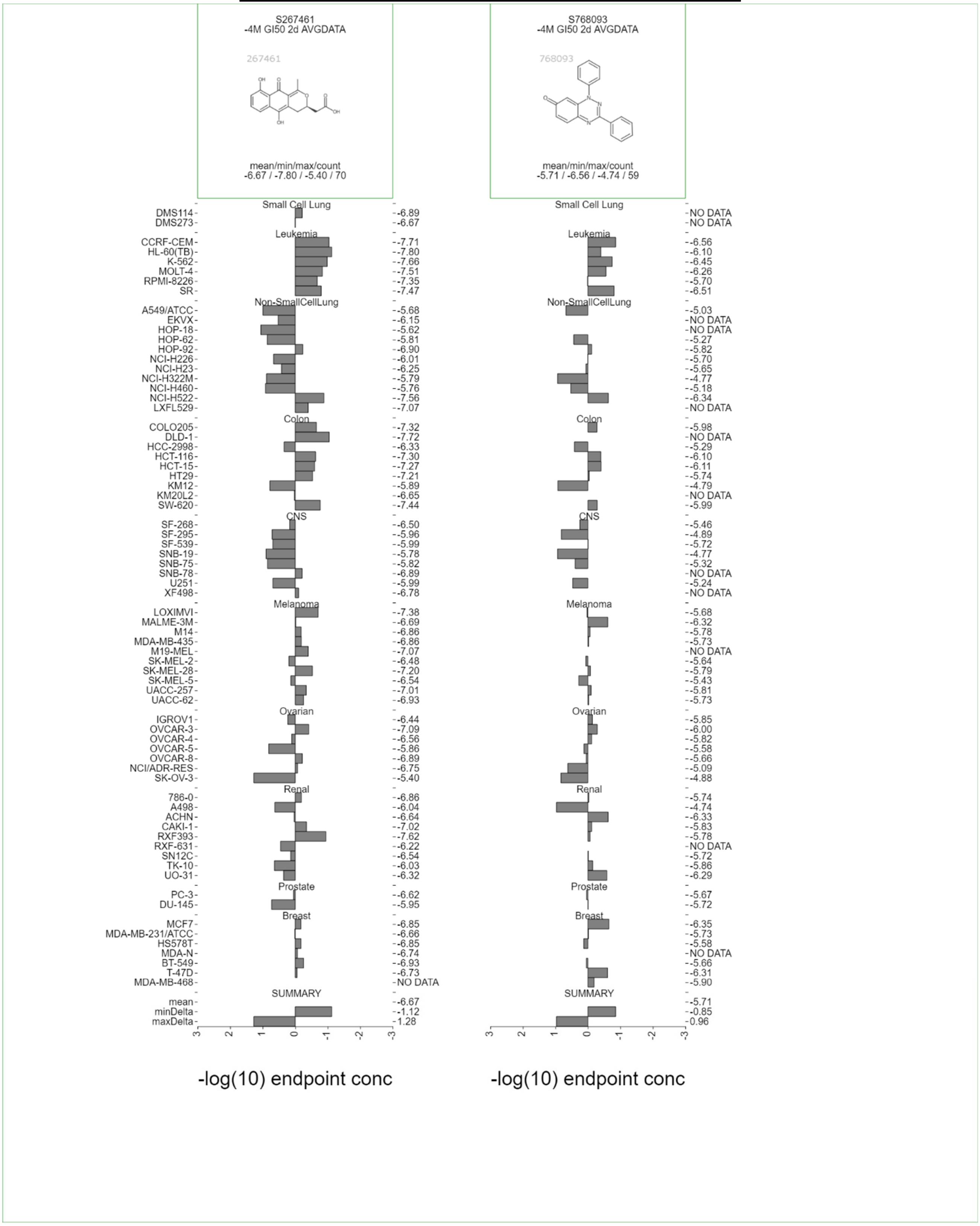

Figure S49

COMPARE Analysis of Nanaomycin A (NSC267461) to Pleurotin (NSC401005)

---SEED---
NSC:S267461 Endpoint:GI50 ExpId:AVGDATA logHiConc:-4.0
Id: 121294 Count Experiments: 4
---TARGET---
NSC:S401005 Endpoint:GI50 ExpId:AVGDATA logHiConc:-4.0
Id: 243481 Count Experiments: 2

Correlation: 0.79
JobId: 2128577860017888719

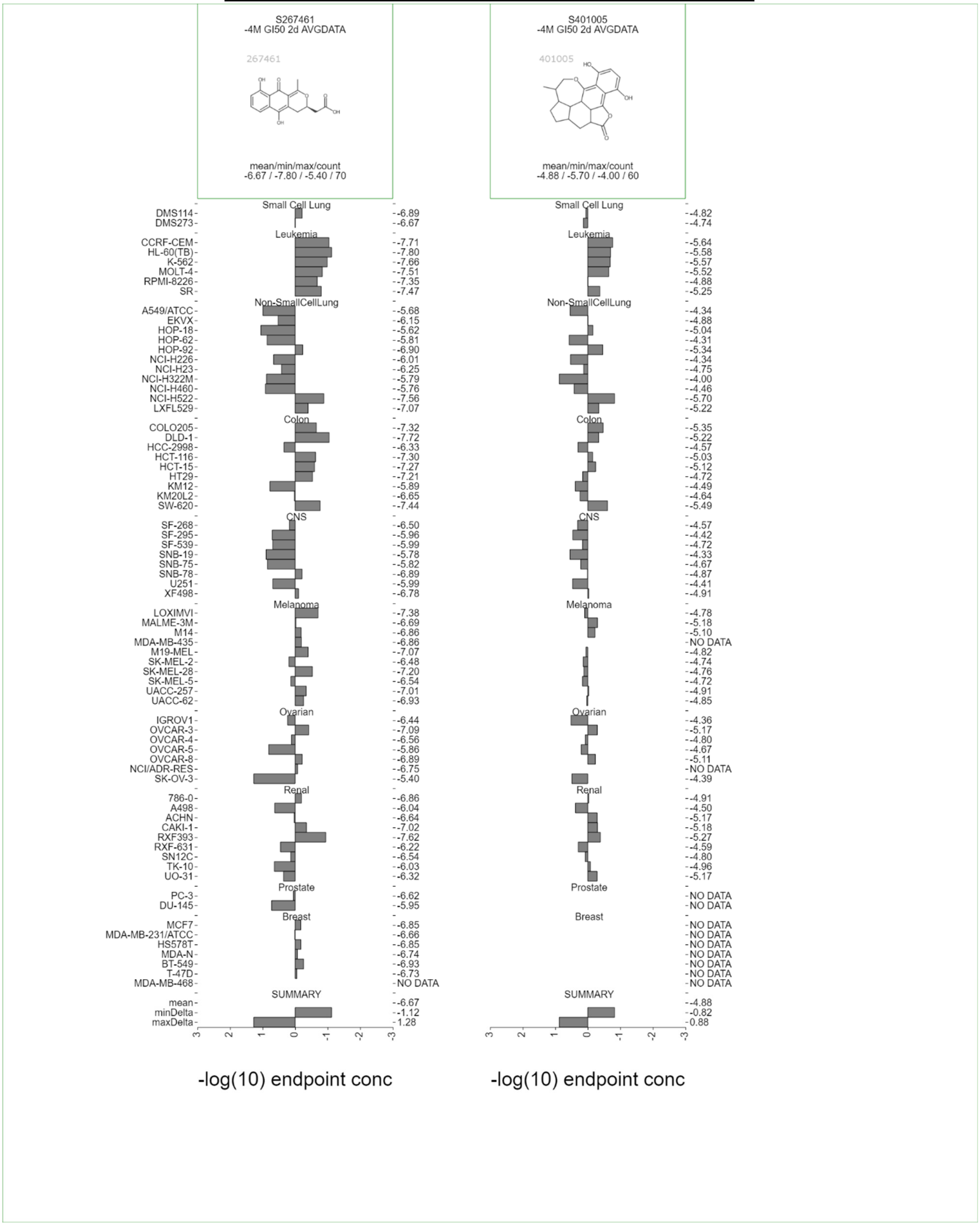

Figure S50

COMPARE Analysis of 5a (NSC768093) to 15a (NSC641401)

---SEED---
NSC:S768093 Endpoint:GI50 ExpId:AVGDATA logHiConc:-4.0
Id: 253700 Count Experiments: 2
---TARGET---
NSC:S641401 Endpoint:GI50 ExpId:AVGDATA logHiConc:-4.0
Id: 303785 Count Experiments: 1

Correlation: 0.78
JobId: 5187925863101103006

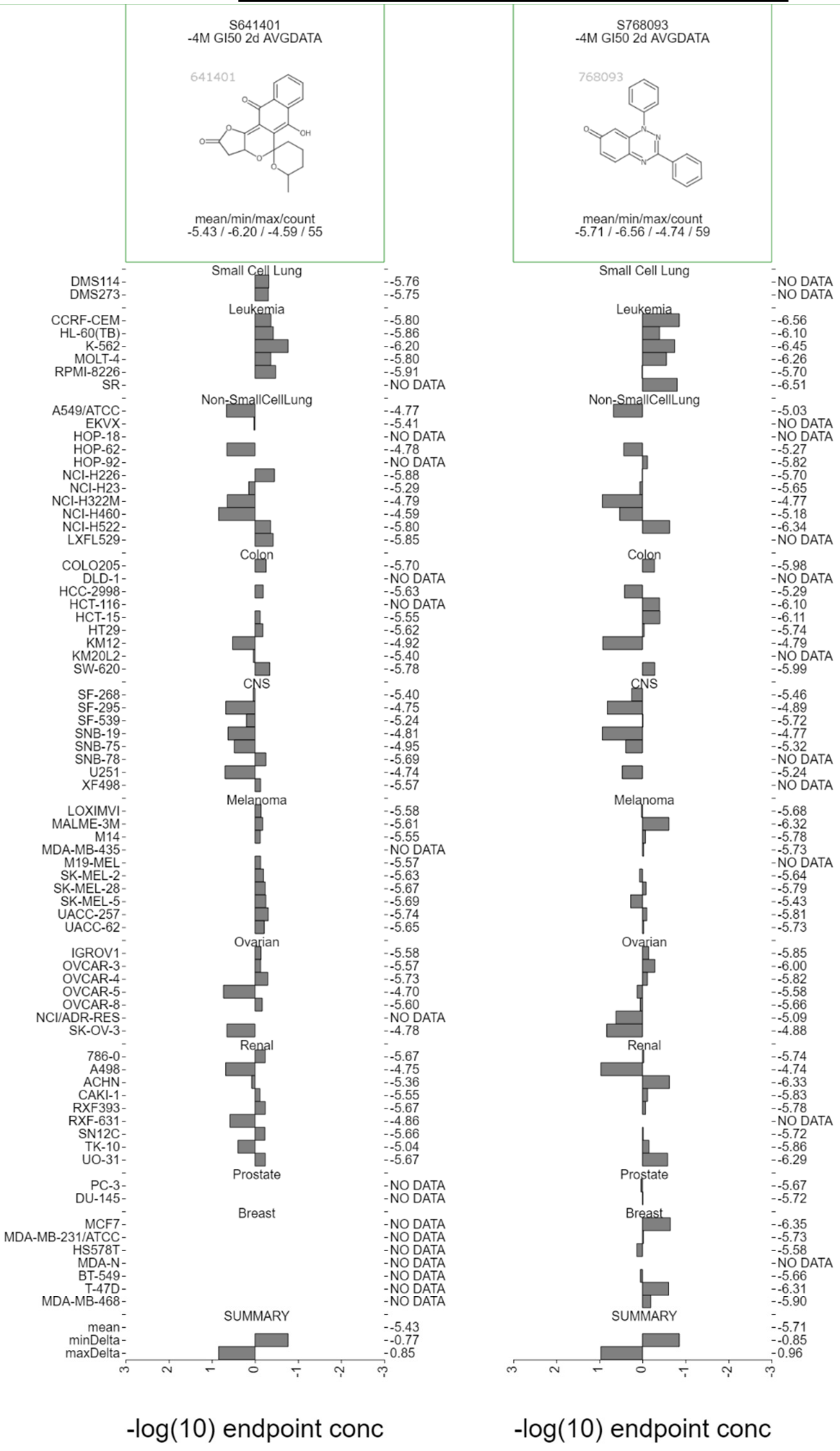

COMPARE Analysis of **15a** (NSC641401) to Pleurotin (NSC401005)

---SEED---
NSC:S641401 Endpoint:GI50 ExpId:AVGDATA logHiConc:-4.0
Id: 303785 Count Experiments: 1
---TARGET---
NSC:S401005 Endpoint:GI50 ExpId:AVGDATA logHiConc:-4.0
Id: 243481 Count Experiments: 2

Correlation: 0.68
JobId: 9106697070602025420

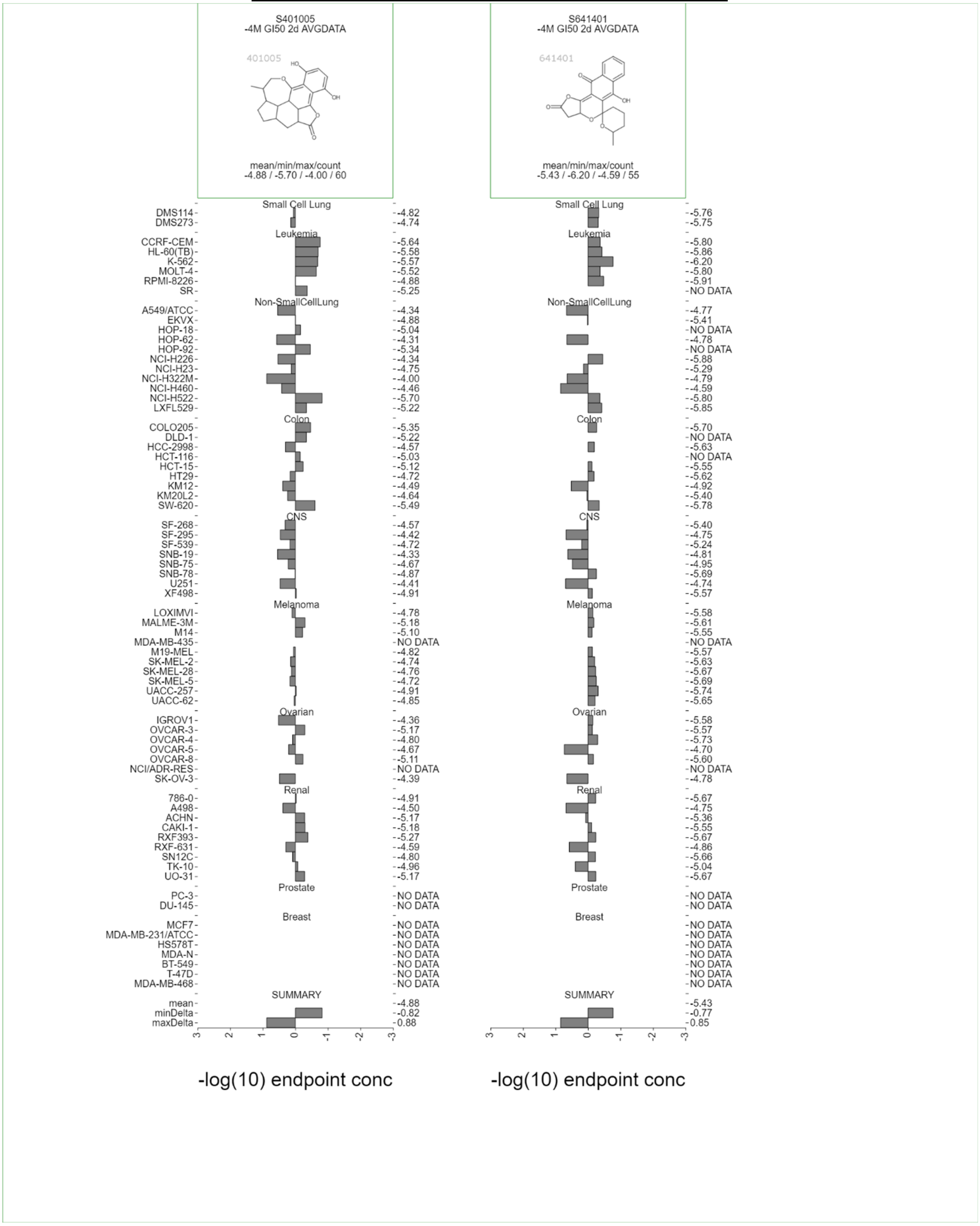

Figure S52

COMPARE Analysis of **5a** (NSC768093) to Discorhabdin C (NSC626162)

---SEED---
NSC:S768093 Endpoint:GI50 ExpId:AVGDATA logHiConc:-4.0
Id: 253700 Count Experiments: 2
---TARGET---
NSC:S626162 Endpoint:GI50 ExpId:AVGDATA logHiConc:-4.0
Id: 421973 Count Experiments: 5

Correlation: 0.78
JobId: 193534783601839350

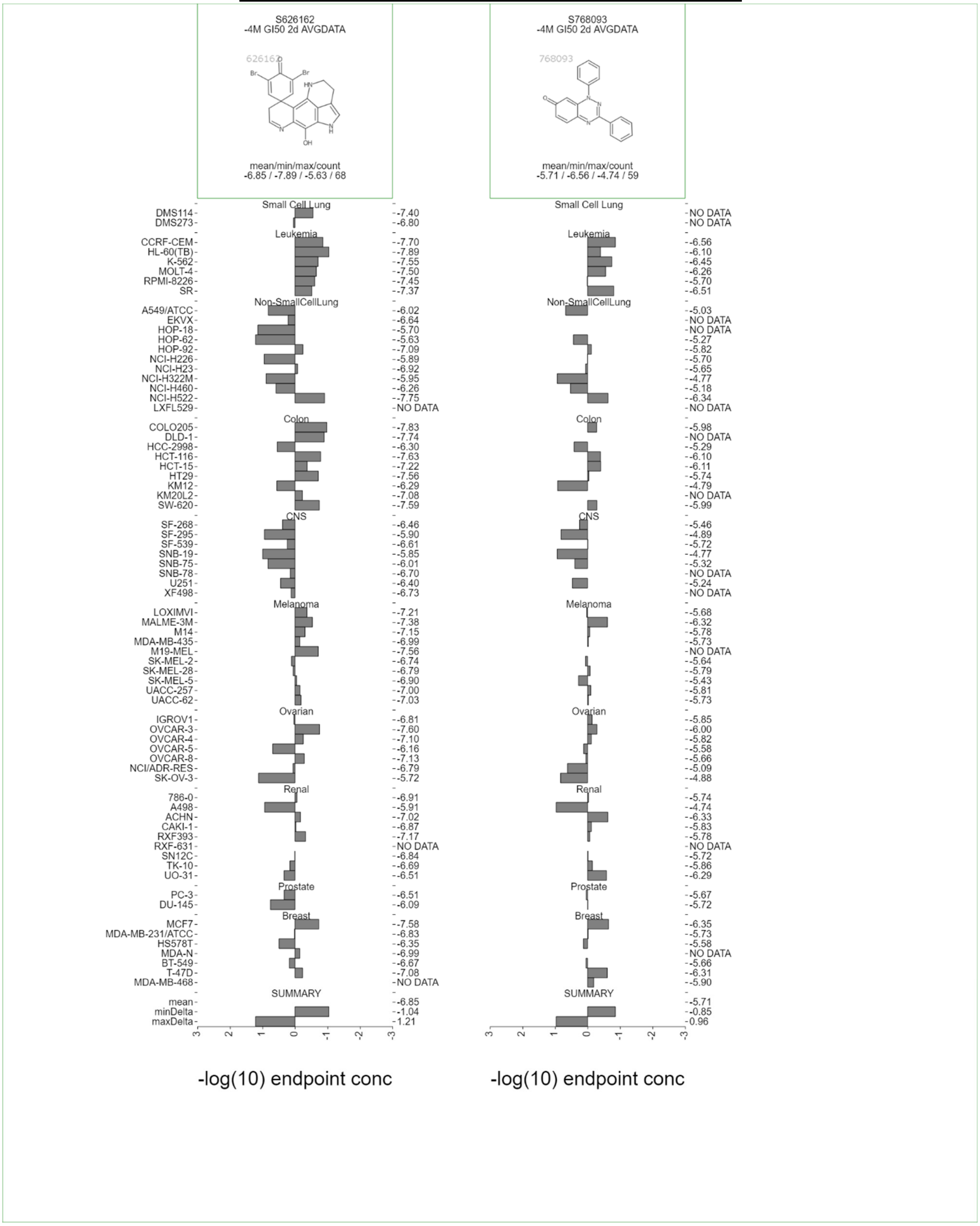

COMPARE Analysis of Discorhabdin C (NSC626162) to Pleurotin (NSC401005)

---SEED---
NSC:S626162 Endpoint:GI50 ExpId:AVGDATA logHiConc:-4.0
Id: 421973 Count Experiments: 5
---TARGET---
NSC:S401005 Endpoint:GI50 ExpId:AVGDATA logHiConc:-4.0
Id: 243481 Count Experiments: 2

Correlation: 0.78
JobId: 1403932220819377460

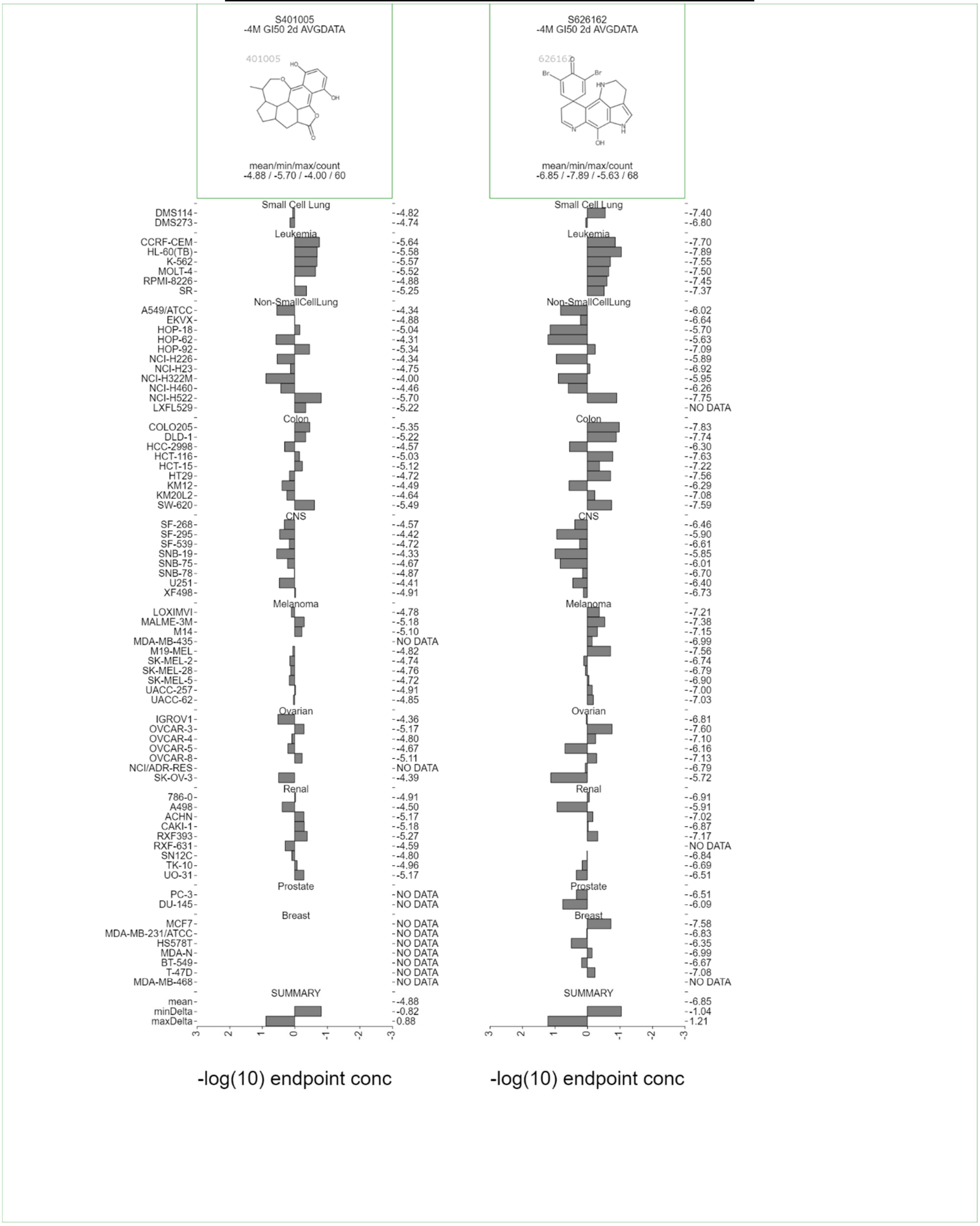

COMPARE Analysis of **5a** (NSC768093) to **9b** (NSC682995)

---SEED---
NSC:S768093 Endpoint:GI50 ExpId:AVGDATA logHiConc:-4.0
Id: 253700 Count Experiments: 2
---TARGET---
NSC:S682995 Endpoint:GI50 ExpId:AVGDATA logHiConc:-4.0
Id: 4367529 Count Experiments: 2

Correlation: 0.71
JobId: 5303183562120266702

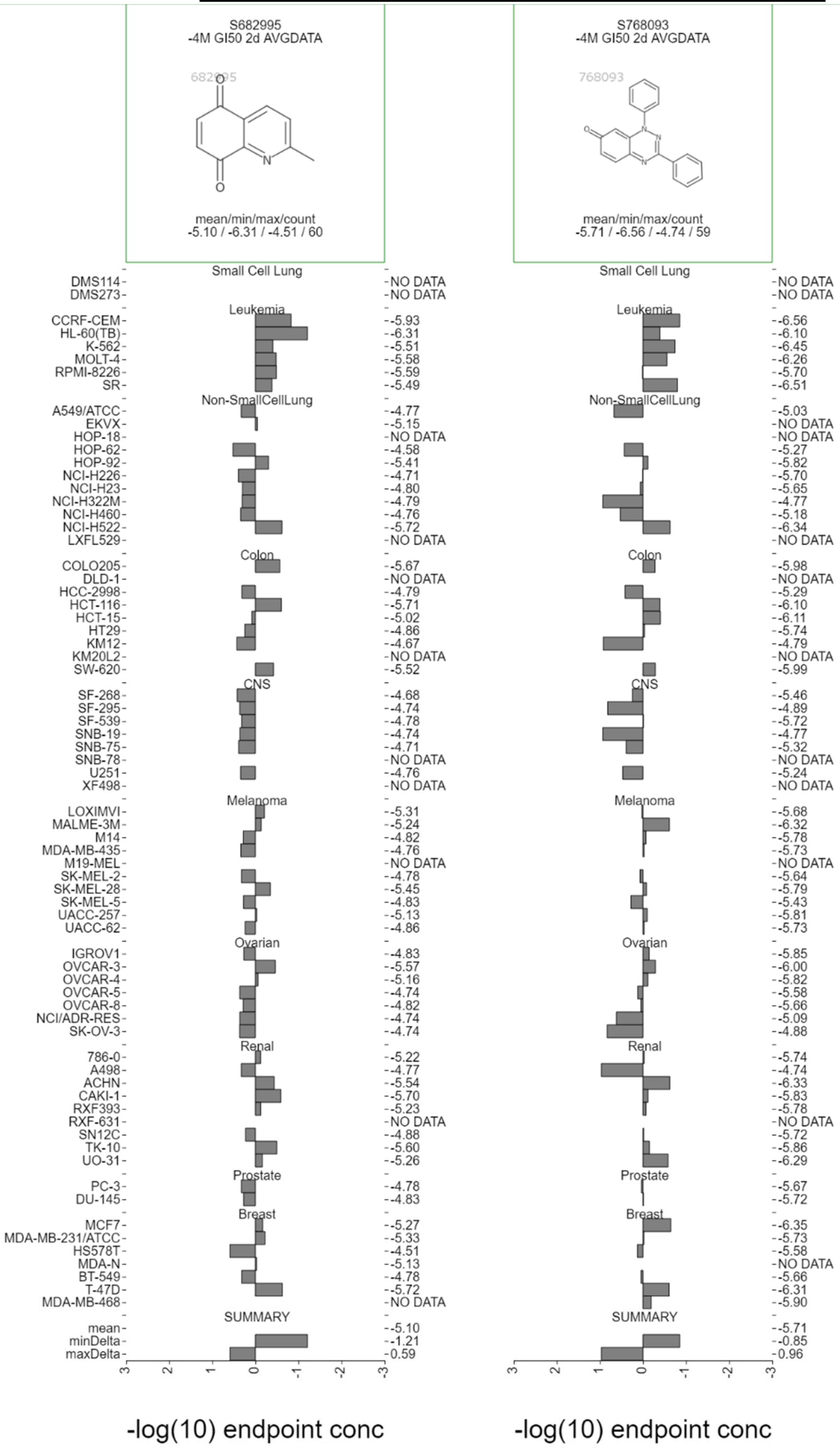

Figure S55

COMPARE Analysis of **9b** (NSC682995) to Pleurotin (NSC401005)

---SEED---
NSC:S682995 Endpoint:GI50 ExpId:AVGDATA logHiConc:-4.0
Id: 4367529 Count Experiments: 2
---TARGET---
NSC:S401005 Endpoint:GI50 ExpId:AVGDATA logHiConc:-4.0
Id: 243481 Count Experiments: 2

Correlation: 0.81
JobId: 8272072227347731043

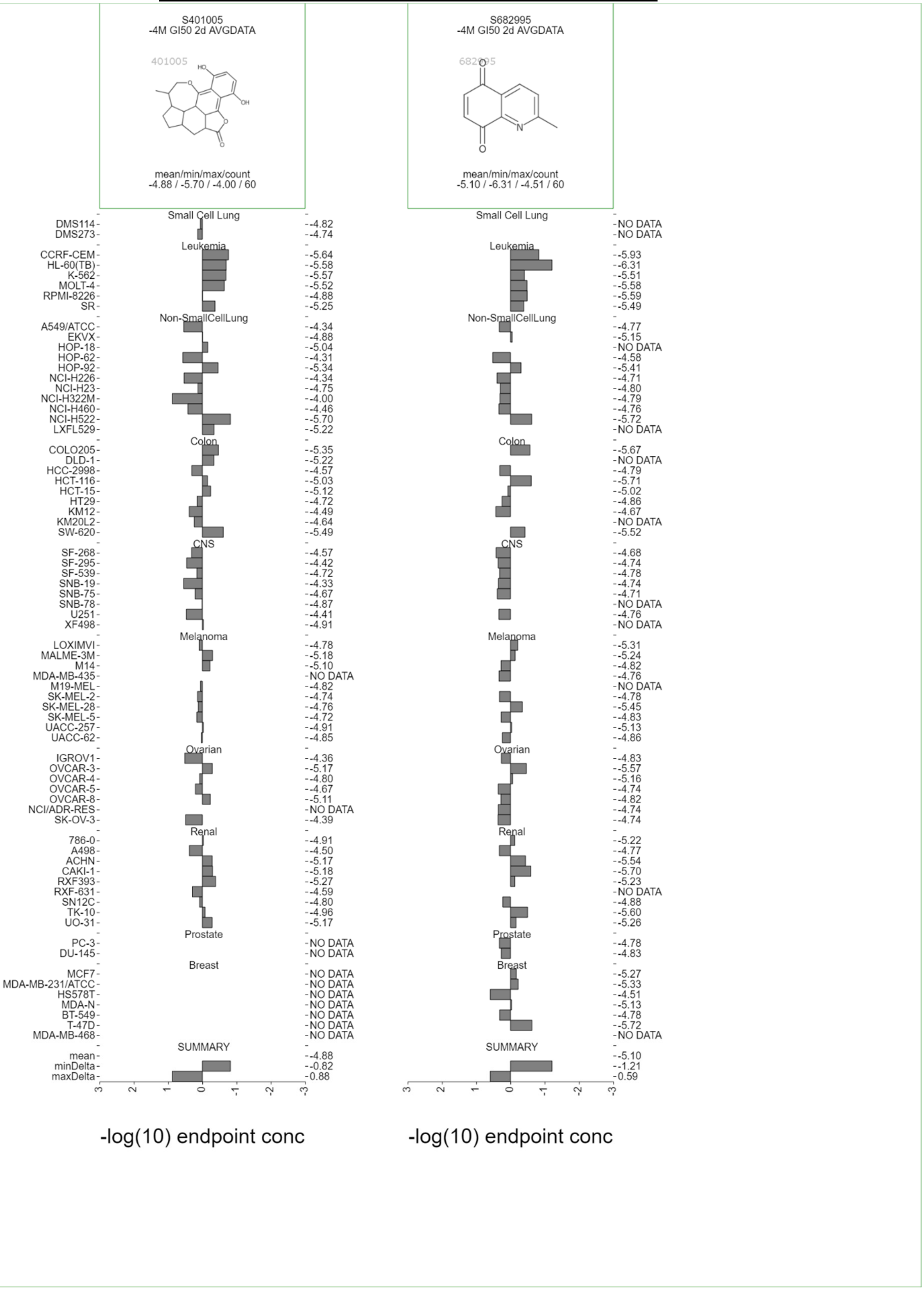

Figure S56

COMPARE Analysis of **5a** (NSC768093) to MMB analogue (NSC801078)

---SEED---
NSC:S768093 Endpoint:GI50 ExpId:AVGDATA logHiConc:-4.0
Id: 253700 Count Experiments: 2
---TARGET---
NSC:S801078 Endpoint:GI50 ExpId:AVGDATA logHiConc:-4.0
Id: 133157 Count Experiments: 1

Correlation: 0.86
JobId: 8801576231309923097

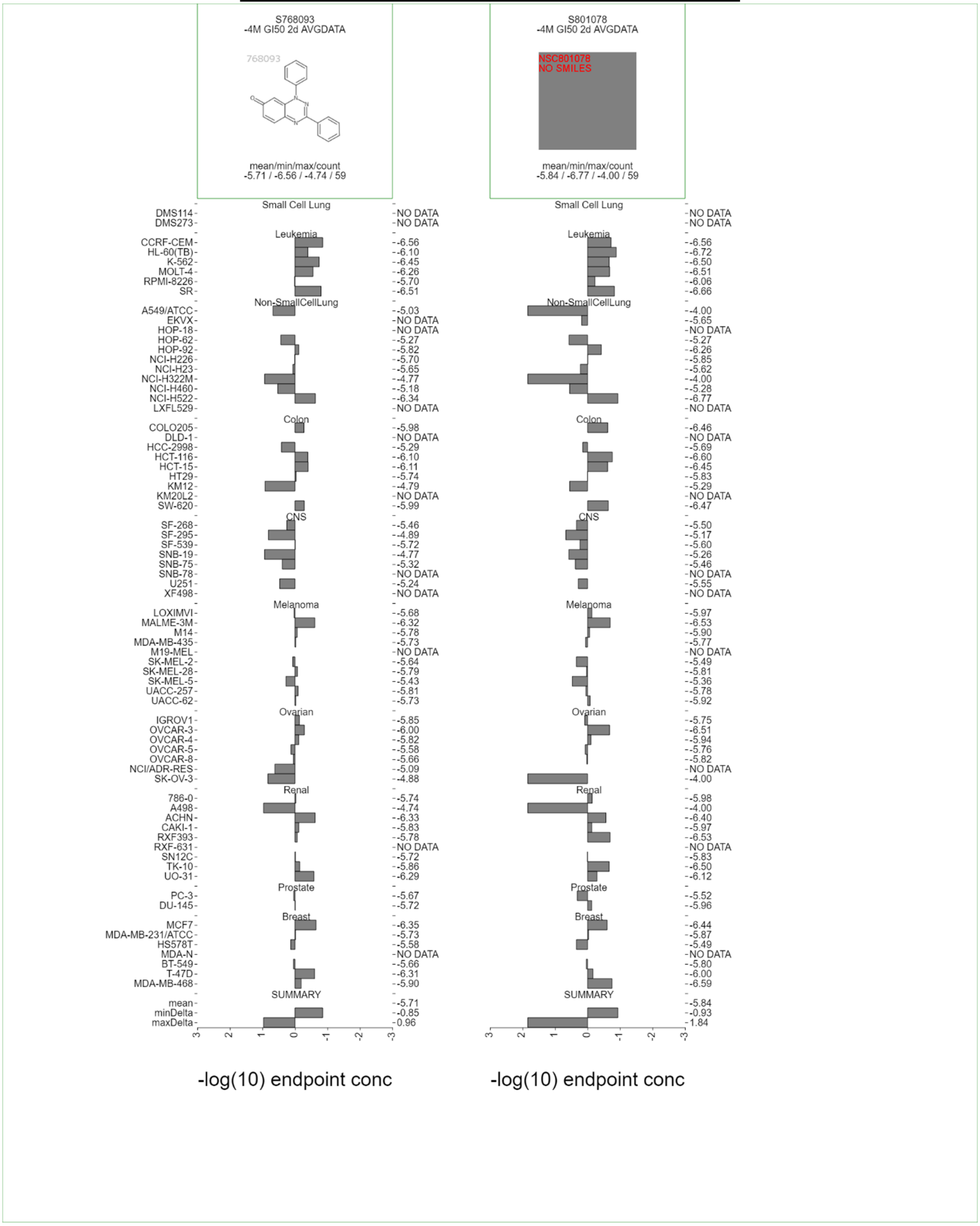

COMPARE Analysis of MMB analogue (NSC801078) to Pleurotin (NSC401005)

---SEED---
NSC:S801078 Endpoint:GI50 ExpId:AVGDATA logHiConc:-4.0
Id: 133157 Count Experiments: 1
---TARGET---
NSC:S401005 Endpoint:GI50 ExpId:AVGDATA logHiConc:-4.0
Id: 243481 Count Experiments: 2

Correlation: 0.82
JobId: 866925007213814722

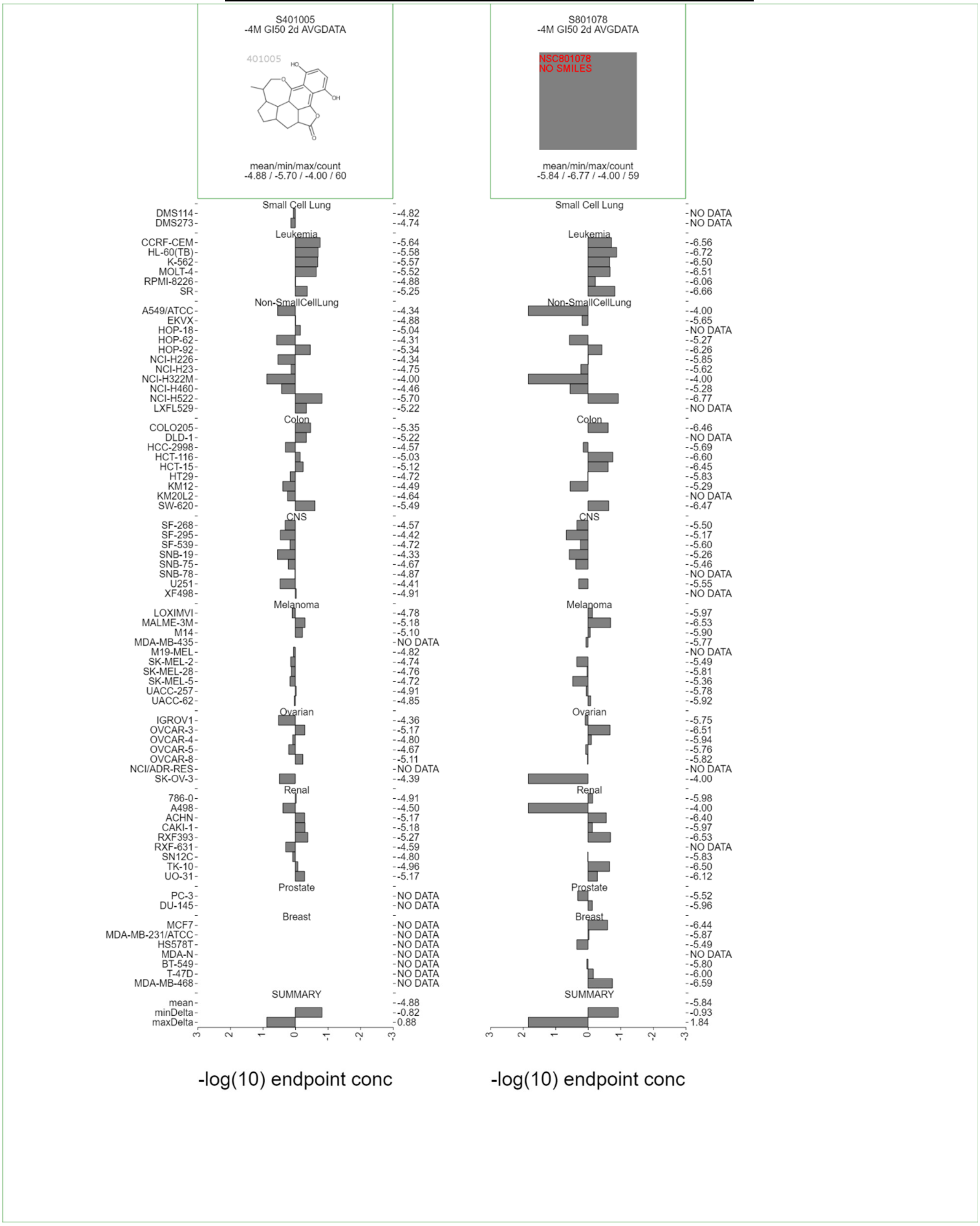

Figure S58

COMPARE Analysis of NQO1 to **19** (NSC409304)

---SEED---
MoltId:MT22 Genecard:NQO1 TargetSet:MOLTID_MT_SERIES
---TARGET---
NSC:S409304 Endpoint:GI50 ExpId:AVGDATA logHiConc:-4.0
Id: 251495 Count Experiments: 1

Correlation: 0.67
JobId: 7364063256090692400

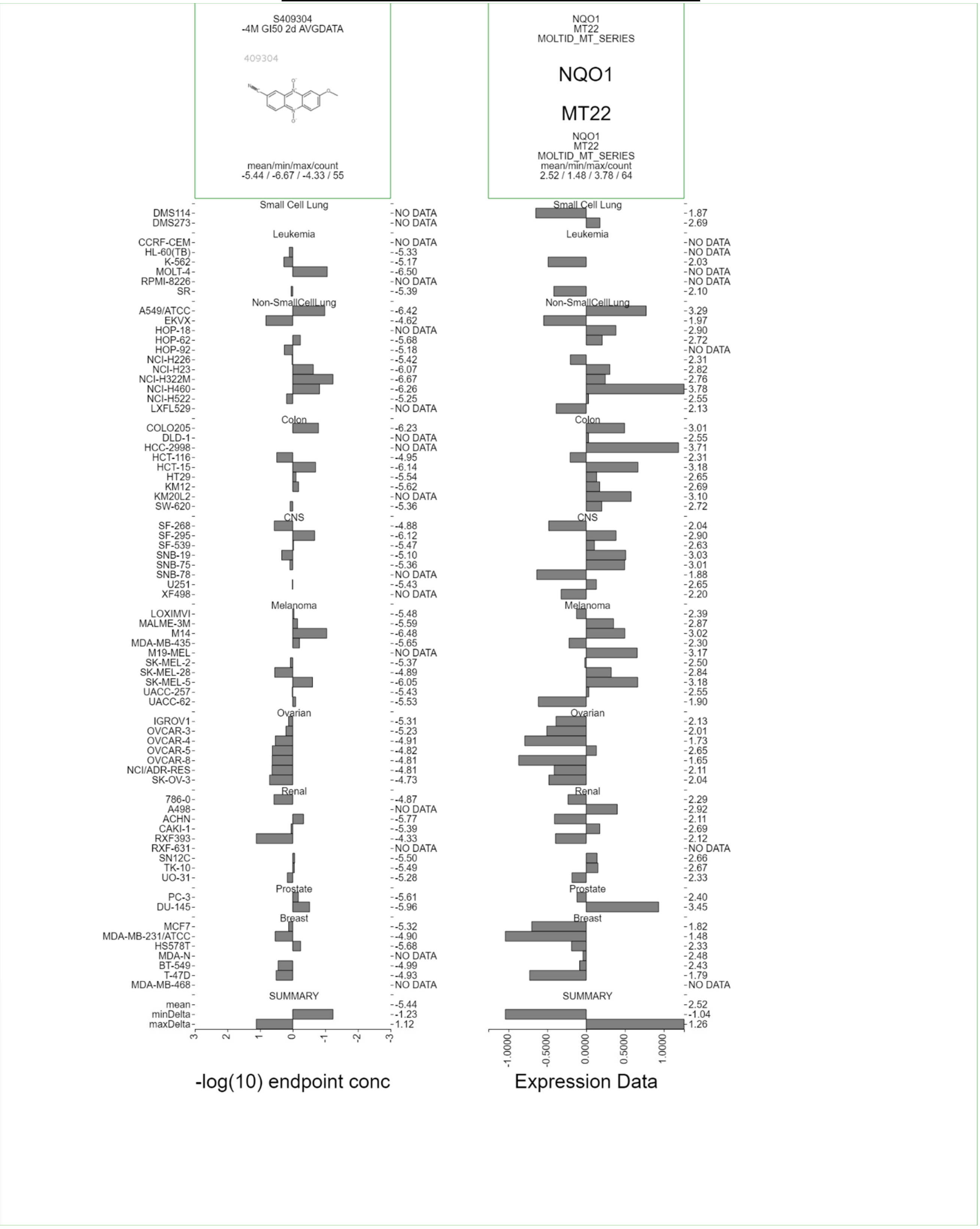

Figure S59

COMPARE Analysis of **19** (NSC409304) to DPIQ (NSC753790)

---SEED---
NSC:S409304 Endpoint:GI50 ExpId:AVGDATA logHiConc:-4.0
Id: 251495 Count Experiments: 1
---TARGET---
NSC:S753790 Endpoint:GI50 ExpId:AVGDATA logHiConc:-4.0
Id: 373263 Count Experiments: 2

Correlation: 0.51
JobId: 7364063256090692400

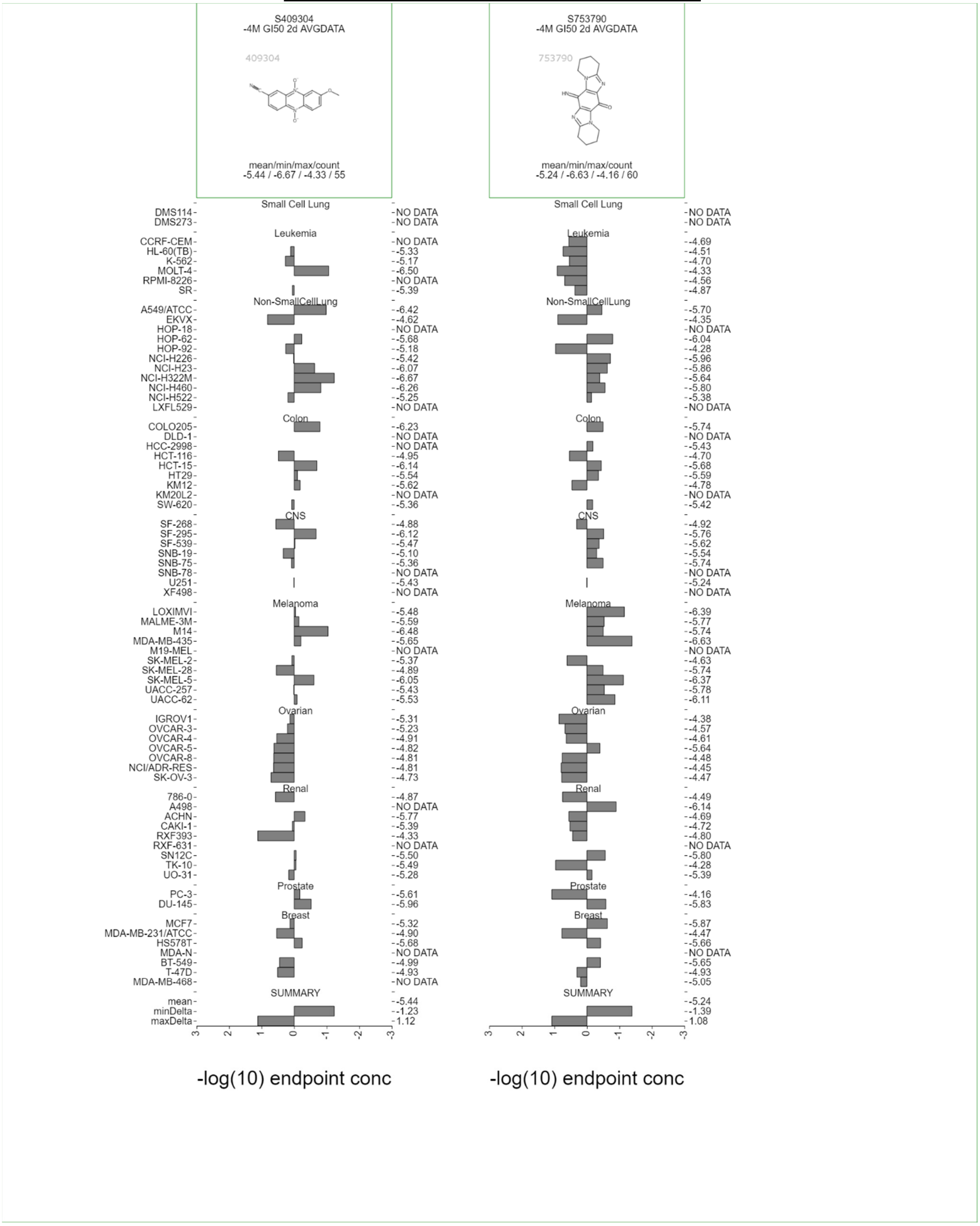

Figure S60

COMPARE Analysis of NQO1 to **20** (NSC2785)

---SEED---

MoltId:MT22 Genecard:NQO1 TargetSet:MOLTID_MT_SERIES

---TARGET---

NSC:S2785 Endpoint:GI50 ExpId:AVGDATA logHiConc:-4.0

Id: 71580 Count Experiments: 1

Correlation: 0.66

JobId: 1356468010017973524

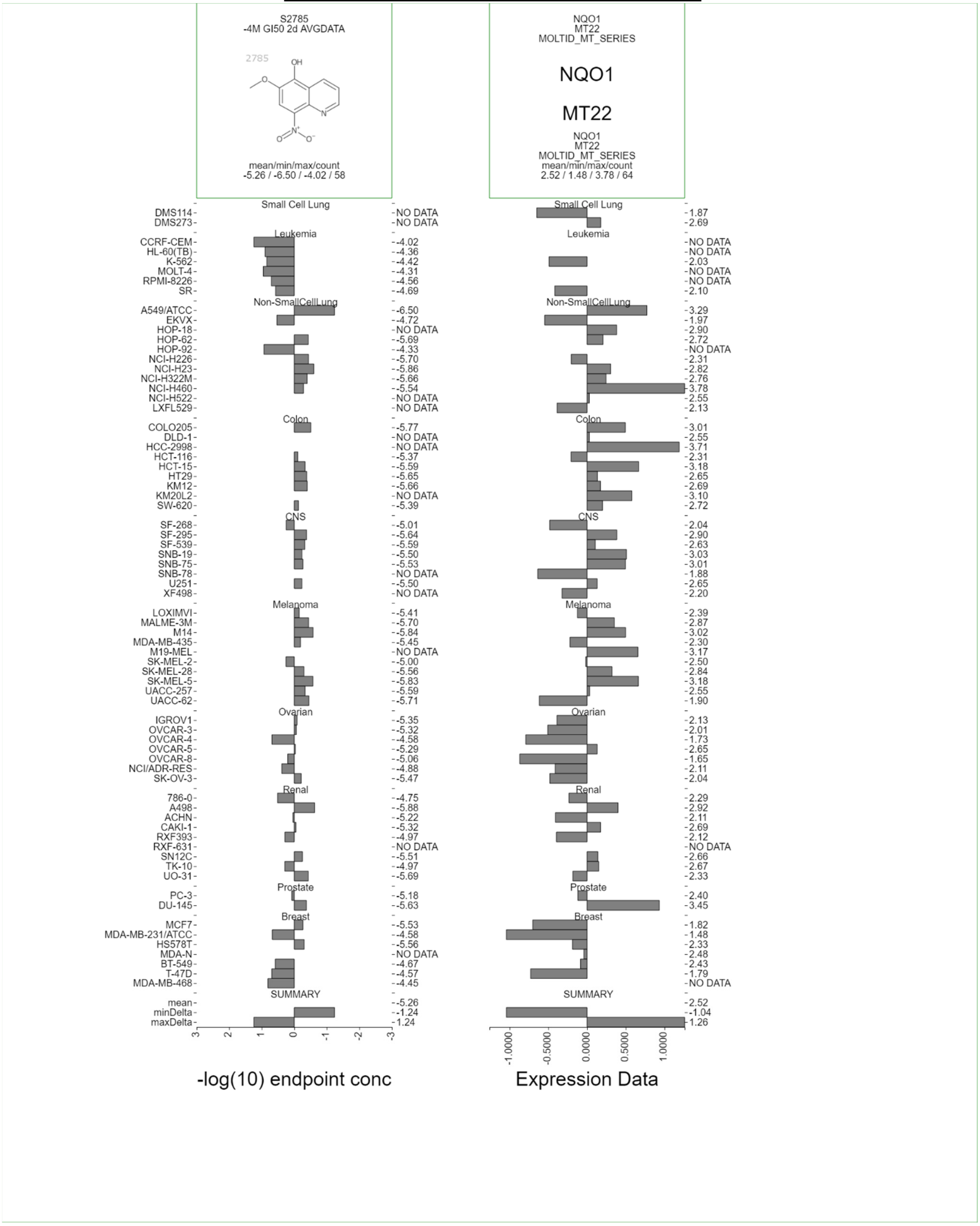

Figure S61

COMPARE Analysis of **20** (NSC2785) to DPIQ (NSC753790)

---SEED---
NSC:S2785 Endpoint:GI50 ExpId:AVGDATA logHiConc:-4.0
Id: 71580 Count Experiments: 1
---TARGET---
NSC:S753790 Endpoint:GI50 ExpId:AVGDATA logHiConc:-4.0
Id: 373263 Count Experiments: 2

Correlation: 0.69
JobId: 8298870972283168025

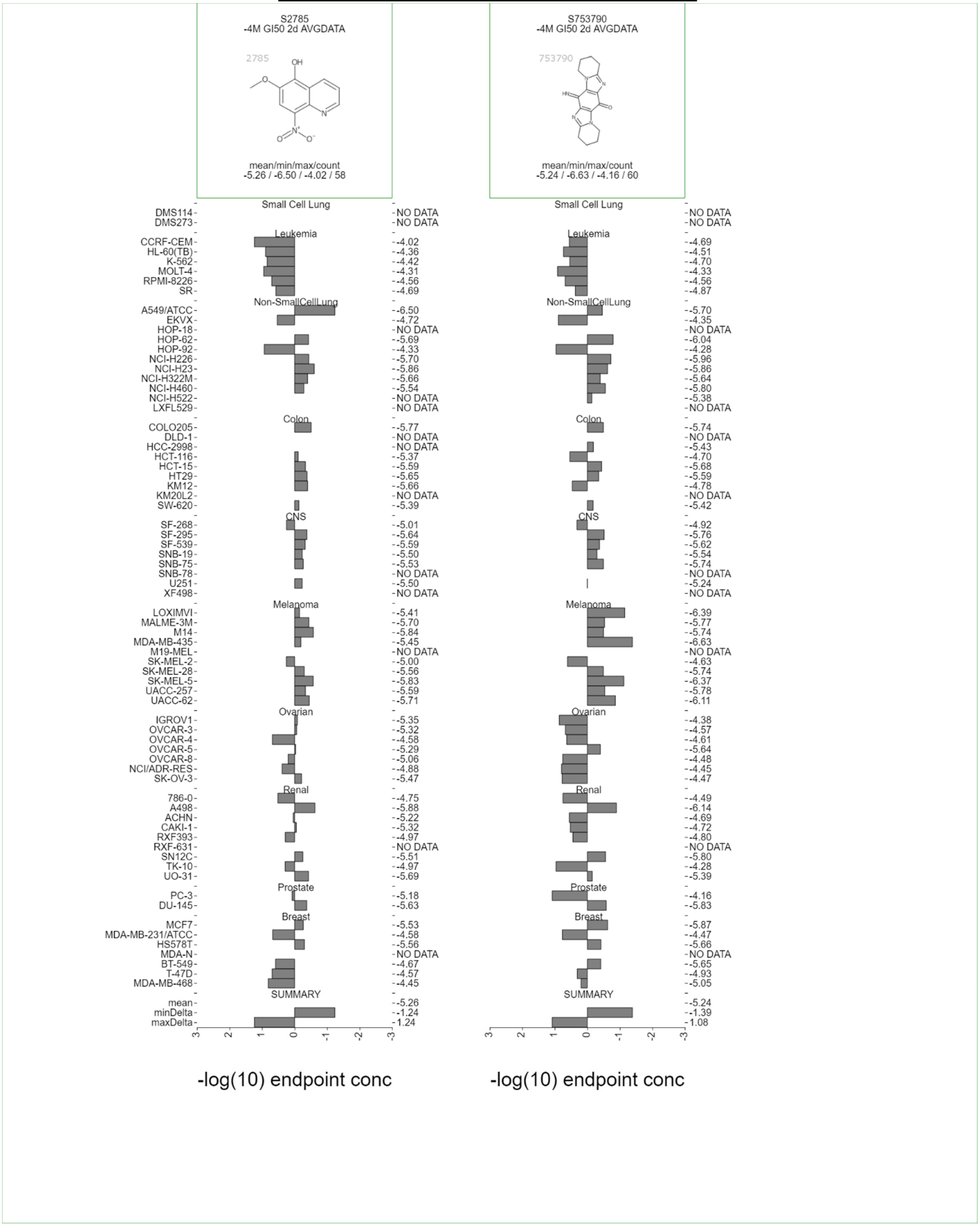

Figure S62

COMPARE Analysis of NQO1 to **21a** (NSC783640)

---SEED---

MoltId:MT22 Genecard:NQO1 TargetSet:MOLTID_MT_SERIES

---TARGET---

NSC:S783640 Endpoint:GI50 ExpId:AVGDATA logHiConc:-4.0

Id: 72655 Count Experiments: 2

Correlation: 0.64

JobId: 7715194222435053083

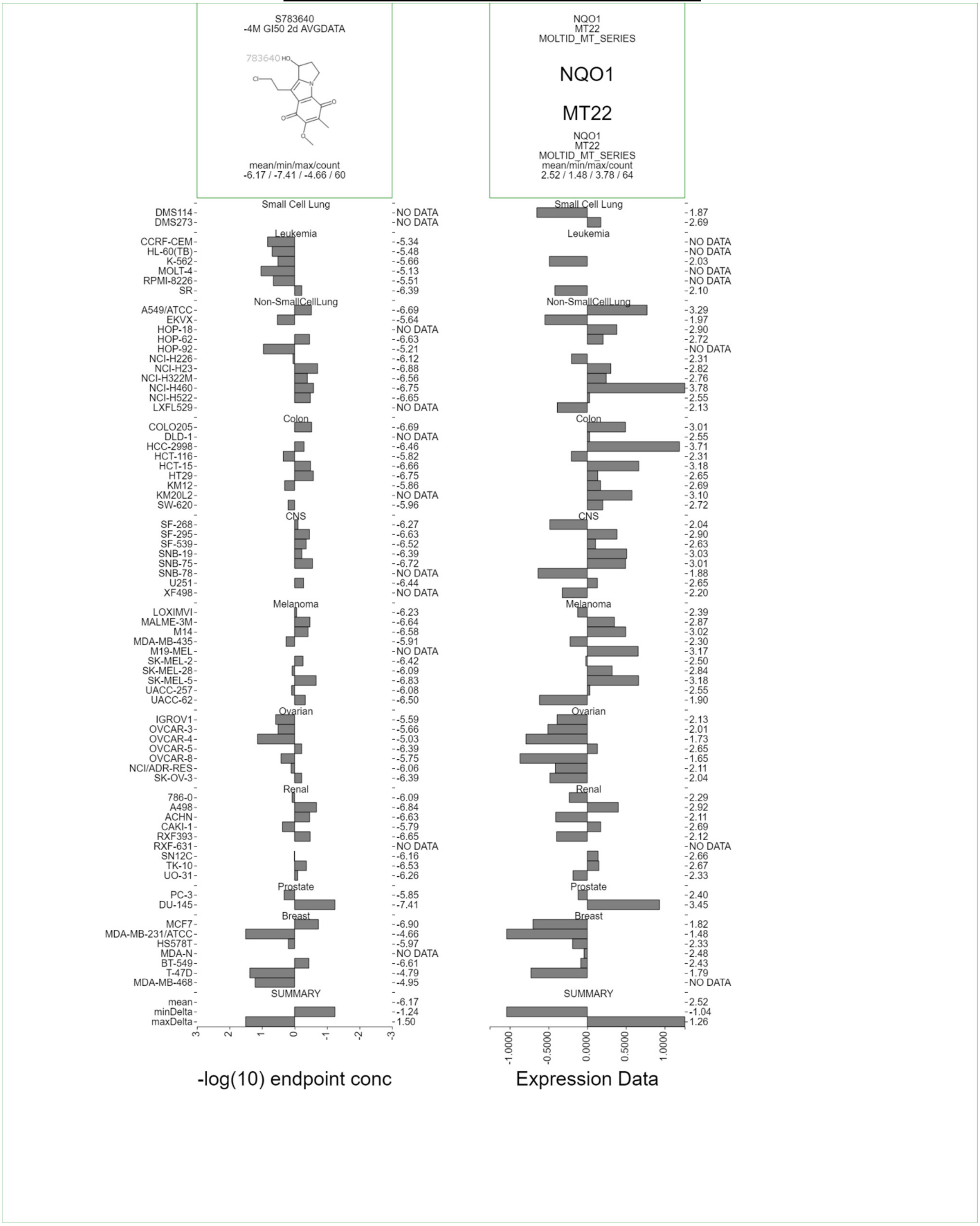

Figure S63

COMPARE Analysis of **21a** (NSC783640) to DPIQ (NSC753790)

---SEED---
NSC:S783640 Endpoint:GI50 ExpId:AVGDATA logHiConc:-4.0
Id: 72655 Count Experiments: 2
---TARGET---
NSC:S753790 Endpoint:GI50 ExpId:AVGDATA logHiConc:-4.0
Id: 373263 Count Experiments: 2

Correlation: 0.65
JobId: 7715194222435053083

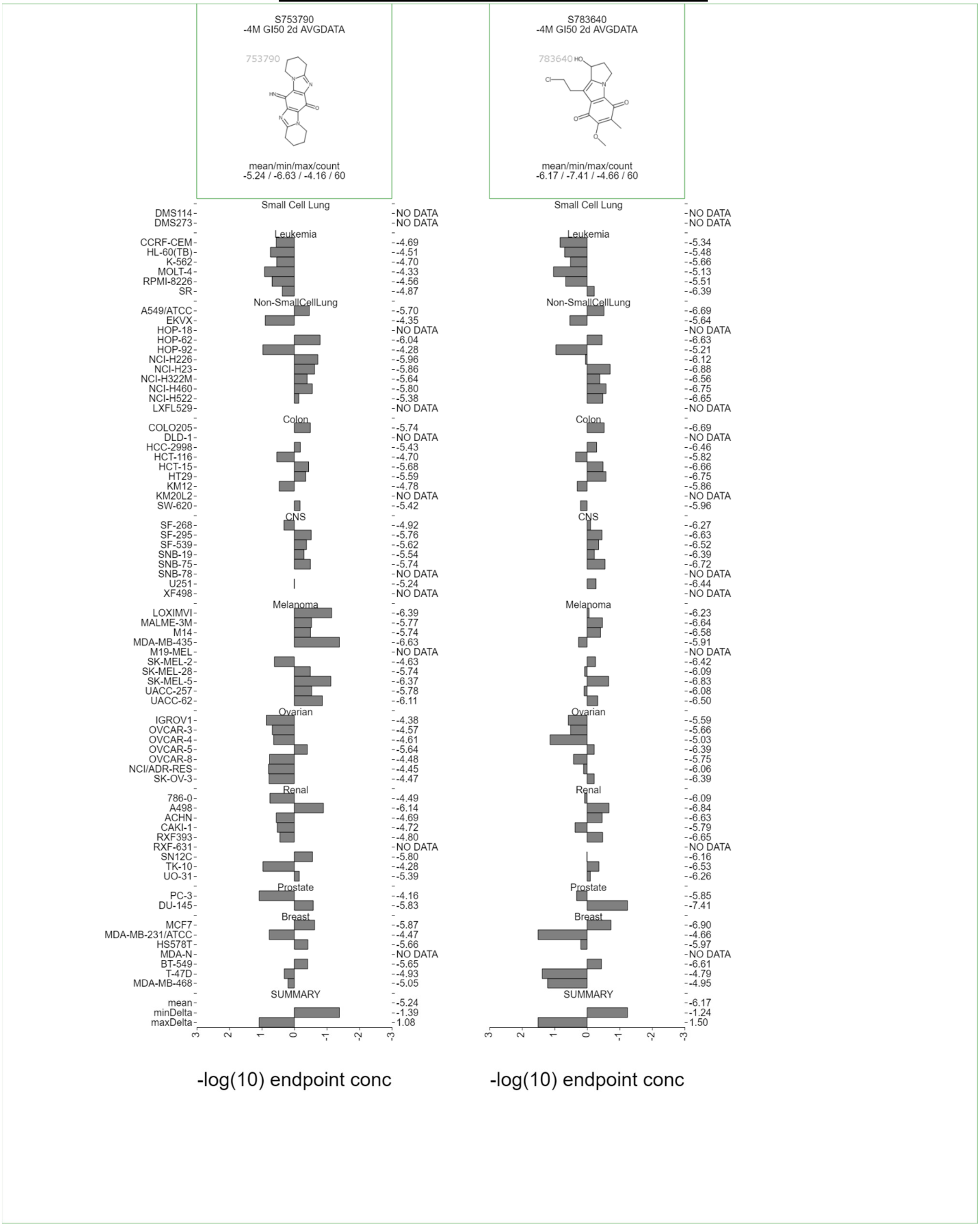

Figure S64

COMPARE Analysis of NQO1 to **21b** (NSC783641)

---SEED---
MoltId:MT22 Genecard:NQO1 TargetSet:MOLTID_MT_SERIES
---TARGET---
NSC:S783641 Endpoint:GI50 ExpId:AVGDATA logHiConc:-4.0
Id: 314263 Count Experiments: 2

Correlation: 0.66
JobId: 3646587695121137667

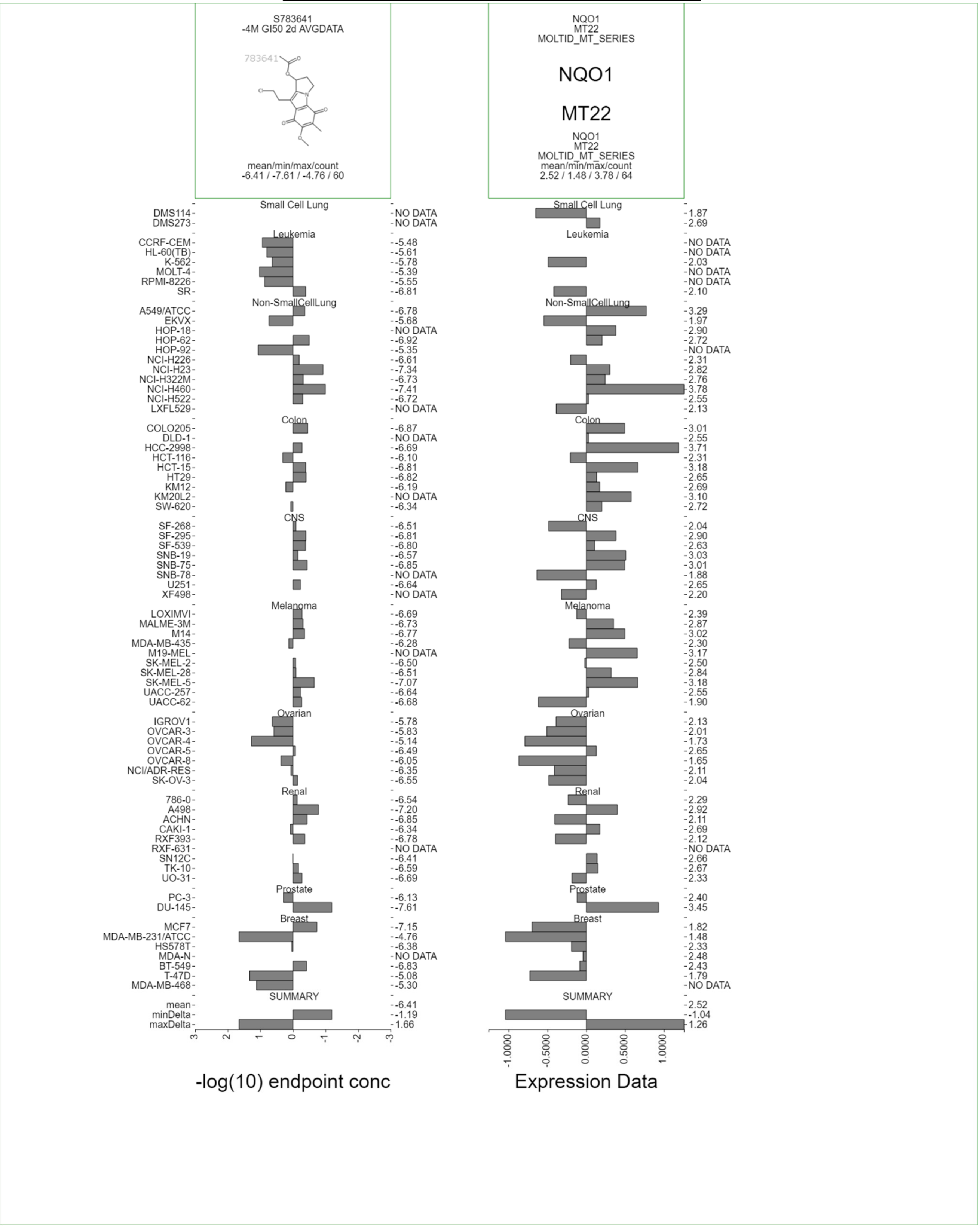

Figure S65

COMPARE Analysis of **21b** (NSC783641) to DPIQ (NSC753790)

---SEED---
NSC:S783641 Endpoint:GI50 ExpId:AVGDATA logHiConc:-4.0
Id: 314263 Count Experiments: 2
---TARGET---
NSC:S753790 Endpoint:GI50 ExpId:AVGDATA logHiConc:-4.0
Id: 373263 Count Experiments: 2

Correlation: 0.66
JobId: 3646587695121137667

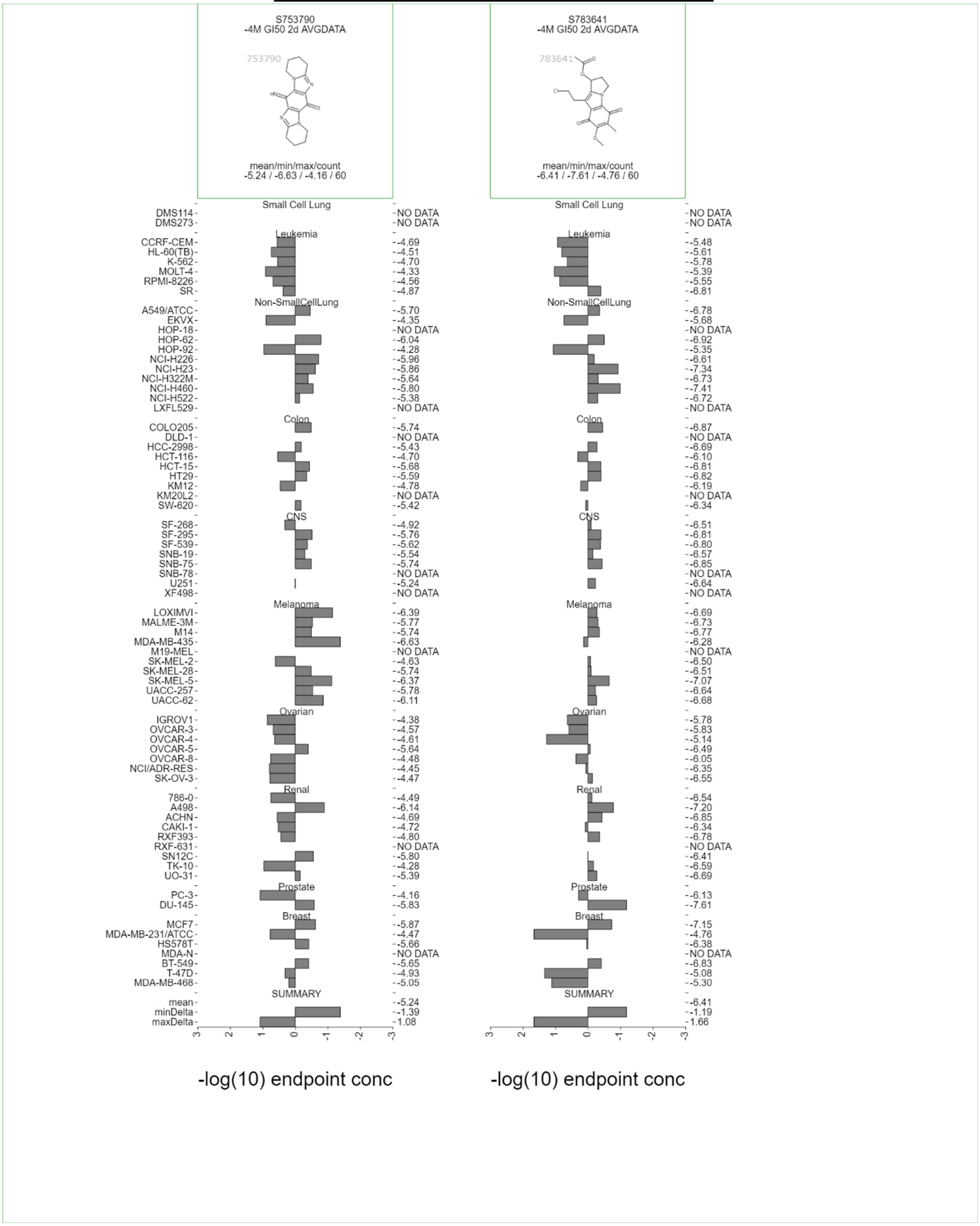

Figure S66

COMPARE Analysis of NQO1 to **5a** (NSC768093)

---SEED---

MoltId:MT22 Genecard:NQO1 TargetSet:MOLTID_MT_SERIES

---TARGET---

NSC:S768093 Endpoint:GI50 ExpId:AVGDATA logHiConc:-4.0

Id: 253700 Count Experiments: 2

Correlation: -0.37

JobId: 2580018557142257876

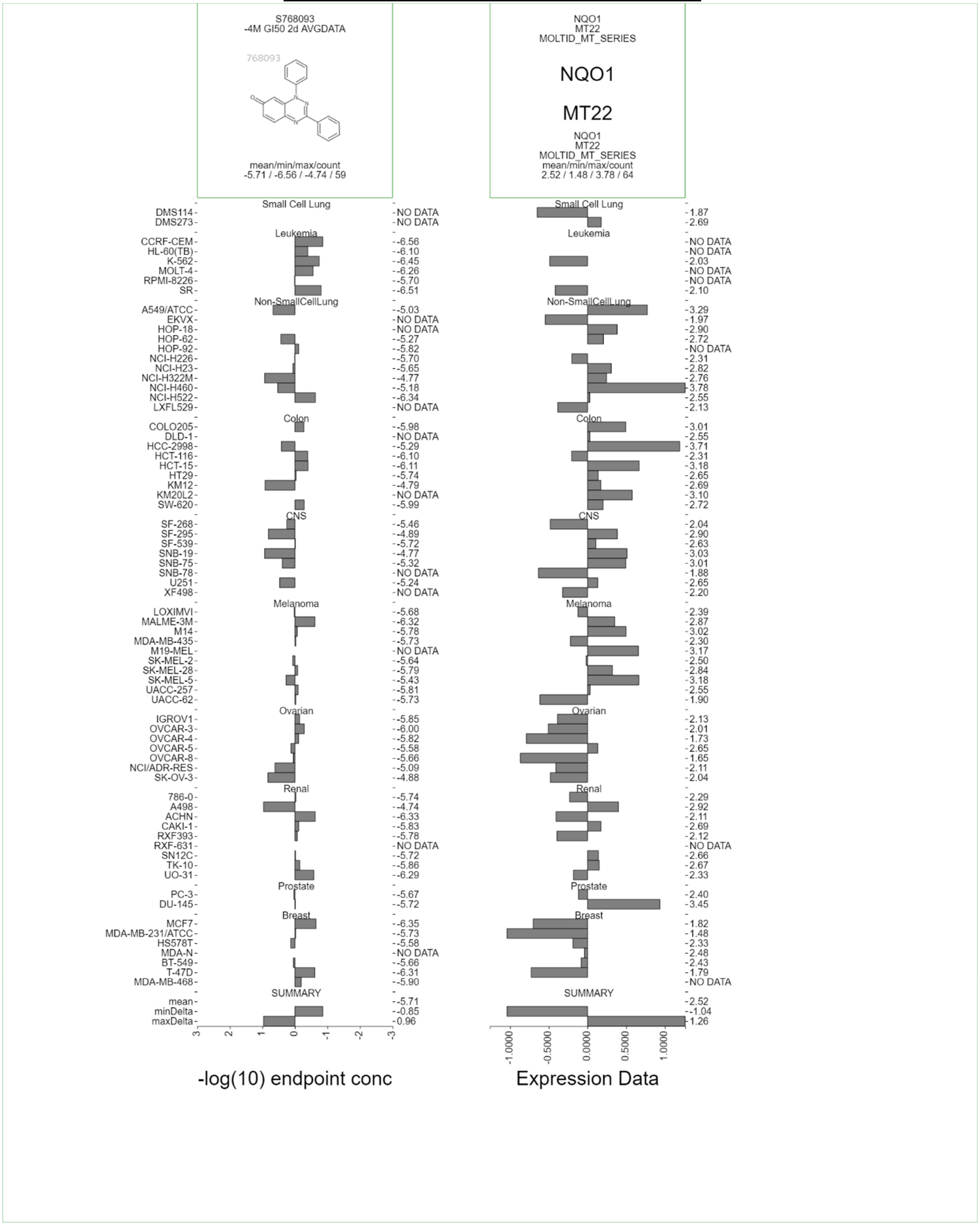

Figure S67

COMPARE Analysis of NQO1 to **5b** (NSC780813)

---SEED---

MoltId:MT22 Genecard:NQO1 TargetSet:MOLTID_MT_SERIES

---TARGET---

NSC:S780813 Endpoint:GI50 ExpId:AVGDATA logHiConc:-4.0

Id: 314185 Count Experiments: 1

Correlation: -0.30

JobId: 7799368897062004237

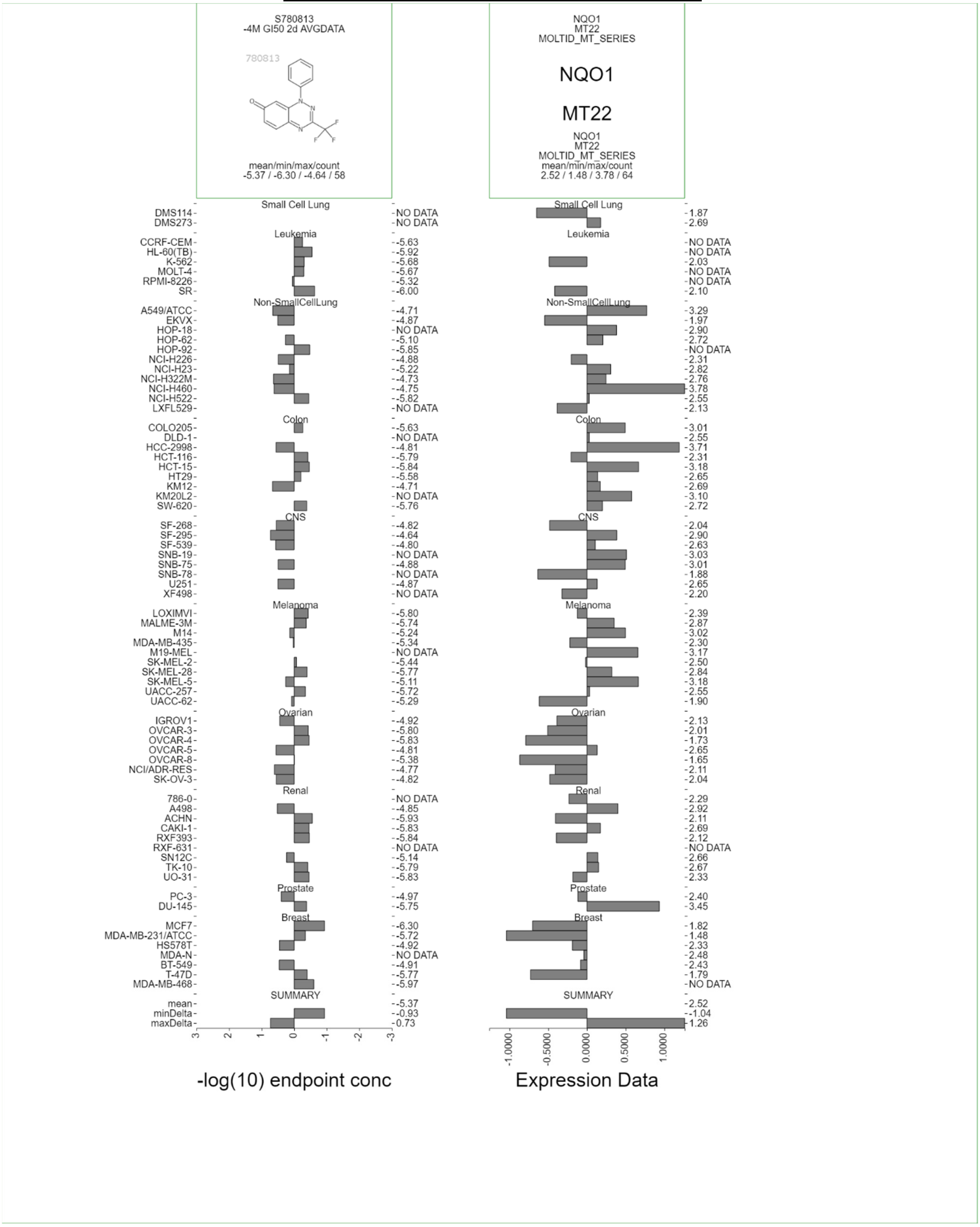

Figure S68

COMPARE Analysis of NQO1 to Pleurotin (NSC401005)

---SEED---
MoltId:MT22 Genecard:NQO1 TargetSet:MOLTID_MT_SERIES
---TARGET---
NSC:S401005 Endpoint:GI50 ExpId:AVGDATA logHiConc:-4.0
Id: 243481 Count Experiments: 2

Correlation: -0.27
JobId: 5196849035899921588

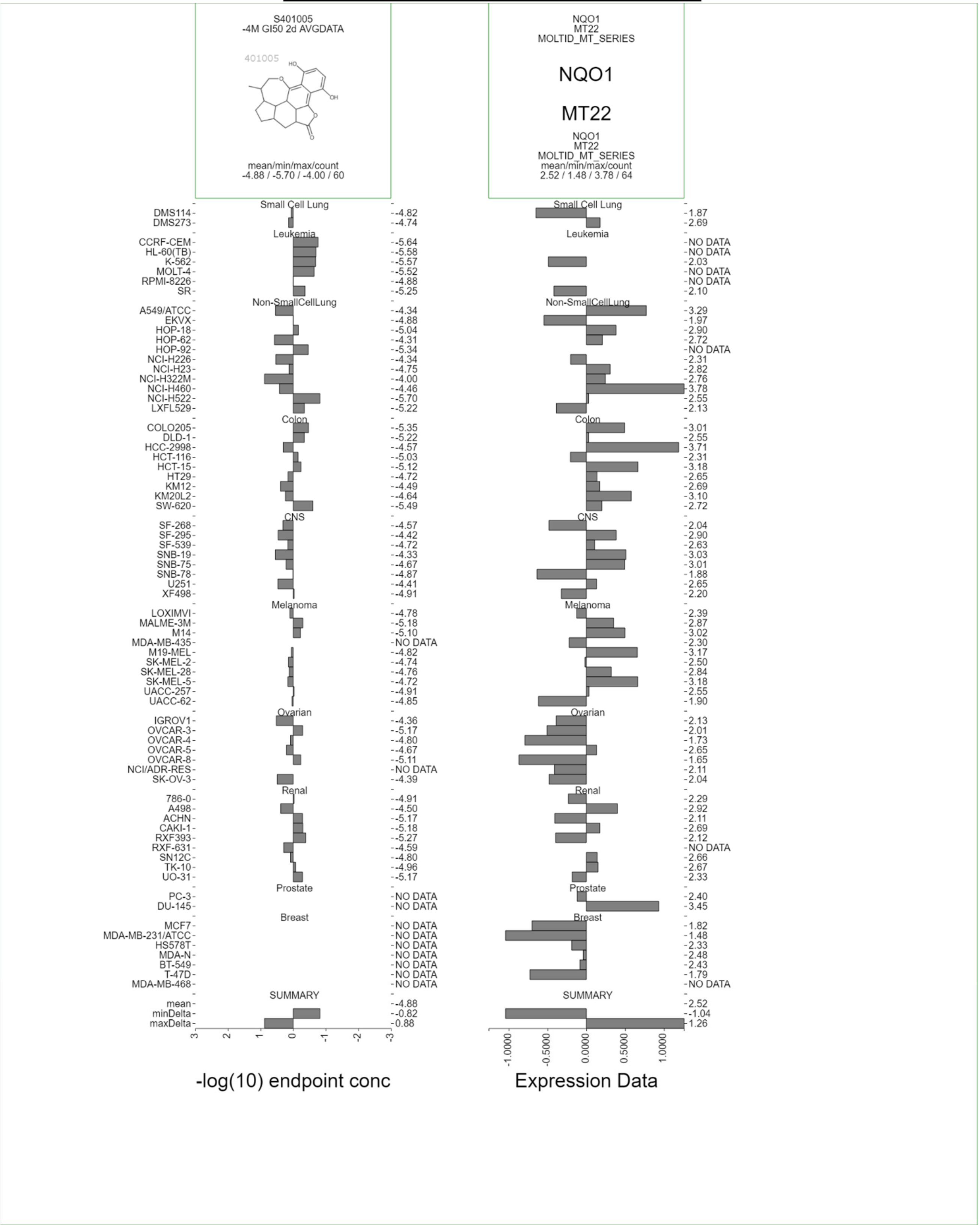

Figure S69

COMPARE Analysis of NQO1 to **14b** (NSC668844)

---SEED---
MoltId:MT22 Genecard:NQO1 TargetSet:MOLTID_MT_SERIES
---TARGET---
NSC:S668844 Endpoint:GI50 ExpId:AVGDATA logHiConc:-4.0
Id: 426305 Count Experiments: 3

Correlation: -0.48
JobId: 3829946830593806600

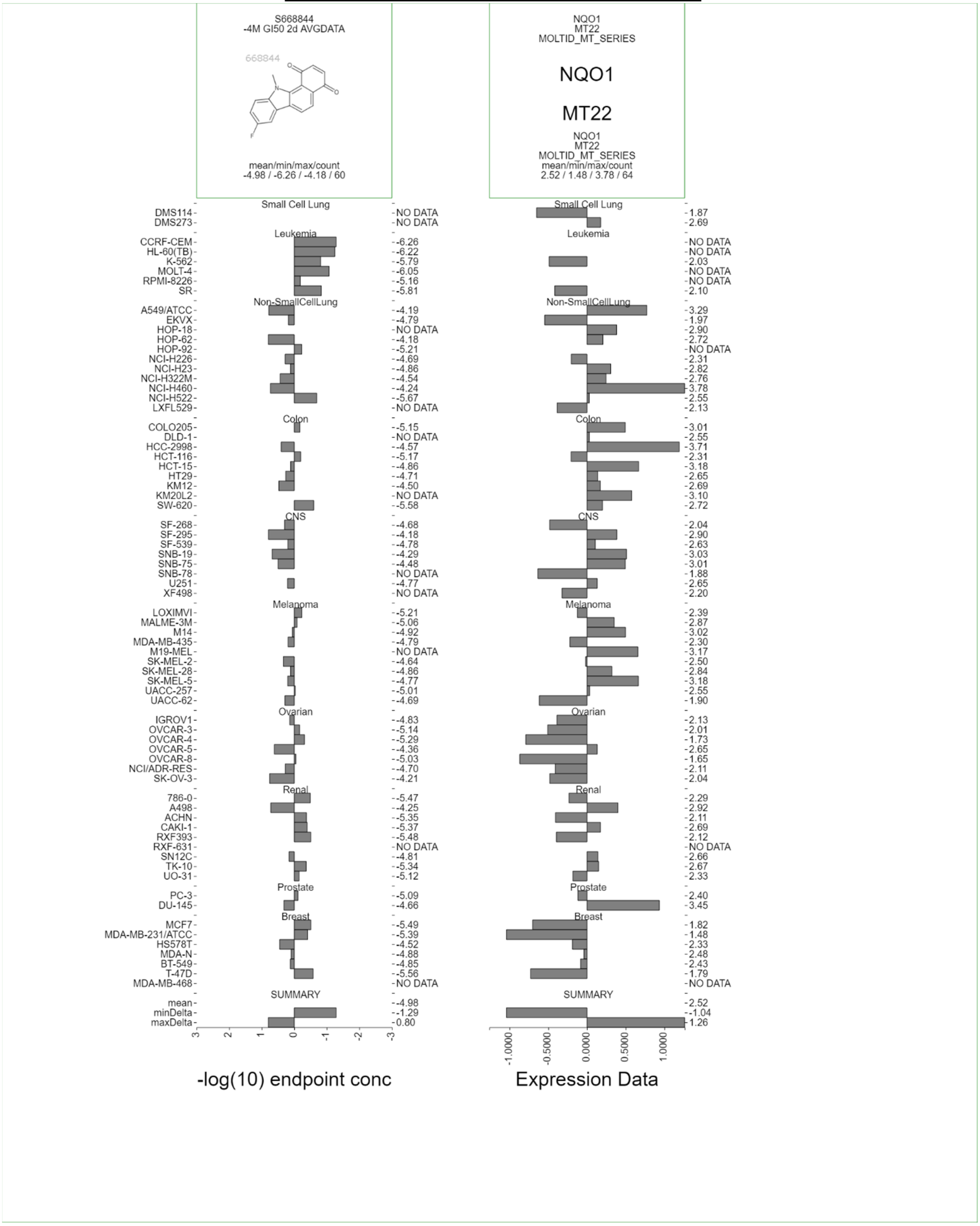

Figure S70

COMPARE Analysis of NQO1 to Kalafungin (NSC137443)

---SEED---
MoltId:MT22 Genecard:NQO1 TargetSet:MOLTID_MT_SERIES
---TARGET---
NSC:S137443 Endpoint:GI50 ExpId:AVGDATA logHiConc:-4.0
Id: 71608 Count Experiments: 2

Correlation: -0.27
JobId: 7682632898986498469

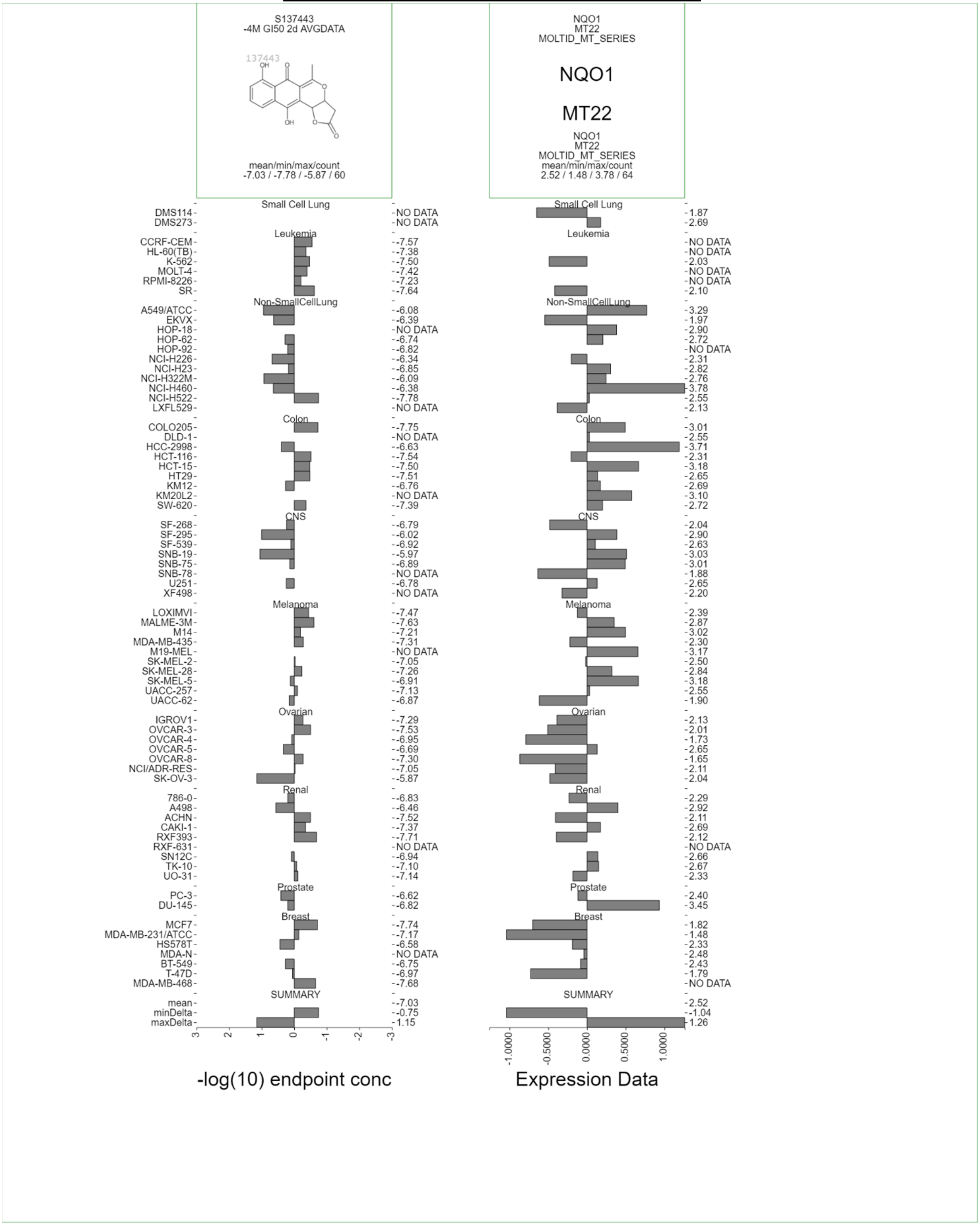

Figure S71

COMPARE Analysis of NQO1 to Discorhabdin C (NSC626162)

---SEED---
MoltId:MT22 Genecard:NQO1 TargetSet:MOLTID_MT_SERIES
---TARGET---
NSC:S626162 Endpoint:GI50 ExpId:AVGDATA logHiConc:-4.0
Id: 421973 Count Experiments: 5

Correlation: -0.29
JobId: 3262975264366953011

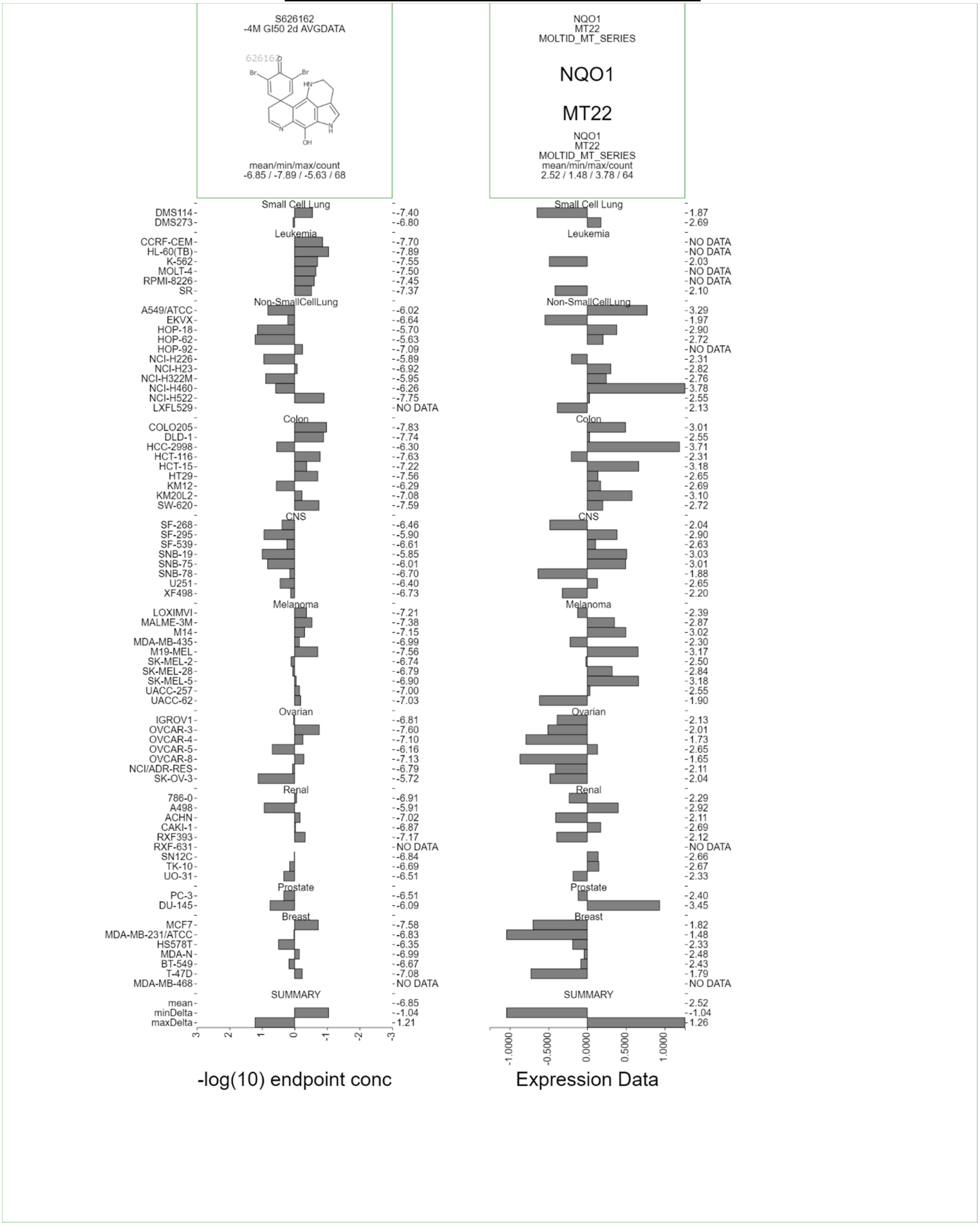

Figure S72

COMPARE Analysis of TrxR to **5a** (NSC768093)

---SEED---

MoltId:MT143 Genecard:TXNRD1 TargetSet:MOLTID_MT_SERIES

---TARGET---

NSC:S768093 Endpoint:GI50 ExpId:AVGDATA logHiConc:-4.0

Id: 253700 Count Experiments: 2

Correlation: -0.24

JobId: 766082888249896201

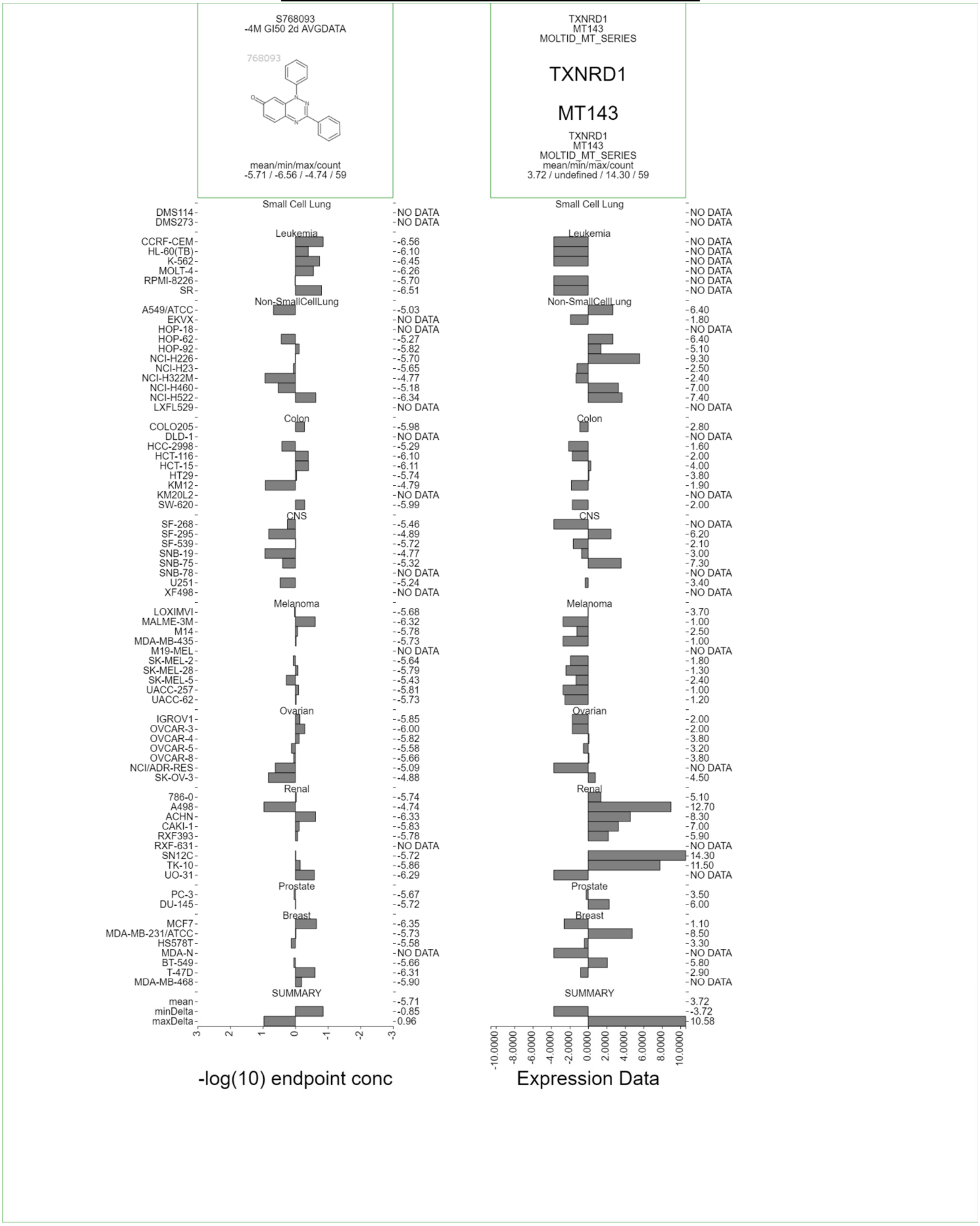

Figure S73

COMPARE Analysis of TrxR to **5b** (NSC780813)

---SEED---
MoltId:MT143 Genecard:TXNRD1 TargetSet:MOLTID_MT_SERIES
---TARGET---
NSC:S780813 Endpoint:GI50 ExpId:AVGDATA logHiConc:-4.0
Id: 314185 Count Experiments: 1

Correlation: -0.12
JobId: 4305186664534873894

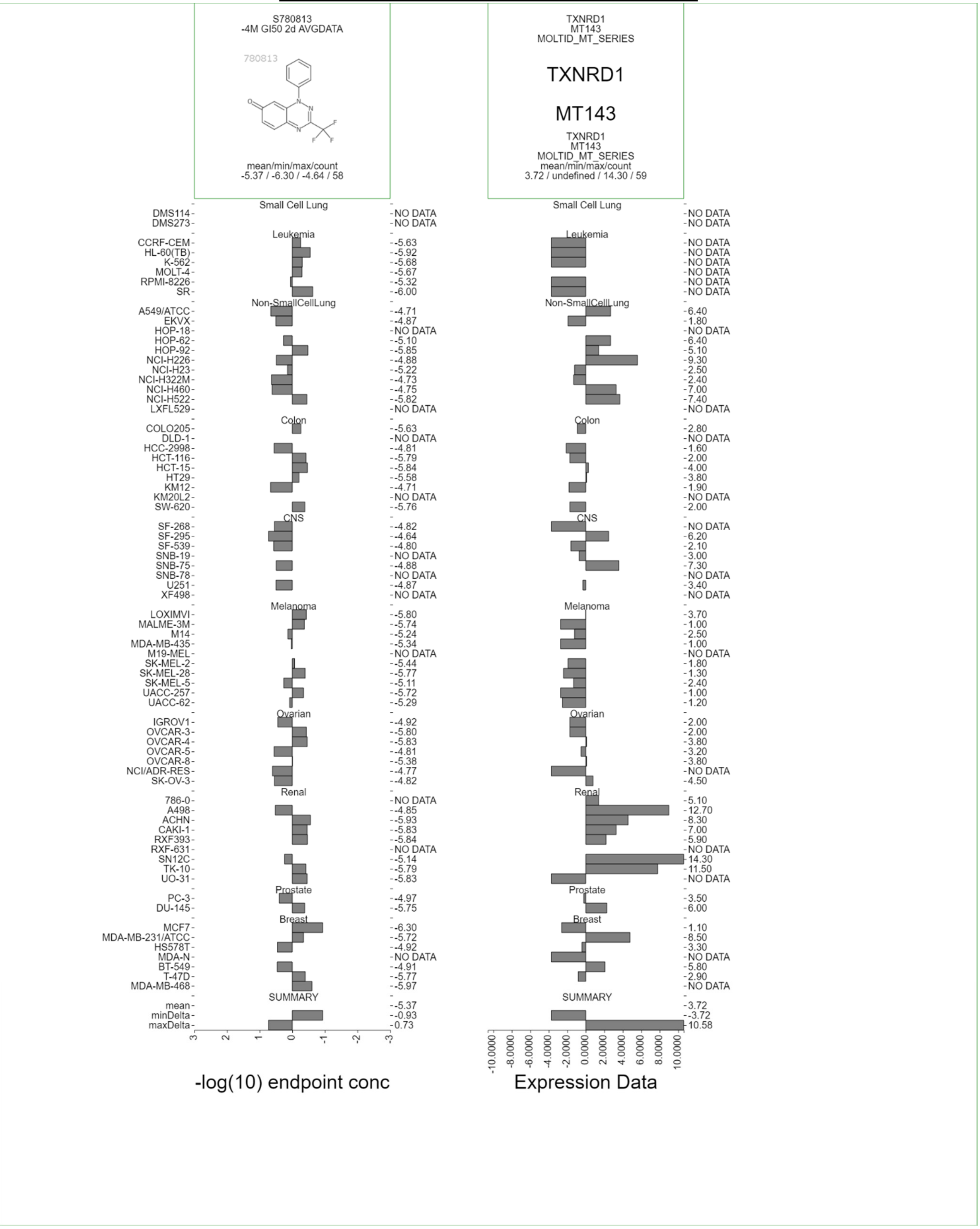

Figure S74

COMPARE Analysis of TrxR to Pleurotin (NSC401005)

---SEED---
MoltId:MT143 Genecard:TXNRD1 TargetSet:MOLTID_MT_SERIES
---TARGET---
NSC:S401005 Endpoint:GI50 ExpId:AVGDATA logHiConc:-4.0
Id: 243481 Count Experiments: 2

Correlation: -0.21
JobId: 1840548376507877519

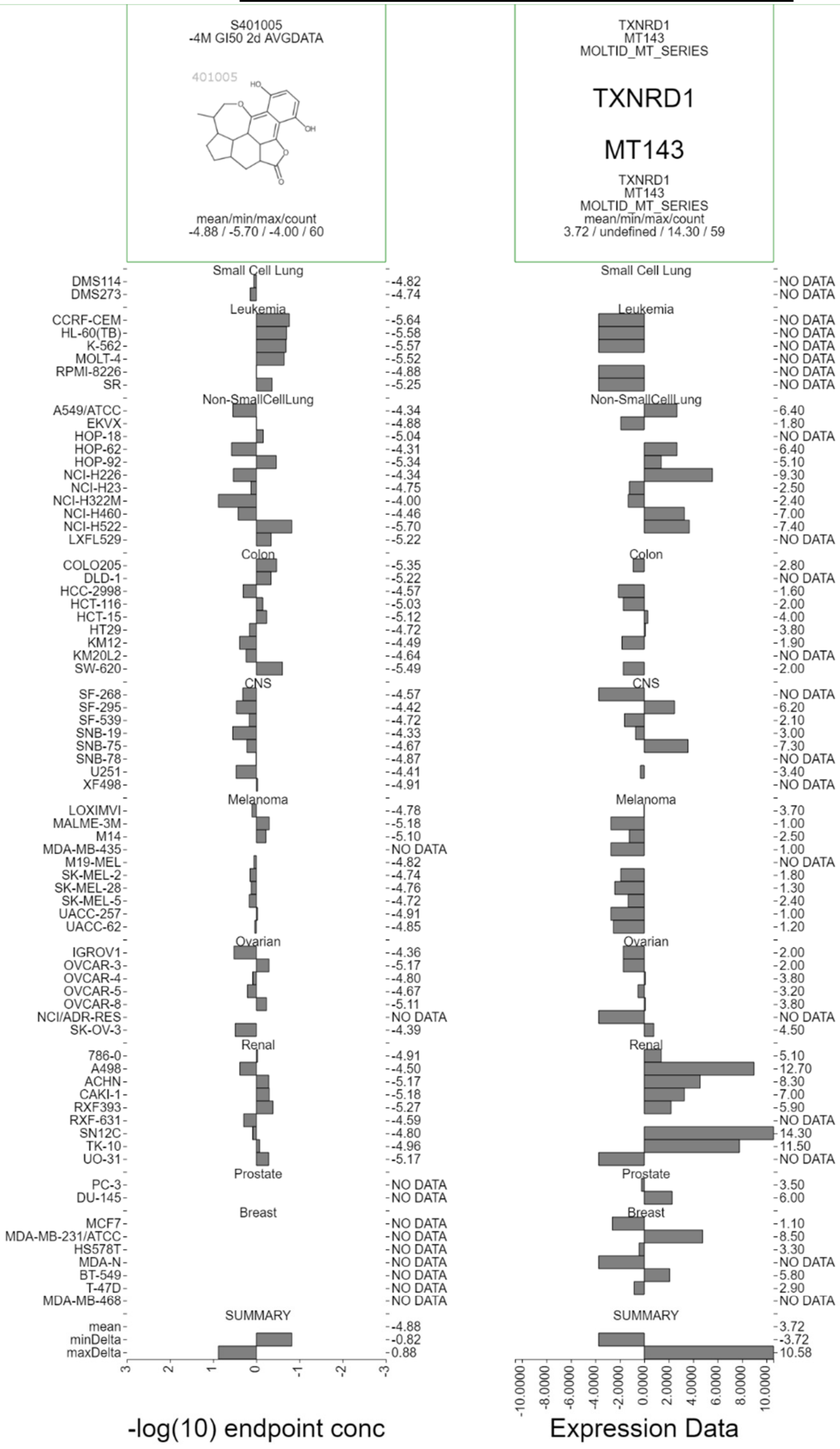

Figure S75

COMPARE Analysis of TrxR to**14b** (NSC668844)

---SEED---
MoltId:MT143 Genecard:TXNRD1 TargetSet:MOLTID_MT_SERIES
---TARGET---
NSC:S668844 Endpoint:GI50 ExpId:AVGDATA logHiConc:-4.0
Id: 426305 Count Experiments: 3

Correlation: -0.22
JobId: 1113719844959210894

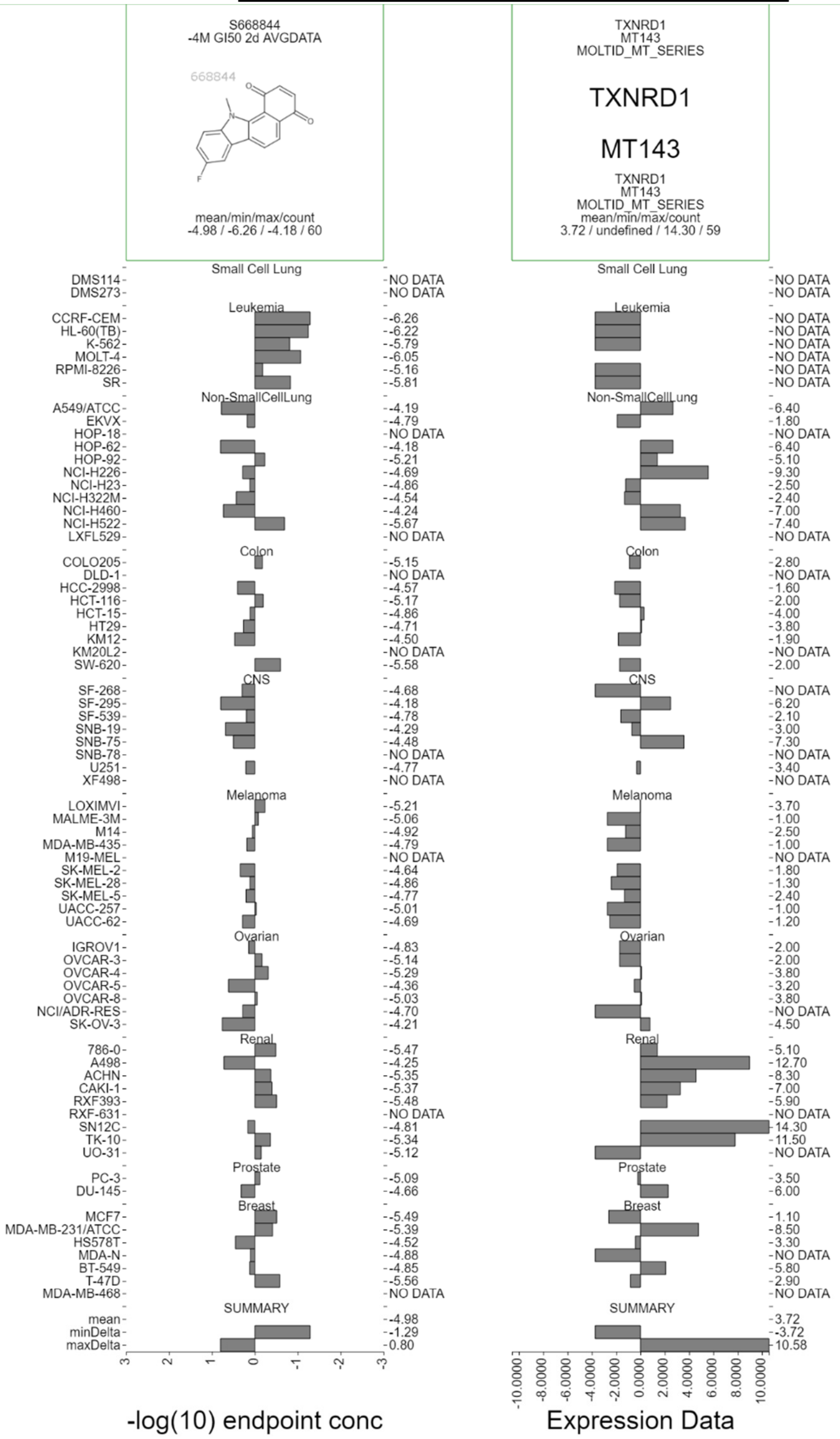

Figure S76

COMPARE Analysis of TrxR to Kalafungin (NSC137443)

---SEED---

MoltId:MT143 Genecard:TXNRD1 TargetSet:MOLTID_MT_SERIES

---TARGET---

NSC:S137443 Endpoint:GI50 ExpId:AVGDATA logHiConc:-4.0

Id: 71608 Count Experiments: 2

Correlation: -0.24

JobId: 2893728923787660700

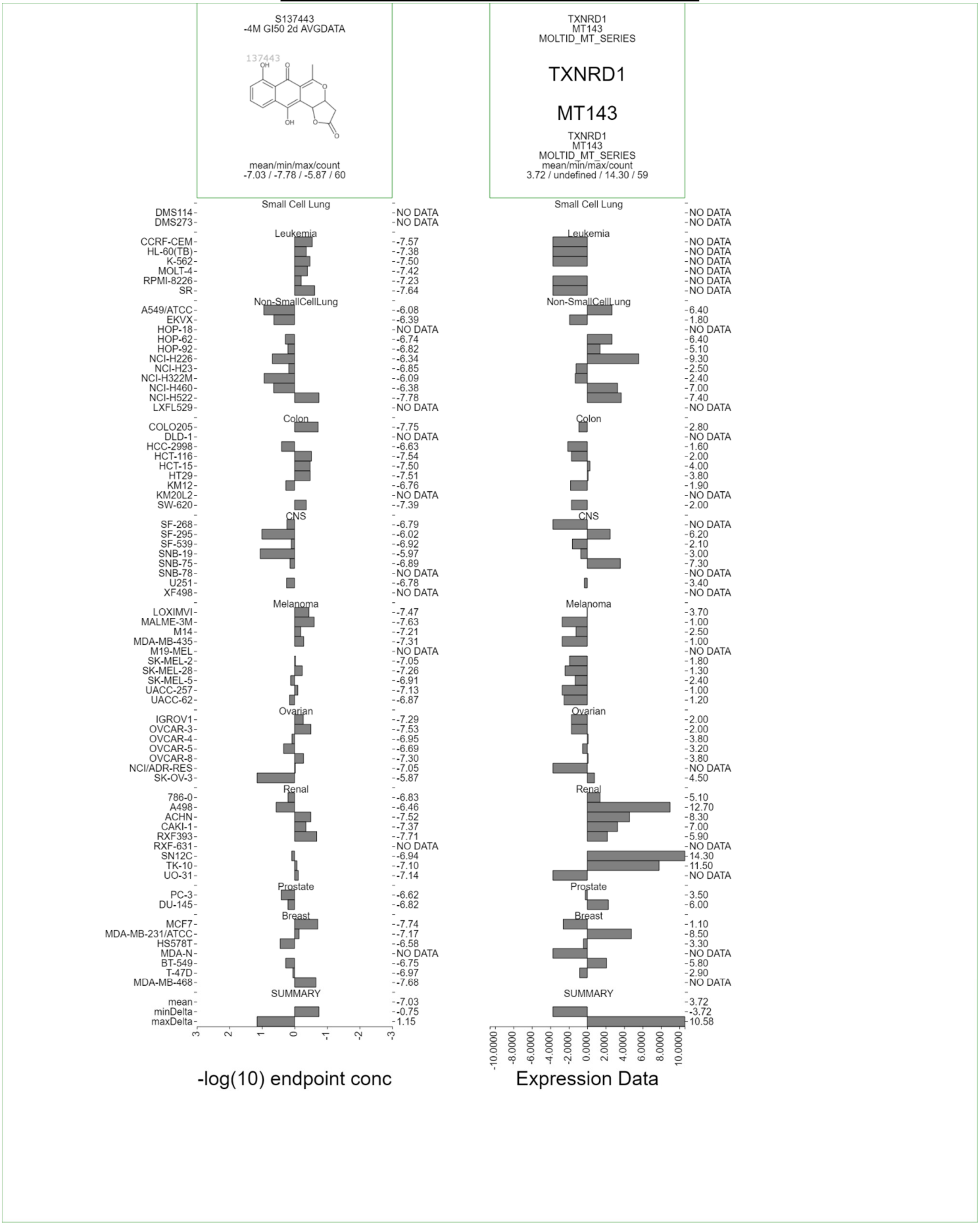

Figure S77

COMPARE Analysis of TrxR to Discorhabdin C (NSC626162)

---SEED---
MoltId:MT143 Genecard:TXNRD1 TargetSet:MOLTID_MT_SERIES
---TARGET---
NSC:S626162 Endpoint:GI50 ExpId:AVGDATA logHiConc:-4.0
Id: 421973 Count Experiments: 5

Correlation: -0.37
JobId: 3192043257499528905

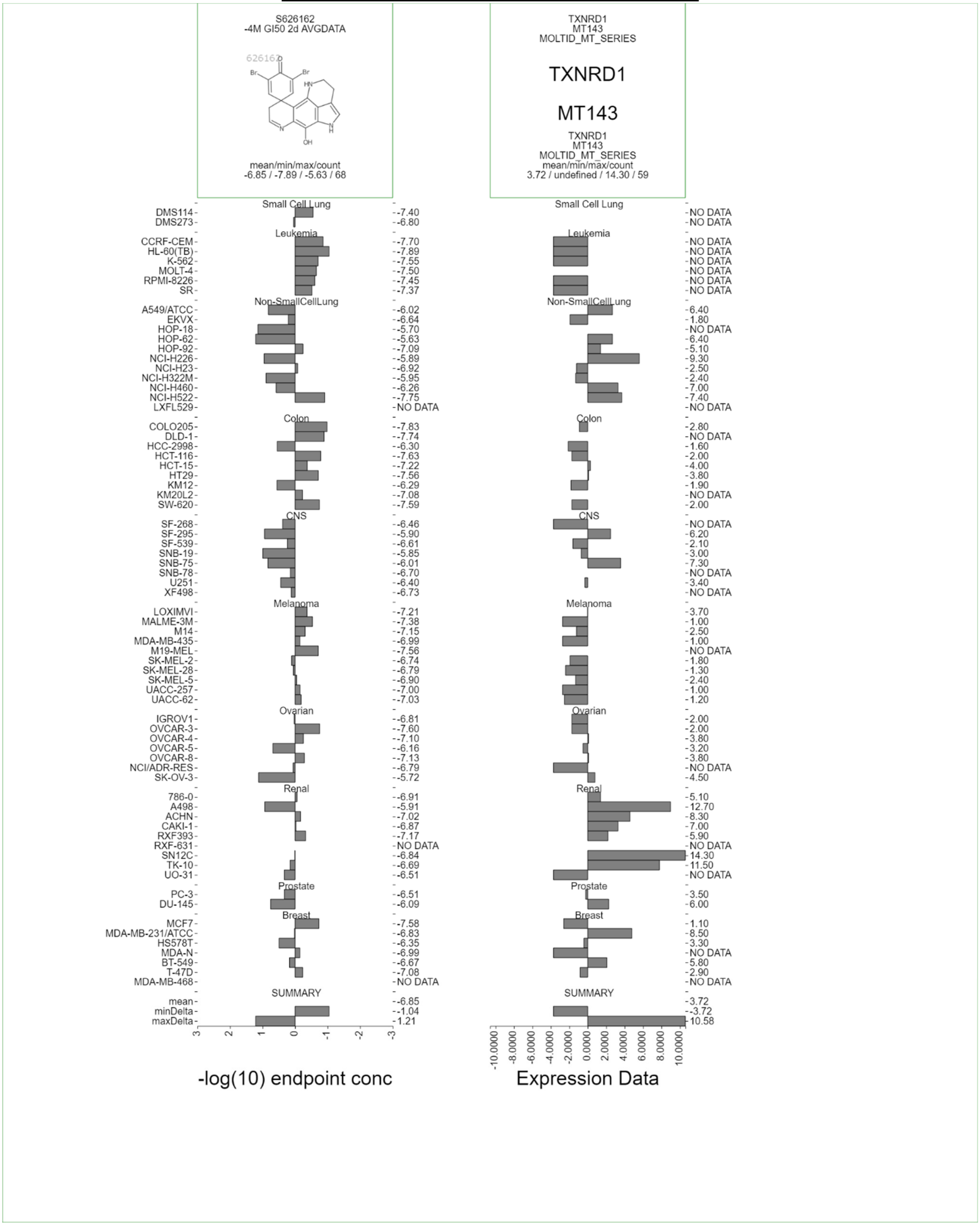

Figure S78

COMPARE Analysis of TrxR to DPIQ (NSC753790)

---SEED---
MoltId:MT143 Genecard:TXNRD1 TargetSet:MOLTID_MT_SERIES
---TARGET---
NSC:S753790 Endpoint:GI50 ExpId:AVGDATA logHiConc:-4.0
Id: 373263 Count Experiments: 2

Correlation: 0.12
JobId: 2313037008200469252

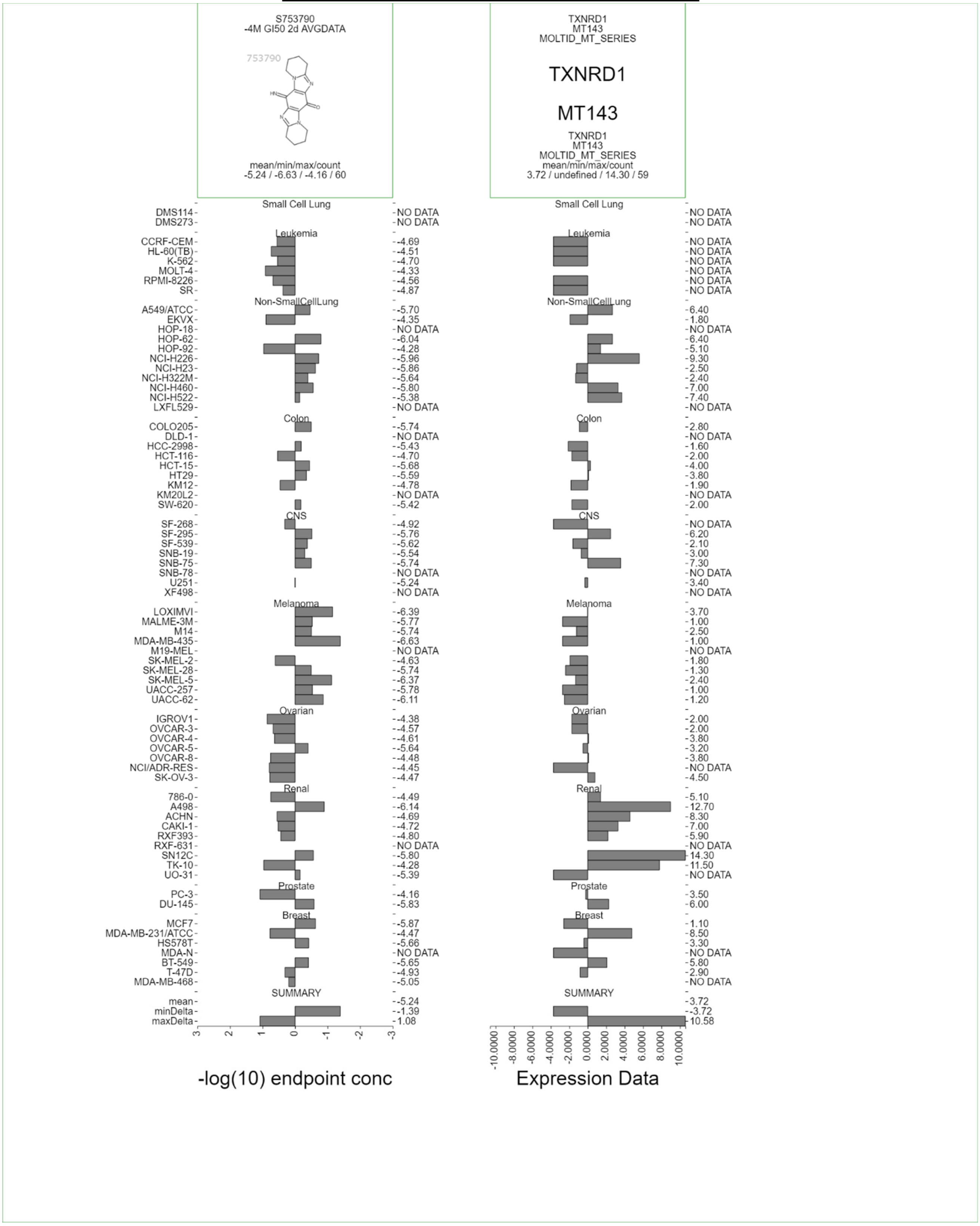

Figure S79

COMPARE Analysis of TrxR to **3a** (NSC84167)

---SEED---
MoltId:MT143 Genecard:TXNRD1 TargetSet:MOLTID_MT_SERIES
---TARGET---
NSC:S84167 Endpoint:GI50 ExpId:AVGDATA logHiConc:-4.0
Id: 372937 Count Experiments: 2

Correlation: 0.11
JobId: 6979304215872694200

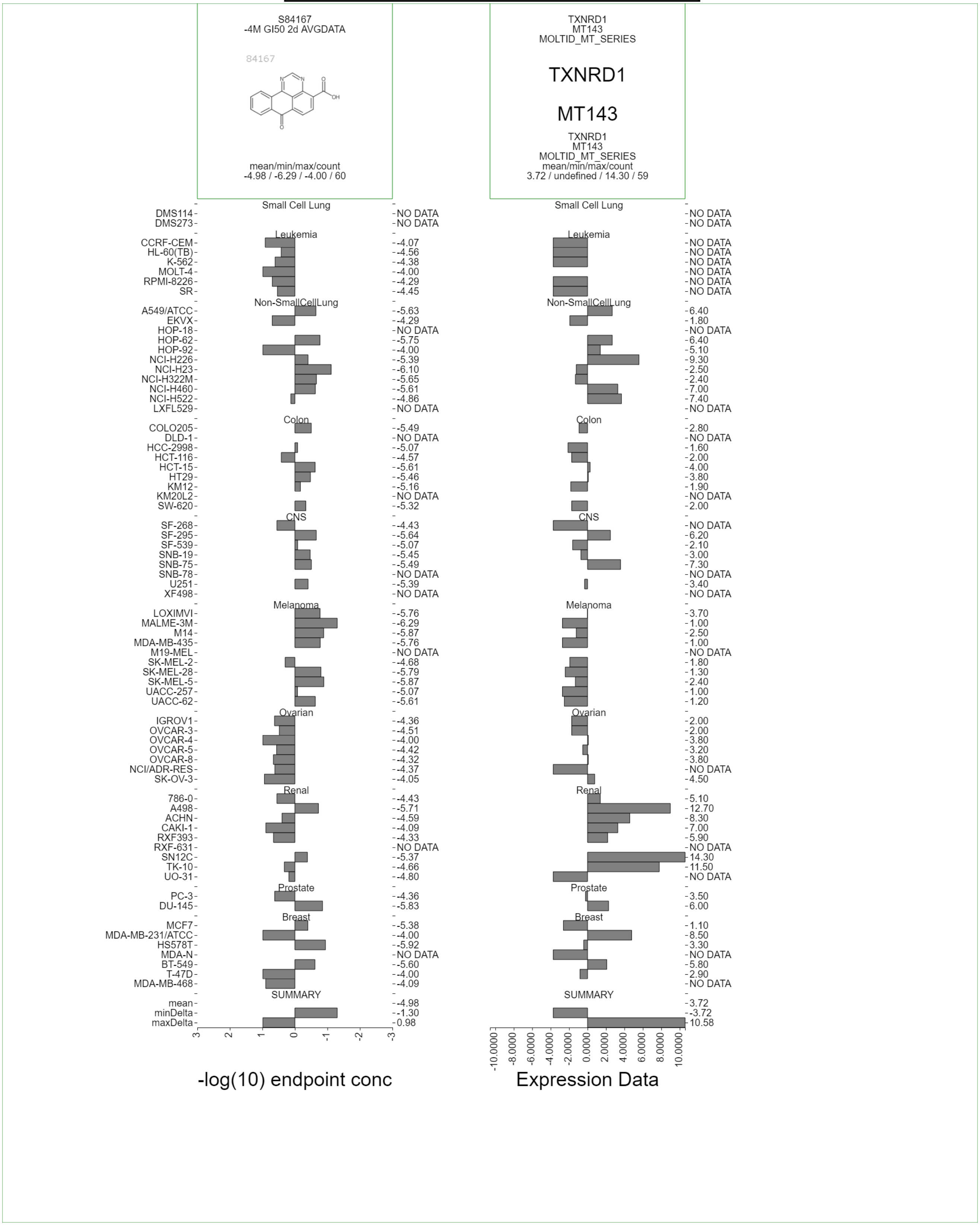

Figure S80

COMPARE Analysis of TrxR to **4** (NSC94945)

---SEED---

MoltId:MT143 Genecard:TXNRD1 TargetSet:MOLTID_MT_SERIES

---TARGET---

NSC:S94945 Endpoint:GI50 ExpId:AVGDATA logHiConc:-4.0

Id: 11715 Count Experiments: 2

Correlation: -0.15

JobId: 9112064582006927938

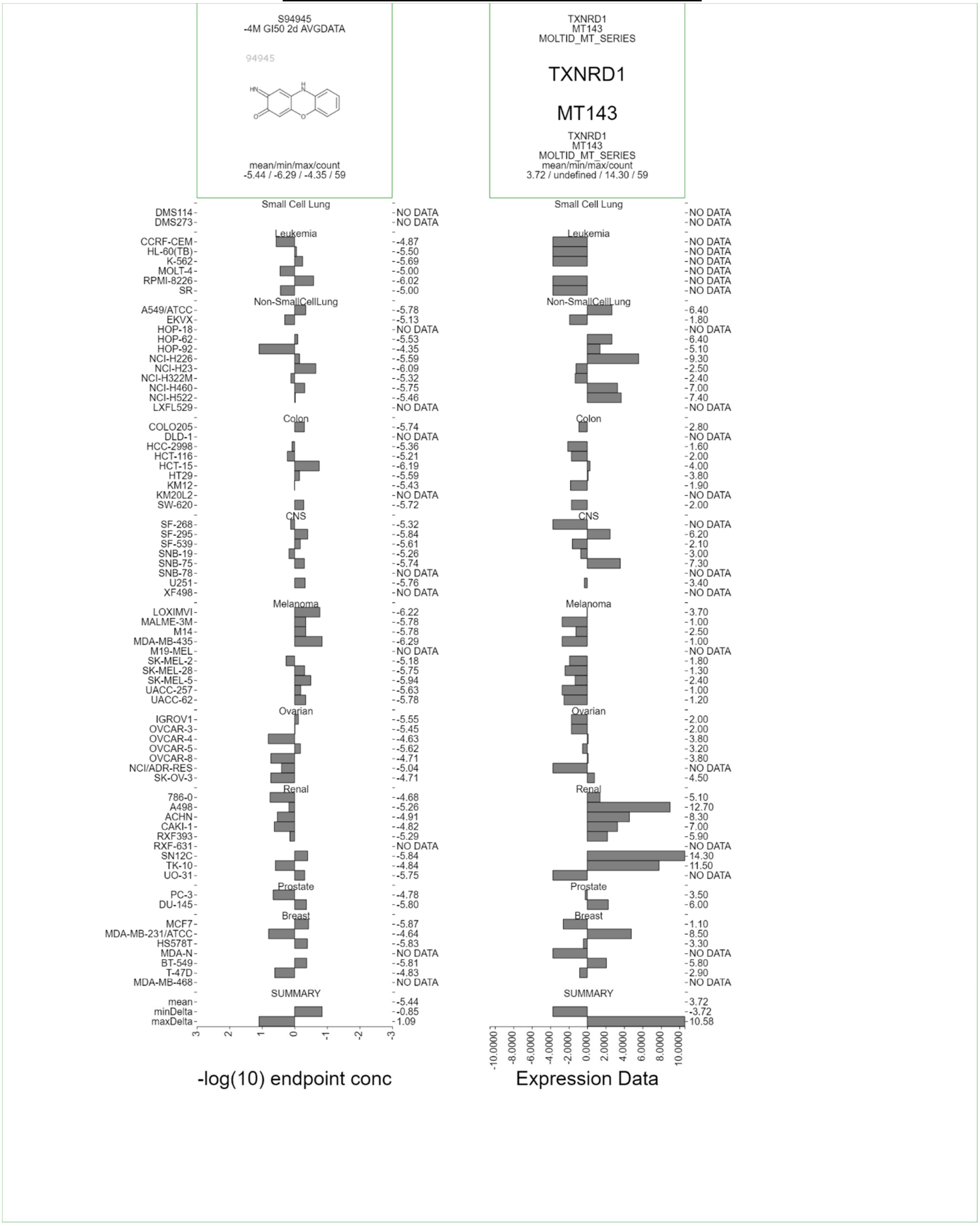

Figure S81

COMPARE Analysis of TrxR to **19** (NSC409304)

---SEED---

MoltId:MT143 Genecard:TXNRD1 TargetSet:MOLTID_MT_SERIES

---TARGET---

NSC:S409304 Endpoint:GI50 ExpId:AVGDATA logHiConc:-4.0

Id: 251495 Count Experiments: 1

Correlation: 0.10

JobId: 8407460412331887372

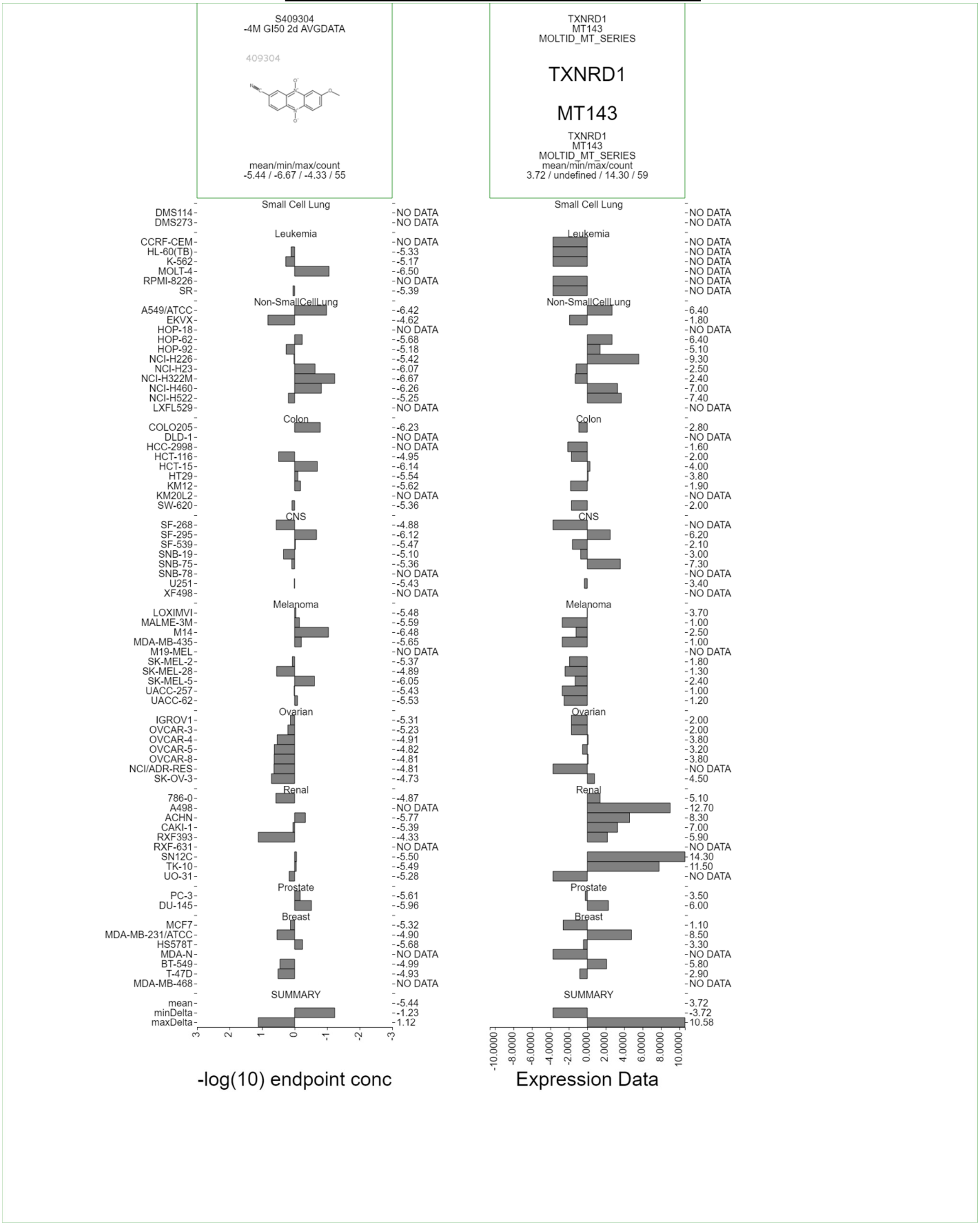

Figure S82
